# Feasibility study of single-image super-resolution scanning system based on deep learning for pathological diagnosis of oral epithelial dysplasia

**DOI:** 10.3389/fmed.2025.1550512

**Published:** 2025-03-12

**Authors:** Zhaochen Liu, Peiyan Wang, Nian Deng, Hui Zhang, Fangjie Xin, Xiaofei Yu, Mujie Yuan, Qiyue Yu, Yuhao Tang, Keke Dou, Jie Zhao, Bing He, Jing Deng

**Affiliations:** ^1^Department of Stomatology, The Affiliated Hospital of Qingdao University, Qingdao, China; ^2^School of Stomatology, Qingdao University, Qingdao, China; ^3^Qingdao Hiser Hospital Affiliated of Qingdao University (Qingdao Traditional Chinese Medicine Hospital), Qingdao, China; ^4^Department of Pathology, The Affiliated Hospital of Qingdao University, Qingdao, China; ^5^Dakewe (Shenzhen) Medical Equipment Co., Ltd., Shenzhen, China; ^6^Dental Digital Medicine and 3D Printing Engineering Laboratory of Qingdao, Qingdao, China

**Keywords:** oral epithelial dysplasia, deep learning, pathological diagnosis, digital pathology, artificial intelligence

## Abstract

This study aimed to evaluate the feasibility of applying deep learning combined with a super-resolution scanner for the digital scanning and diagnosis of oral epithelial dysplasia (OED) slides. A model of a super-resolution digital slide scanning system based on deep learning was built and trained using 40 pathological slides of oral epithelial tissue. Two hundred slides with definite OED diagnoses were scanned into digital slides by the DS30R and Nikon scanners, and the scanner parameters were obtained for comparison. Considering that diagnosis under a microscope is the gold standard, the sensitivity and specificity of OED pathological feature recognition by the same pathologist when reading different scanner images were evaluated. Furthermore, the consistency of whole-slide diagnosis results obtained by pathologists using various digital scanning imaging systems was assessed. This was done to evaluate the feasibility of the super-resolution digital slide-scanning system, which is based on deep learning, for the pathological diagnosis of OED. The DS30R scanner processes an entire slide in a single layer within 0.25 min, occupying 0.35GB of storage. In contrast, the Nikon scanner requires 15 min for scanning, utilizing 0.5GB of storage. Following model training, the system enhanced the clarity of imaging pathological sections of oral epithelial tissue. Both the DS30R and Nikon scanners demonstrate high sensitivity and specificity for detecting structural features in OED pathological images; however, DS30R excels at identifying certain cellular features. The agreement in full-section diagnostic conclusions by the same pathologist using different imaging systems was exceptionally high, with kappa values of 0.969 for DS30R-optical microscope and 0.979 for DS30R-Nikon-optical microscope. The performance of the super-resolution microscopic imaging system based on deep learning has improved. It preserves the diagnostic information of the OED and addresses the shortcomings of existing digital scanners, such as slow imaging speed, large data volumes, and challenges in rapid transmission and sharing. This high-quality super-resolution image lays a solid foundation for the future popularization of artificial intelligence (AI) technology and will aid AI in the accurate diagnosis of oral potential malignant diseases.

## 1 Introduction

Oral potential malignant diseases (OPMDs) are a general term for a class of diseases with the potential for malignant transformation occurring in the oral mucosal membrane, including oral leukoplakia, oral erythema, oral submucous fibrosis, etc. Current research shows that the overall malignant transformation rate of OPMDs is between 2.6 and 7.9% (1–5). Once OPMDs become malignant into oral squamous cell carcinoma, the patient’s 5-year survival rate and quality of life are significantly reduced. Pathological diagnosis often directly guides clinical intervention measures in the management of OPMDs patients. Oral epithelial dysplasia (OED) is the primary predictor of malignant transformation risk. The gold standard for tumor discrimination and grading is histopathological examination (6, 7). However, due to the diversity of pathological manifestations of OED and the subjective differences among pathologists, the diagnostic consistency for OED grading is low (8–10). Considering these issues in the pathological diagnosis of OED, numerous studies have indicated that consulting different pathologists on challenging cases can enhance the accuracy of the diagnosis (11, 12). Furthermore, with the advancement of computer technology, AI-assisted diagnosis has emerged as a prominent research focus in the pathological diagnosis of OED. Adeoye et al. (13) developed an OED malignant transformation prediction system that integrates clinical photographs of oral leukoplakia with deep learning algorithms, which achieved favorable calibration and discrimination. However, researchers have argued that this system cannot replace conventional biopsy for oral leukoplakia assessment. Consequently, recent investigations have explored the integration of digital pathology techniques, advanced imaging technologies, and deep learning methodologies to achieve precise diagnosis of OED (14).

Digital pathology technology utilizes advanced digital scanners to capture slices, transforming slice information into digital data that can be stored, analyzed, and shared. This advancement significantly enhances the efficiency of remote consultations between hospitals and establishes the groundwork for using AI technology in OED diagnosis (15, 16). The digital scanning of slices is an important aspect of the application of digital pathology technology. However, traditional scanning technologies encounter several problems. When a high-magnification objective lens is used to scan clear, high-resolution images, the scanning efficiency, equipment stability, and single-scanning success rate of traditional scanning systems must be improved. In addition, the large storage capacity of digital images and the high cost of scanning equipment limit the use of digital slice scanning and remote diagnosis in primary medical institutions and pose a great burden to individuals or small and medium-sized scientific research teams. To meet these challenges and make the obtained higher-definition cell microstructure images easy for clinical application, many optical super-resolution technologies have been developed, such as structured illumination microscopy, photoactivated localization microscopy (17), and sub-diffraction-limit imaging using the stochastic optical reconstruction microscopy technique (18). Single-image super-resolution (SISR) technology, an image-transformation method, reconstructs high-resolution (HR) images from degraded low-resolution (LR) images (19). Recently, SISR technology, enhanced by deep learning, has been extensively utilized by researchers in digital pathology and clinically verified. This technology has been applied to the pathological diagnosis of leiomyosarcoma of the ovary and uterus. It was confirmed that both whole and local features were preserved during the reconstruction of high-resolution images using deep learning. Additionally, 20× whole slide images (WSIs) were converted into 40× WSIs (20). By maintaining image clarity, super-resolution scanning technology significantly accelerates scanning speeds compared to previous technologies, facilitating the widespread adoption and application of digital pathology in clinical settings (21). In this study, we integrated a digital pathological scanner with super-resolution technology and developed a digital slice optical scanner using an array objective lens (DS30R DAKEWE). We utilized images of OED slides as training data for deep learning. By combining it with SISR technology, the OED slide imaging algorithm was established. Given the pathological characteristics of oral tissues, this integration enables clearer and more detailed microscopic imaging, enhancing the accuracy of observations of dense cells in oral epithelial tissues and their nuclear morphological features.

## 2 Materials and methods

### 2.1 Dataset

This is a cross-sectional diagnostic study in which the dataset included 240 hematoxylin and eosin (H&E) stained WSIs of OPMDs with biopsy-proven dysplasia and clinical presentation as homogeneous and non-homogeneous leukoplakias at one or more sites, diagnosed between 2018 and 2023. This study adhered to the Declaration of Helsinki and received approval from the ethics committee of The Affiliated Hospital of Qingdao University (registration number: QDFYWZLL29058). All patients were required to have at least 1 year of follow-up, with comprehensive photography and documentation. Patients with oral lichen planus were excluded, while those presenting with simple hyperplasia and mild, moderate, or severe dysplasia/carcinoma *in situ* were included (7). All pathological sections have previously passed quality control checks. The fixation time for the samples was uniformly maintained between 6 to 24 h, and the thickness of all sections consistently measured within the range of 3-5 μm. The slides were scanned and stored using a DS30R scanner and a Nikon scanner. Of these materials, 40 slides were utilized for super-resolution machine training, and 200 slides were used for diagnosis and verification. The proposal super-resolution deep learning model is deployed locally on the DS30R product, without connecting to the Internet or being accessible from the Internet. The pathology slides used for training and evaluation data are sourced from the Affiliated Hospital of Qingdao University. The digital images are stored in a private storage of the Affiliated Hospital of Qingdao University. The storage and use of digital images are conducted in an environment with data network security and under the authorization of relevant regulations. The super-resolution deep learning model and software system in the DS30R comply with the standards of ICE/TR 80001–2-2 and ISO/IEC 81001–5-1, as well as the YY/T 1843–2022 standard.

### 2.2 Imaging optimization of super-resolution scanning system based on deep learning

We used the SISR technology based on the transition from LR image to HR image to realize super-resolution imaging. Based on SISR framework, this technology used multiscale fully convolutional networks and conditional generative adversarial networks. Through stochastic curriculum learning training strategies, the complexity of data pairs was gradually increased in the training stage to urge the model to capture more complex image details, thus achieving the imaging clarity equivalent to 20× NA0.8 objective lens.

#### 2.2.1 Collection and preparation of super-resolution image data set

Forty oral tissue slides were scanned using a DS30R scanner equipped with a 20× array objective lens to generate low-resolution WSIs. Comparative high-resolution acquisitions were performed through a Nikon scanner under dual configurations: 20× NA0.4 optical parameters (designated HR01) and 20× NA0.8 specifications (designated HR02). The resultant dataset comprising 150,000 LR-HR01-HR02 triplets was partitioned through stratified randomization into training (*n* = 120,000), testing (*n* = 15,000), and validation (*n* = 15,000) subsets, maintaining an 8:1:1 allocation ratio.

HR01 images, acquired through the 20× NA0.4 objective lens, served as the ground-truth reference for establishing baseline optical parameters. These were paired with array-derived LR images to computationally model a point spread function (PSF) and imaging resolution parameters equivalent to conventional optical microscopy. Subsequent super-resolution learning employed stochastic curriculum learning (SCL) with progressive difficulty escalation, wherein HR02 images (20× NA0.8 acquisitions) were integrated as advanced training targets to enhance imaging fidelity.

Two distinct training paradigms were implemented:

**Dataset A**: HR01 (20× NA0.4) ↔ LR (array-based) mapping.

**Dataset B**: HR02 (20× NA0.8) ↔ LR (array-based) mapping.

**Figure 1:**
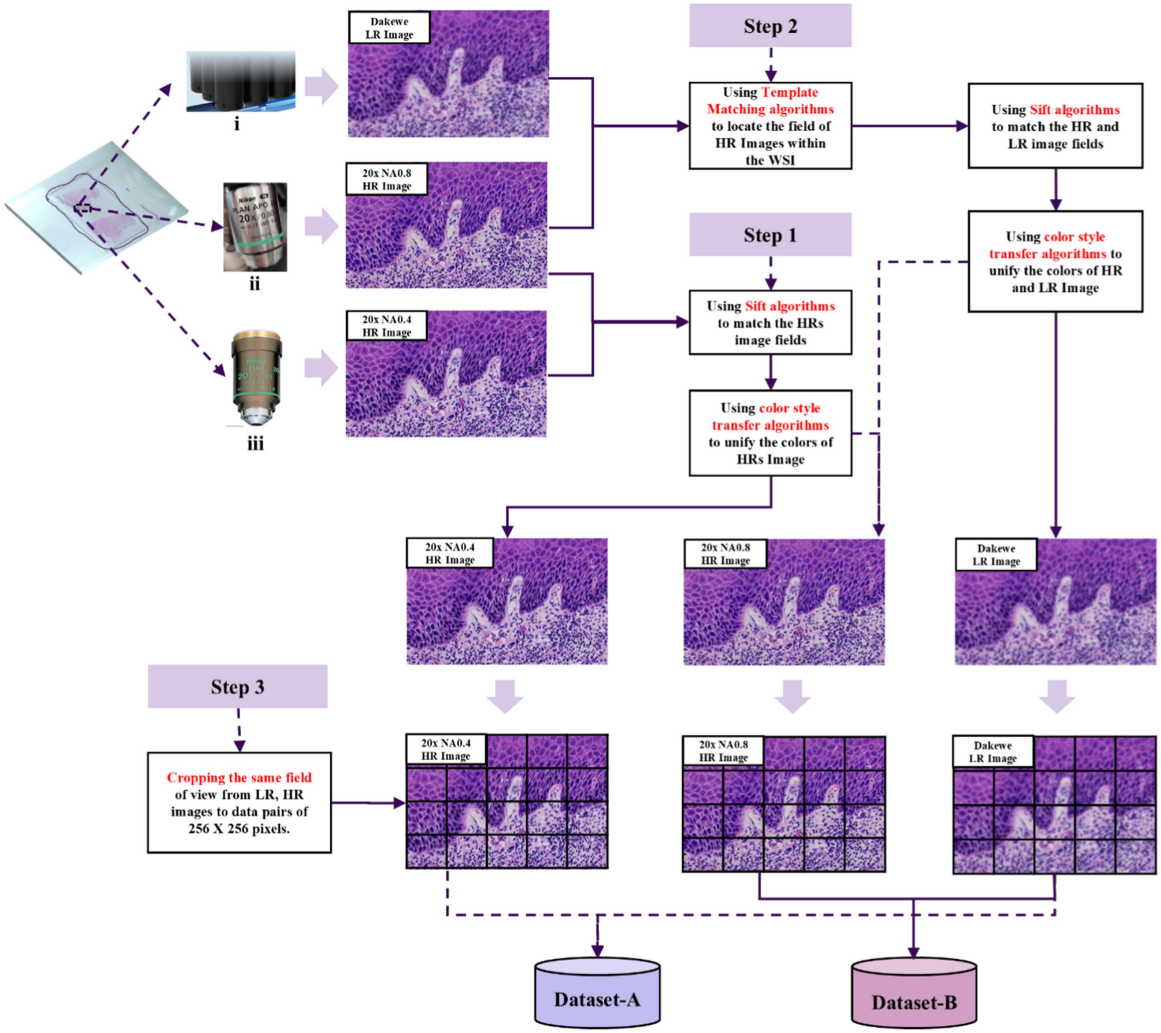
Preparation process of super-resolution data set. Image data were acquired from H&E-stained oral pathology slides using three objectives: (i) DAKEWE DS30R array objective 20×, (ii) NIKON CFI Plan Apochromat 20× NA0.8, and (iii) NIKON CFI Plan Achromat 20× NA0.4. Step 1 Cross-high-resolution registration: Scale-Invariant Feature Transform (SIFT) feature matching achieved subpixel alignment between HR01 and HR02 (error < 1px), with adaptive color normalization to minimize brightness differences (Δ grayscale ≤ ±2). Step 2 Cross-scale precise registration: A novel combination of Template Matching and SIFT algorithms aligned low-resolution WSI to HR02 (error < 1px), followed by adaptive color normalization (Δ grayscale ≤ ±2). Step 3 Dataset creation: Cropped images to 256 × 256 pixels, generating two datasets: A (LR-HR01 pairs) and B (LR-HR02 pairs).

Critical implementation consideration: Spatial registration between Datasets A and B was rigorously maintained through field-of-view alignment, effectively mitigating catastrophic forgetting during incremental model training. The flow chart of the collection and preparation of super-resolution image data sets is shown in Figure 1.

#### 2.2.2 Super-resolution model training

In the OED slide dataset, the scaling factor between the LR and HR images is 6, and SISR with this and other large scaling factors presents significant challenges. Consequently, adopting a coarse learning mechanism proves advantageous for training the SISR model to handle large scaling factors. Initially, the model is trained using simple samples, and progressively, more complex samples are incorporated into the training set. This strategy enhances the model’s ability to restore low-resolution images to high-resolution ones. Therefore, utilizing the coarse-learning mechanism, we segmented the model training process into two distinct phases: Stage 1: Training begins on an untrained model using Dataset-A, focusing on super-resolution training in deep learning to match the imaging clarity of 20 × NA0.4. Stage 2: Building on the model trained in the first stage, further super-resolution training is conducted using Dataset-B, aiming to achieve imaging clarity of 20 × NA0.8 and meet diagnostic definition requirements.

### 2.3 Super-resolution scanning system based on deep learning for OED pathological image feature recognition and full-slide diagnosis evaluation

#### 2.3.1 Recognition of pathological features of OED images

The OED pathological tissue samples from our slide database were randomly selected based on their positions within the epithelium. Images in the same field of vision were captured using a DS30R scanner, Nikon scanner, and optical microscope, comprising 200 low magnification and 200 high magnification images. The images were categorized into groups A (DS30R 10×), B (Nikon 10×), C (optical microscope 10×), D (DS30R 20×), E (Nikon 20×), and F (optical microscope 20×). Groups A, B, and C focused on identifying structural features, while groups D, E, and F targeted cellular features. Upon acquiring the images, a senior oral pathologist randomly reviewed and annotated each group based on the pathological features defined in the WHO classification standard (2022) for OED (22). The results from the optical microscope groups (C and F) served as the gold standard. Subsequently, the sensitivity and specificity of feature recognition in groups A, B, D, and E were calculated. The calculation formulas for sensitivity and specificity are as follows:


$$\text{Sensitivity}=\frac{\text{True Positives}}{\text{True Positives}+\text{False Negatives}}\times 100\%$$

$$\text{Specificity}=\frac{\text{True Negatives}}{\text{True Negatives}+\text{False Positives}}\times 100\%$$

These metrics represent fundamental statistical parameters, where:

True Positives = number of correctly identified positive features.

False Negatives = number of incorrectly identified negative features.

True Negatives = number of correctly identified negative features.

False Positives = number of incorrectly identified positive features.

#### 2.3.2 Evaluation of pathological full-slide diagnosis of OED

A senior oral pathologist successively browsed and read the whole sections using a DS30R scanner, a Nikon scanner, and an optical microscope. The order of each section was randomized. The pathologist arrived at the diagnostic conclusion for each slide and recorded it. All slide samples were grouped according to the original diagnostic conclusion, and the agreement rate between the diagnostic conclusions of the pathologists in each group and the original diagnostic conclusion was calculated. The formula used is as follows:


$$\text{Agreement Rate}=\frac{\begin{array}{l} \text{Number of slides with the same}\;\\ \text{diagnosis within the group} \end{array}}{\begin{array}{l} \text{Total number of slides}\;\\ \text{contained in the group} \end{array}}\times 100\%$$

The consistency of the diagnoses made by pathologists using the three instruments was compared, with DS30R-optical microscope, DS30R-optical microscope, Nikon-DS30R and three evaluators (23, 24). The results were consistent by Kappa test.

### 2.4 Statistical analysis

The collected data were analyzed using the Statistical Package for the Social Sciences (SPSS) for Windows, version 22.0. The weighted kappa test was used for the consistency test between two, and Fleiss’ kappa test was used for the consistency test between three. When the Kappa coefficient is greater than 0, it indicates meaningful consistency. A larger Kappa coefficient denotes better consistency. A Kappa coefficient of 0.00–0.20 indicates low consistency, 0.21–0.40 indicates general consistency, 0.41–0.60 indicates moderate consistency, 0.61–0.80 indicates high consistency, and 0.81–1.00 also indicates very high consistency.

## 3 Results

### 3.1 Imaging optimization of super-resolution scanning system based on deep learning

When preparing the super-resolution dataset, we used the DS30R and Nikon scanners to scan the same slice data and save them as digital data. The operating parameters of the two scanning devices are listed in Table 1.

**Table 1:** Performance comparison between DS30R and Nikon scanners.

Parameter	DS30R	NIKON
Scanning mode	Microscope Array Scanning	Area Image Scanning
Imaging mode	Bright Field	Bright Field
Imaging resolution	0.179 um/pixel	0.24 um/pixel
Scanning speed*	0.25 min	15 min
Storage capacity*	0.35 GB/WSI	0.5 GB/WSI
Multi-layer scanning	Supportive	Unsupportive
Auto-focusing	Supportive	Supportive

Scanning speed*, Scanning capacity*: based on a full slice with a scanning range of 15 mm*15 mm.

**Figure 2:**
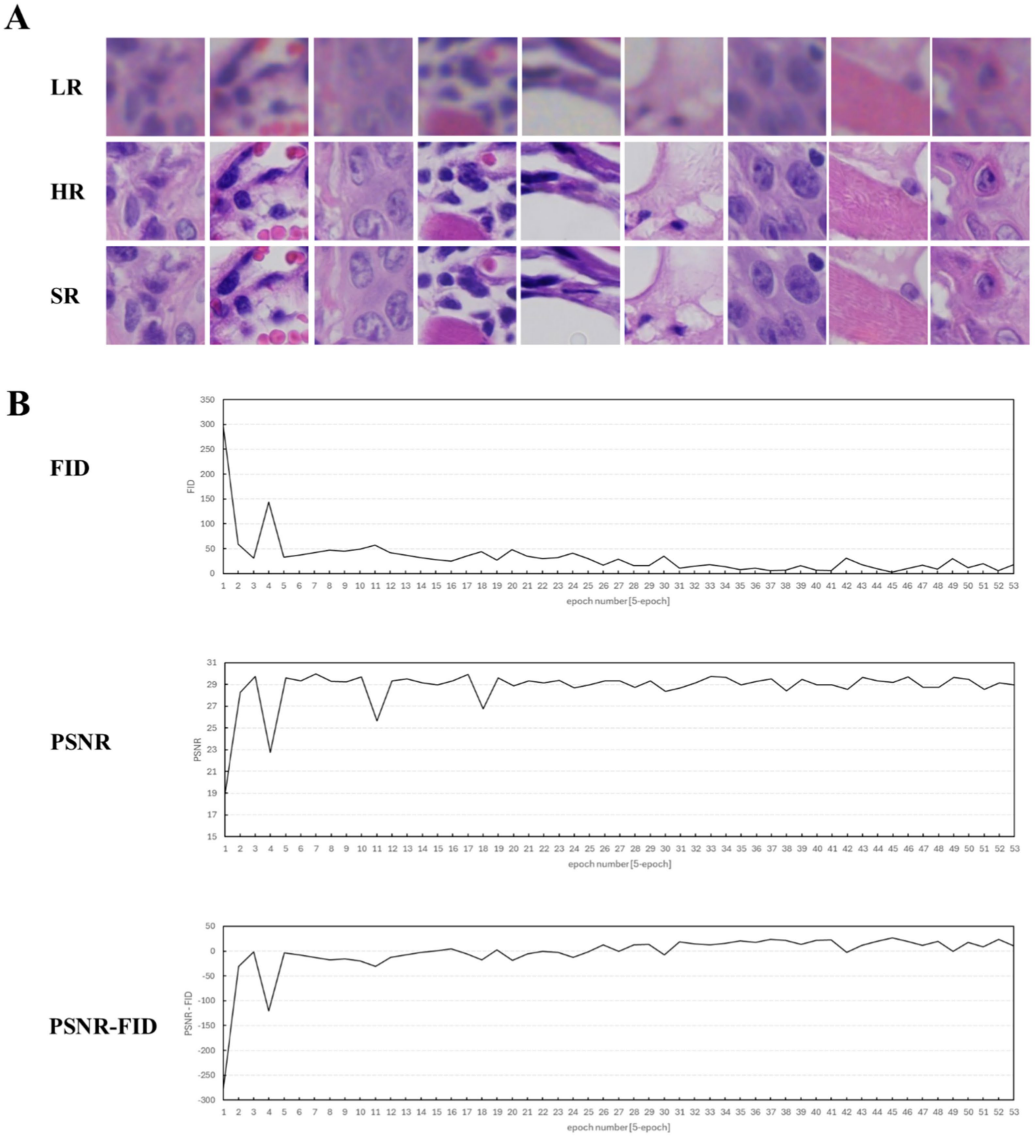
Display of training process data and key image results (epoch = 1–165). **(A)** Key H&E-stained image results of the training. LR, the original image captured by the DS30R array objective lens. HR, the image captured by the microscope using a Nikon 20× NA0.8 objective lens. SR, the super-resolution image, which is obtained by processing the LR image through the super-resolution deep learning model. **(B)** Evaluation curves of the training results during the super-resolution model training process. The evaluation metrics selected FID, PSNR, and the difference between FID and PSNR.

The DS30R scanner can automatically focus the slide and scan it in single or multiple layers through parallel array scanning. It requires 0.25 min to scan a slide in a single layer, with the data storage volume for the entire slide at 0.35GB. A Nikon scanner was used for area scanning, taking 15 min per slice, with a storage volume of 0.5GB. In the deep learning framework, the critical hyperparameters were configured as follows: the model underwent 900 training epochs with a generator learning rate of 1 × 10^−4^ and a discriminator learning rate of 1 × 10^−5^, while the adversarial loss coefficient was maintained at 0.9 throughout the training process. We employed two metrics, the peak signal-to-noise ratio (PSNR) and Fréchet inception distance (FID), to assess the model’s performance and produce a super-resolution image, as depicted in Figure 2. Throughout the training, we examined the image data and determined that the PSNR primarily assessed the image clarity, whereas the FID gaged the image fidelity. In the SISR model training, these two metrics produced divergent outcomes; the peak PSNR did not guarantee the lowest FID. This divergence arises because the PSNR measures only image clarity and not fidelity. An overly high PSNR may indicate that an image is either too smooth or distorted, leading to a feature distribution that does not align with the actual image, thereby elevating the FID. To generate a comprehensive evaluation index that considers both definition and fidelity, we introduced the PSNR-FID difference. A greater difference suggests that the model output is more balanced in terms of definition and fidelity, offering a more thorough evaluation of the model’s performance. Pivotal images from the SISR model training are illustrated in Figure 2A. After super-resolution training, the images became clearer, and intracellular granularity was enhanced. As shown in Figure 2B, the deep learning model underwent progressive optimization (manifested by a reduction in the FID metric) and enhanced processing performance (demonstrated by an elevation in the PSNR values) during training, ultimately stabilizing at equilibrium. The maximal discrepancy between the PSNR and FID metrics served as the determinant for optimal training termination, thereby achieving an optimal equilibrium between the model optimization (fidelity preservation) and processing efficacy (output clarity). The full dataset is available in the Supplementary material (Sections S1–S3).

### 3.2 Super-resolution scanning system based on deep learning for OED pathological image feature recognition and full-slide diagnostic evaluation

**Figure 3:**
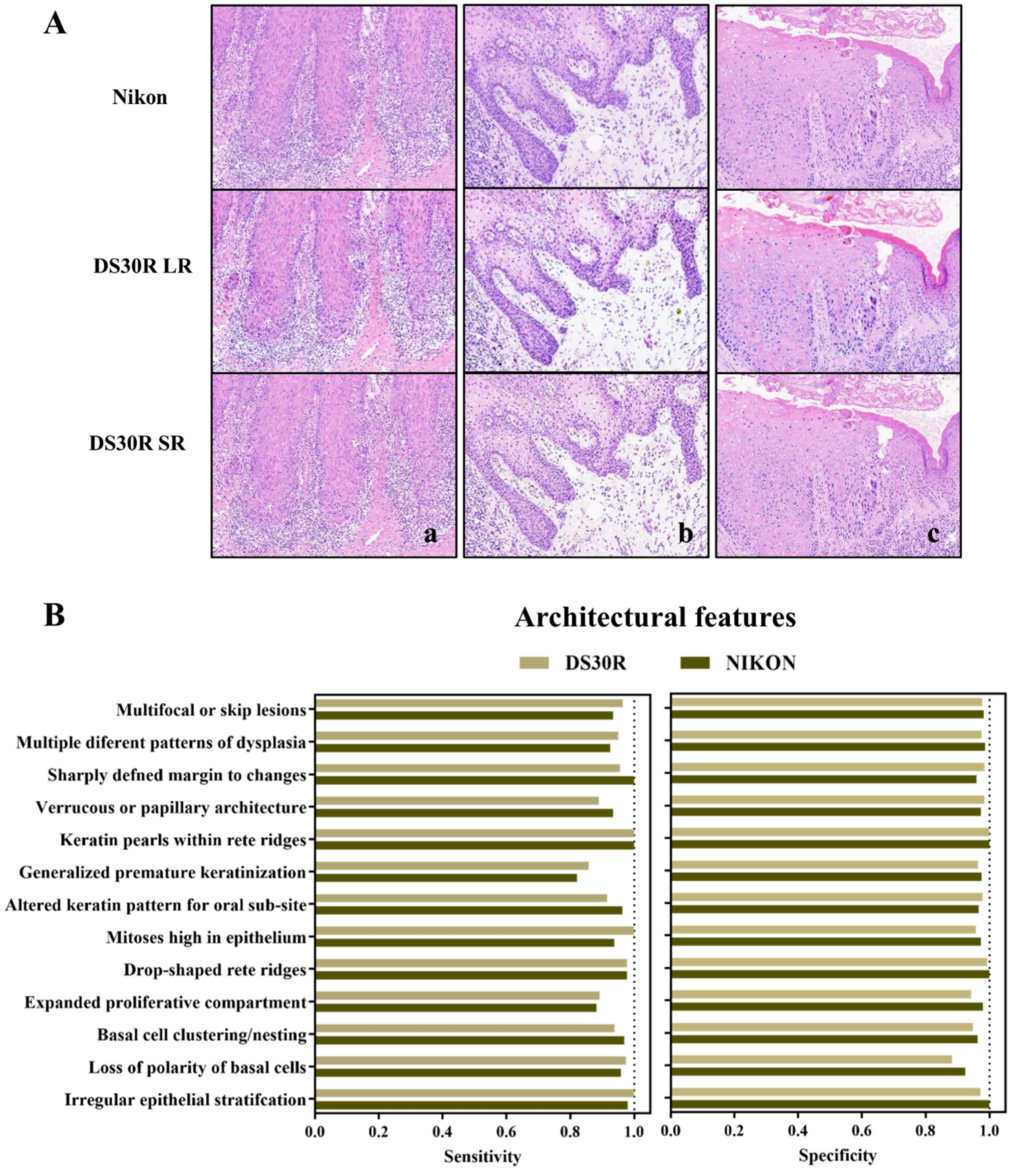
Structure characteristics in OED slides. **(A)** Examples of OED structural feature images: (a) irregular epithelial stratification, (b) drop-shaped rete ridges, and (c) altered keratin pattern for oral sub-site. Images at 10× magnification were obtained from H&E-stained tissue sections scanned using DS30R and Nikon. **(B)** Sensitivity and specificity of structural features recognition.

**Figure 4:**
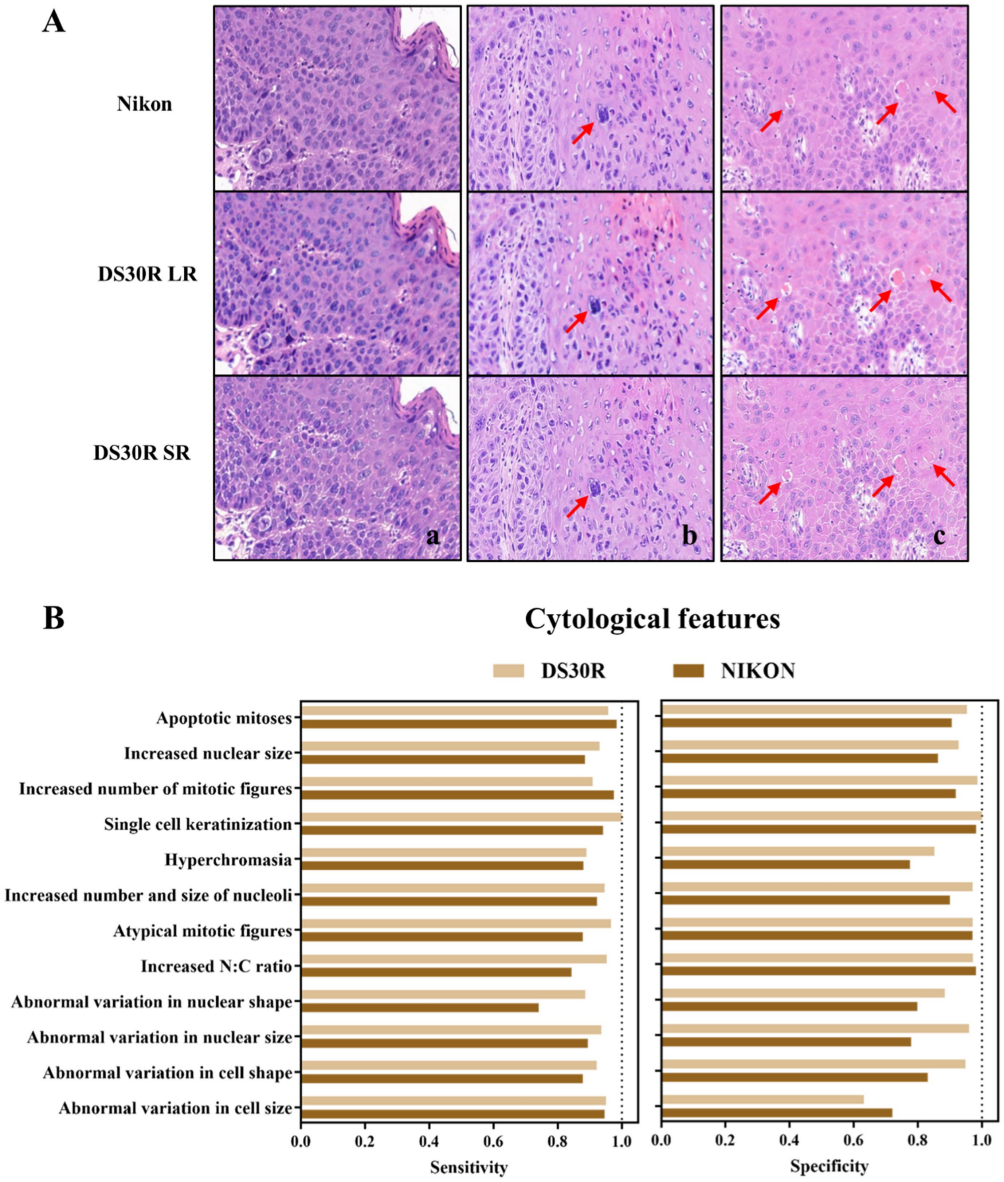
Cell morphological characteristics in OED slides. **(A)** Examples of OED cell feature images: (a) increased N/C ratio, (b) atypical mitotic figures, (c) single cell keratinization. Images at 20× magnification were obtained from H&E-stained tissue sections scanned using DS30R and Nikon. **(B)** Sensitivity and specificity of cell morphological characteristics recognition.

Oral pathologists examined the characteristic pathological images of each group and identified the information they contained. As shown in Figure 3A, the histopathological analysis of OED lesions revealed characteristic architectural disturbances. Figure 3Aa shows the disorganization of epithelial stratification accompanied by the loss of basal cell polarity and irregular cellular alignment. Figure 3Ab illustrates the well-defined epithelial drop-shaped rete ridges, which are a hallmark of early dysplastic progression. Figure 3Ac shows abrupt transition zones between orthokeratotic and parakeratotic keratinization patterns, indicative of advanced epithelial maturation abnormalities. Notably, these diagnostic morphological signatures were preserved with high fidelity following computational enhancement using the DS30R super-resolution imaging system. Moreover, the color information of the H&E-stained slices was closer to the actual values. In the structural feature group, as illustrated in Figure 3, the sensitivity and specificity of the feature recognition were high, particularly when the specificity exceeded 95%. The sensitivity and specificity of image recognition using the DS30R and Nikon scanners closely matched. Detailed data are presented in Supplementary Table S1. Figure 4A shows the three characteristic cellular features observed in OED. Figure 4Aa shows an increased nuclear-to-cytoplasmic (N/C) ratio, accompanied by nuclear abnormalities, such as nucleolar enlargement and hyperchromasia, indicative of enhanced cellular proliferation and elevated mitotic activity. Figure 4Ab shows a distinct abnormal mitotic pattern with concomitant nuclear enlargement and chromatin condensation. Figure 4Ac shows multiple single-cell keratinization patterns, a hallmark of dysplastic epithelial differentiation. Initial low-resolution scanning using the DS30R system yielded suboptimal image clarity for these subtle cytological features compared to the Nikon scanner. However, following the application of super-resolution reconstruction algorithms optimized with a generative adversarial network loss function, the DS30R SR output exhibited significantly enhanced image quality. The processed images showed improved spatial resolution, optical translucency, and intracellular granularity. As shown in Figure 4, the sensitivity for the identification of cellular features was high, although the specificity was somewhat lower than that of the structural feature group. Specificity diminished when the size and morphology of the cells and nuclei were recognized. With the exception of cell size, the DS30R’s specificity for identifying cellular characteristics surpassed that of the Nikon scanner. Further details are provided in Supplementary Table S2.

**Figure 5:**
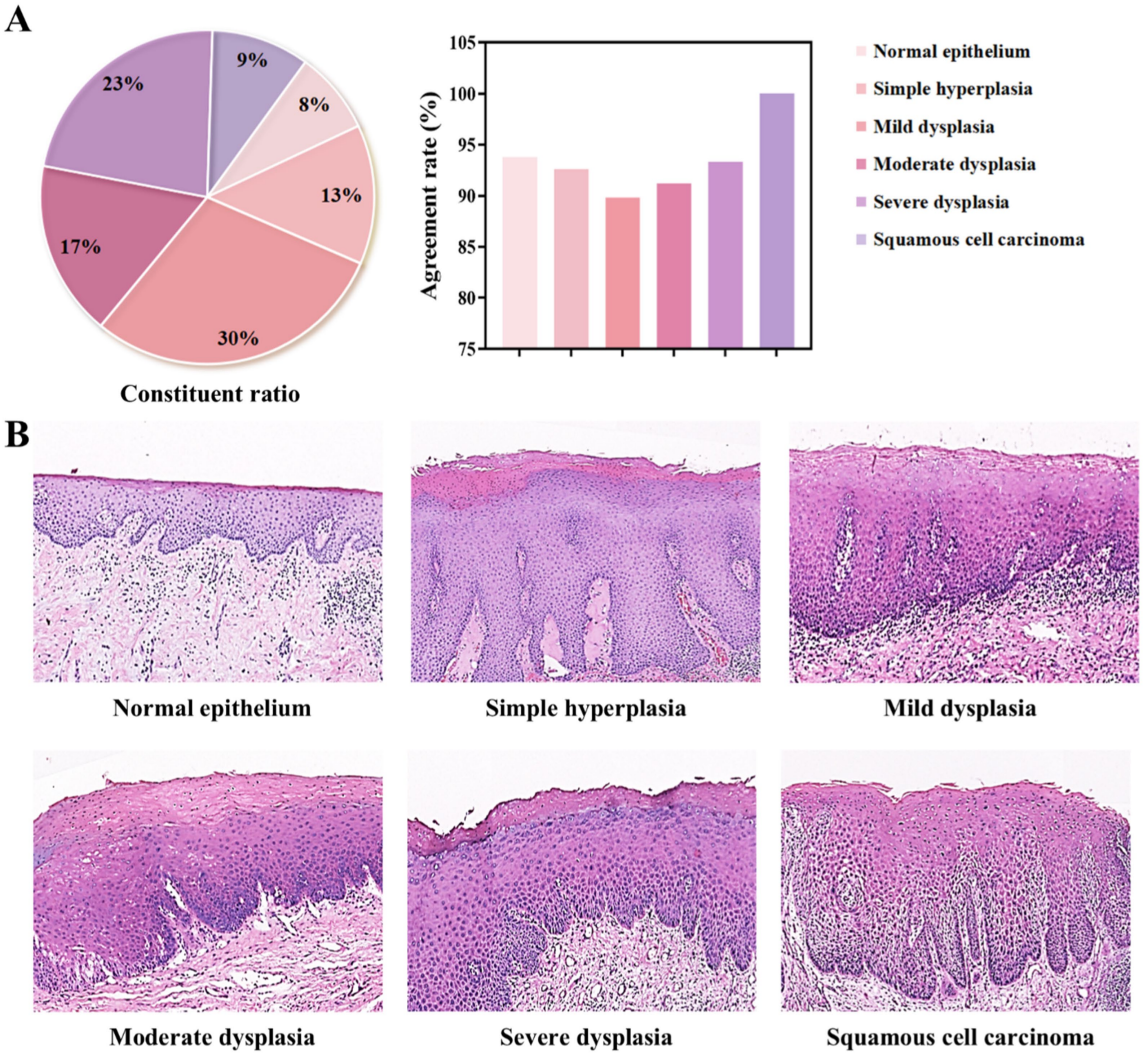
Classification of oral epithelial tissues with different grading of dysplasia degree. **(A)** Composition ratios of each diagnostic category and their agreement rates in diagnostic outcomes. **(B)** Representative pathological H&E-stained images for each category, all acquired using the DS30R scanner at 10× objective magnification.

Based on the original diagnoses, the 200 oral histopathological sections selected in this study were classified as normal epithelium, simple hyperplasia, mild dysplasia, moderate dysplasia, severe dysplasia, or squamous cell carcinoma (Figure 5A and Supplementary Table S3). The coincidence rates between the diagnostic conclusion under light microscopy and the historical diagnostic conclusion were as follows: normal, 93.8%; simple hyperplasia, 92.6%; mild dysplasia, 89.8%; moderate dysplasia, 91.2%; severe dysplasia, 93.3%; and cancer, 100%. The DS30R scanning images corresponding to each diagnosis are shown in Figure 5B. These images were generated using super-resolution technology after scanning with a 20× array objective lens using a DS30R pathological section scanner. This demonstrated the characteristic features of OED across different histopathological grades. The key diagnostic criteria include the extent of epithelial involvement due to dysplastic changes and the presence of localized cellular atypia, such as nuclear pleomorphism, loss of polarity, and abnormal mitotic activity. These features are critical for distinguishing mild, moderate, and severe dysplasia. For example, in Figure 5B, normal or hyperplastic epithelial tissues showed no cellular or structural atypia. In mild dysplasia, atypical hyperplasia is confined to the lower third of the epithelial layer. Moderate dysplasia exhibits features extending to the middle third of the epithelial layer, whereas severe dysplasia demonstrates characteristics involving almost the entire epithelial layer and/or shows significant cellular atypia. The pathologists used a DS30R scanner, a Nikon scanner, and a light microscope to examine the entire section and establish a definitive diagnosis. The consistencies in the diagnoses made using the three methods were calculated and compared (Supplementary Table S4 and Section S4). The kappa values were 0.981 for the Nikon-optical microscope (*p* < 0.05), 0.969 for the DS30R-optical microscope (*p* < 0.05), and 0.988 for the Nikon-DS30R (*p* < 0.05). The consistency test among the three evaluators was conducted using Fleiss’ kappa test, with a value of 0.979 (*p* < 0.05), indicating very high consistency among the evaluators.

## 4 Discussion

To the best of our knowledge, this study is the first to use deep-learning-based super-resolution technology with the morphological features of oral epithelial tissues through methodological refinement for OED imaging. Employing H&E-stained slides of oral mucosal epithelial tissue for machine learning and continuously adjusting parameters, such as granularity, during training enhances image clarity and suitability for oral histopathological diagnosis. Our research confirmed that scanning the images of OED slides with this system effectively preserved the characteristics of tissue structure and cells. By incorporating a deep learning algorithm, the final generated image exhibits high sensitivity and specificity for identifying lesion features, particularly for imaging fine cellular structures. Compared with the regional scanning mode of the Nikon scanner, our system significantly enhanced the scanning speed and reduced the storage volume of the image files. These results indicate that the array objective scanning imaging system embodied by the DS30R combined with deep learning has significant application value in the histopathological diagnosis of OED.

To overcome the diffraction limit and achieve higher-resolution imaging of cellular microstructures, various microscopic cellular imaging techniques have been developed since 2000, including super-resolution technologies based on the structured illumination microscopy (SIM) principles (25) and photoactivated localization microscopy (PALM) developed by Betzig et al. (17), which utilize fluorescent molecules combined with proteins. However, these techniques require expensive optical equipment and involve complex procedures. The cellular imaging technology employed in this study adopts a pyramid architecture based on convolutional neural networks, referencing the SISR framework proposed by Li et al. in 2020 (26). This is one of the few clinically validated methodologies, and our findings demonstrate its applicability for the histopathological diagnosis of OED using H&E-stained slides.

Some limitations exist in this study. In OPMDs histopathological slides, epithelial tissue alterations such as inflammatory infiltration and ulceration - which frequently coexist with OED - may concurrently influence cellular and architectural characteristics, yet these confounding factors were not systematically addressed. Regarding OED diagnosis and intervention, immunohistochemical staining is occasionally required to assess the risk of malignant transformation (27). However, such analyses were not performed in this investigation. Therefore, future research should evaluate the efficacy of this technology for interpreting immunohistochemically stained specimens.

Moreover, different pathologists noted certain discrepancies in the diagnoses of the same tissue sections. This aligns with the findings of Allard et al. (28), namely that despite clear standards for identifying pathological features, subjective variances among diagnosing pathologists are inevitable due to OED. Although it specifies the boundary involving epithelial layers, the exact indicators for pathological features remain vague, leading to a grading system that assesses the overall morphology of several attributes (29, 30). Owing to the challenges in diagnosing this disease, particularly in difficult cases, consultation with a more experienced physician may be required to confirm the diagnosis. In such instances, the utility of DS30R slice scanning data becomes evident. These histopathological data can be quickly shared among various physicians, enhancing collaboration, and facilitating educational sessions. With the advancements in computer science and technology, an increasing number of researchers are creating more objective systems for evaluating pathological sections using AI (31–33). Gupta et al. (34) used a deep learning algorithm to categorize the severity of epithelial dysplasia in 52 histopathological sections of potentially malignant oral conditions, achieving an accuracy of 89.3%. Employing AI to develop a computer-aided diagnostic system offers the benefits of high precision, time and labor savings, and comprehensive analyses (35, 36). Compared to traditional scanners, the DS30R scanning system, which is enhanced with super-resolution imaging technology, shows significant potential for AI-assisted diagnosis owing to its faster imaging capabilities and lower storage and operational requirements. In future studies, we plan to further assess the effectiveness of the AI-assisted diagnosis of oral diseases.

## Data Availability

The raw data supporting the conclusions of this article will be made available by the authors, without undue reservation.
