# Supplementary material for: Feasibility study of single-image super-resolution scanning system based on deep learning for pathological diagnosis of oral epithelial dysplasia
Source: Front Med (Lausanne). 2025 Mar 12;12:1550512. doi: 10.3389/fmed.2025.1550512 (PMC11936936; doi:10.3389/fmed.2025.1550512)
Supplement: Supplementary file 2 [file Table_2.docx]

Supplementary Material

# Training Dataset

**Training Set for Phase 1**: 176,939 low-resolution and high-resolution images, utilized for super-resolution deep learning training. The image dimensions are 256 x 256 pixels. The low-resolution images were captured using the DS30R array objective lens, while the high-resolution images were captured using the Nikon 20x NA0.4 objective lens.

**Testing Set for Phase 1**: 894 low-resolution and high-resolution images, used for process quality testing during the super-resolution deep learning training process. Process quality testing is conducted every 5 training iterations. The testing outputs PSNR (Peak Signal-to-Noise Ratio) and FID (Fréchet Inception Distance) metrics, which guide the optimization of the deep learning model. Model weights are saved based on the PSNR and FID data and their differences. The image dimensions are 256 x 256 pixels. The low-resolution images were captured using the DS30R array objective lens, while the high-resolution images were captured using the Nikon 20x NA0.4 objective lens.

**Validation Set for Phase 1**: 894 low-resolution and high-resolution images, used for validating the training results during the super-resolution deep learning process. Validation is performed every 10 training iterations. The validation outputs super-resolution images, which are subjectively assessed for quality to monitor the training performance. However, PSNR and FID metrics are not outputted, nor do they guide the optimization of the deep learning model. Model weights are not saved. The image dimensions are 256 x 256 pixels. The low-resolution images were captured using the DS30R array objective lens, while the high-resolution images were captured using the Nikon 20x NA0.4 objective lens.

The image file paths for the training, testing, and validation sets of Phase 1 are located in the CSV file under the "Training Phase1 data list" folder.

**Training Set for Phase 2**: 176,939 low-resolution and high-resolution images, used for super-resolution deep learning training. The image dimensions are 256 x 256 pixels. The low-resolution images were captured using the DS30R array objective lens, while the high-resolution images were captured using the Nikon 20x NA0.8 objective lens.

**Testing Set for Phase 2**: 894 low-resolution and high-resolution images, used for process quality testing during the super-resolution deep learning training process. Process quality testing is conducted every 5 training iterations. The testing outputs PSNR (Peak Signal-to-Noise Ratio) and FID (Fréchet Inception Distance) metrics, which guide the optimization of the deep learning model. Model weights are saved based on the PSNR and FID data and their differences. The image dimensions are 256 x 256 pixels. The low-resolution images were captured using the DS30R array objective lens, while the high-resolution images were captured using the Nikon 20x NA0.8 objective lens.

**Validation Set for Phase 2**: 894 low-resolution and high-resolution images, used for validating the training results during the super-resolution deep learning process. Validation is performed every 10 training iterations. The validation outputs super-resolution images, which are subjectively assessed for quality to monitor the training performance. However, PSNR (Peak Signal-to-Noise Ratio) and FID (Fréchet Inception Distance) metrics are not outputted, nor do they guide the optimization of the deep learning model. Model weights are not saved. The image dimensions are 256 x 256 pixels. The low-resolution images were captured using the DS30R array objective lens, while the high-resolution images were captured using the Nikon 20x NA0.8 objective lens.

The image file paths for the training, testing, and validation sets of Phase 2 are located in the CSV file under the "Training Phase2 data list" folder.

# Training process data

**logs folder**: This folder contains the logs of the training process. The logs are recorded in TensorBoard format and can be opened using TensorBoard. They document the FID (Fréchet Inception Distance), PSNR (Peak Signal-to-Noise Ratio), the differences between FID and PSNR, and the model loss data generated during the training of the super-resolution deep learning model. Specifically, **03.00.01_phase-1_GL0.0** indicates the training data for Phase 1 when the GAN Loss parameter is set to 0.0, while **03.00.01_phase-2_GL0.9** indicates the training data for Phase 2 when the GAN Loss parameter is set to 0.9.

**Evaluation dataset folder**: This folder contains the super-resolution validation images outputted from the best training results. Specifically, **LR** indicates the original images captured by the DS30R, which are used as model inputs; **HR** indicates the images captured by the Nikon 20x NA0.8 objective lens, which are used for comparison; **SR** indicates the images after super-resolution processing.

# Visualization files for training process data

The file **train curve fid&psnr.xlsx** contains the FID (Fréchet Inception Distance) and PSNR (Peak Signal-to-Noise Ratio) data, as well as their difference values, which are outputted during the training process. These data are used for the visualization of charts in this thesis.

# Kappa consistency test for Nikon & DS30R & optical microscope

Fleiss' Kappa for m Raters

Subjects = 200

Raters = 3

Kappa = 0.979

z = 49.5

p-value = 0

Subjects = 200: indicates that the data you are analyzing contains 200 evaluated items or cases.

Raters = 3: indicates that 3 raters participated in the evaluation.

Kappa = 0.979: This is the Fleiss 'Kappa value, which measures agreement between reviewers. Kappa values range from -1 (completely inconsistent) to 1 (completely consistent). A value close to 1 indicates a high degree of agreement among the evaluators. In this case, 0.979 is a very high value, indicating very good agreement between the three raters.

z = 49.5: This is a standardized statistic for the Kappa value and is used to test whether the Kappa value is significantly different from 0 (i.e. there is no agreement between the evaluators).

p-value = 0: This is the p-value associated with the z statistic and is used to determine the statistical significance of the Kappa value. A P-value of 0 indicates that the difference between Kappa values and 0 is extremely significant, and there is almost certainly a high degree of agreement among the evaluators.

**Table S1 Recognition of architectural features**

**Table S2 Recognition of cytological features**

**Table S3 Diagnostic agreement rate of each category**

|  | Amount | Agreement rate |
| --- | --- | --- |
| Normal epithelium | 15/16 | 93.80% |
| Simple hyperplasia | 25/27 | 92.60% |
| Mild dysplasia | 53/59 | 89.80% |
| Moderate dysplasia | 31/34 | 91.20% |
| Severe dysplasia | 42/45 | 93.30% |
| Squamous cell carcinoma | 19/19 | 100.00% |

**Table S4 Results of the Kappa test**

|  | Kappa Value | Asymptotic Standard Error ^a^ | Approximate T ^b^ | Asymptotic Significance |
| --- | --- | --- | --- | --- |
| Nikon-optical microscope | 0.981 | 0.011 | 28.656 | 0.000 |
| DS30R-optical microscope | 0.969 | 0.014 | 28.303 | 0.000 |
| Nikon-DS30R | 0.988 | 0.009 | 28.835 | 0.000 |
| a. Not assuming the null hypothesis. | | | | |
| b. Using asymptotic standard error assuming the null hypothesis. | | | | |
