# Supplementary figures and images for: Feasibility study of single-image super-resolution scanning system based on deep learning for pathological diagnosis of oral epithelial dysplasia (part 2 of 21)
Source: Front Med (Lausanne). 2025 Mar 12;12:1550512. doi: 10.3389/fmed.2025.1550512 (PMC11936936; doi:10.3389/fmed.2025.1550512)

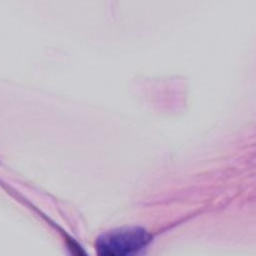

Supplement: Supplementary file 6 [file Data_Sheet_4.zip › HR-01/20_4.tiff]

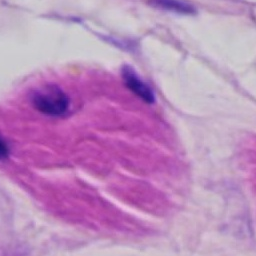

Supplement: Supplementary file 6 [file Data_Sheet_4.zip › HR-01/20_5.tiff]

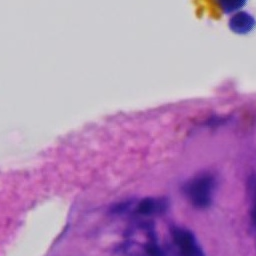

Supplement: Supplementary file 6 [file Data_Sheet_4.zip › HR-01/20_6.tiff]

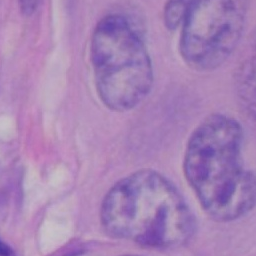

Supplement: Supplementary file 6 [file Data_Sheet_4.zip › HR-01/20_7.tiff]

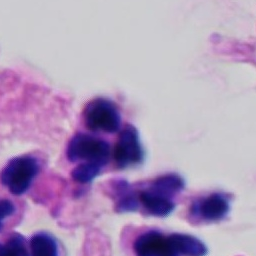

Supplement: Supplementary file 6 [file Data_Sheet_4.zip › HR-01/2_0.tiff]

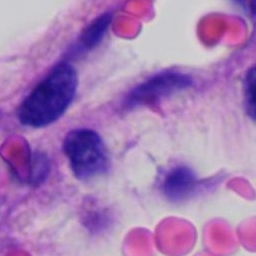

Supplement: Supplementary file 6 [file Data_Sheet_4.zip › HR-01/2_1.tiff]

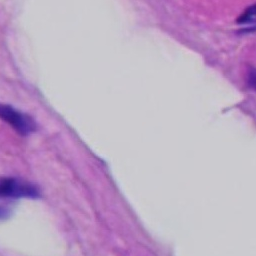

Supplement: Supplementary file 6 [file Data_Sheet_4.zip › HR-01/2_2.tiff]

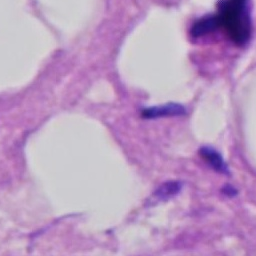

Supplement: Supplementary file 6 [file Data_Sheet_4.zip › HR-01/2_3.tiff]

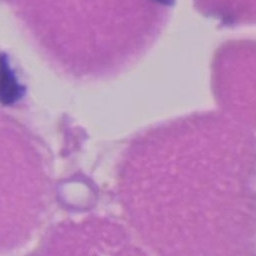

Supplement: Supplementary file 6 [file Data_Sheet_4.zip › HR-01/2_4.tiff]

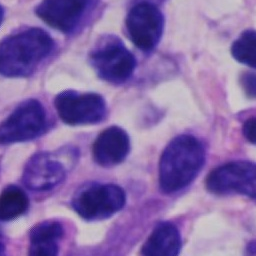

Supplement: Supplementary file 6 [file Data_Sheet_4.zip › HR-01/2_5.tiff]

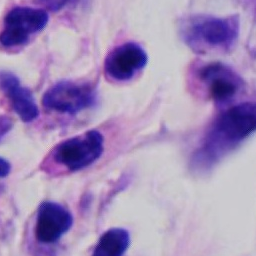

Supplement: Supplementary file 6 [file Data_Sheet_4.zip › HR-01/2_6.tiff]

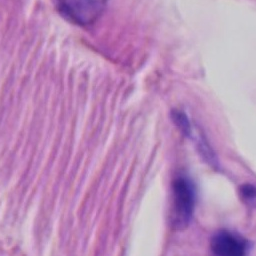

Supplement: Supplementary file 6 [file Data_Sheet_4.zip › HR-01/2_7.tiff]

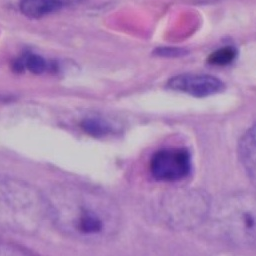

Supplement: Supplementary file 6 [file Data_Sheet_4.zip › HR-01/3_0.tiff]

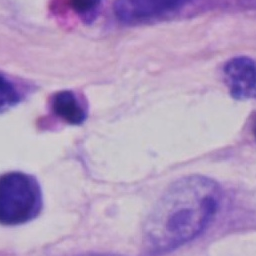

Supplement: Supplementary file 6 [file Data_Sheet_4.zip › HR-01/3_1.tiff]

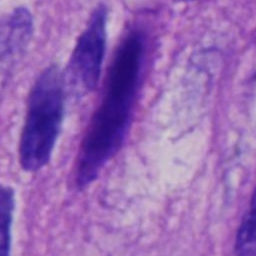

Supplement: Supplementary file 6 [file Data_Sheet_4.zip › HR-01/3_2.tiff]

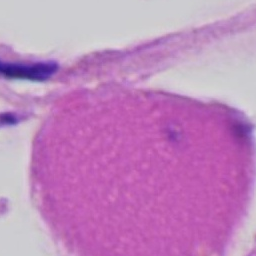

Supplement: Supplementary file 6 [file Data_Sheet_4.zip › HR-01/3_3.tiff]

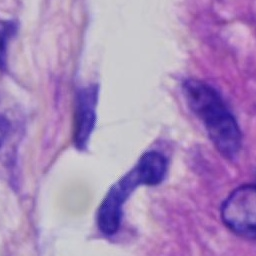

Supplement: Supplementary file 6 [file Data_Sheet_4.zip › HR-01/3_4.tiff]

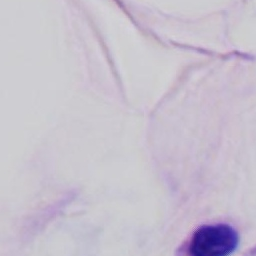

Supplement: Supplementary file 6 [file Data_Sheet_4.zip › HR-01/3_5.tiff]

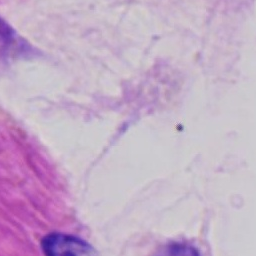

Supplement: Supplementary file 6 [file Data_Sheet_4.zip › HR-01/3_6.tiff]

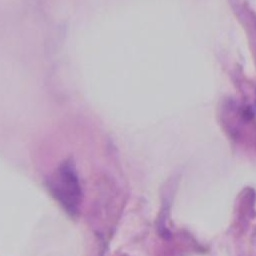

Supplement: Supplementary file 6 [file Data_Sheet_4.zip › HR-01/3_7.tiff]

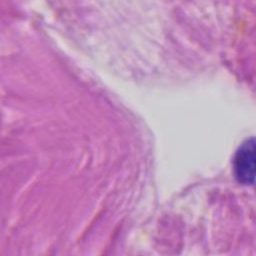

Supplement: Supplementary file 6 [file Data_Sheet_4.zip › HR-01/4_0.tiff]

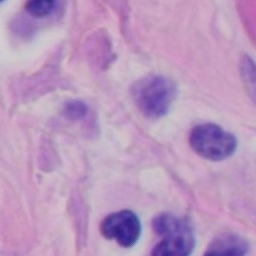

Supplement: Supplementary file 6 [file Data_Sheet_4.zip › HR-01/4_1.tiff]

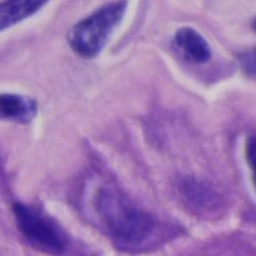

Supplement: Supplementary file 6 [file Data_Sheet_4.zip › HR-01/4_2.tiff]

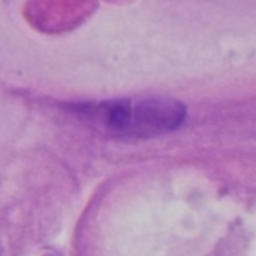

Supplement: Supplementary file 6 [file Data_Sheet_4.zip › HR-01/4_3.tiff]

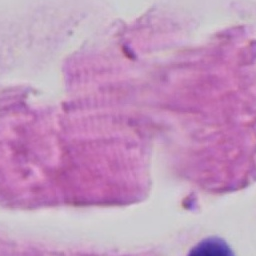

Supplement: Supplementary file 6 [file Data_Sheet_4.zip › HR-01/4_4.tiff]

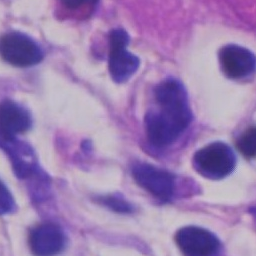

Supplement: Supplementary file 6 [file Data_Sheet_4.zip › HR-01/4_5.tiff]

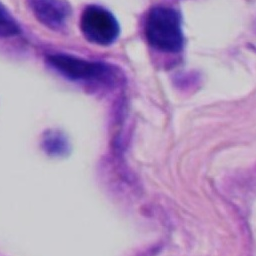

Supplement: Supplementary file 6 [file Data_Sheet_4.zip › HR-01/4_6.tiff]

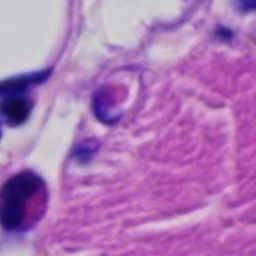

Supplement: Supplementary file 6 [file Data_Sheet_4.zip › HR-01/4_7.tiff]

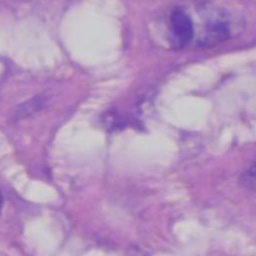

Supplement: Supplementary file 6 [file Data_Sheet_4.zip › HR-01/5_0.tiff]

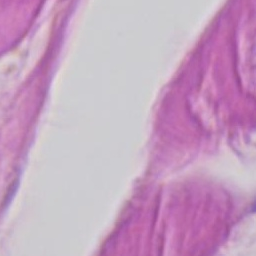

Supplement: Supplementary file 6 [file Data_Sheet_4.zip › HR-01/5_1.tiff]

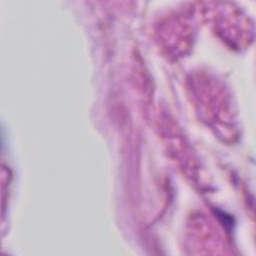

Supplement: Supplementary file 6 [file Data_Sheet_4.zip › HR-01/5_2.tiff]

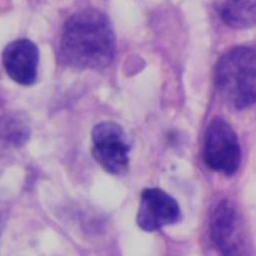

Supplement: Supplementary file 6 [file Data_Sheet_4.zip › HR-01/5_3.tiff]

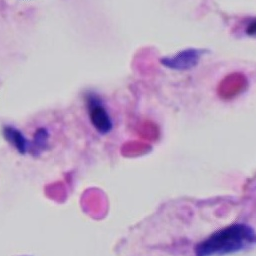

Supplement: Supplementary file 6 [file Data_Sheet_4.zip › HR-01/5_4.tiff]

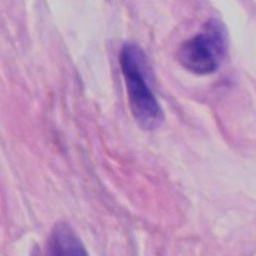

Supplement: Supplementary file 6 [file Data_Sheet_4.zip › HR-01/5_5.tiff]

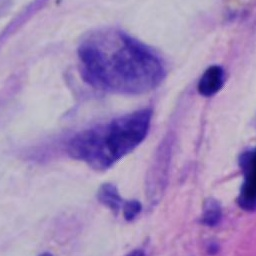

Supplement: Supplementary file 6 [file Data_Sheet_4.zip › HR-01/5_6.tiff]

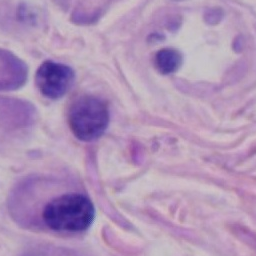

Supplement: Supplementary file 6 [file Data_Sheet_4.zip › HR-01/5_7.tiff]

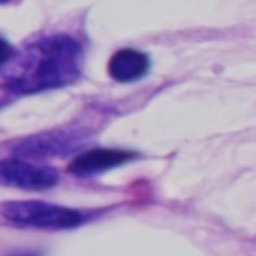

Supplement: Supplementary file 6 [file Data_Sheet_4.zip › HR-01/6_0.tiff]

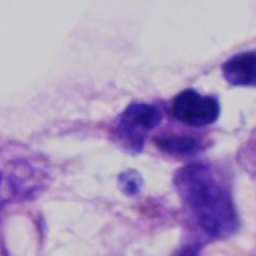

Supplement: Supplementary file 6 [file Data_Sheet_4.zip › HR-01/6_1.tiff]

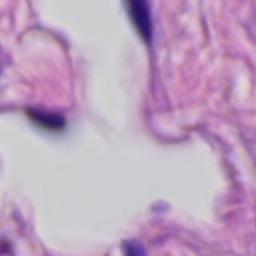

Supplement: Supplementary file 6 [file Data_Sheet_4.zip › HR-01/6_2.tiff]

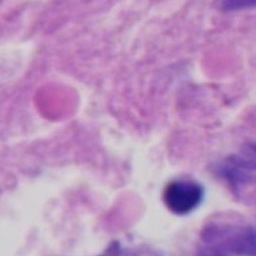

Supplement: Supplementary file 6 [file Data_Sheet_4.zip › HR-01/6_3.tiff]

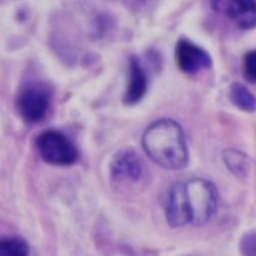

Supplement: Supplementary file 6 [file Data_Sheet_4.zip › HR-01/6_4.tiff]

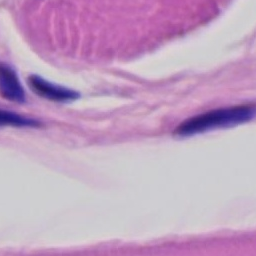

Supplement: Supplementary file 6 [file Data_Sheet_4.zip › HR-01/6_5.tiff]

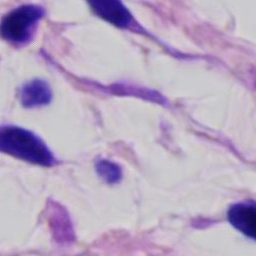

Supplement: Supplementary file 6 [file Data_Sheet_4.zip › HR-01/6_6.tiff]

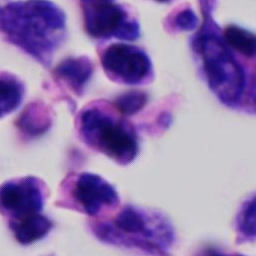

Supplement: Supplementary file 6 [file Data_Sheet_4.zip › HR-01/6_7.tiff]

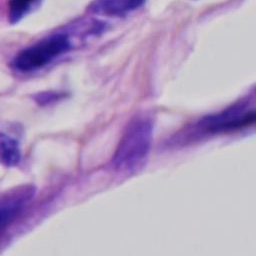

Supplement: Supplementary file 6 [file Data_Sheet_4.zip › HR-01/7_0.tiff]

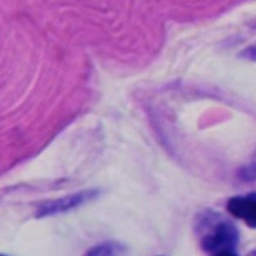

Supplement: Supplementary file 6 [file Data_Sheet_4.zip › HR-01/7_1.tiff]

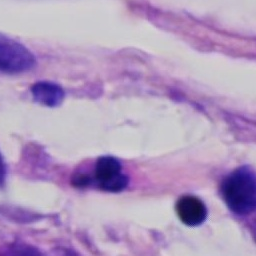

Supplement: Supplementary file 6 [file Data_Sheet_4.zip › HR-01/7_2.tiff]

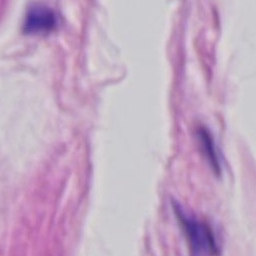

Supplement: Supplementary file 6 [file Data_Sheet_4.zip › HR-01/7_3.tiff]

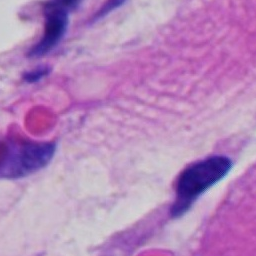

Supplement: Supplementary file 6 [file Data_Sheet_4.zip › HR-01/7_4.tiff]

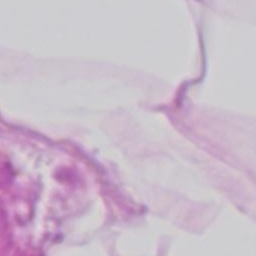

Supplement: Supplementary file 6 [file Data_Sheet_4.zip › HR-01/7_5.tiff]

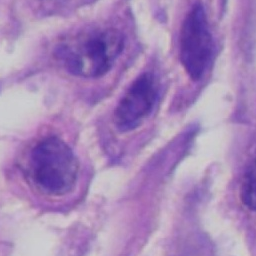

Supplement: Supplementary file 6 [file Data_Sheet_4.zip › HR-01/7_6.tiff]

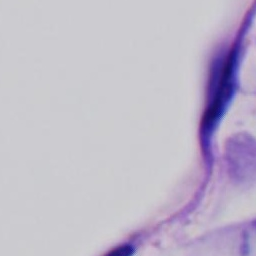

Supplement: Supplementary file 6 [file Data_Sheet_4.zip › HR-01/7_7.tiff]

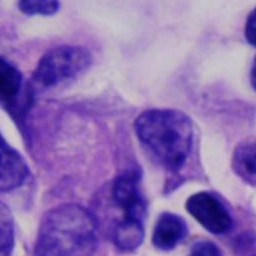

Supplement: Supplementary file 6 [file Data_Sheet_4.zip › HR-01/8_0.tiff]

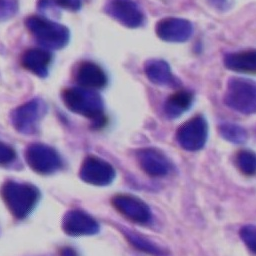

Supplement: Supplementary file 6 [file Data_Sheet_4.zip › HR-01/8_1.tiff]

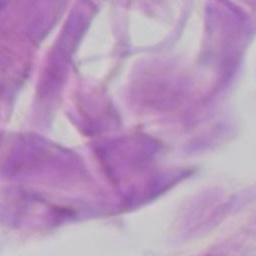

Supplement: Supplementary file 6 [file Data_Sheet_4.zip › HR-01/8_2.tiff]

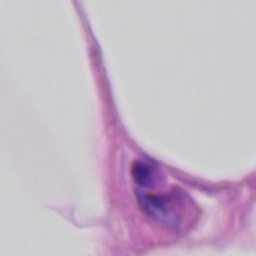

Supplement: Supplementary file 6 [file Data_Sheet_4.zip › HR-01/8_3.tiff]

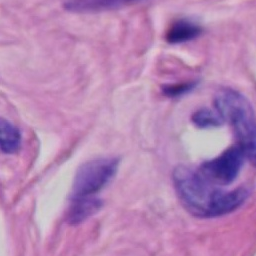

Supplement: Supplementary file 6 [file Data_Sheet_4.zip › HR-01/8_4.tiff]

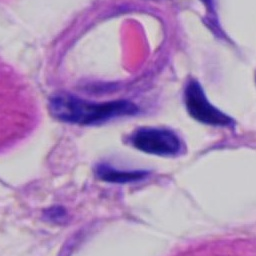

Supplement: Supplementary file 6 [file Data_Sheet_4.zip › HR-01/8_5.tiff]

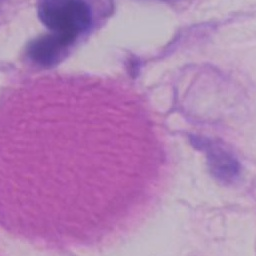

Supplement: Supplementary file 6 [file Data_Sheet_4.zip › HR-01/8_6.tiff]

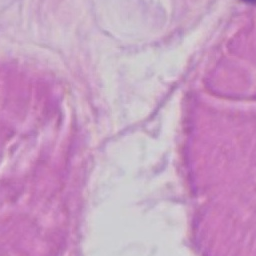

Supplement: Supplementary file 6 [file Data_Sheet_4.zip › HR-01/8_7.tiff]

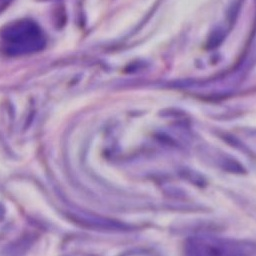

Supplement: Supplementary file 6 [file Data_Sheet_4.zip › HR-01/9_0.tiff]

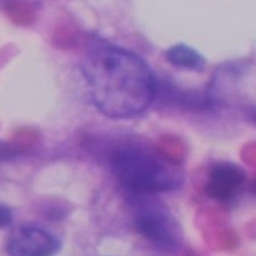

Supplement: Supplementary file 6 [file Data_Sheet_4.zip › HR-01/9_1.tiff]

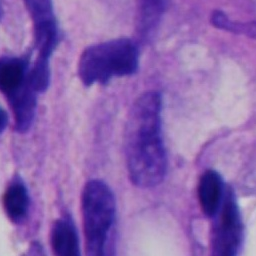

Supplement: Supplementary file 6 [file Data_Sheet_4.zip › HR-01/9_2.tiff]

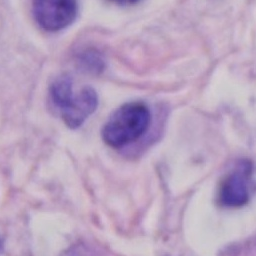

Supplement: Supplementary file 6 [file Data_Sheet_4.zip › HR-01/9_3.tiff]

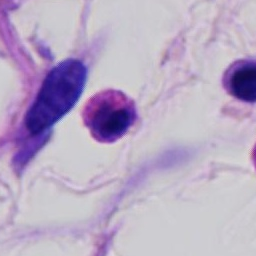

Supplement: Supplementary file 6 [file Data_Sheet_4.zip › HR-01/9_4.tiff]

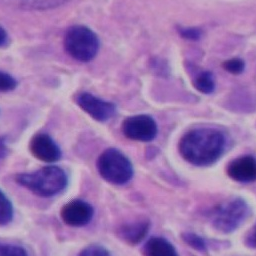

Supplement: Supplementary file 6 [file Data_Sheet_4.zip › HR-01/9_5.tiff]

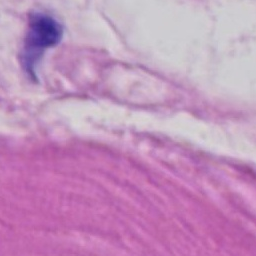

Supplement: Supplementary file 6 [file Data_Sheet_4.zip › HR-01/9_6.tiff]

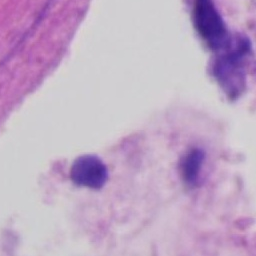

Supplement: Supplementary file 6 [file Data_Sheet_4.zip › HR-01/9_7.tiff]

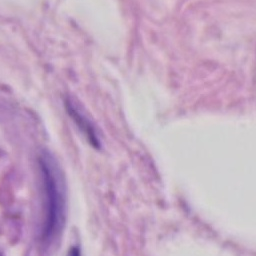

Supplement: Supplementary file 7 [file Data_Sheet_5.zip › HR-02/21_0.tiff]

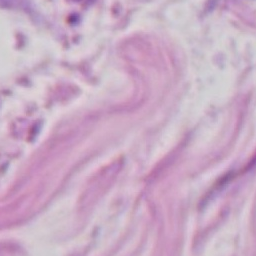

Supplement: Supplementary file 7 [file Data_Sheet_5.zip › HR-02/21_1.tiff]

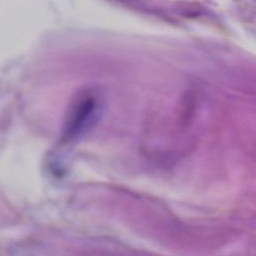

Supplement: Supplementary file 7 [file Data_Sheet_5.zip › HR-02/21_2.tiff]

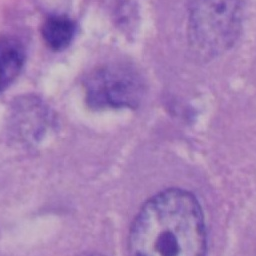

Supplement: Supplementary file 7 [file Data_Sheet_5.zip › HR-02/21_3.tiff]

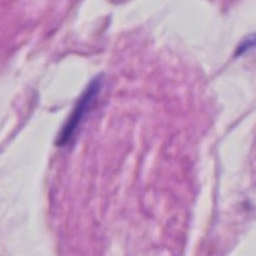

Supplement: Supplementary file 7 [file Data_Sheet_5.zip › HR-02/21_4.tiff]

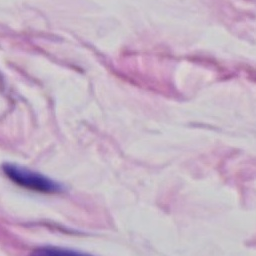

Supplement: Supplementary file 7 [file Data_Sheet_5.zip › HR-02/21_5.tiff]

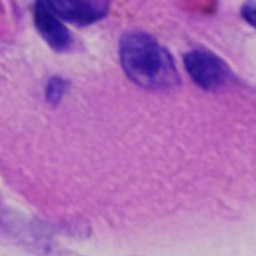

Supplement: Supplementary file 7 [file Data_Sheet_5.zip › HR-02/21_6.tiff]

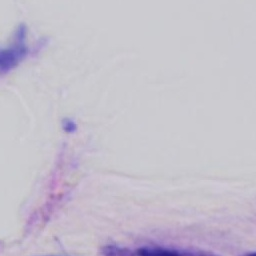

Supplement: Supplementary file 7 [file Data_Sheet_5.zip › HR-02/21_7.tiff]

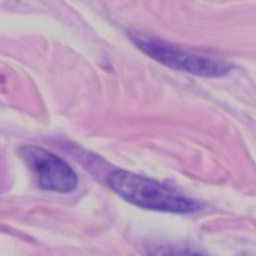

Supplement: Supplementary file 7 [file Data_Sheet_5.zip › HR-02/22_0.tiff]

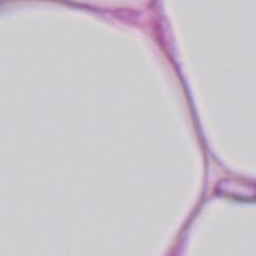

Supplement: Supplementary file 7 [file Data_Sheet_5.zip › HR-02/22_1.tiff]

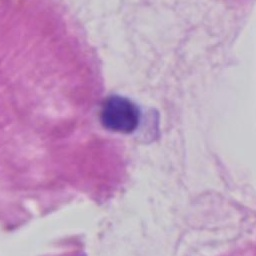

Supplement: Supplementary file 7 [file Data_Sheet_5.zip › HR-02/22_2.tiff]

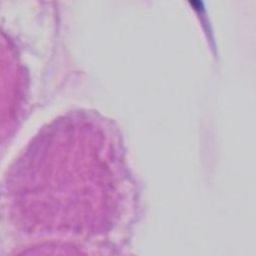

Supplement: Supplementary file 7 [file Data_Sheet_5.zip › HR-02/22_3.tiff]

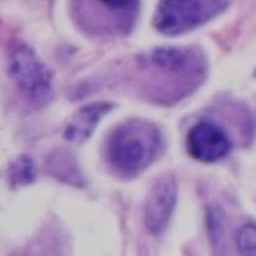

Supplement: Supplementary file 7 [file Data_Sheet_5.zip › HR-02/22_4.tiff]

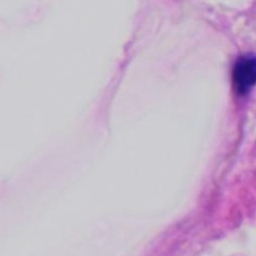

Supplement: Supplementary file 7 [file Data_Sheet_5.zip › HR-02/22_5.tiff]

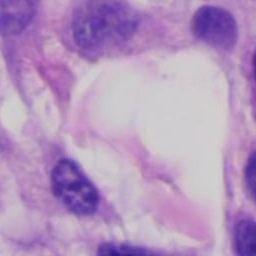

Supplement: Supplementary file 7 [file Data_Sheet_5.zip › HR-02/22_6.tiff]

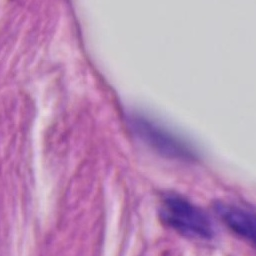

Supplement: Supplementary file 7 [file Data_Sheet_5.zip › HR-02/22_7.tiff]

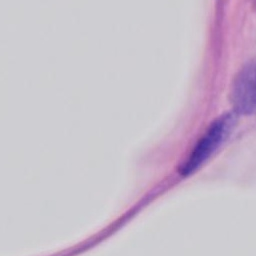

Supplement: Supplementary file 7 [file Data_Sheet_5.zip › HR-02/23_0.tiff]

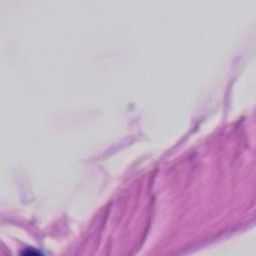

Supplement: Supplementary file 7 [file Data_Sheet_5.zip › HR-02/23_1.tiff]

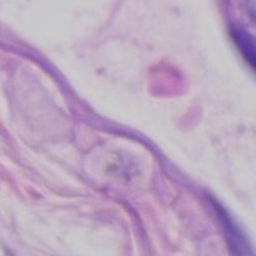

Supplement: Supplementary file 7 [file Data_Sheet_5.zip › HR-02/23_2.tiff]

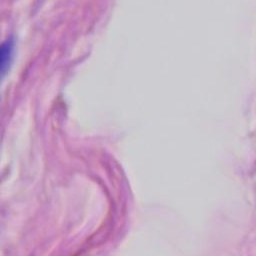

Supplement: Supplementary file 7 [file Data_Sheet_5.zip › HR-02/23_3.tiff]

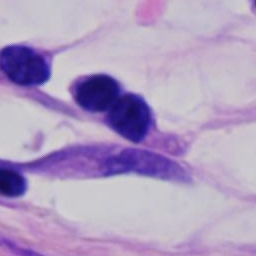

Supplement: Supplementary file 7 [file Data_Sheet_5.zip › HR-02/23_4.tiff]

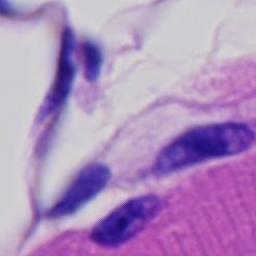

Supplement: Supplementary file 7 [file Data_Sheet_5.zip › HR-02/23_5.tiff]

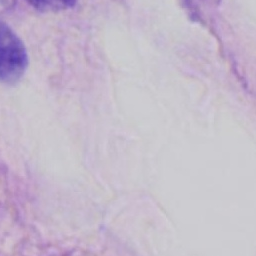

Supplement: Supplementary file 7 [file Data_Sheet_5.zip › HR-02/23_6.tiff]

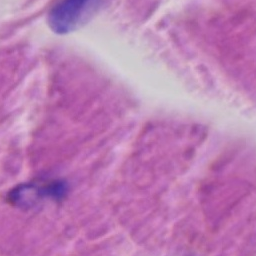

Supplement: Supplementary file 7 [file Data_Sheet_5.zip › HR-02/23_7.tiff]

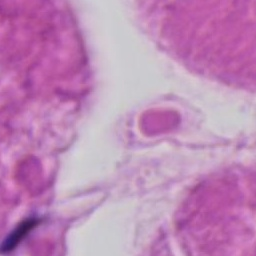

Supplement: Supplementary file 7 [file Data_Sheet_5.zip › HR-02/24_0.tiff]

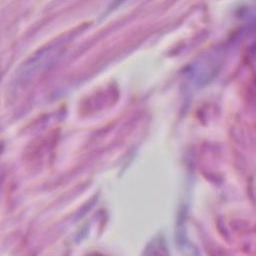

Supplement: Supplementary file 7 [file Data_Sheet_5.zip › HR-02/24_1.tiff]

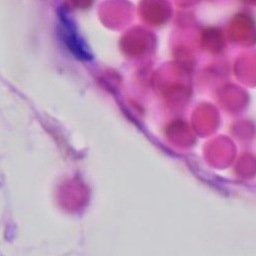

Supplement: Supplementary file 7 [file Data_Sheet_5.zip › HR-02/24_2.tiff]

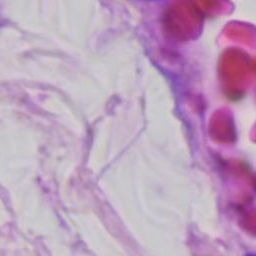

Supplement: Supplementary file 7 [file Data_Sheet_5.zip › HR-02/24_3.tiff]

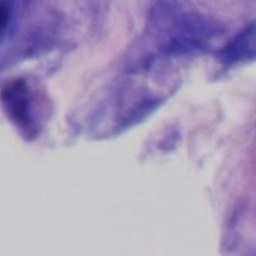

Supplement: Supplementary file 7 [file Data_Sheet_5.zip › HR-02/24_4.tiff]

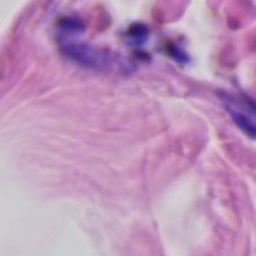

Supplement: Supplementary file 7 [file Data_Sheet_5.zip › HR-02/24_5.tiff]

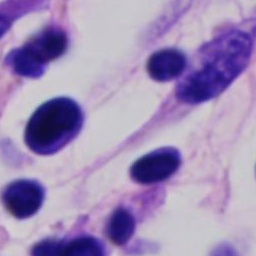

Supplement: Supplementary file 7 [file Data_Sheet_5.zip › HR-02/24_6.tiff]

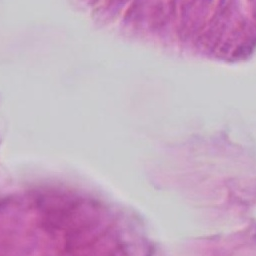

Supplement: Supplementary file 7 [file Data_Sheet_5.zip › HR-02/24_7.tiff]
