# Supplementary figures and images for: Feasibility study of single-image super-resolution scanning system based on deep learning for pathological diagnosis of oral epithelial dysplasia (part 1 of 21)
Source: Front Med (Lausanne). 2025 Mar 12;12:1550512. doi: 10.3389/fmed.2025.1550512 (PMC11936936; doi:10.3389/fmed.2025.1550512)

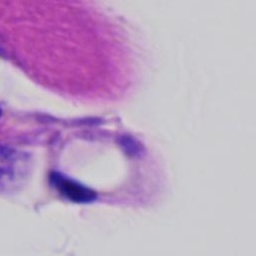

Supplement: Supplementary file 6 [file Data_Sheet_4.zip › HR-01/0_0.tiff]

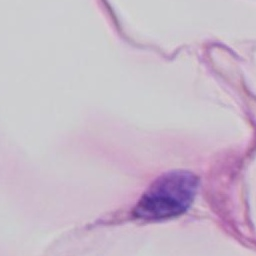

Supplement: Supplementary file 6 [file Data_Sheet_4.zip › HR-01/0_1.tiff]

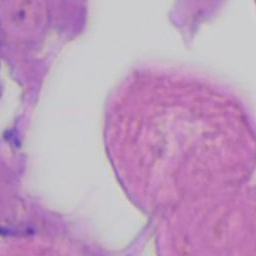

Supplement: Supplementary file 6 [file Data_Sheet_4.zip › HR-01/0_2.tiff]

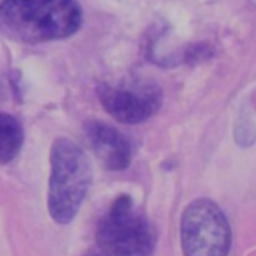

Supplement: Supplementary file 6 [file Data_Sheet_4.zip › HR-01/0_3.tiff]

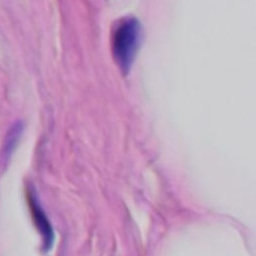

Supplement: Supplementary file 6 [file Data_Sheet_4.zip › HR-01/0_4.tiff]

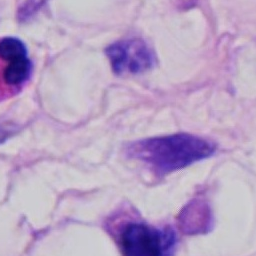

Supplement: Supplementary file 6 [file Data_Sheet_4.zip › HR-01/0_5.tiff]

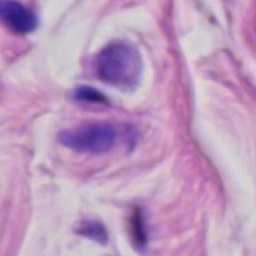

Supplement: Supplementary file 6 [file Data_Sheet_4.zip › HR-01/0_6.tiff]

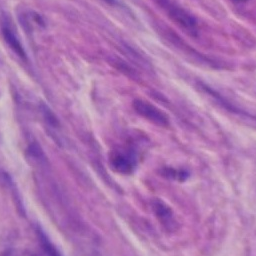

Supplement: Supplementary file 6 [file Data_Sheet_4.zip › HR-01/0_7.tiff]

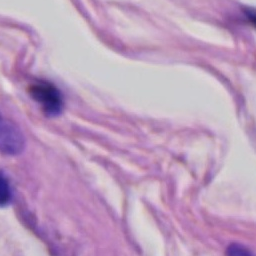

Supplement: Supplementary file 6 [file Data_Sheet_4.zip › HR-01/10_0.tiff]

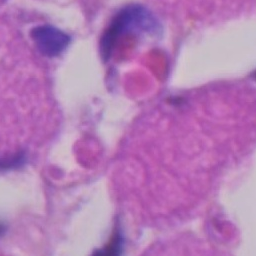

Supplement: Supplementary file 6 [file Data_Sheet_4.zip › HR-01/10_1.tiff]

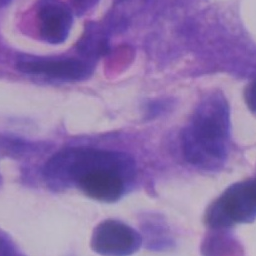

Supplement: Supplementary file 6 [file Data_Sheet_4.zip › HR-01/10_2.tiff]

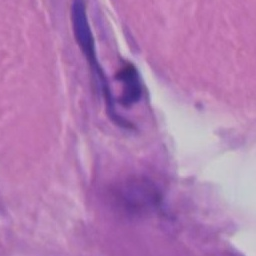

Supplement: Supplementary file 6 [file Data_Sheet_4.zip › HR-01/10_3.tiff]

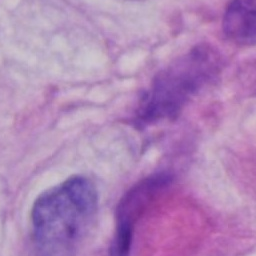

Supplement: Supplementary file 6 [file Data_Sheet_4.zip › HR-01/10_4.tiff]

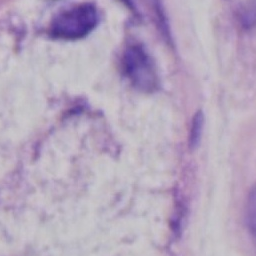

Supplement: Supplementary file 6 [file Data_Sheet_4.zip › HR-01/10_5.tiff]

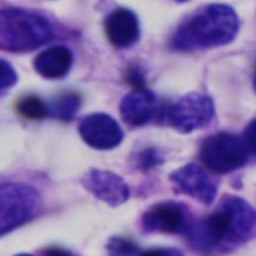

Supplement: Supplementary file 6 [file Data_Sheet_4.zip › HR-01/10_6.tiff]

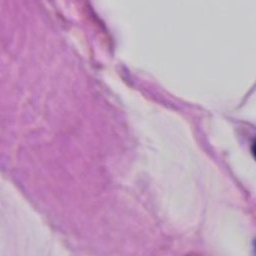

Supplement: Supplementary file 6 [file Data_Sheet_4.zip › HR-01/10_7.tiff]

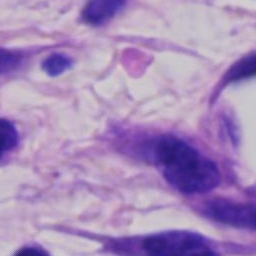

Supplement: Supplementary file 6 [file Data_Sheet_4.zip › HR-01/11_0.tiff]

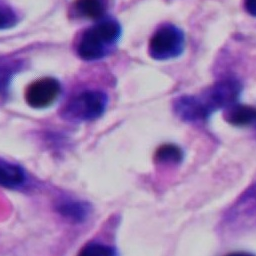

Supplement: Supplementary file 6 [file Data_Sheet_4.zip › HR-01/11_1.tiff]

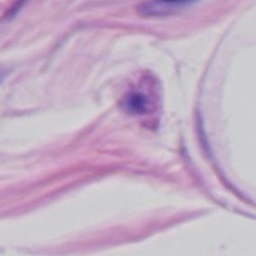

Supplement: Supplementary file 6 [file Data_Sheet_4.zip › HR-01/11_2.tiff]

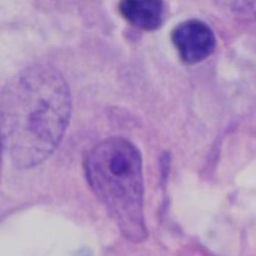

Supplement: Supplementary file 6 [file Data_Sheet_4.zip › HR-01/11_3.tiff]

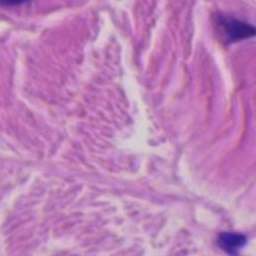

Supplement: Supplementary file 6 [file Data_Sheet_4.zip › HR-01/11_4.tiff]

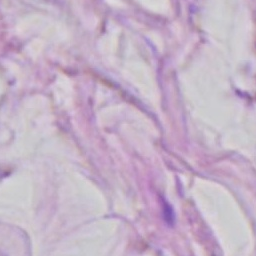

Supplement: Supplementary file 6 [file Data_Sheet_4.zip › HR-01/11_5.tiff]

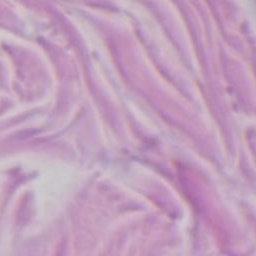

Supplement: Supplementary file 6 [file Data_Sheet_4.zip › HR-01/11_6.tiff]

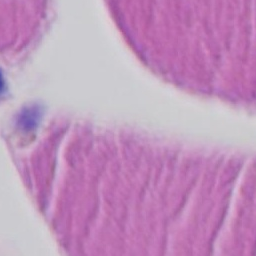

Supplement: Supplementary file 6 [file Data_Sheet_4.zip › HR-01/11_7.tiff]

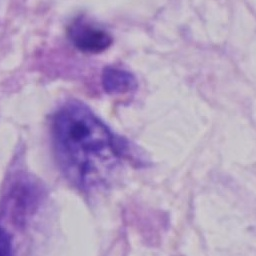

Supplement: Supplementary file 6 [file Data_Sheet_4.zip › HR-01/12_0.tiff]

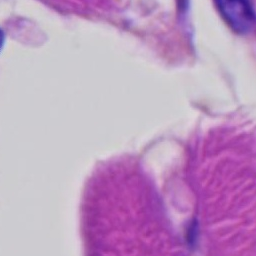

Supplement: Supplementary file 6 [file Data_Sheet_4.zip › HR-01/12_1.tiff]

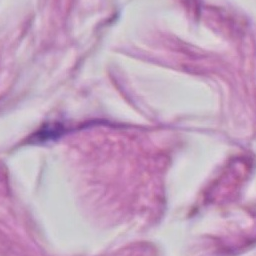

Supplement: Supplementary file 6 [file Data_Sheet_4.zip › HR-01/12_2.tiff]

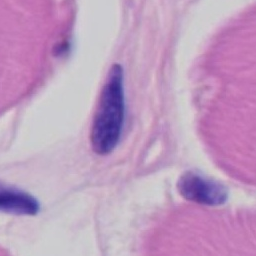

Supplement: Supplementary file 6 [file Data_Sheet_4.zip › HR-01/12_3.tiff]

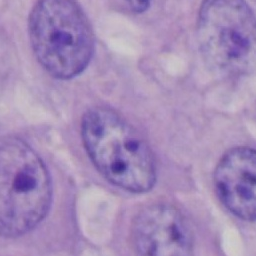

Supplement: Supplementary file 6 [file Data_Sheet_4.zip › HR-01/12_4.tiff]

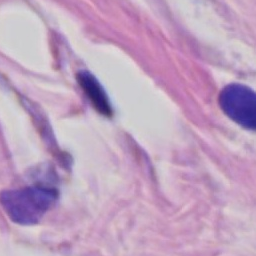

Supplement: Supplementary file 6 [file Data_Sheet_4.zip › HR-01/12_5.tiff]

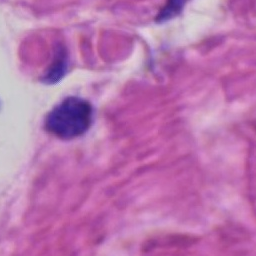

Supplement: Supplementary file 6 [file Data_Sheet_4.zip › HR-01/12_6.tiff]

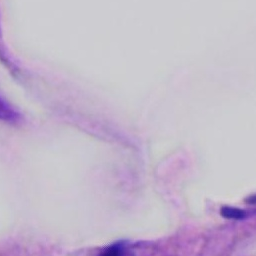

Supplement: Supplementary file 6 [file Data_Sheet_4.zip › HR-01/12_7.tiff]

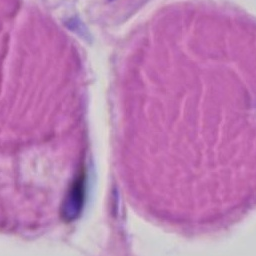

Supplement: Supplementary file 6 [file Data_Sheet_4.zip › HR-01/13_0.tiff]

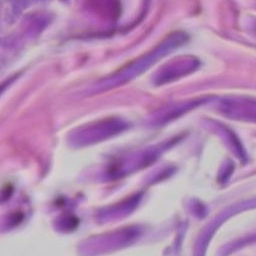

Supplement: Supplementary file 6 [file Data_Sheet_4.zip › HR-01/13_1.tiff]

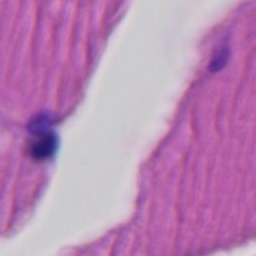

Supplement: Supplementary file 6 [file Data_Sheet_4.zip › HR-01/13_2.tiff]

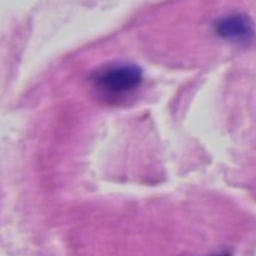

Supplement: Supplementary file 6 [file Data_Sheet_4.zip › HR-01/13_3.tiff]

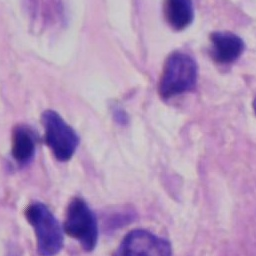

Supplement: Supplementary file 6 [file Data_Sheet_4.zip › HR-01/13_4.tiff]

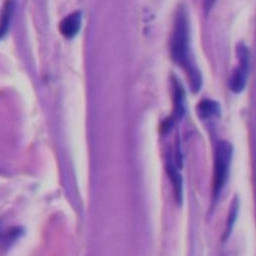

Supplement: Supplementary file 6 [file Data_Sheet_4.zip › HR-01/13_5.tiff]

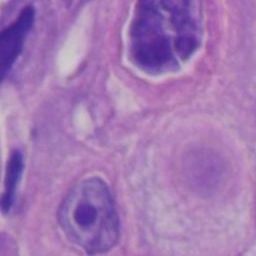

Supplement: Supplementary file 6 [file Data_Sheet_4.zip › HR-01/13_6.tiff]

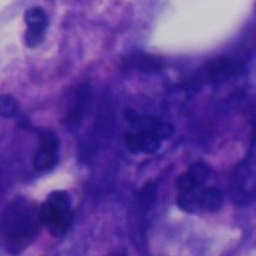

Supplement: Supplementary file 6 [file Data_Sheet_4.zip › HR-01/13_7.tiff]

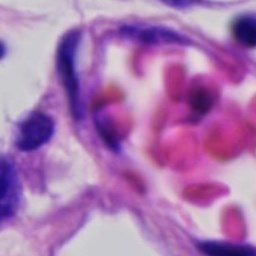

Supplement: Supplementary file 6 [file Data_Sheet_4.zip › HR-01/14_0.tiff]

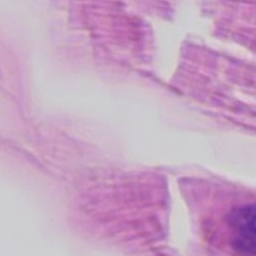

Supplement: Supplementary file 6 [file Data_Sheet_4.zip › HR-01/14_1.tiff]

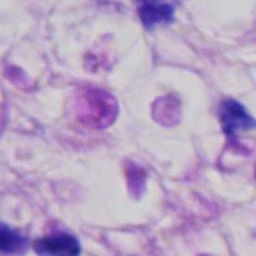

Supplement: Supplementary file 6 [file Data_Sheet_4.zip › HR-01/14_2.tiff]

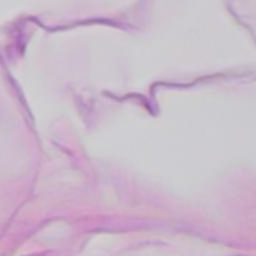

Supplement: Supplementary file 6 [file Data_Sheet_4.zip › HR-01/14_3.tiff]

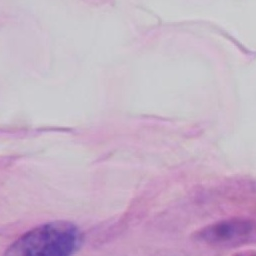

Supplement: Supplementary file 6 [file Data_Sheet_4.zip › HR-01/14_4.tiff]

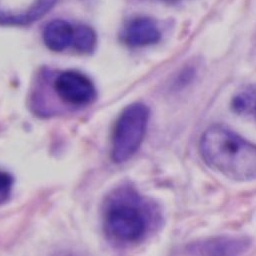

Supplement: Supplementary file 6 [file Data_Sheet_4.zip › HR-01/14_5.tiff]

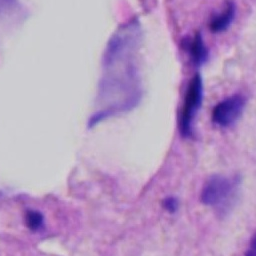

Supplement: Supplementary file 6 [file Data_Sheet_4.zip › HR-01/14_6.tiff]

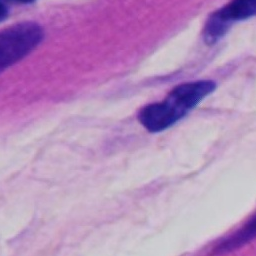

Supplement: Supplementary file 6 [file Data_Sheet_4.zip › HR-01/14_7.tiff]

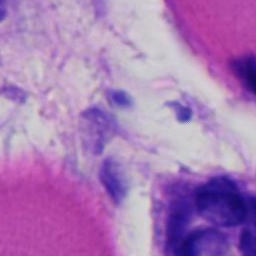

Supplement: Supplementary file 6 [file Data_Sheet_4.zip › HR-01/15_0.tiff]

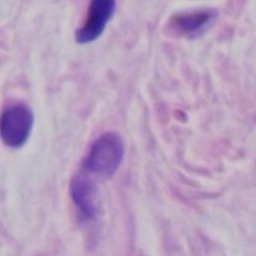

Supplement: Supplementary file 6 [file Data_Sheet_4.zip › HR-01/15_1.tiff]

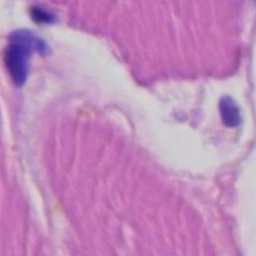

Supplement: Supplementary file 6 [file Data_Sheet_4.zip › HR-01/15_2.tiff]

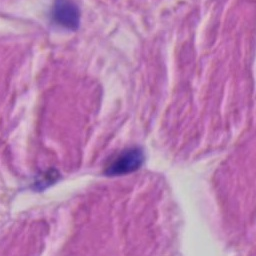

Supplement: Supplementary file 6 [file Data_Sheet_4.zip › HR-01/15_3.tiff]

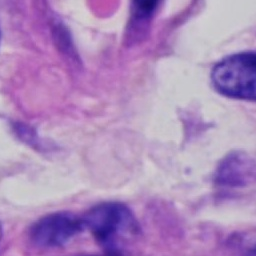

Supplement: Supplementary file 6 [file Data_Sheet_4.zip › HR-01/15_4.tiff]

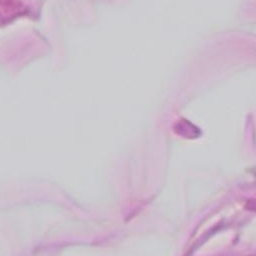

Supplement: Supplementary file 6 [file Data_Sheet_4.zip › HR-01/15_5.tiff]

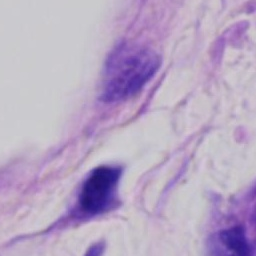

Supplement: Supplementary file 6 [file Data_Sheet_4.zip › HR-01/15_6.tiff]

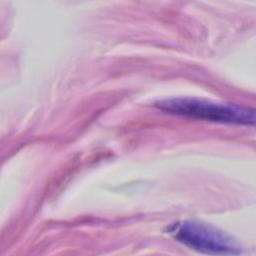

Supplement: Supplementary file 6 [file Data_Sheet_4.zip › HR-01/15_7.tiff]

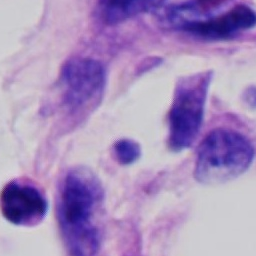

Supplement: Supplementary file 6 [file Data_Sheet_4.zip › HR-01/16_0.tiff]

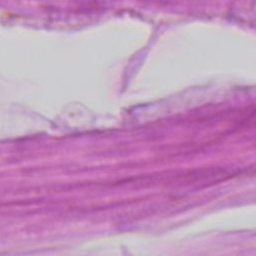

Supplement: Supplementary file 6 [file Data_Sheet_4.zip › HR-01/16_1.tiff]

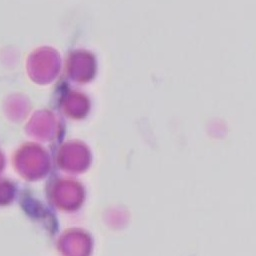

Supplement: Supplementary file 6 [file Data_Sheet_4.zip › HR-01/16_2.tiff]

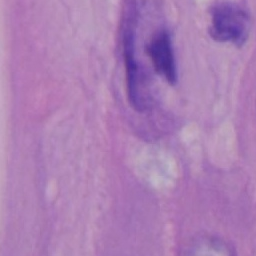

Supplement: Supplementary file 6 [file Data_Sheet_4.zip › HR-01/16_3.tiff]

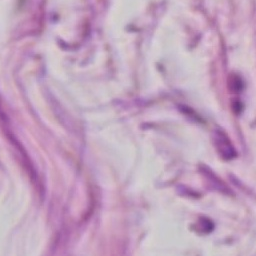

Supplement: Supplementary file 6 [file Data_Sheet_4.zip › HR-01/16_4.tiff]

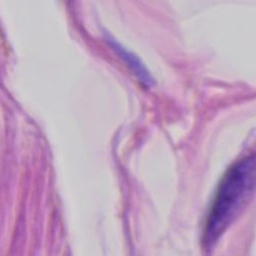

Supplement: Supplementary file 6 [file Data_Sheet_4.zip › HR-01/16_5.tiff]

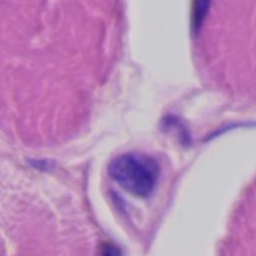

Supplement: Supplementary file 6 [file Data_Sheet_4.zip › HR-01/16_6.tiff]

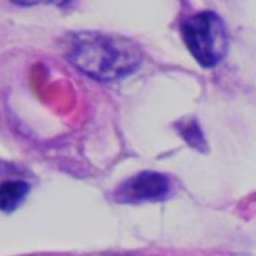

Supplement: Supplementary file 6 [file Data_Sheet_4.zip › HR-01/16_7.tiff]

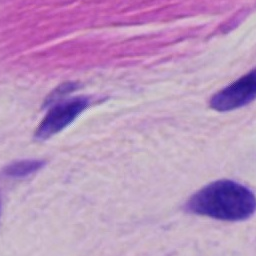

Supplement: Supplementary file 6 [file Data_Sheet_4.zip › HR-01/17_0.tiff]

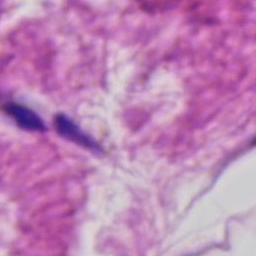

Supplement: Supplementary file 6 [file Data_Sheet_4.zip › HR-01/17_1.tiff]

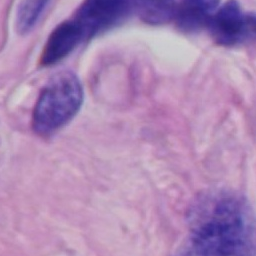

Supplement: Supplementary file 6 [file Data_Sheet_4.zip › HR-01/17_2.tiff]

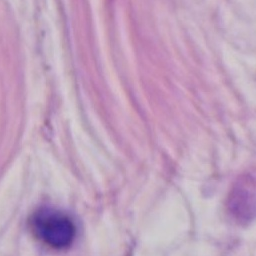

Supplement: Supplementary file 6 [file Data_Sheet_4.zip › HR-01/17_3.tiff]

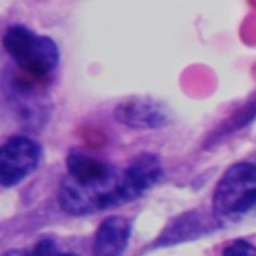

Supplement: Supplementary file 6 [file Data_Sheet_4.zip › HR-01/17_4.tiff]

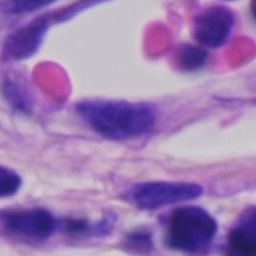

Supplement: Supplementary file 6 [file Data_Sheet_4.zip › HR-01/17_5.tiff]

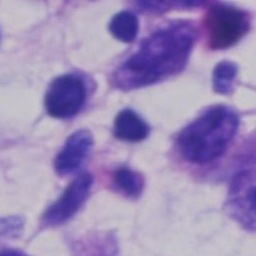

Supplement: Supplementary file 6 [file Data_Sheet_4.zip › HR-01/17_6.tiff]

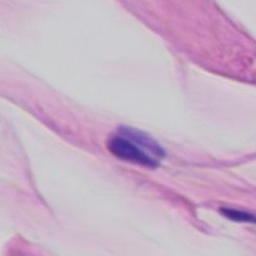

Supplement: Supplementary file 6 [file Data_Sheet_4.zip › HR-01/17_7.tiff]

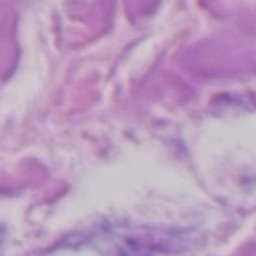

Supplement: Supplementary file 6 [file Data_Sheet_4.zip › HR-01/18_0.tiff]

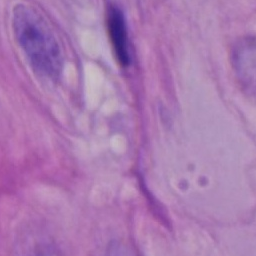

Supplement: Supplementary file 6 [file Data_Sheet_4.zip › HR-01/18_1.tiff]

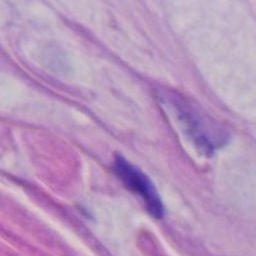

Supplement: Supplementary file 6 [file Data_Sheet_4.zip › HR-01/18_2.tiff]

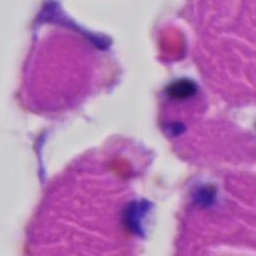

Supplement: Supplementary file 6 [file Data_Sheet_4.zip › HR-01/18_3.tiff]

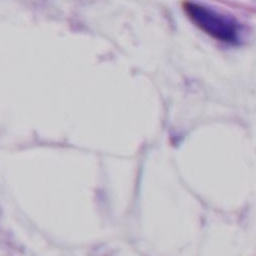

Supplement: Supplementary file 6 [file Data_Sheet_4.zip › HR-01/18_4.tiff]

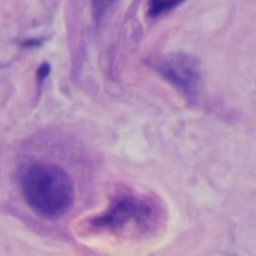

Supplement: Supplementary file 6 [file Data_Sheet_4.zip › HR-01/18_5.tiff]

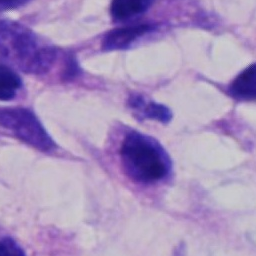

Supplement: Supplementary file 6 [file Data_Sheet_4.zip › HR-01/18_6.tiff]

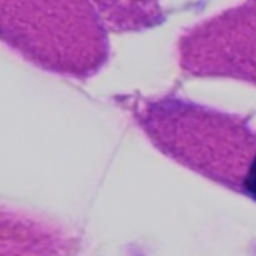

Supplement: Supplementary file 6 [file Data_Sheet_4.zip › HR-01/18_7.tiff]

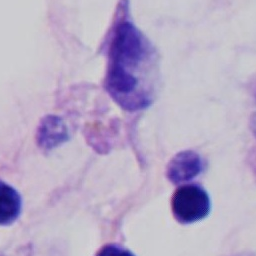

Supplement: Supplementary file 6 [file Data_Sheet_4.zip › HR-01/19_0.tiff]

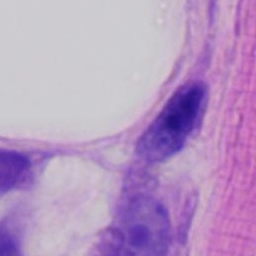

Supplement: Supplementary file 6 [file Data_Sheet_4.zip › HR-01/19_1.tiff]

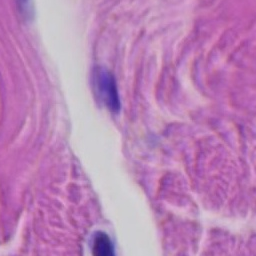

Supplement: Supplementary file 6 [file Data_Sheet_4.zip › HR-01/19_2.tiff]

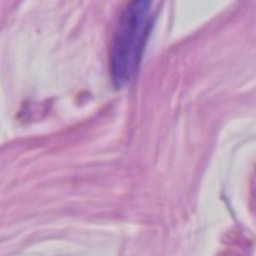

Supplement: Supplementary file 6 [file Data_Sheet_4.zip › HR-01/19_3.tiff]

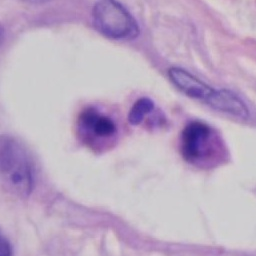

Supplement: Supplementary file 6 [file Data_Sheet_4.zip › HR-01/19_4.tiff]

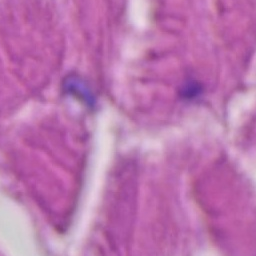

Supplement: Supplementary file 6 [file Data_Sheet_4.zip › HR-01/19_5.tiff]

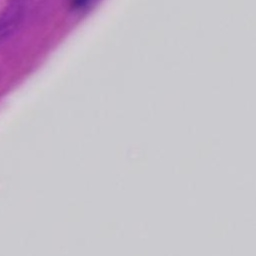

Supplement: Supplementary file 6 [file Data_Sheet_4.zip › HR-01/19_6.tiff]

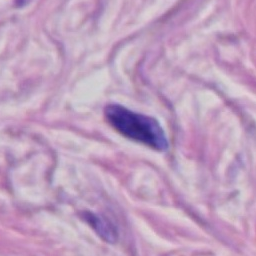

Supplement: Supplementary file 6 [file Data_Sheet_4.zip › HR-01/19_7.tiff]

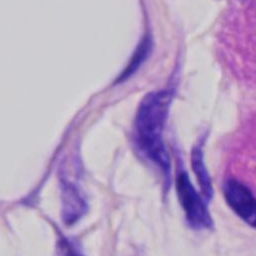

Supplement: Supplementary file 6 [file Data_Sheet_4.zip › HR-01/1_0.tiff]

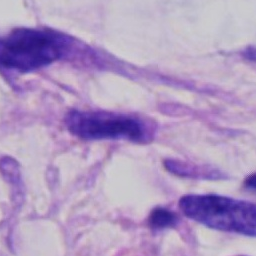

Supplement: Supplementary file 6 [file Data_Sheet_4.zip › HR-01/1_1.tiff]

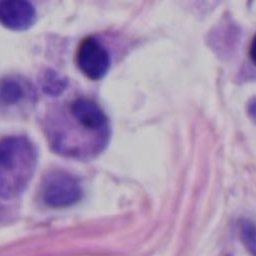

Supplement: Supplementary file 6 [file Data_Sheet_4.zip › HR-01/1_2.tiff]

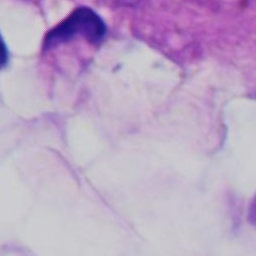

Supplement: Supplementary file 6 [file Data_Sheet_4.zip › HR-01/1_3.tiff]

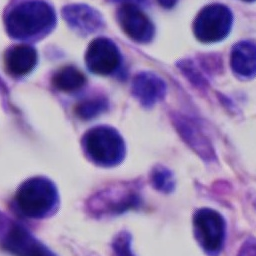

Supplement: Supplementary file 6 [file Data_Sheet_4.zip › HR-01/1_4.tiff]

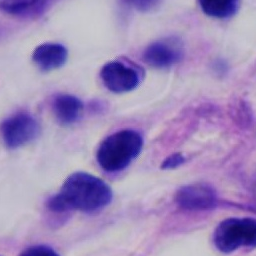

Supplement: Supplementary file 6 [file Data_Sheet_4.zip › HR-01/1_5.tiff]

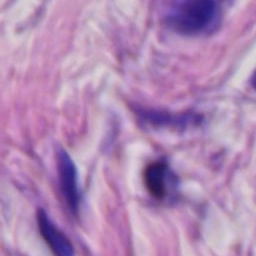

Supplement: Supplementary file 6 [file Data_Sheet_4.zip › HR-01/1_6.tiff]

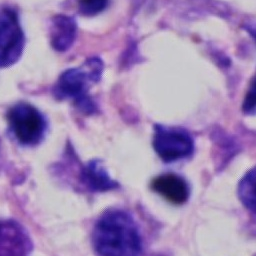

Supplement: Supplementary file 6 [file Data_Sheet_4.zip › HR-01/1_7.tiff]

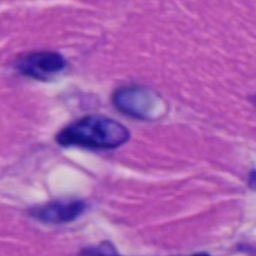

Supplement: Supplementary file 6 [file Data_Sheet_4.zip › HR-01/20_0.tiff]

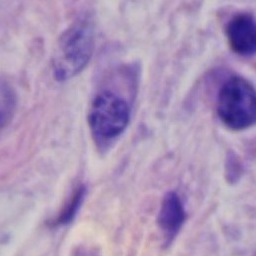

Supplement: Supplementary file 6 [file Data_Sheet_4.zip › HR-01/20_1.tiff]

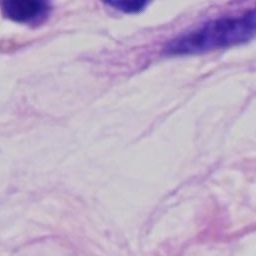

Supplement: Supplementary file 6 [file Data_Sheet_4.zip › HR-01/20_2.tiff]

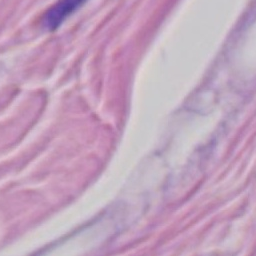

Supplement: Supplementary file 6 [file Data_Sheet_4.zip › HR-01/20_3.tiff]
