# Supplementary figures and images for: Feasibility study of single-image super-resolution scanning system based on deep learning for pathological diagnosis of oral epithelial dysplasia (part 4 of 21)
Source: Front Med (Lausanne). 2025 Mar 12;12:1550512. doi: 10.3389/fmed.2025.1550512 (PMC11936936; doi:10.3389/fmed.2025.1550512)

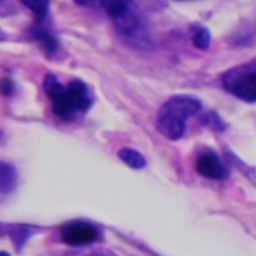

Supplement: Supplementary file 7 [file Data_Sheet_5.zip › HR-02/37_4.tiff]

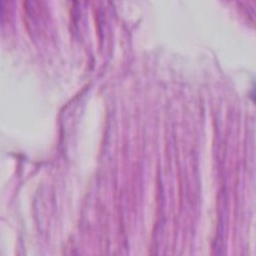

Supplement: Supplementary file 7 [file Data_Sheet_5.zip › HR-02/37_5.tiff]

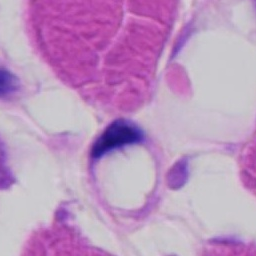

Supplement: Supplementary file 7 [file Data_Sheet_5.zip › HR-02/37_6.tiff]

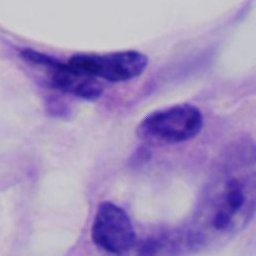

Supplement: Supplementary file 7 [file Data_Sheet_5.zip › HR-02/37_7.tiff]

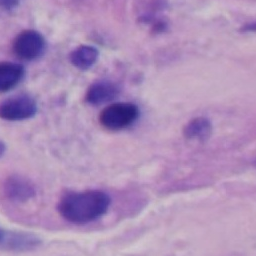

Supplement: Supplementary file 7 [file Data_Sheet_5.zip › HR-02/38_0.tiff]

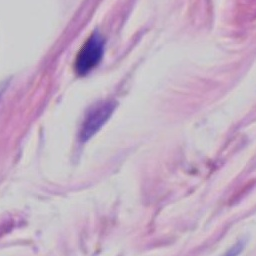

Supplement: Supplementary file 7 [file Data_Sheet_5.zip › HR-02/38_1.tiff]

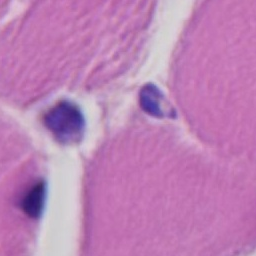

Supplement: Supplementary file 7 [file Data_Sheet_5.zip › HR-02/38_2.tiff]

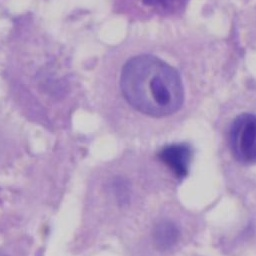

Supplement: Supplementary file 7 [file Data_Sheet_5.zip › HR-02/38_3.tiff]

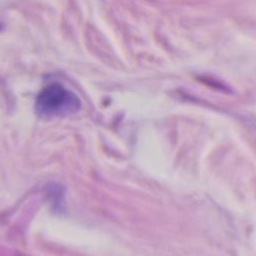

Supplement: Supplementary file 7 [file Data_Sheet_5.zip › HR-02/38_4.tiff]

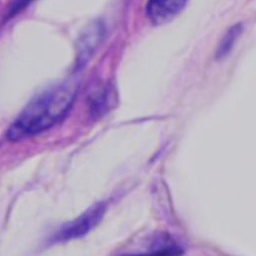

Supplement: Supplementary file 7 [file Data_Sheet_5.zip › HR-02/38_5.tiff]

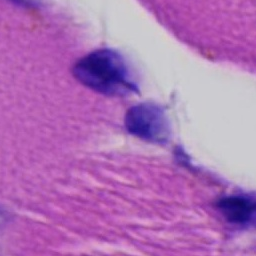

Supplement: Supplementary file 7 [file Data_Sheet_5.zip › HR-02/38_6.tiff]

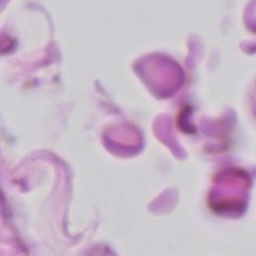

Supplement: Supplementary file 7 [file Data_Sheet_5.zip › HR-02/38_7.tiff]

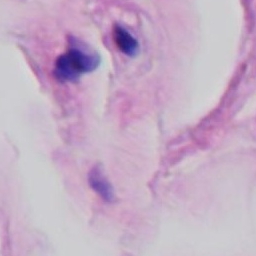

Supplement: Supplementary file 7 [file Data_Sheet_5.zip › HR-02/39_0.tiff]

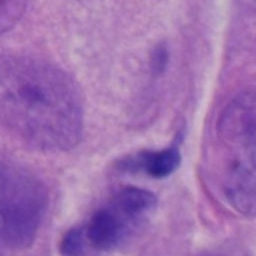

Supplement: Supplementary file 7 [file Data_Sheet_5.zip › HR-02/39_1.tiff]

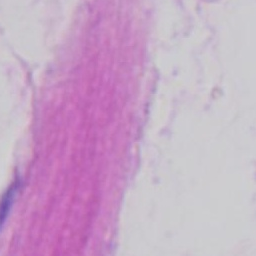

Supplement: Supplementary file 7 [file Data_Sheet_5.zip › HR-02/39_2.tiff]

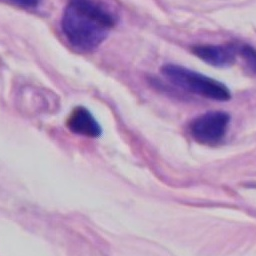

Supplement: Supplementary file 7 [file Data_Sheet_5.zip › HR-02/39_3.tiff]

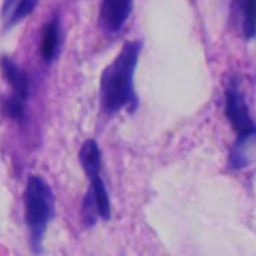

Supplement: Supplementary file 7 [file Data_Sheet_5.zip › HR-02/39_4.tiff]

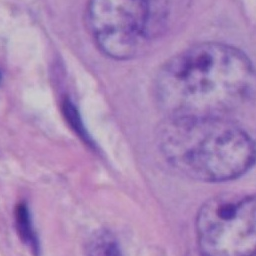

Supplement: Supplementary file 7 [file Data_Sheet_5.zip › HR-02/39_5.tiff]

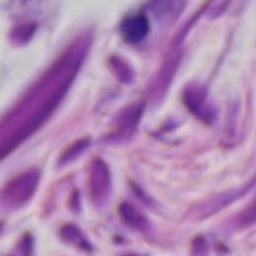

Supplement: Supplementary file 7 [file Data_Sheet_5.zip › HR-02/39_6.tiff]

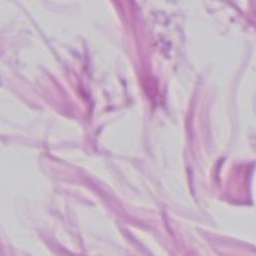

Supplement: Supplementary file 7 [file Data_Sheet_5.zip › HR-02/39_7.tiff]

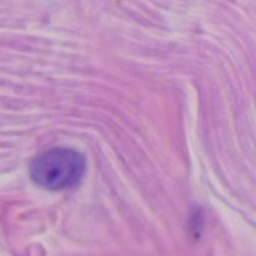

Supplement: Supplementary file 7 [file Data_Sheet_5.zip › HR-02/40_0.tiff]

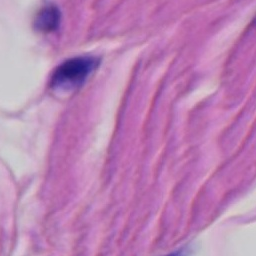

Supplement: Supplementary file 7 [file Data_Sheet_5.zip › HR-02/40_1.tiff]

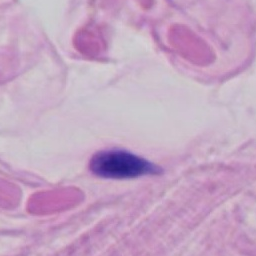

Supplement: Supplementary file 7 [file Data_Sheet_5.zip › HR-02/40_2.tiff]

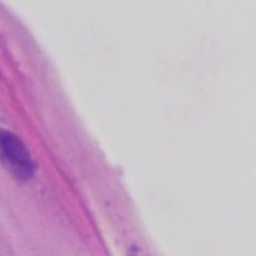

Supplement: Supplementary file 7 [file Data_Sheet_5.zip › HR-02/40_3.tiff]

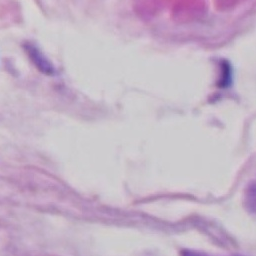

Supplement: Supplementary file 7 [file Data_Sheet_5.zip › HR-02/40_4.tiff]

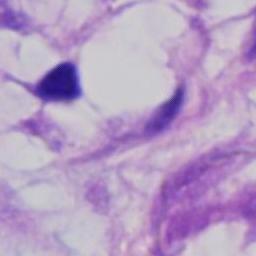

Supplement: Supplementary file 7 [file Data_Sheet_5.zip › HR-02/40_5.tiff]

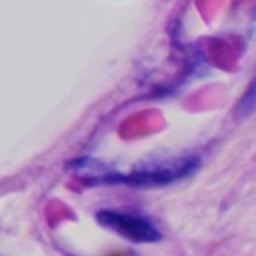

Supplement: Supplementary file 7 [file Data_Sheet_5.zip › HR-02/40_6.tiff]

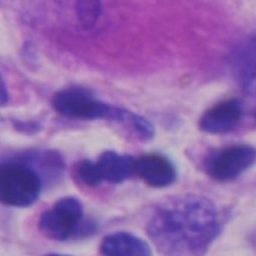

Supplement: Supplementary file 7 [file Data_Sheet_5.zip › HR-02/40_7.tiff]

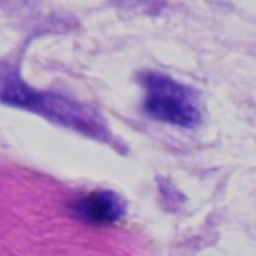

Supplement: Supplementary file 7 [file Data_Sheet_5.zip › HR-02/41_0.tiff]

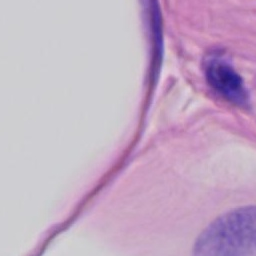

Supplement: Supplementary file 7 [file Data_Sheet_5.zip › HR-02/41_1.tiff]

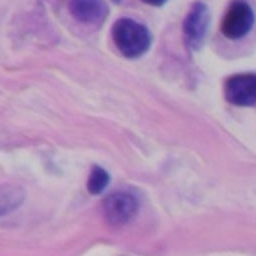

Supplement: Supplementary file 7 [file Data_Sheet_5.zip › HR-02/41_2.tiff]

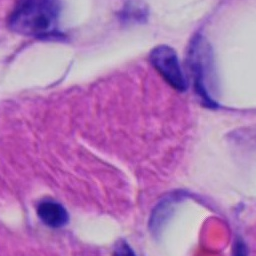

Supplement: Supplementary file 7 [file Data_Sheet_5.zip › HR-02/41_3.tiff]

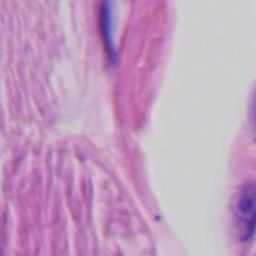

Supplement: Supplementary file 7 [file Data_Sheet_5.zip › HR-02/41_4.tiff]

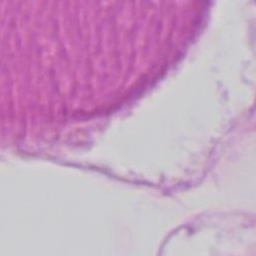

Supplement: Supplementary file 7 [file Data_Sheet_5.zip › HR-02/41_5.tiff]

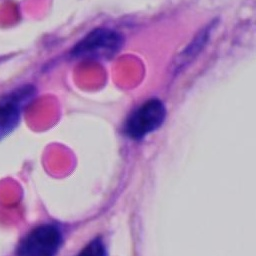

Supplement: Supplementary file 7 [file Data_Sheet_5.zip › HR-02/41_6.tiff]

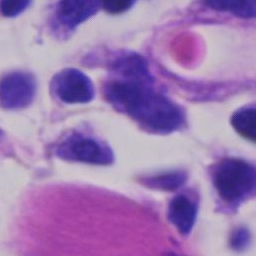

Supplement: Supplementary file 7 [file Data_Sheet_5.zip › HR-02/41_7.tiff]

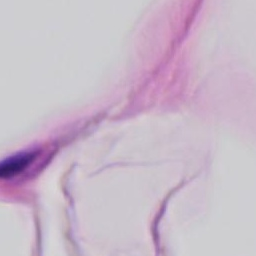

Supplement: Supplementary file 7 [file Data_Sheet_5.zip › HR-02/42_0.tiff]

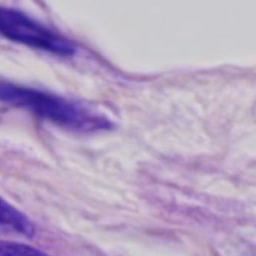

Supplement: Supplementary file 7 [file Data_Sheet_5.zip › HR-02/42_1.tiff]

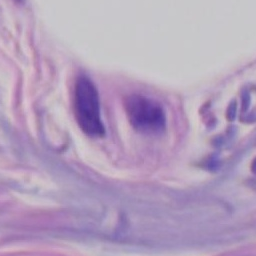

Supplement: Supplementary file 7 [file Data_Sheet_5.zip › HR-02/42_2.tiff]

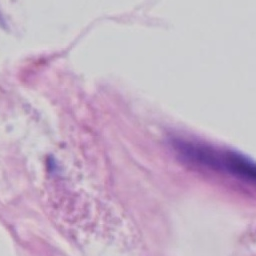

Supplement: Supplementary file 7 [file Data_Sheet_5.zip › HR-02/42_3.tiff]

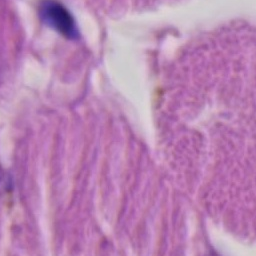

Supplement: Supplementary file 7 [file Data_Sheet_5.zip › HR-02/42_4.tiff]

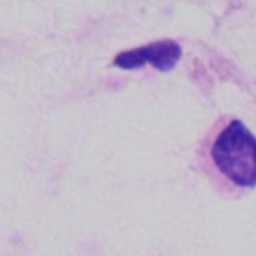

Supplement: Supplementary file 7 [file Data_Sheet_5.zip › HR-02/42_5.tiff]

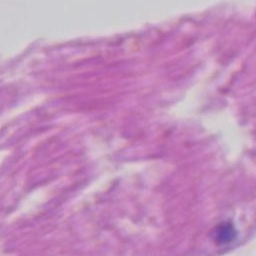

Supplement: Supplementary file 7 [file Data_Sheet_5.zip › HR-02/42_6.tiff]

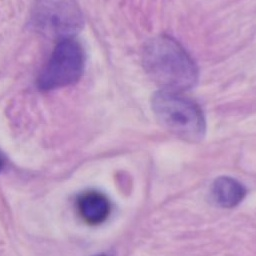

Supplement: Supplementary file 7 [file Data_Sheet_5.zip › HR-02/42_7.tiff]

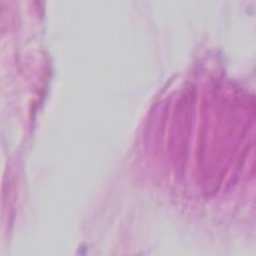

Supplement: Supplementary file 7 [file Data_Sheet_5.zip › HR-02/43_0.tiff]

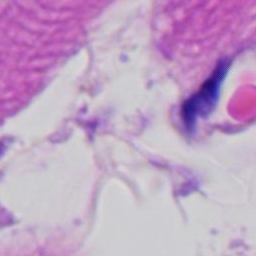

Supplement: Supplementary file 7 [file Data_Sheet_5.zip › HR-02/43_1.tiff]

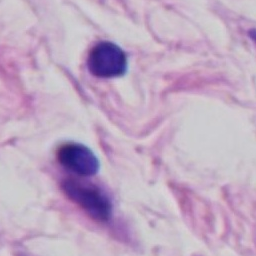

Supplement: Supplementary file 7 [file Data_Sheet_5.zip › HR-02/43_2.tiff]

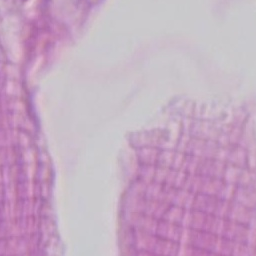

Supplement: Supplementary file 7 [file Data_Sheet_5.zip › HR-02/43_3.tiff]

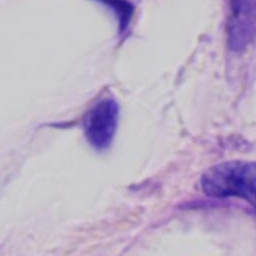

Supplement: Supplementary file 7 [file Data_Sheet_5.zip › HR-02/43_4.tiff]

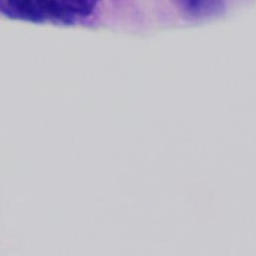

Supplement: Supplementary file 7 [file Data_Sheet_5.zip › HR-02/43_5.tiff]

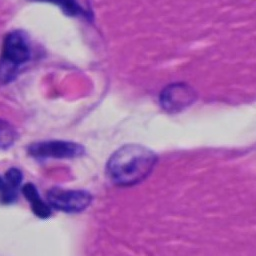

Supplement: Supplementary file 7 [file Data_Sheet_5.zip › HR-02/43_6.tiff]

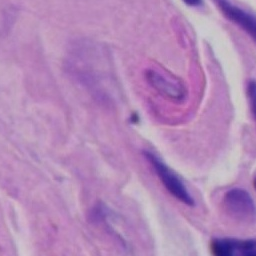

Supplement: Supplementary file 7 [file Data_Sheet_5.zip › HR-02/43_7.tiff]

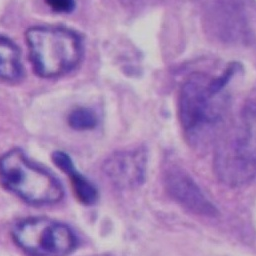

Supplement: Supplementary file 7 [file Data_Sheet_5.zip › HR-02/44_0.tiff]

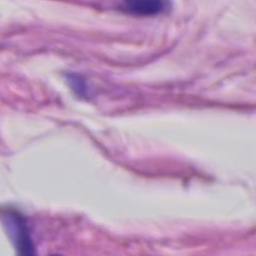

Supplement: Supplementary file 7 [file Data_Sheet_5.zip › HR-02/44_1.tiff]

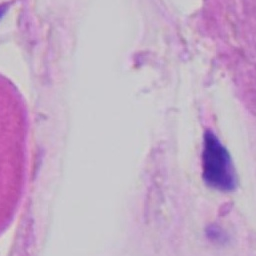

Supplement: Supplementary file 7 [file Data_Sheet_5.zip › HR-02/44_2.tiff]

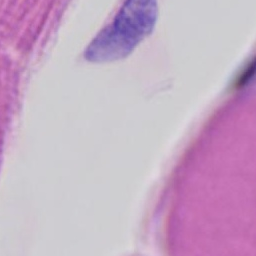

Supplement: Supplementary file 7 [file Data_Sheet_5.zip › HR-02/44_3.tiff]

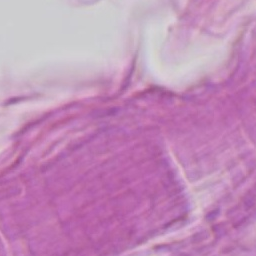

Supplement: Supplementary file 7 [file Data_Sheet_5.zip › HR-02/44_4.tiff]

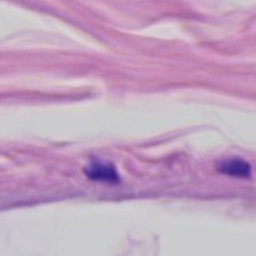

Supplement: Supplementary file 7 [file Data_Sheet_5.zip › HR-02/44_5.tiff]

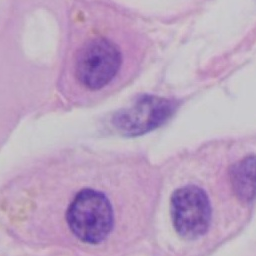

Supplement: Supplementary file 7 [file Data_Sheet_5.zip › HR-02/44_6.tiff]

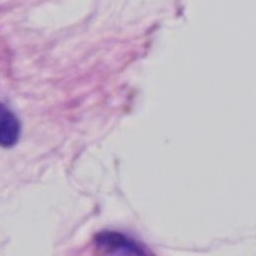

Supplement: Supplementary file 7 [file Data_Sheet_5.zip › HR-02/44_7.tiff]

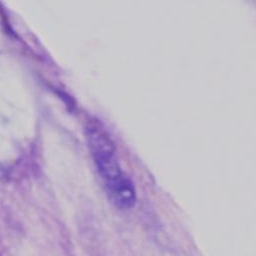

Supplement: Supplementary file 7 [file Data_Sheet_5.zip › HR-02/45_0.tiff]

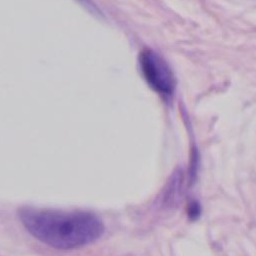

Supplement: Supplementary file 7 [file Data_Sheet_5.zip › HR-02/45_1.tiff]

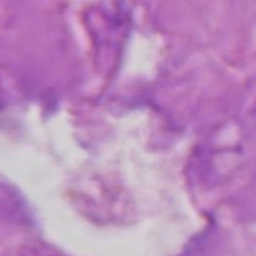

Supplement: Supplementary file 7 [file Data_Sheet_5.zip › HR-02/45_2.tiff]

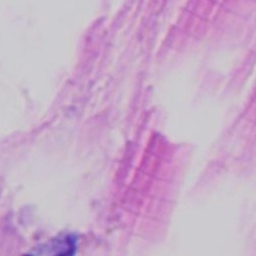

Supplement: Supplementary file 7 [file Data_Sheet_5.zip › HR-02/45_3.tiff]

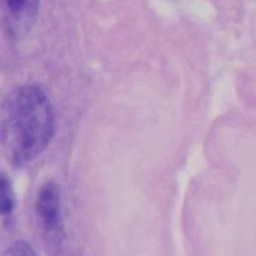

Supplement: Supplementary file 7 [file Data_Sheet_5.zip › HR-02/45_4.tiff]

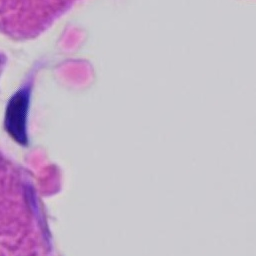

Supplement: Supplementary file 7 [file Data_Sheet_5.zip › HR-02/45_5.tiff]

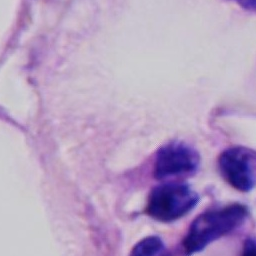

Supplement: Supplementary file 7 [file Data_Sheet_5.zip › HR-02/45_6.tiff]

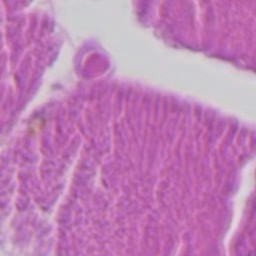

Supplement: Supplementary file 7 [file Data_Sheet_5.zip › HR-02/45_7.tiff]

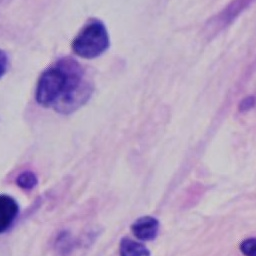

Supplement: Supplementary file 8 [file Data_Sheet_6.zip › HR-03/51_0.tiff]

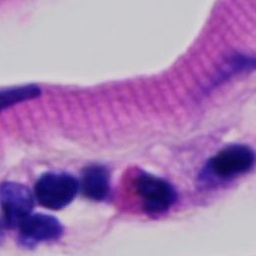

Supplement: Supplementary file 8 [file Data_Sheet_6.zip › HR-03/51_1.tiff]

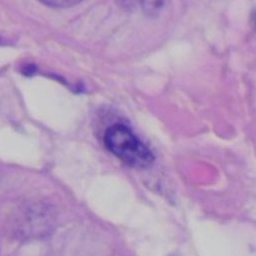

Supplement: Supplementary file 8 [file Data_Sheet_6.zip › HR-03/51_2.tiff]

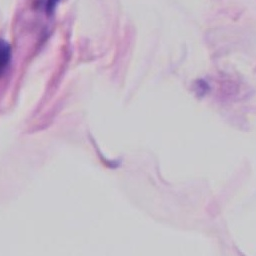

Supplement: Supplementary file 8 [file Data_Sheet_6.zip › HR-03/51_3.tiff]

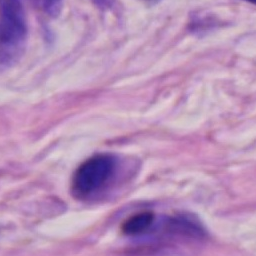

Supplement: Supplementary file 8 [file Data_Sheet_6.zip › HR-03/51_4.tiff]

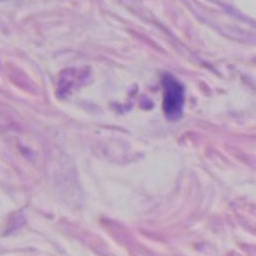

Supplement: Supplementary file 8 [file Data_Sheet_6.zip › HR-03/51_5.tiff]

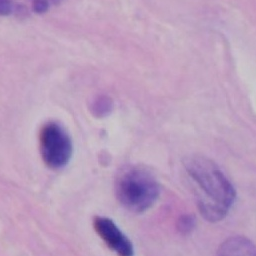

Supplement: Supplementary file 8 [file Data_Sheet_6.zip › HR-03/51_6.tiff]

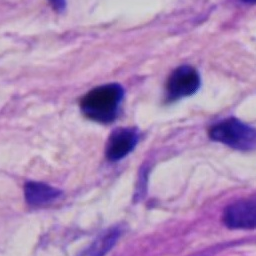

Supplement: Supplementary file 8 [file Data_Sheet_6.zip › HR-03/51_7.tiff]

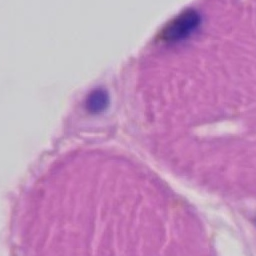

Supplement: Supplementary file 8 [file Data_Sheet_6.zip › HR-03/52_0.tiff]

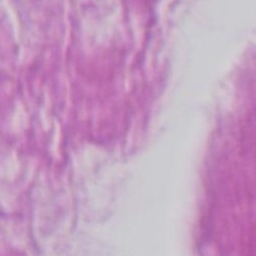

Supplement: Supplementary file 8 [file Data_Sheet_6.zip › HR-03/52_1.tiff]

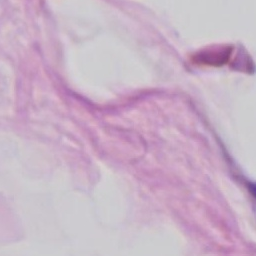

Supplement: Supplementary file 8 [file Data_Sheet_6.zip › HR-03/52_2.tiff]

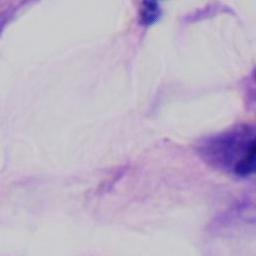

Supplement: Supplementary file 8 [file Data_Sheet_6.zip › HR-03/52_3.tiff]

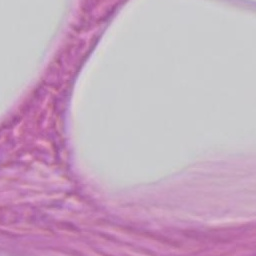

Supplement: Supplementary file 8 [file Data_Sheet_6.zip › HR-03/52_4.tiff]

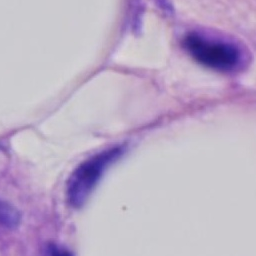

Supplement: Supplementary file 8 [file Data_Sheet_6.zip › HR-03/52_5.tiff]

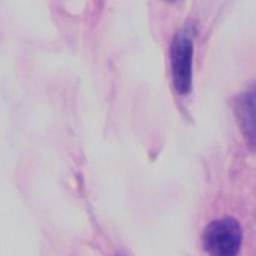

Supplement: Supplementary file 8 [file Data_Sheet_6.zip › HR-03/52_6.tiff]

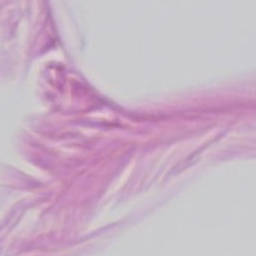

Supplement: Supplementary file 8 [file Data_Sheet_6.zip › HR-03/52_7.tiff]

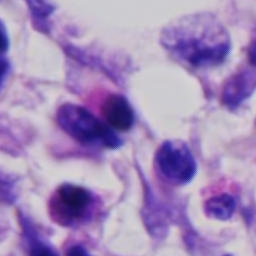

Supplement: Supplementary file 8 [file Data_Sheet_6.zip › HR-03/53_0.tiff]

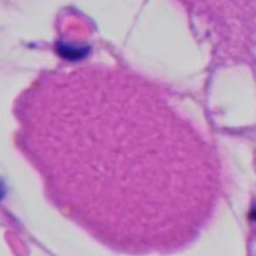

Supplement: Supplementary file 8 [file Data_Sheet_6.zip › HR-03/53_1.tiff]

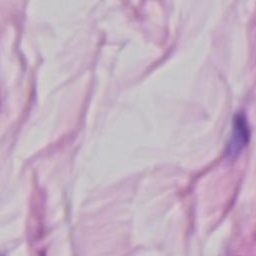

Supplement: Supplementary file 8 [file Data_Sheet_6.zip › HR-03/53_2.tiff]

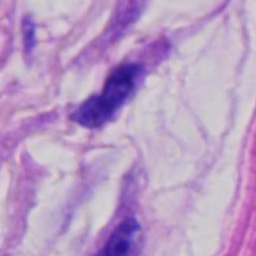

Supplement: Supplementary file 8 [file Data_Sheet_6.zip › HR-03/53_3.tiff]

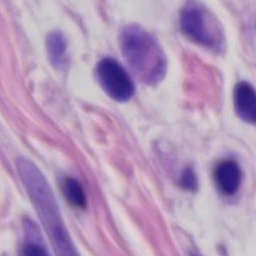

Supplement: Supplementary file 8 [file Data_Sheet_6.zip › HR-03/53_4.tiff]

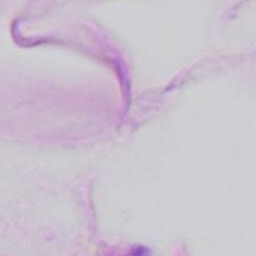

Supplement: Supplementary file 8 [file Data_Sheet_6.zip › HR-03/53_5.tiff]

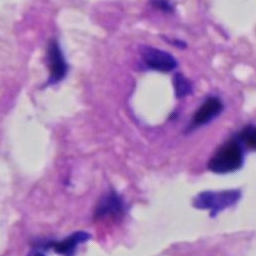

Supplement: Supplementary file 8 [file Data_Sheet_6.zip › HR-03/53_6.tiff]

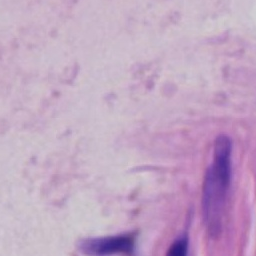

Supplement: Supplementary file 8 [file Data_Sheet_6.zip › HR-03/53_7.tiff]

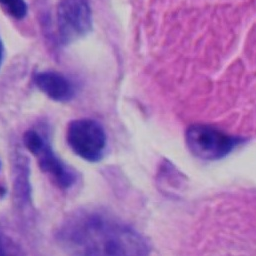

Supplement: Supplementary file 8 [file Data_Sheet_6.zip › HR-03/54_0.tiff]

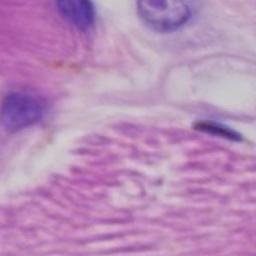

Supplement: Supplementary file 8 [file Data_Sheet_6.zip › HR-03/54_1.tiff]

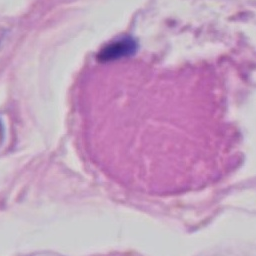

Supplement: Supplementary file 8 [file Data_Sheet_6.zip › HR-03/54_2.tiff]

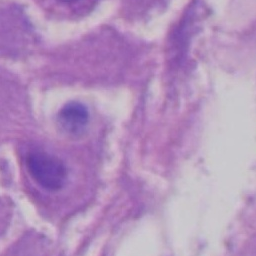

Supplement: Supplementary file 8 [file Data_Sheet_6.zip › HR-03/54_3.tiff]

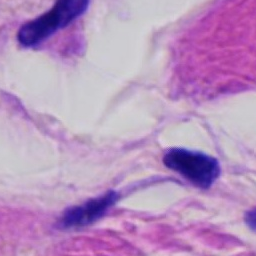

Supplement: Supplementary file 8 [file Data_Sheet_6.zip › HR-03/54_4.tiff]

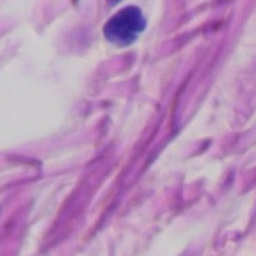

Supplement: Supplementary file 8 [file Data_Sheet_6.zip › HR-03/54_5.tiff]

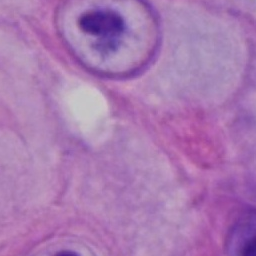

Supplement: Supplementary file 8 [file Data_Sheet_6.zip › HR-03/54_6.tiff]

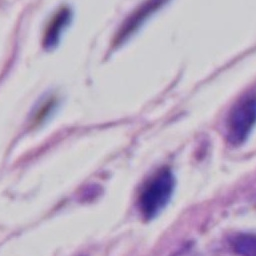

Supplement: Supplementary file 8 [file Data_Sheet_6.zip › HR-03/54_7.tiff]
