# Supplementary figures and images for: Feasibility study of single-image super-resolution scanning system based on deep learning for pathological diagnosis of oral epithelial dysplasia (part 3 of 21)
Source: Front Med (Lausanne). 2025 Mar 12;12:1550512. doi: 10.3389/fmed.2025.1550512 (PMC11936936; doi:10.3389/fmed.2025.1550512)

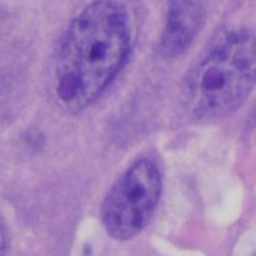

Supplement: Supplementary file 7 [file Data_Sheet_5.zip › HR-02/25_0.tiff]

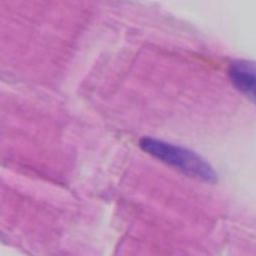

Supplement: Supplementary file 7 [file Data_Sheet_5.zip › HR-02/25_1.tiff]

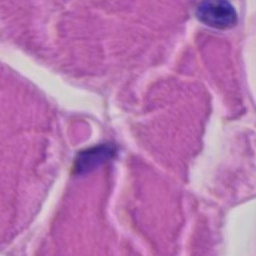

Supplement: Supplementary file 7 [file Data_Sheet_5.zip › HR-02/25_2.tiff]

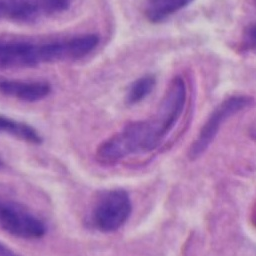

Supplement: Supplementary file 7 [file Data_Sheet_5.zip › HR-02/25_3.tiff]

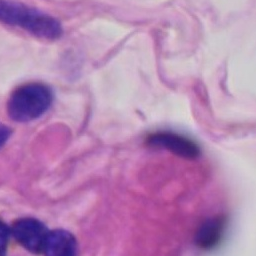

Supplement: Supplementary file 7 [file Data_Sheet_5.zip › HR-02/25_4.tiff]

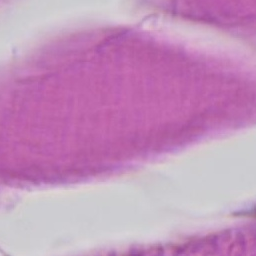

Supplement: Supplementary file 7 [file Data_Sheet_5.zip › HR-02/25_5.tiff]

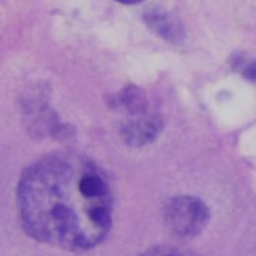

Supplement: Supplementary file 7 [file Data_Sheet_5.zip › HR-02/25_6.tiff]

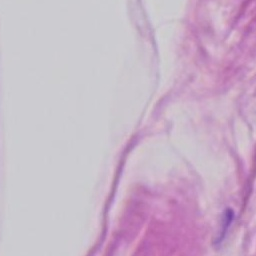

Supplement: Supplementary file 7 [file Data_Sheet_5.zip › HR-02/25_7.tiff]

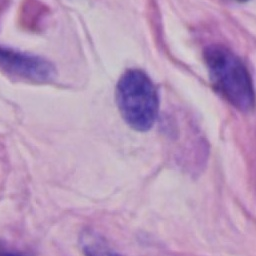

Supplement: Supplementary file 7 [file Data_Sheet_5.zip › HR-02/26_0.tiff]

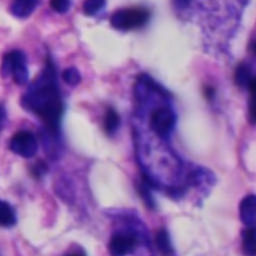

Supplement: Supplementary file 7 [file Data_Sheet_5.zip › HR-02/26_1.tiff]

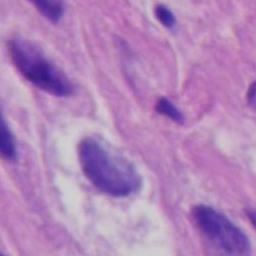

Supplement: Supplementary file 7 [file Data_Sheet_5.zip › HR-02/26_2.tiff]

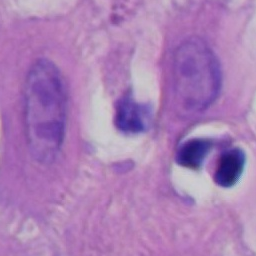

Supplement: Supplementary file 7 [file Data_Sheet_5.zip › HR-02/26_3.tiff]

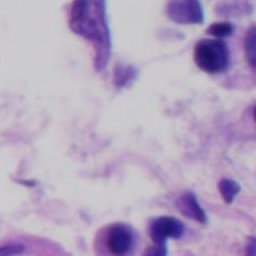

Supplement: Supplementary file 7 [file Data_Sheet_5.zip › HR-02/26_4.tiff]

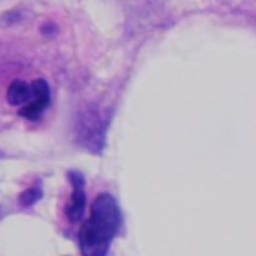

Supplement: Supplementary file 7 [file Data_Sheet_5.zip › HR-02/26_5.tiff]

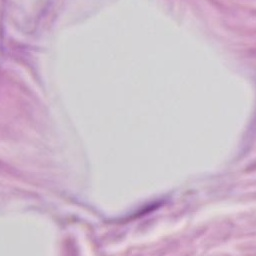

Supplement: Supplementary file 7 [file Data_Sheet_5.zip › HR-02/26_6.tiff]

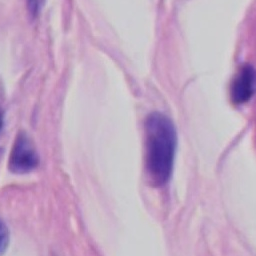

Supplement: Supplementary file 7 [file Data_Sheet_5.zip › HR-02/26_7.tiff]

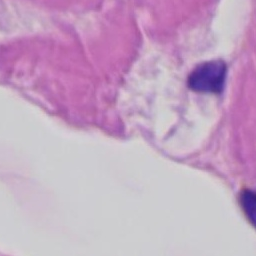

Supplement: Supplementary file 7 [file Data_Sheet_5.zip › HR-02/27_0.tiff]

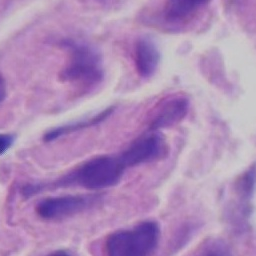

Supplement: Supplementary file 7 [file Data_Sheet_5.zip › HR-02/27_1.tiff]

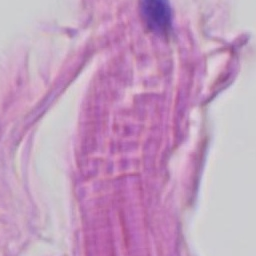

Supplement: Supplementary file 7 [file Data_Sheet_5.zip › HR-02/27_2.tiff]

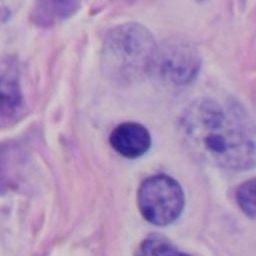

Supplement: Supplementary file 7 [file Data_Sheet_5.zip › HR-02/27_3.tiff]

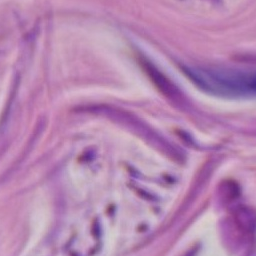

Supplement: Supplementary file 7 [file Data_Sheet_5.zip › HR-02/27_4.tiff]

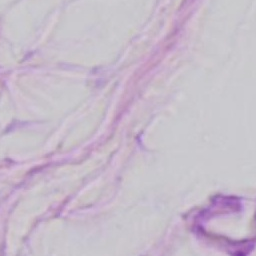

Supplement: Supplementary file 7 [file Data_Sheet_5.zip › HR-02/27_5.tiff]

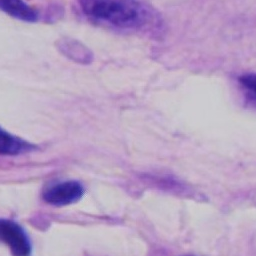

Supplement: Supplementary file 7 [file Data_Sheet_5.zip › HR-02/27_6.tiff]

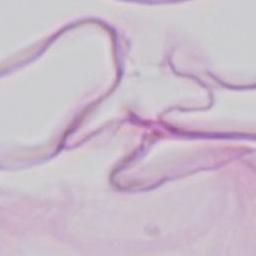

Supplement: Supplementary file 7 [file Data_Sheet_5.zip › HR-02/27_7.tiff]

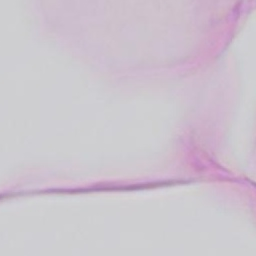

Supplement: Supplementary file 7 [file Data_Sheet_5.zip › HR-02/28_0.tiff]

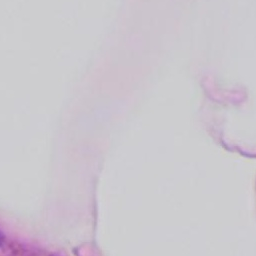

Supplement: Supplementary file 7 [file Data_Sheet_5.zip › HR-02/28_1.tiff]

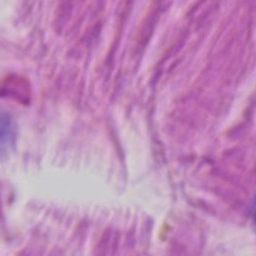

Supplement: Supplementary file 7 [file Data_Sheet_5.zip › HR-02/28_2.tiff]

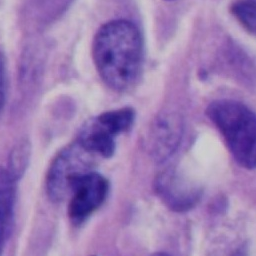

Supplement: Supplementary file 7 [file Data_Sheet_5.zip › HR-02/28_3.tiff]

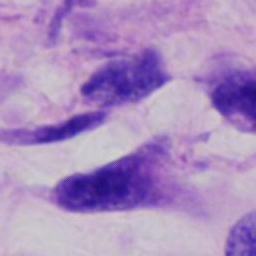

Supplement: Supplementary file 7 [file Data_Sheet_5.zip › HR-02/28_4.tiff]

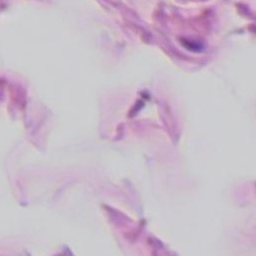

Supplement: Supplementary file 7 [file Data_Sheet_5.zip › HR-02/28_5.tiff]

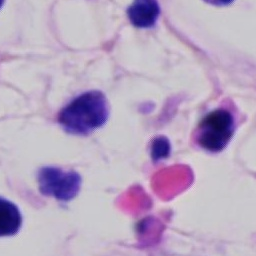

Supplement: Supplementary file 7 [file Data_Sheet_5.zip › HR-02/28_6.tiff]

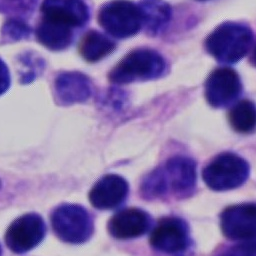

Supplement: Supplementary file 7 [file Data_Sheet_5.zip › HR-02/28_7.tiff]

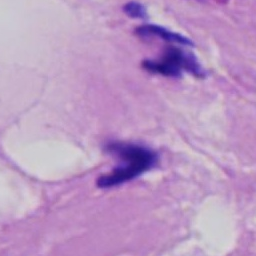

Supplement: Supplementary file 7 [file Data_Sheet_5.zip › HR-02/29_0.tiff]

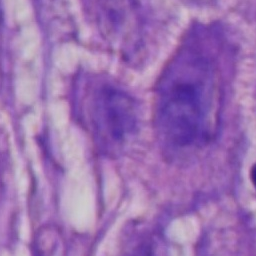

Supplement: Supplementary file 7 [file Data_Sheet_5.zip › HR-02/29_1.tiff]

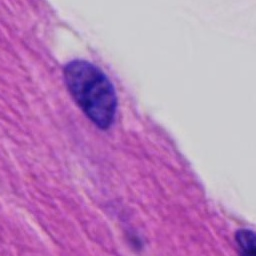

Supplement: Supplementary file 7 [file Data_Sheet_5.zip › HR-02/29_2.tiff]

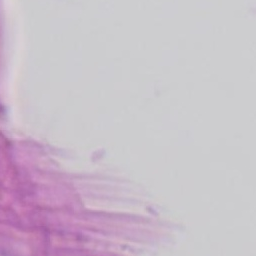

Supplement: Supplementary file 7 [file Data_Sheet_5.zip › HR-02/29_3.tiff]

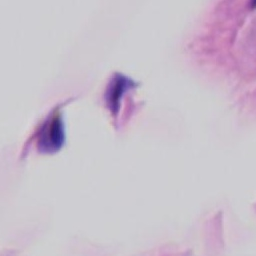

Supplement: Supplementary file 7 [file Data_Sheet_5.zip › HR-02/29_4.tiff]

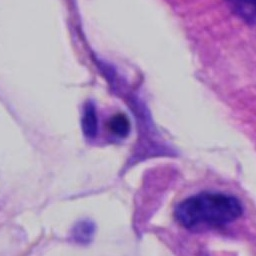

Supplement: Supplementary file 7 [file Data_Sheet_5.zip › HR-02/29_5.tiff]

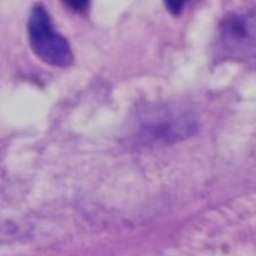

Supplement: Supplementary file 7 [file Data_Sheet_5.zip › HR-02/29_6.tiff]

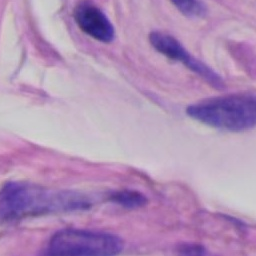

Supplement: Supplementary file 7 [file Data_Sheet_5.zip › HR-02/29_7.tiff]

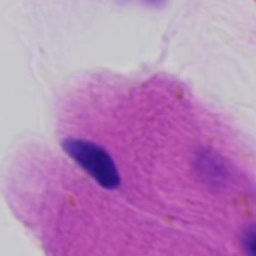

Supplement: Supplementary file 7 [file Data_Sheet_5.zip › HR-02/30_0.tiff]

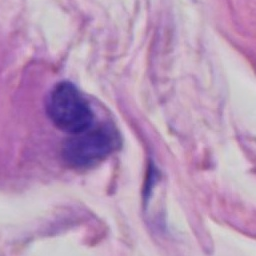

Supplement: Supplementary file 7 [file Data_Sheet_5.zip › HR-02/30_1.tiff]

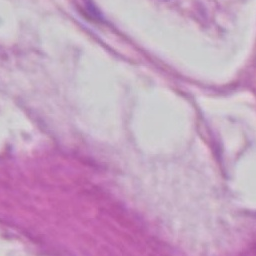

Supplement: Supplementary file 7 [file Data_Sheet_5.zip › HR-02/30_2.tiff]

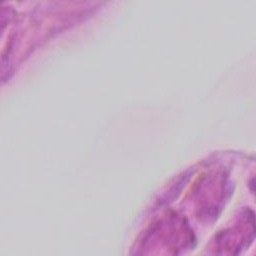

Supplement: Supplementary file 7 [file Data_Sheet_5.zip › HR-02/30_3.tiff]

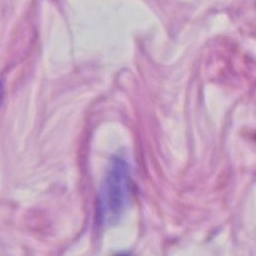

Supplement: Supplementary file 7 [file Data_Sheet_5.zip › HR-02/30_4.tiff]

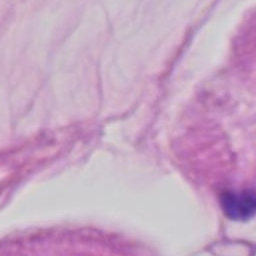

Supplement: Supplementary file 7 [file Data_Sheet_5.zip › HR-02/30_5.tiff]

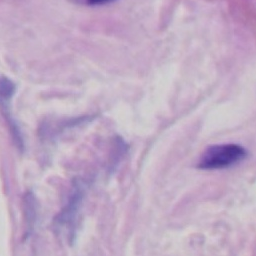

Supplement: Supplementary file 7 [file Data_Sheet_5.zip › HR-02/30_6.tiff]

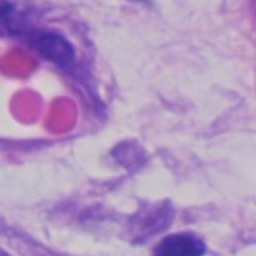

Supplement: Supplementary file 7 [file Data_Sheet_5.zip › HR-02/30_7.tiff]

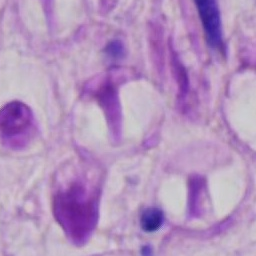

Supplement: Supplementary file 7 [file Data_Sheet_5.zip › HR-02/31_0.tiff]

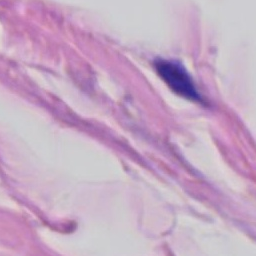

Supplement: Supplementary file 7 [file Data_Sheet_5.zip › HR-02/31_1.tiff]

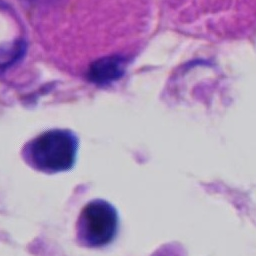

Supplement: Supplementary file 7 [file Data_Sheet_5.zip › HR-02/31_2.tiff]

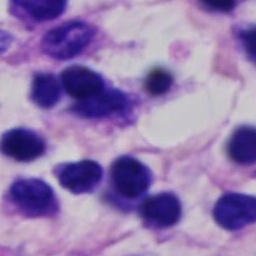

Supplement: Supplementary file 7 [file Data_Sheet_5.zip › HR-02/31_3.tiff]

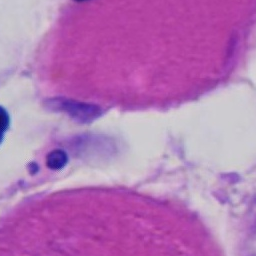

Supplement: Supplementary file 7 [file Data_Sheet_5.zip › HR-02/31_4.tiff]

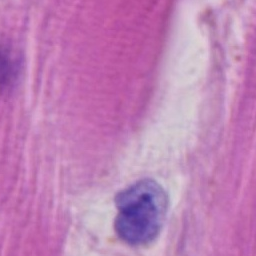

Supplement: Supplementary file 7 [file Data_Sheet_5.zip › HR-02/31_5.tiff]

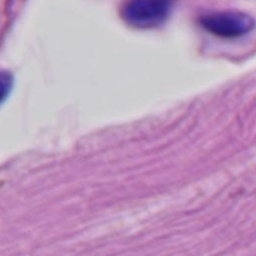

Supplement: Supplementary file 7 [file Data_Sheet_5.zip › HR-02/31_6.tiff]

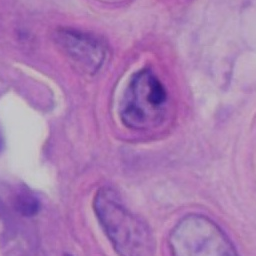

Supplement: Supplementary file 7 [file Data_Sheet_5.zip › HR-02/31_7.tiff]

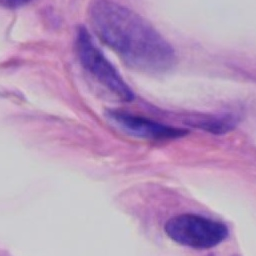

Supplement: Supplementary file 7 [file Data_Sheet_5.zip › HR-02/32_0.tiff]

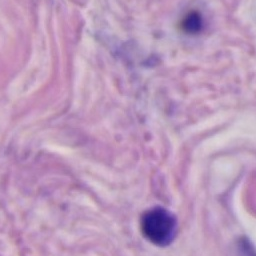

Supplement: Supplementary file 7 [file Data_Sheet_5.zip › HR-02/32_1.tiff]

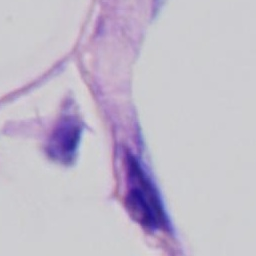

Supplement: Supplementary file 7 [file Data_Sheet_5.zip › HR-02/32_2.tiff]

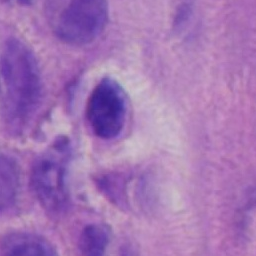

Supplement: Supplementary file 7 [file Data_Sheet_5.zip › HR-02/32_3.tiff]

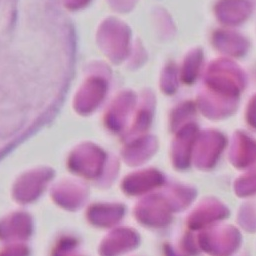

Supplement: Supplementary file 7 [file Data_Sheet_5.zip › HR-02/32_4.tiff]

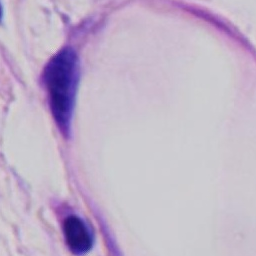

Supplement: Supplementary file 7 [file Data_Sheet_5.zip › HR-02/32_5.tiff]

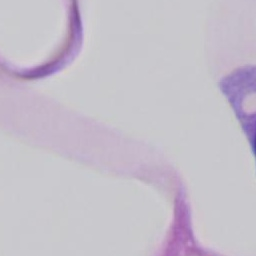

Supplement: Supplementary file 7 [file Data_Sheet_5.zip › HR-02/32_6.tiff]

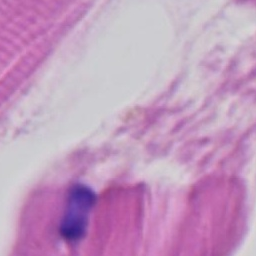

Supplement: Supplementary file 7 [file Data_Sheet_5.zip › HR-02/32_7.tiff]

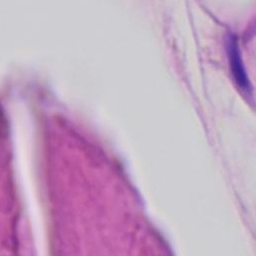

Supplement: Supplementary file 7 [file Data_Sheet_5.zip › HR-02/33_0.tiff]

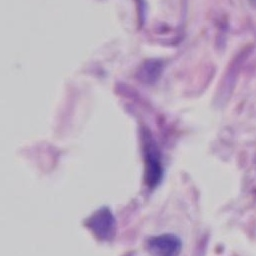

Supplement: Supplementary file 7 [file Data_Sheet_5.zip › HR-02/33_1.tiff]

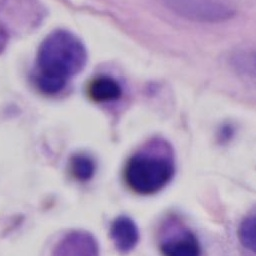

Supplement: Supplementary file 7 [file Data_Sheet_5.zip › HR-02/33_2.tiff]

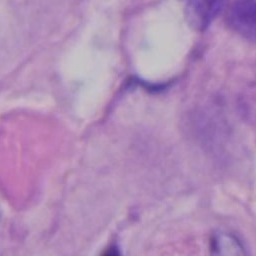

Supplement: Supplementary file 7 [file Data_Sheet_5.zip › HR-02/33_3.tiff]

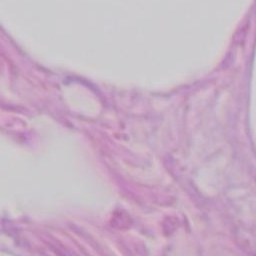

Supplement: Supplementary file 7 [file Data_Sheet_5.zip › HR-02/33_4.tiff]

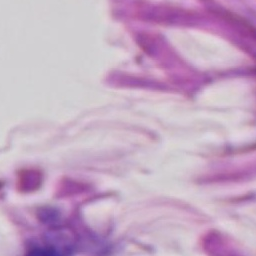

Supplement: Supplementary file 7 [file Data_Sheet_5.zip › HR-02/33_5.tiff]

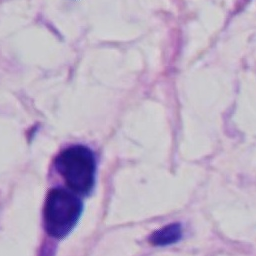

Supplement: Supplementary file 7 [file Data_Sheet_5.zip › HR-02/33_6.tiff]

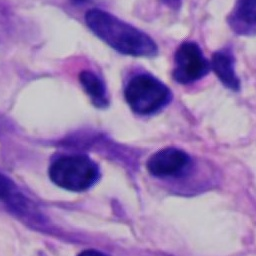

Supplement: Supplementary file 7 [file Data_Sheet_5.zip › HR-02/33_7.tiff]

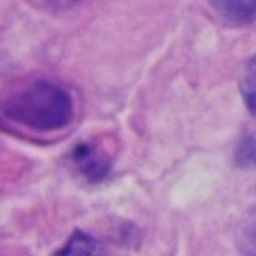

Supplement: Supplementary file 7 [file Data_Sheet_5.zip › HR-02/34_0.tiff]

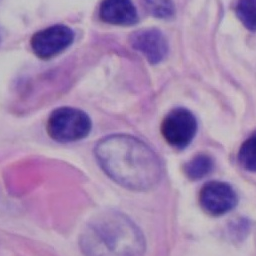

Supplement: Supplementary file 7 [file Data_Sheet_5.zip › HR-02/34_1.tiff]

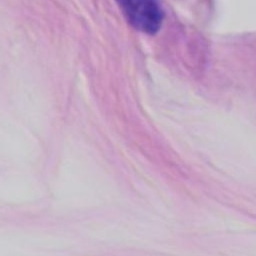

Supplement: Supplementary file 7 [file Data_Sheet_5.zip › HR-02/34_2.tiff]

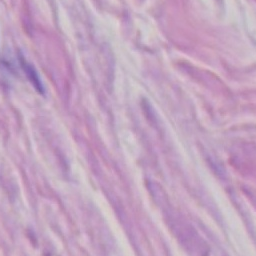

Supplement: Supplementary file 7 [file Data_Sheet_5.zip › HR-02/34_3.tiff]

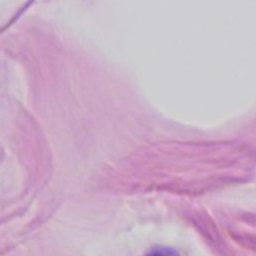

Supplement: Supplementary file 7 [file Data_Sheet_5.zip › HR-02/34_4.tiff]

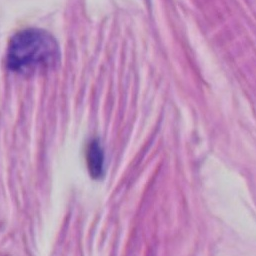

Supplement: Supplementary file 7 [file Data_Sheet_5.zip › HR-02/34_5.tiff]

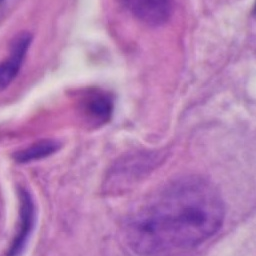

Supplement: Supplementary file 7 [file Data_Sheet_5.zip › HR-02/34_6.tiff]

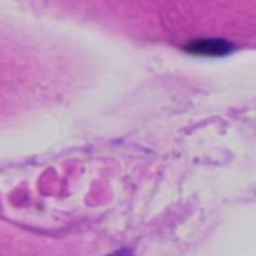

Supplement: Supplementary file 7 [file Data_Sheet_5.zip › HR-02/34_7.tiff]

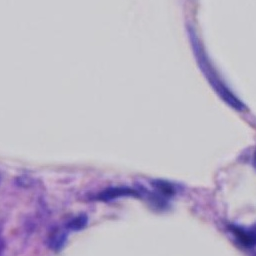

Supplement: Supplementary file 7 [file Data_Sheet_5.zip › HR-02/35_0.tiff]

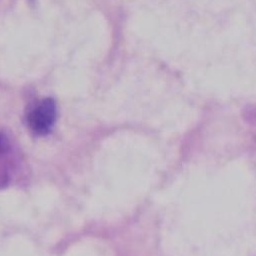

Supplement: Supplementary file 7 [file Data_Sheet_5.zip › HR-02/35_1.tiff]

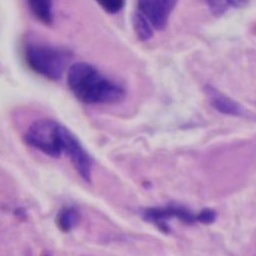

Supplement: Supplementary file 7 [file Data_Sheet_5.zip › HR-02/35_2.tiff]

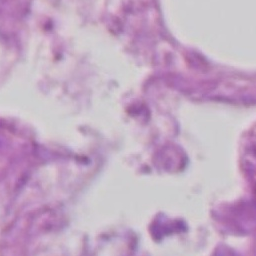

Supplement: Supplementary file 7 [file Data_Sheet_5.zip › HR-02/35_3.tiff]

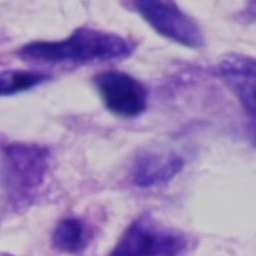

Supplement: Supplementary file 7 [file Data_Sheet_5.zip › HR-02/35_4.tiff]

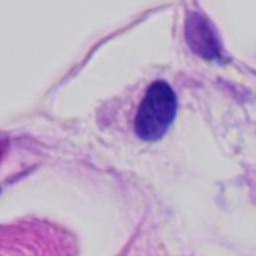

Supplement: Supplementary file 7 [file Data_Sheet_5.zip › HR-02/35_5.tiff]

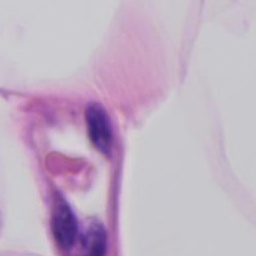

Supplement: Supplementary file 7 [file Data_Sheet_5.zip › HR-02/35_6.tiff]

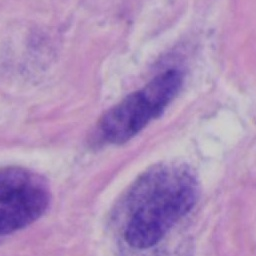

Supplement: Supplementary file 7 [file Data_Sheet_5.zip › HR-02/35_7.tiff]

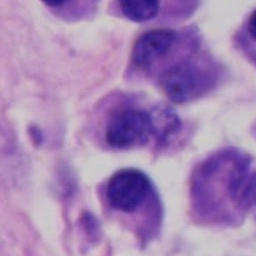

Supplement: Supplementary file 7 [file Data_Sheet_5.zip › HR-02/36_0.tiff]

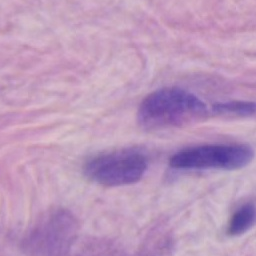

Supplement: Supplementary file 7 [file Data_Sheet_5.zip › HR-02/36_1.tiff]

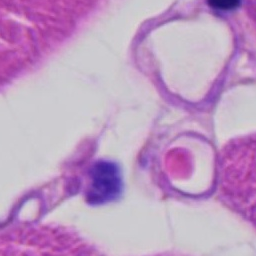

Supplement: Supplementary file 7 [file Data_Sheet_5.zip › HR-02/36_2.tiff]

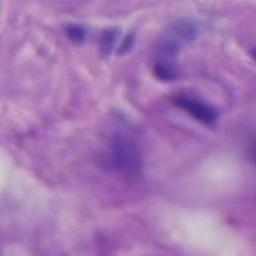

Supplement: Supplementary file 7 [file Data_Sheet_5.zip › HR-02/36_3.tiff]

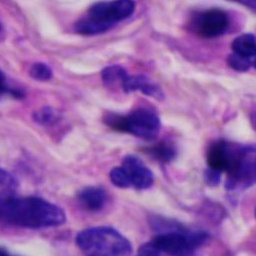

Supplement: Supplementary file 7 [file Data_Sheet_5.zip › HR-02/36_4.tiff]

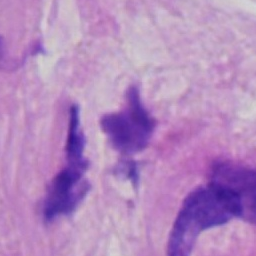

Supplement: Supplementary file 7 [file Data_Sheet_5.zip › HR-02/36_5.tiff]

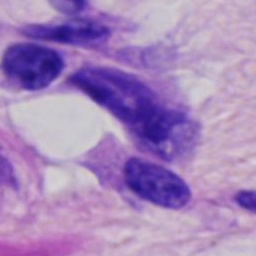

Supplement: Supplementary file 7 [file Data_Sheet_5.zip › HR-02/36_6.tiff]

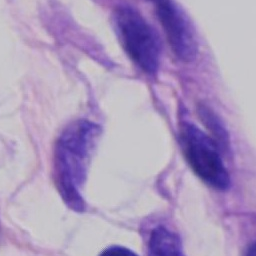

Supplement: Supplementary file 7 [file Data_Sheet_5.zip › HR-02/36_7.tiff]

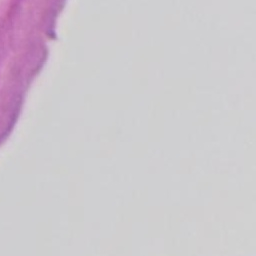

Supplement: Supplementary file 7 [file Data_Sheet_5.zip › HR-02/37_0.tiff]

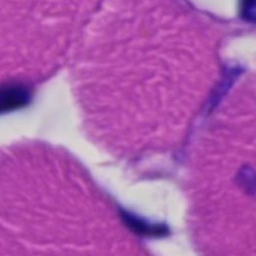

Supplement: Supplementary file 7 [file Data_Sheet_5.zip › HR-02/37_1.tiff]

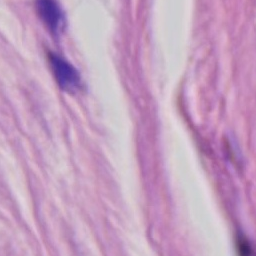

Supplement: Supplementary file 7 [file Data_Sheet_5.zip › HR-02/37_2.tiff]

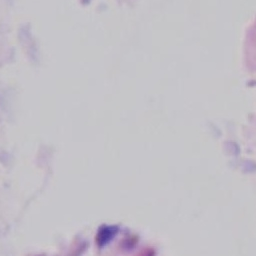

Supplement: Supplementary file 7 [file Data_Sheet_5.zip › HR-02/37_3.tiff]
