# Supplementary figures and images for: Feasibility study of single-image super-resolution scanning system based on deep learning for pathological diagnosis of oral epithelial dysplasia (part 6 of 21)
Source: Front Med (Lausanne). 2025 Mar 12;12:1550512. doi: 10.3389/fmed.2025.1550512 (PMC11936936; doi:10.3389/fmed.2025.1550512)

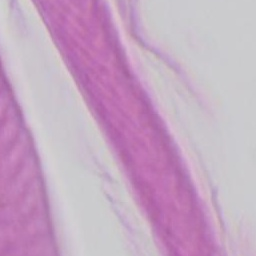

Supplement: Supplementary file 8 [file Data_Sheet_6.zip › HR-03/67_4.tiff]

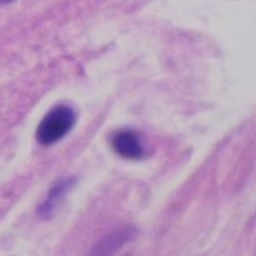

Supplement: Supplementary file 8 [file Data_Sheet_6.zip › HR-03/67_5.tiff]

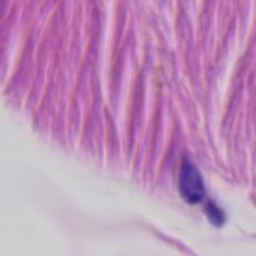

Supplement: Supplementary file 8 [file Data_Sheet_6.zip › HR-03/67_6.tiff]

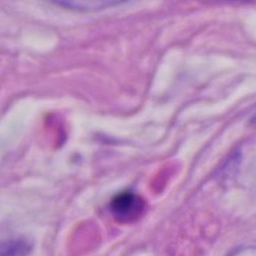

Supplement: Supplementary file 8 [file Data_Sheet_6.zip › HR-03/67_7.tiff]

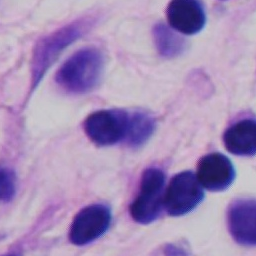

Supplement: Supplementary file 8 [file Data_Sheet_6.zip › HR-03/68_0.tiff]

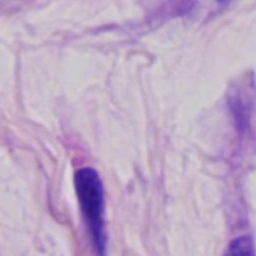

Supplement: Supplementary file 8 [file Data_Sheet_6.zip › HR-03/68_1.tiff]

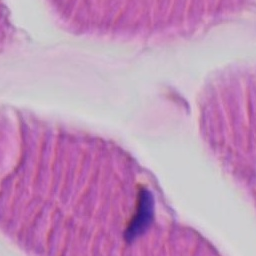

Supplement: Supplementary file 8 [file Data_Sheet_6.zip › HR-03/68_2.tiff]

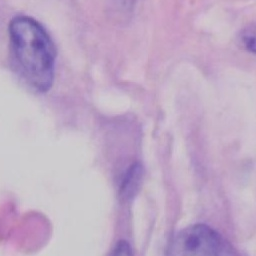

Supplement: Supplementary file 8 [file Data_Sheet_6.zip › HR-03/68_3.tiff]

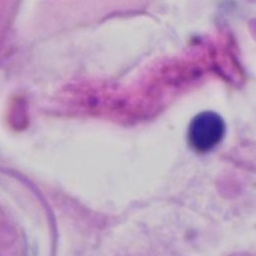

Supplement: Supplementary file 8 [file Data_Sheet_6.zip › HR-03/68_4.tiff]

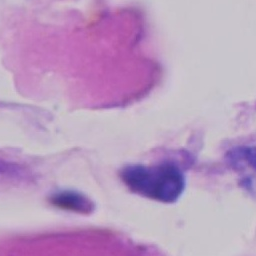

Supplement: Supplementary file 8 [file Data_Sheet_6.zip › HR-03/68_5.tiff]

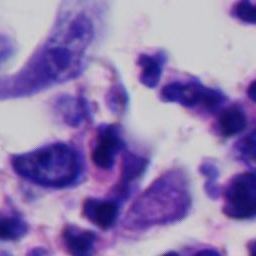

Supplement: Supplementary file 8 [file Data_Sheet_6.zip › HR-03/68_6.tiff]

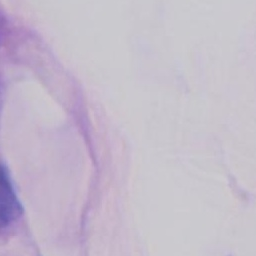

Supplement: Supplementary file 8 [file Data_Sheet_6.zip › HR-03/68_7.tiff]

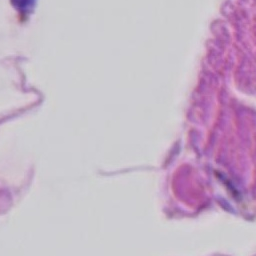

Supplement: Supplementary file 8 [file Data_Sheet_6.zip › HR-03/69_0.tiff]

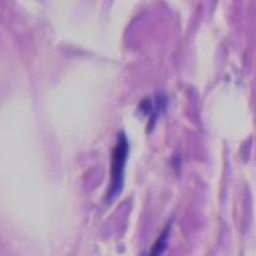

Supplement: Supplementary file 8 [file Data_Sheet_6.zip › HR-03/69_1.tiff]

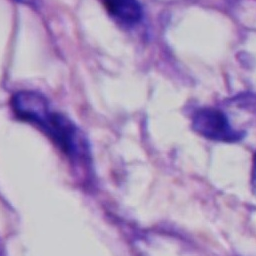

Supplement: Supplementary file 8 [file Data_Sheet_6.zip › HR-03/69_2.tiff]

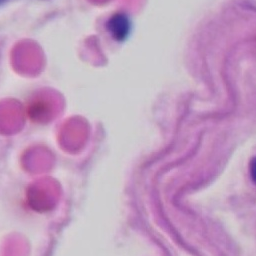

Supplement: Supplementary file 8 [file Data_Sheet_6.zip › HR-03/69_3.tiff]

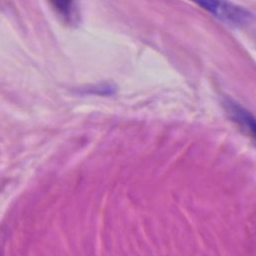

Supplement: Supplementary file 8 [file Data_Sheet_6.zip › HR-03/69_4.tiff]

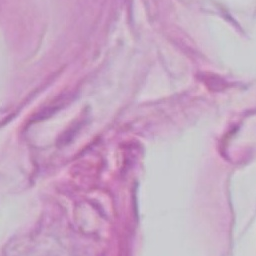

Supplement: Supplementary file 8 [file Data_Sheet_6.zip › HR-03/69_5.tiff]

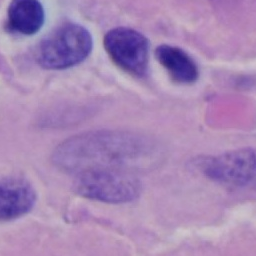

Supplement: Supplementary file 8 [file Data_Sheet_6.zip › HR-03/69_6.tiff]

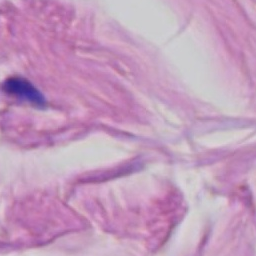

Supplement: Supplementary file 8 [file Data_Sheet_6.zip › HR-03/69_7.tiff]

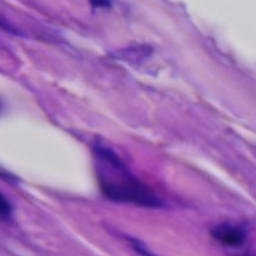

Supplement: Supplementary file 8 [file Data_Sheet_6.zip › HR-03/70_0.tiff]

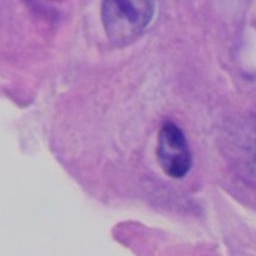

Supplement: Supplementary file 8 [file Data_Sheet_6.zip › HR-03/70_1.tiff]

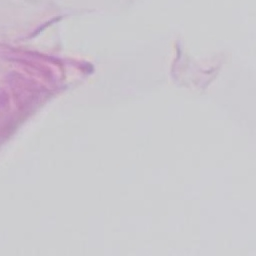

Supplement: Supplementary file 8 [file Data_Sheet_6.zip › HR-03/70_2.tiff]

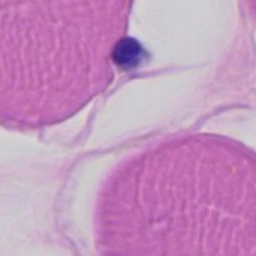

Supplement: Supplementary file 8 [file Data_Sheet_6.zip › HR-03/70_3.tiff]

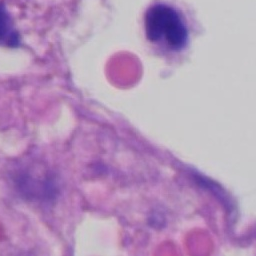

Supplement: Supplementary file 8 [file Data_Sheet_6.zip › HR-03/70_4.tiff]

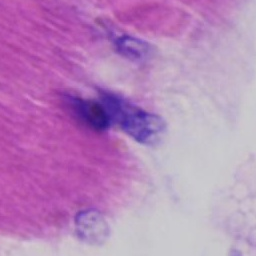

Supplement: Supplementary file 8 [file Data_Sheet_6.zip › HR-03/70_5.tiff]

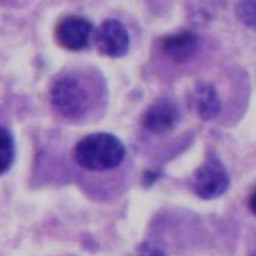

Supplement: Supplementary file 8 [file Data_Sheet_6.zip › HR-03/70_6.tiff]

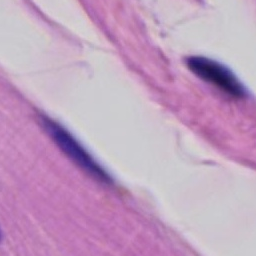

Supplement: Supplementary file 8 [file Data_Sheet_6.zip › HR-03/70_7.tiff]

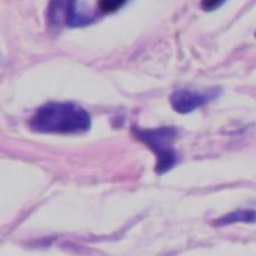

Supplement: Supplementary file 8 [file Data_Sheet_6.zip › HR-03/71_0.tiff]

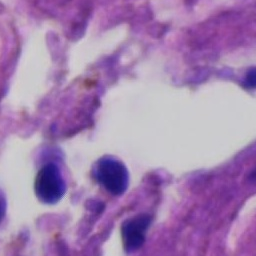

Supplement: Supplementary file 8 [file Data_Sheet_6.zip › HR-03/71_1.tiff]

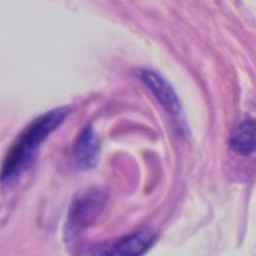

Supplement: Supplementary file 8 [file Data_Sheet_6.zip › HR-03/71_2.tiff]

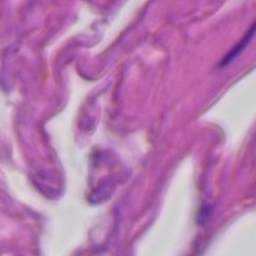

Supplement: Supplementary file 8 [file Data_Sheet_6.zip › HR-03/71_3.tiff]

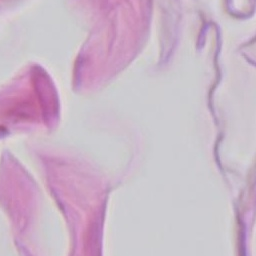

Supplement: Supplementary file 8 [file Data_Sheet_6.zip › HR-03/71_4.tiff]

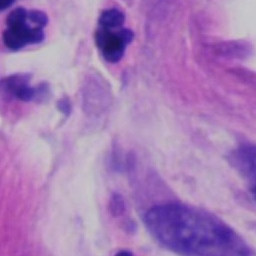

Supplement: Supplementary file 8 [file Data_Sheet_6.zip › HR-03/71_5.tiff]

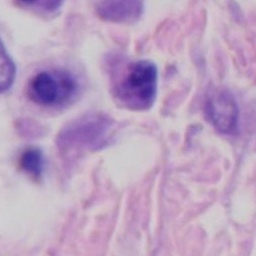

Supplement: Supplementary file 8 [file Data_Sheet_6.zip › HR-03/71_6.tiff]

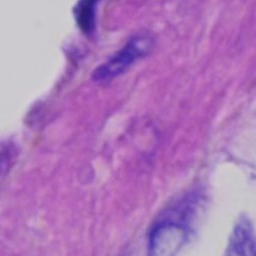

Supplement: Supplementary file 8 [file Data_Sheet_6.zip › HR-03/71_7.tiff]

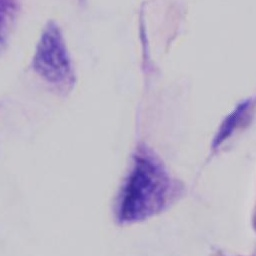

Supplement: Supplementary file 8 [file Data_Sheet_6.zip › HR-03/72_0.tiff]

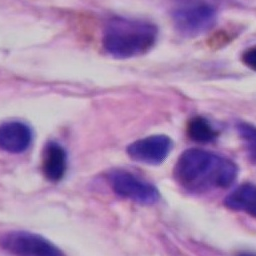

Supplement: Supplementary file 8 [file Data_Sheet_6.zip › HR-03/72_1.tiff]

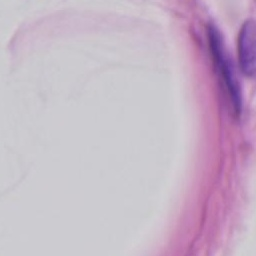

Supplement: Supplementary file 8 [file Data_Sheet_6.zip › HR-03/72_2.tiff]

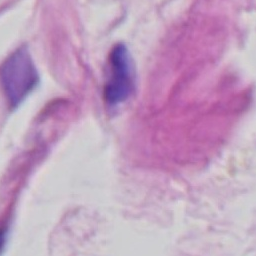

Supplement: Supplementary file 8 [file Data_Sheet_6.zip › HR-03/72_3.tiff]

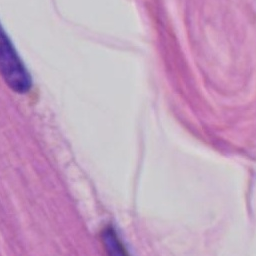

Supplement: Supplementary file 8 [file Data_Sheet_6.zip › HR-03/72_4.tiff]

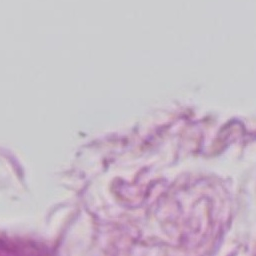

Supplement: Supplementary file 8 [file Data_Sheet_6.zip › HR-03/72_5.tiff]

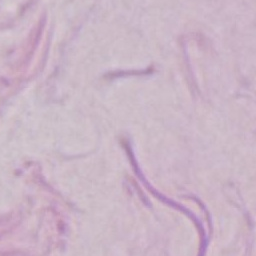

Supplement: Supplementary file 8 [file Data_Sheet_6.zip › HR-03/72_6.tiff]

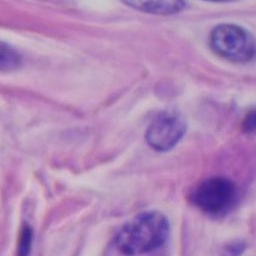

Supplement: Supplementary file 8 [file Data_Sheet_6.zip › HR-03/72_7.tiff]

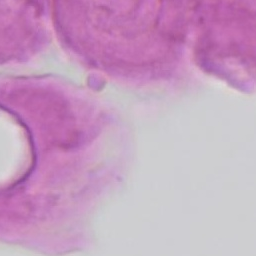

Supplement: Supplementary file 8 [file Data_Sheet_6.zip › HR-03/73_0.tiff]

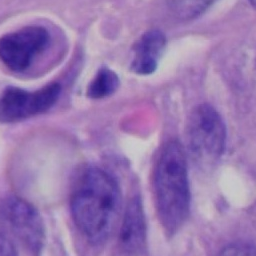

Supplement: Supplementary file 8 [file Data_Sheet_6.zip › HR-03/73_1.tiff]

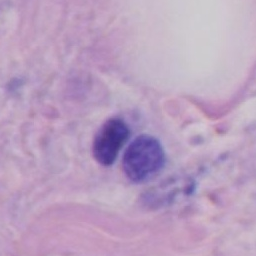

Supplement: Supplementary file 8 [file Data_Sheet_6.zip › HR-03/73_2.tiff]

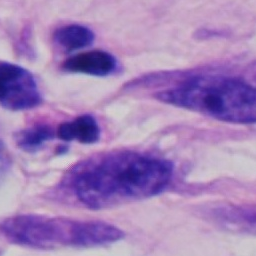

Supplement: Supplementary file 8 [file Data_Sheet_6.zip › HR-03/73_3.tiff]

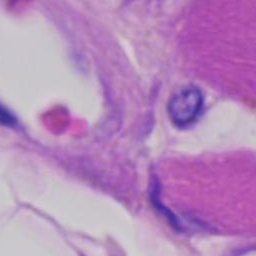

Supplement: Supplementary file 8 [file Data_Sheet_6.zip › HR-03/73_4.tiff]

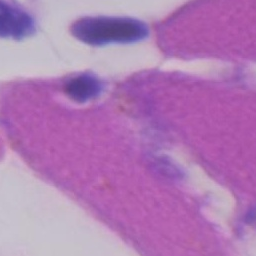

Supplement: Supplementary file 8 [file Data_Sheet_6.zip › HR-03/73_5.tiff]

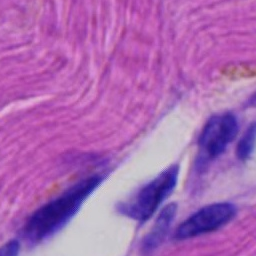

Supplement: Supplementary file 8 [file Data_Sheet_6.zip › HR-03/73_6.tiff]

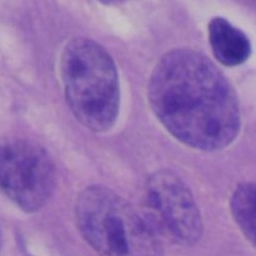

Supplement: Supplementary file 8 [file Data_Sheet_6.zip › HR-03/73_7.tiff]

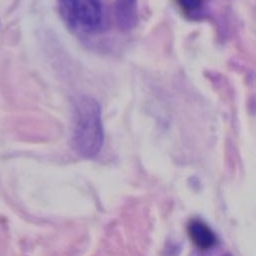

Supplement: Supplementary file 8 [file Data_Sheet_6.zip › HR-03/74_0.tiff]

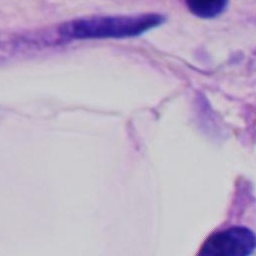

Supplement: Supplementary file 8 [file Data_Sheet_6.zip › HR-03/74_1.tiff]

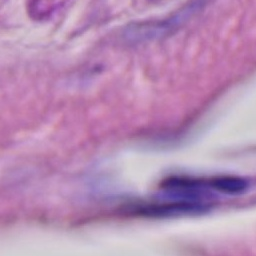

Supplement: Supplementary file 8 [file Data_Sheet_6.zip › HR-03/74_2.tiff]

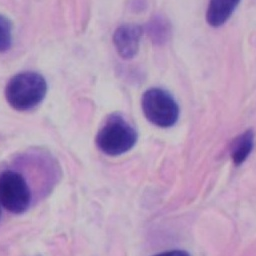

Supplement: Supplementary file 8 [file Data_Sheet_6.zip › HR-03/74_3.tiff]

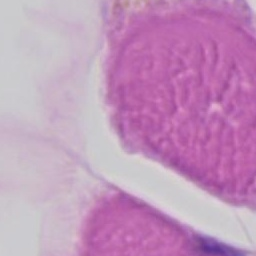

Supplement: Supplementary file 8 [file Data_Sheet_6.zip › HR-03/74_4.tiff]

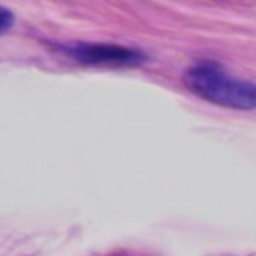

Supplement: Supplementary file 8 [file Data_Sheet_6.zip › HR-03/74_5.tiff]

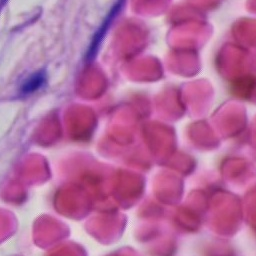

Supplement: Supplementary file 8 [file Data_Sheet_6.zip › HR-03/74_6.tiff]

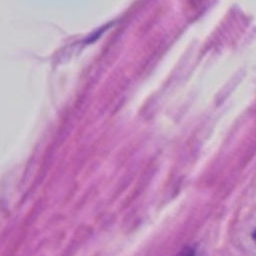

Supplement: Supplementary file 8 [file Data_Sheet_6.zip › HR-03/74_7.tiff]

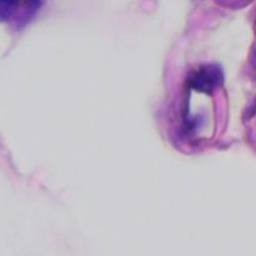

Supplement: Supplementary file 8 [file Data_Sheet_6.zip › HR-03/75_0.tiff]

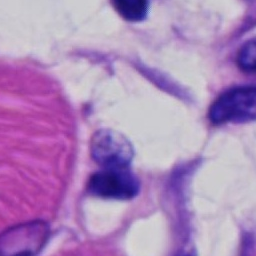

Supplement: Supplementary file 8 [file Data_Sheet_6.zip › HR-03/75_1.tiff]

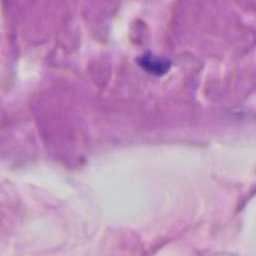

Supplement: Supplementary file 8 [file Data_Sheet_6.zip › HR-03/75_2.tiff]

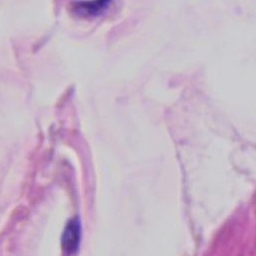

Supplement: Supplementary file 8 [file Data_Sheet_6.zip › HR-03/75_3.tiff]

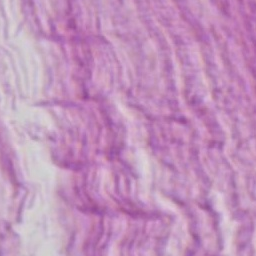

Supplement: Supplementary file 8 [file Data_Sheet_6.zip › HR-03/75_4.tiff]

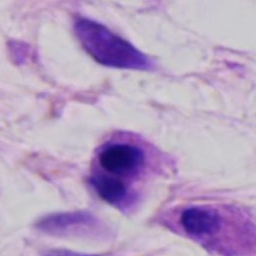

Supplement: Supplementary file 8 [file Data_Sheet_6.zip › HR-03/75_5.tiff]

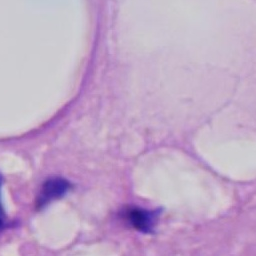

Supplement: Supplementary file 8 [file Data_Sheet_6.zip › HR-03/75_6.tiff]

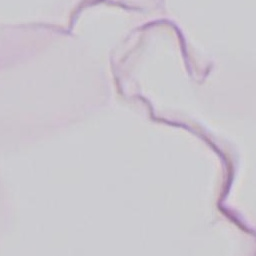

Supplement: Supplementary file 8 [file Data_Sheet_6.zip › HR-03/75_7.tiff]

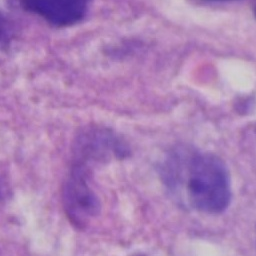

Supplement: Supplementary file 9 [file Data_Sheet_7.zip › HR-04/100_0.tiff]

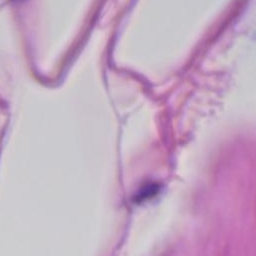

Supplement: Supplementary file 9 [file Data_Sheet_7.zip › HR-04/100_1.tiff]

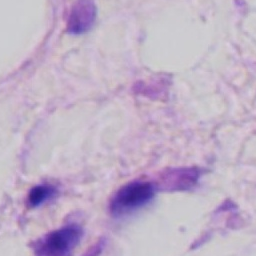

Supplement: Supplementary file 9 [file Data_Sheet_7.zip › HR-04/100_2.tiff]

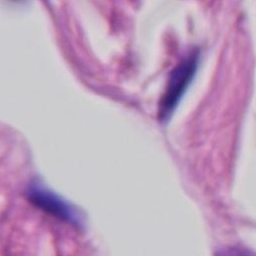

Supplement: Supplementary file 9 [file Data_Sheet_7.zip › HR-04/100_3.tiff]

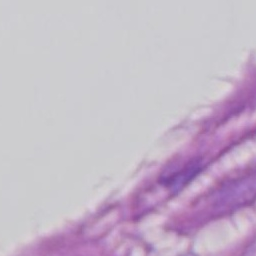

Supplement: Supplementary file 9 [file Data_Sheet_7.zip › HR-04/100_4.tiff]

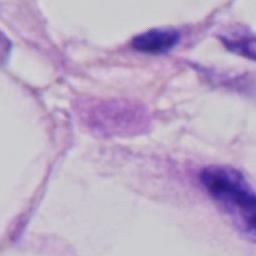

Supplement: Supplementary file 9 [file Data_Sheet_7.zip › HR-04/100_5.tiff]

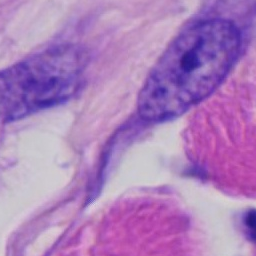

Supplement: Supplementary file 9 [file Data_Sheet_7.zip › HR-04/100_6.tiff]

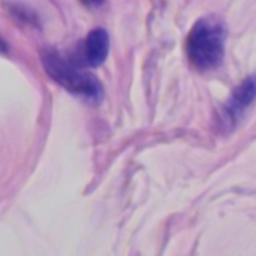

Supplement: Supplementary file 9 [file Data_Sheet_7.zip › HR-04/100_7.tiff]

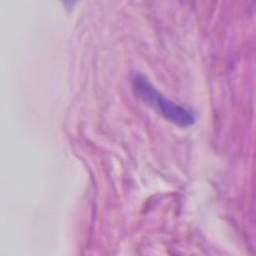

Supplement: Supplementary file 9 [file Data_Sheet_7.zip › HR-04/101_0.tiff]

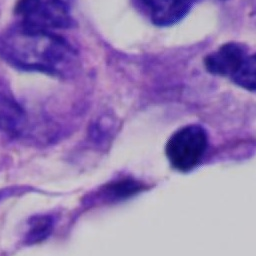

Supplement: Supplementary file 9 [file Data_Sheet_7.zip › HR-04/101_1.tiff]

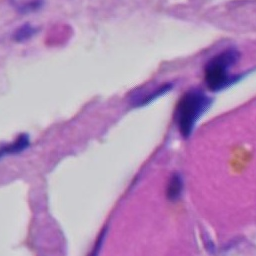

Supplement: Supplementary file 9 [file Data_Sheet_7.zip › HR-04/101_2.tiff]

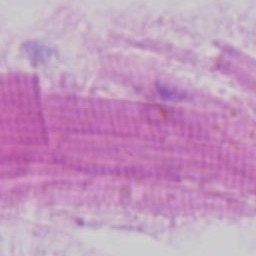

Supplement: Supplementary file 9 [file Data_Sheet_7.zip › HR-04/101_3.tiff]

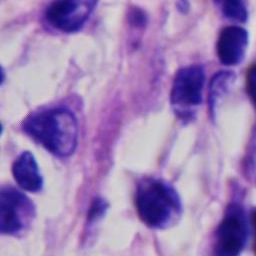

Supplement: Supplementary file 9 [file Data_Sheet_7.zip › HR-04/101_4.tiff]

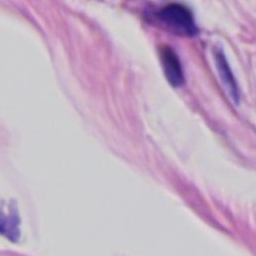

Supplement: Supplementary file 9 [file Data_Sheet_7.zip › HR-04/101_5.tiff]

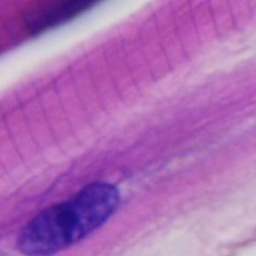

Supplement: Supplementary file 9 [file Data_Sheet_7.zip › HR-04/101_6.tiff]

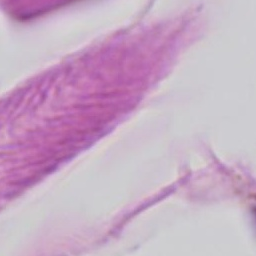

Supplement: Supplementary file 9 [file Data_Sheet_7.zip › HR-04/101_7.tiff]

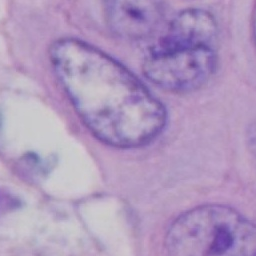

Supplement: Supplementary file 9 [file Data_Sheet_7.zip › HR-04/102_0.tiff]

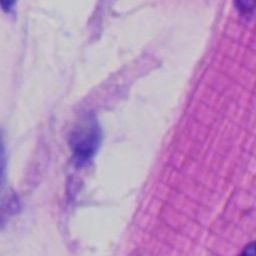

Supplement: Supplementary file 9 [file Data_Sheet_7.zip › HR-04/102_1.tiff]

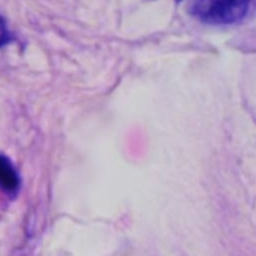

Supplement: Supplementary file 9 [file Data_Sheet_7.zip › HR-04/102_2.tiff]

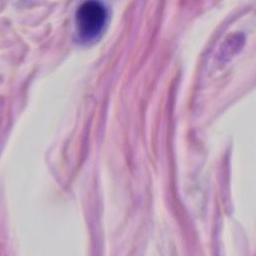

Supplement: Supplementary file 9 [file Data_Sheet_7.zip › HR-04/102_3.tiff]

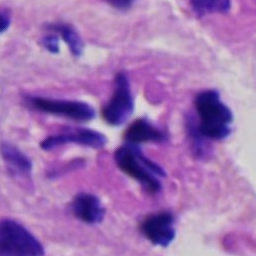

Supplement: Supplementary file 9 [file Data_Sheet_7.zip › HR-04/102_4.tiff]

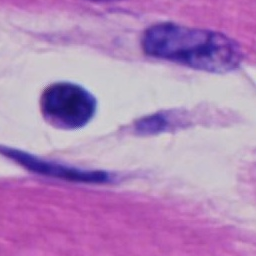

Supplement: Supplementary file 9 [file Data_Sheet_7.zip › HR-04/102_5.tiff]

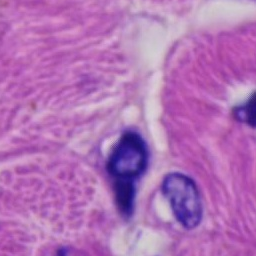

Supplement: Supplementary file 9 [file Data_Sheet_7.zip › HR-04/102_6.tiff]

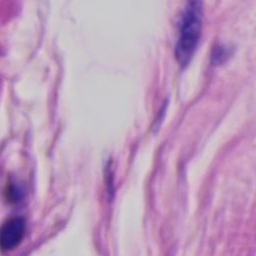

Supplement: Supplementary file 9 [file Data_Sheet_7.zip › HR-04/102_7.tiff]

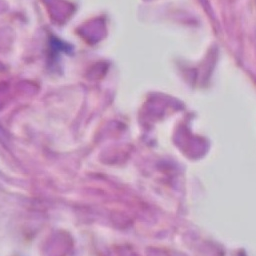

Supplement: Supplementary file 9 [file Data_Sheet_7.zip › HR-04/103_0.tiff]

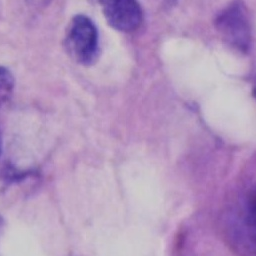

Supplement: Supplementary file 9 [file Data_Sheet_7.zip › HR-04/103_1.tiff]

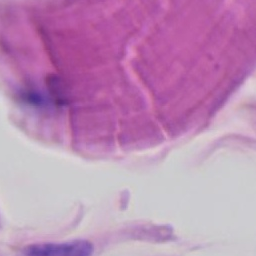

Supplement: Supplementary file 9 [file Data_Sheet_7.zip › HR-04/103_2.tiff]

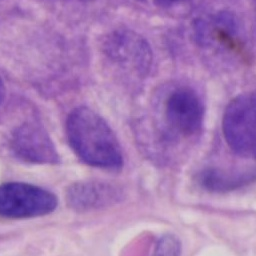

Supplement: Supplementary file 9 [file Data_Sheet_7.zip › HR-04/103_3.tiff]

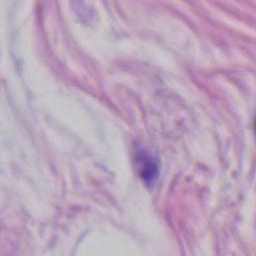

Supplement: Supplementary file 9 [file Data_Sheet_7.zip › HR-04/103_4.tiff]

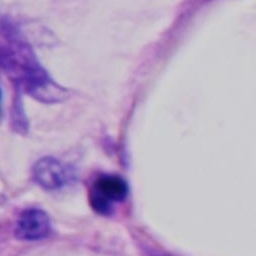

Supplement: Supplementary file 9 [file Data_Sheet_7.zip › HR-04/103_5.tiff]

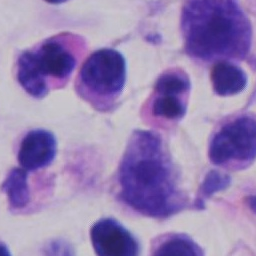

Supplement: Supplementary file 9 [file Data_Sheet_7.zip › HR-04/103_6.tiff]

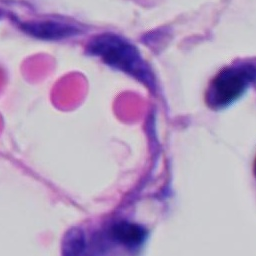

Supplement: Supplementary file 9 [file Data_Sheet_7.zip › HR-04/103_7.tiff]
