# Supplementary figures and images for: Feasibility study of single-image super-resolution scanning system based on deep learning for pathological diagnosis of oral epithelial dysplasia (part 7 of 21)
Source: Front Med (Lausanne). 2025 Mar 12;12:1550512. doi: 10.3389/fmed.2025.1550512 (PMC11936936; doi:10.3389/fmed.2025.1550512)

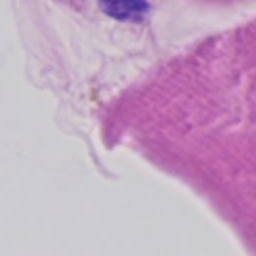

Supplement: Supplementary file 9 [file Data_Sheet_7.zip › HR-04/104_0.tiff]

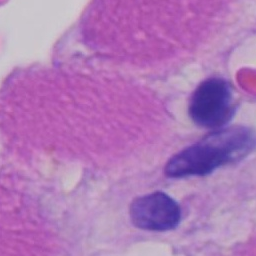

Supplement: Supplementary file 9 [file Data_Sheet_7.zip › HR-04/104_1.tiff]

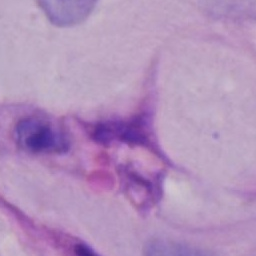

Supplement: Supplementary file 9 [file Data_Sheet_7.zip › HR-04/104_2.tiff]

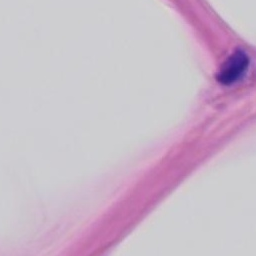

Supplement: Supplementary file 9 [file Data_Sheet_7.zip › HR-04/104_3.tiff]

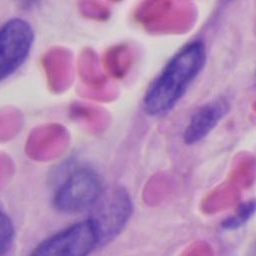

Supplement: Supplementary file 9 [file Data_Sheet_7.zip › HR-04/104_4.tiff]

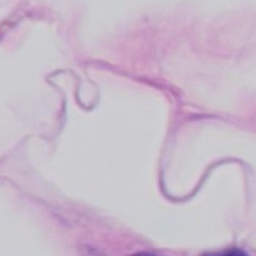

Supplement: Supplementary file 9 [file Data_Sheet_7.zip › HR-04/104_5.tiff]

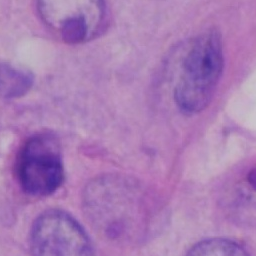

Supplement: Supplementary file 9 [file Data_Sheet_7.zip › HR-04/104_6.tiff]

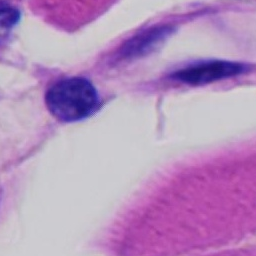

Supplement: Supplementary file 9 [file Data_Sheet_7.zip › HR-04/104_7.tiff]

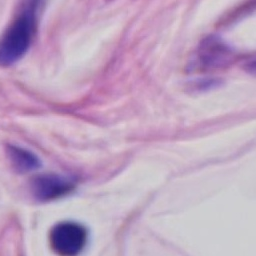

Supplement: Supplementary file 9 [file Data_Sheet_7.zip › HR-04/105_0.tiff]

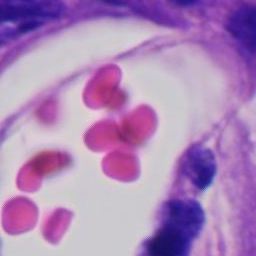

Supplement: Supplementary file 9 [file Data_Sheet_7.zip › HR-04/105_1.tiff]

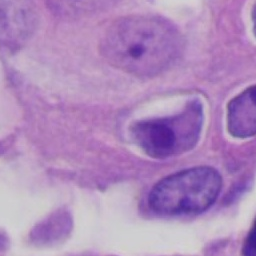

Supplement: Supplementary file 9 [file Data_Sheet_7.zip › HR-04/105_2.tiff]

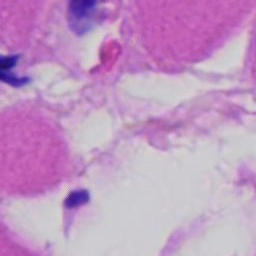

Supplement: Supplementary file 9 [file Data_Sheet_7.zip › HR-04/105_3.tiff]

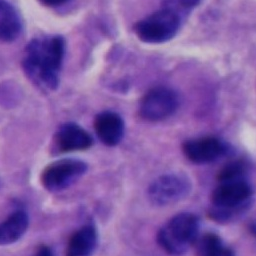

Supplement: Supplementary file 9 [file Data_Sheet_7.zip › HR-04/105_4.tiff]

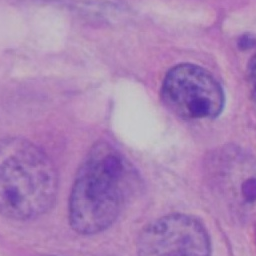

Supplement: Supplementary file 9 [file Data_Sheet_7.zip › HR-04/105_5.tiff]

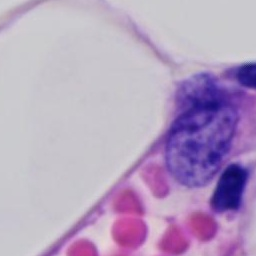

Supplement: Supplementary file 9 [file Data_Sheet_7.zip › HR-04/105_6.tiff]

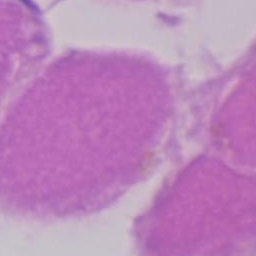

Supplement: Supplementary file 9 [file Data_Sheet_7.zip › HR-04/105_7.tiff]

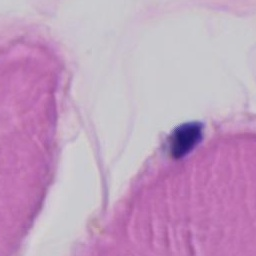

Supplement: Supplementary file 9 [file Data_Sheet_7.zip › HR-04/106_0.tiff]

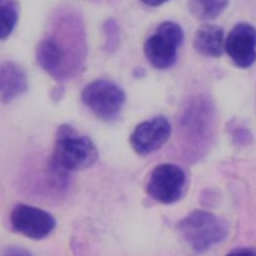

Supplement: Supplementary file 9 [file Data_Sheet_7.zip › HR-04/106_1.tiff]

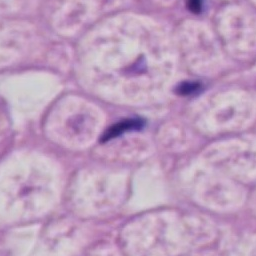

Supplement: Supplementary file 9 [file Data_Sheet_7.zip › HR-04/106_2.tiff]

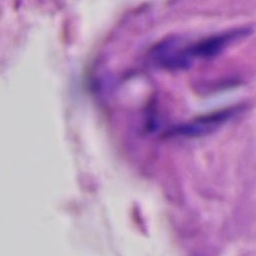

Supplement: Supplementary file 9 [file Data_Sheet_7.zip › HR-04/106_3.tiff]

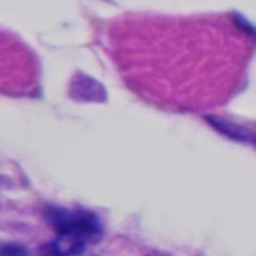

Supplement: Supplementary file 9 [file Data_Sheet_7.zip › HR-04/106_4.tiff]

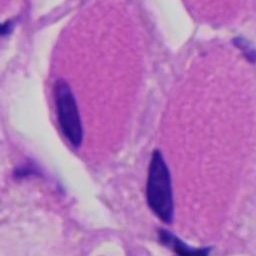

Supplement: Supplementary file 9 [file Data_Sheet_7.zip › HR-04/106_5.tiff]

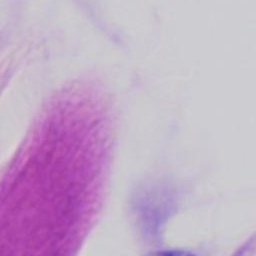

Supplement: Supplementary file 9 [file Data_Sheet_7.zip › HR-04/106_6.tiff]

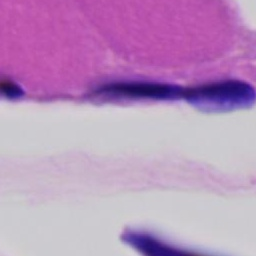

Supplement: Supplementary file 9 [file Data_Sheet_7.zip › HR-04/106_7.tiff]

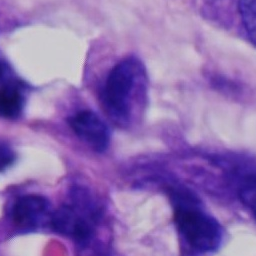

Supplement: Supplementary file 9 [file Data_Sheet_7.zip › HR-04/107_0.tiff]

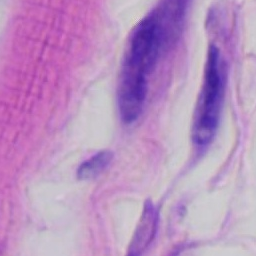

Supplement: Supplementary file 9 [file Data_Sheet_7.zip › HR-04/107_1.tiff]

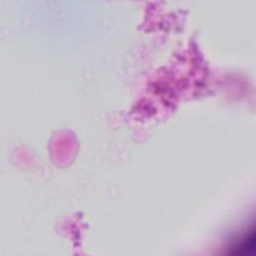

Supplement: Supplementary file 9 [file Data_Sheet_7.zip › HR-04/107_2.tiff]

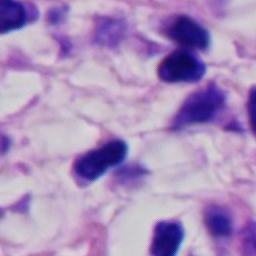

Supplement: Supplementary file 9 [file Data_Sheet_7.zip › HR-04/107_3.tiff]

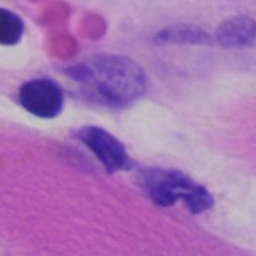

Supplement: Supplementary file 9 [file Data_Sheet_7.zip › HR-04/107_4.tiff]

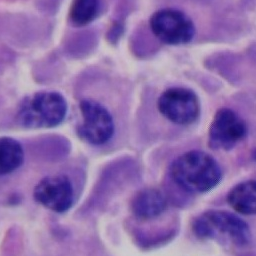

Supplement: Supplementary file 9 [file Data_Sheet_7.zip › HR-04/107_5.tiff]

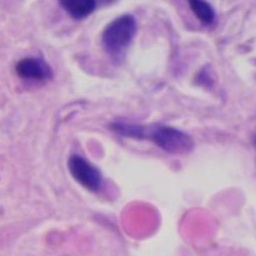

Supplement: Supplementary file 9 [file Data_Sheet_7.zip › HR-04/107_6.tiff]

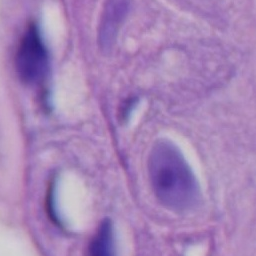

Supplement: Supplementary file 9 [file Data_Sheet_7.zip › HR-04/107_7.tiff]

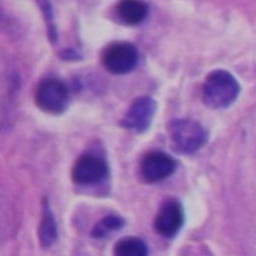

Supplement: Supplementary file 9 [file Data_Sheet_7.zip › HR-04/108_0.tiff]

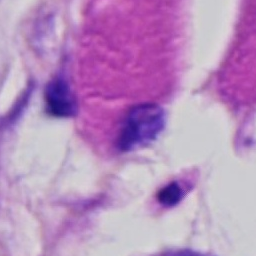

Supplement: Supplementary file 9 [file Data_Sheet_7.zip › HR-04/108_1.tiff]

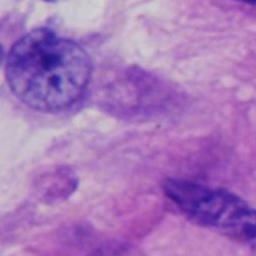

Supplement: Supplementary file 9 [file Data_Sheet_7.zip › HR-04/108_2.tiff]

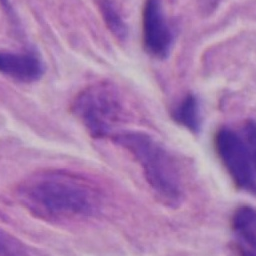

Supplement: Supplementary file 9 [file Data_Sheet_7.zip › HR-04/108_3.tiff]

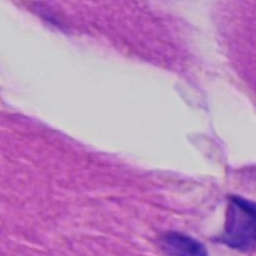

Supplement: Supplementary file 9 [file Data_Sheet_7.zip › HR-04/108_4.tiff]

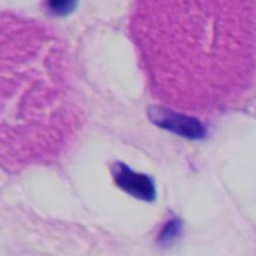

Supplement: Supplementary file 9 [file Data_Sheet_7.zip › HR-04/108_5.tiff]

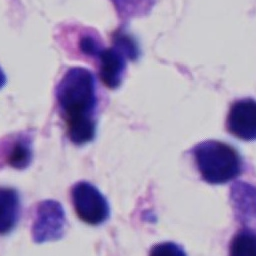

Supplement: Supplementary file 9 [file Data_Sheet_7.zip › HR-04/108_6.tiff]

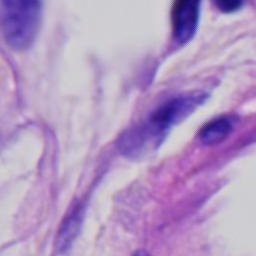

Supplement: Supplementary file 9 [file Data_Sheet_7.zip › HR-04/108_7.tiff]

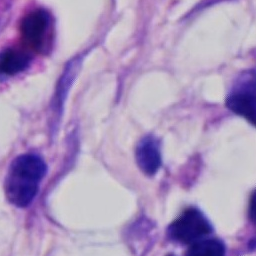

Supplement: Supplementary file 9 [file Data_Sheet_7.zip › HR-04/109_0.tiff]

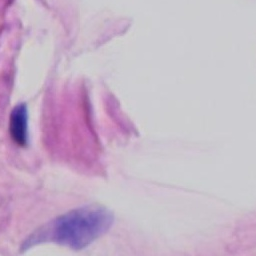

Supplement: Supplementary file 9 [file Data_Sheet_7.zip › HR-04/109_1.tiff]

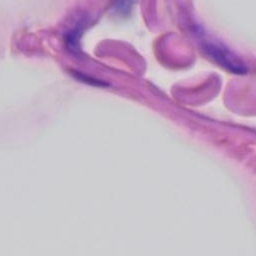

Supplement: Supplementary file 9 [file Data_Sheet_7.zip › HR-04/109_2.tiff]

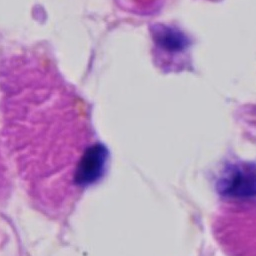

Supplement: Supplementary file 9 [file Data_Sheet_7.zip › HR-04/109_3.tiff]

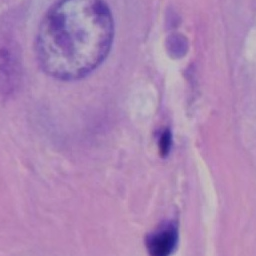

Supplement: Supplementary file 9 [file Data_Sheet_7.zip › HR-04/109_4.tiff]

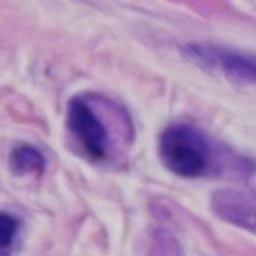

Supplement: Supplementary file 9 [file Data_Sheet_7.zip › HR-04/109_5.tiff]

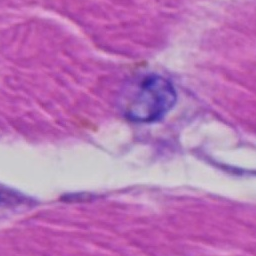

Supplement: Supplementary file 9 [file Data_Sheet_7.zip › HR-04/109_6.tiff]

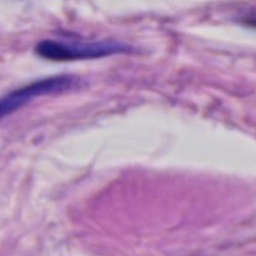

Supplement: Supplementary file 9 [file Data_Sheet_7.zip › HR-04/109_7.tiff]

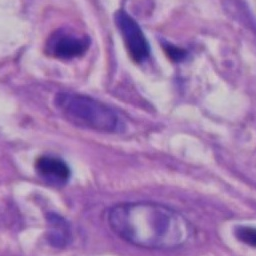

Supplement: Supplementary file 9 [file Data_Sheet_7.zip › HR-04/110_0.tiff]

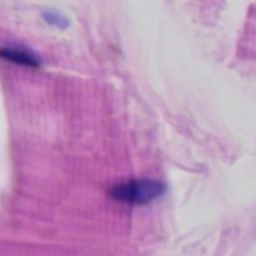

Supplement: Supplementary file 9 [file Data_Sheet_7.zip › HR-04/110_1.tiff]

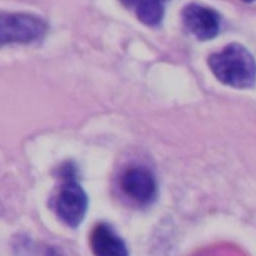

Supplement: Supplementary file 9 [file Data_Sheet_7.zip › HR-04/110_2.tiff]

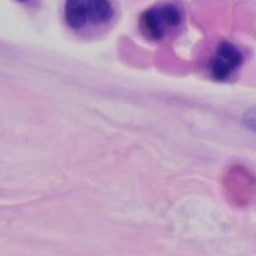

Supplement: Supplementary file 9 [file Data_Sheet_7.zip › HR-04/110_3.tiff]

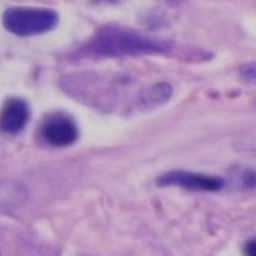

Supplement: Supplementary file 9 [file Data_Sheet_7.zip › HR-04/110_4.tiff]

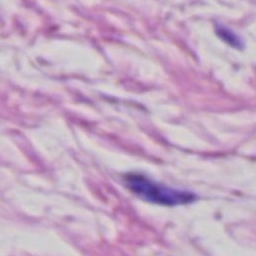

Supplement: Supplementary file 9 [file Data_Sheet_7.zip › HR-04/110_5.tiff]

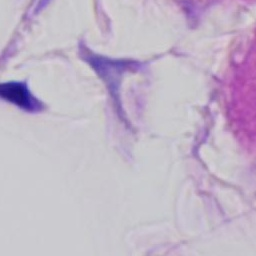

Supplement: Supplementary file 9 [file Data_Sheet_7.zip › HR-04/110_6.tiff]

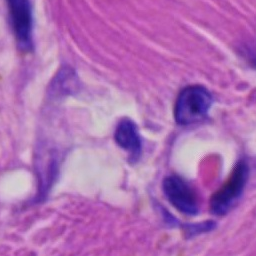

Supplement: Supplementary file 9 [file Data_Sheet_7.zip › HR-04/110_7.tiff]

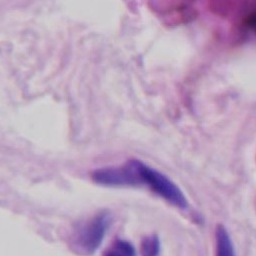

Supplement: Supplementary file 9 [file Data_Sheet_7.zip › HR-04/111_0.tiff]

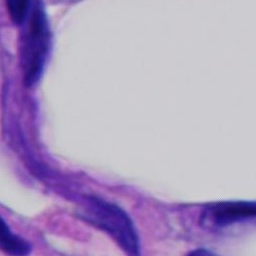

Supplement: Supplementary file 9 [file Data_Sheet_7.zip › HR-04/111_1.tiff]

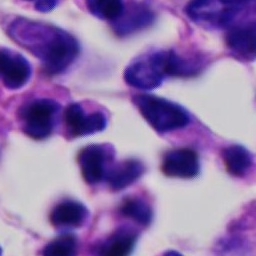

Supplement: Supplementary file 9 [file Data_Sheet_7.zip › HR-04/111_2.tiff]

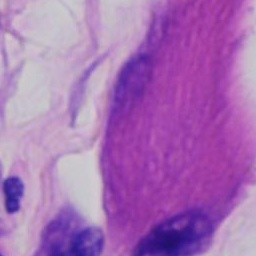

Supplement: Supplementary file 9 [file Data_Sheet_7.zip › HR-04/111_3.tiff]

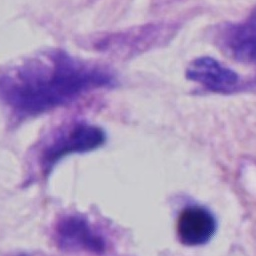

Supplement: Supplementary file 9 [file Data_Sheet_7.zip › HR-04/111_4.tiff]

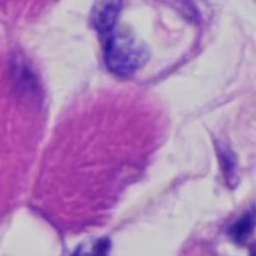

Supplement: Supplementary file 9 [file Data_Sheet_7.zip › HR-04/111_5.tiff]

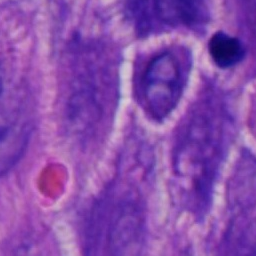

Supplement: Supplementary file 9 [file Data_Sheet_7.zip › HR-04/81_0.tiff]

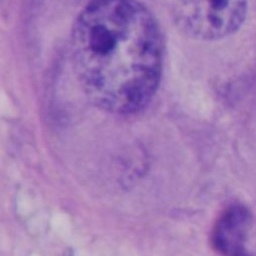

Supplement: Supplementary file 9 [file Data_Sheet_7.zip › HR-04/81_1.tiff]

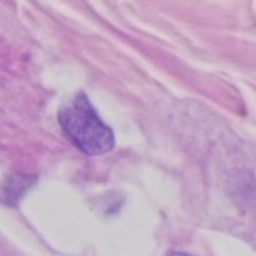

Supplement: Supplementary file 9 [file Data_Sheet_7.zip › HR-04/81_2.tiff]

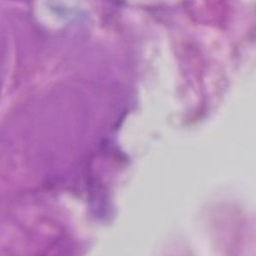

Supplement: Supplementary file 9 [file Data_Sheet_7.zip › HR-04/81_3.tiff]

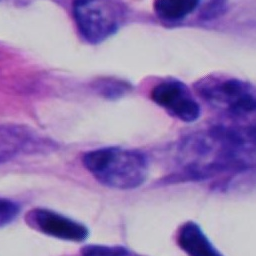

Supplement: Supplementary file 9 [file Data_Sheet_7.zip › HR-04/81_4.tiff]

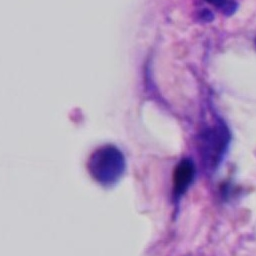

Supplement: Supplementary file 9 [file Data_Sheet_7.zip › HR-04/81_5.tiff]

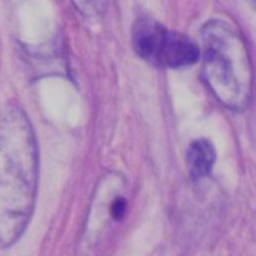

Supplement: Supplementary file 9 [file Data_Sheet_7.zip › HR-04/81_6.tiff]

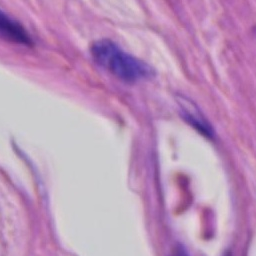

Supplement: Supplementary file 9 [file Data_Sheet_7.zip › HR-04/81_7.tiff]

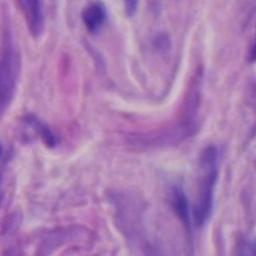

Supplement: Supplementary file 9 [file Data_Sheet_7.zip › HR-04/82_0.tiff]

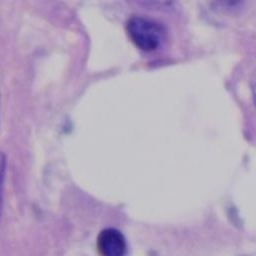

Supplement: Supplementary file 9 [file Data_Sheet_7.zip › HR-04/82_1.tiff]

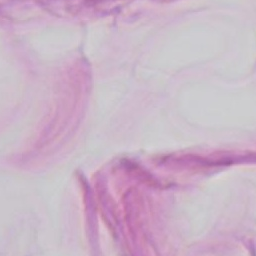

Supplement: Supplementary file 9 [file Data_Sheet_7.zip › HR-04/82_2.tiff]

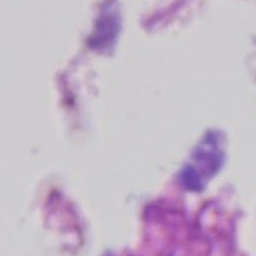

Supplement: Supplementary file 9 [file Data_Sheet_7.zip › HR-04/82_3.tiff]

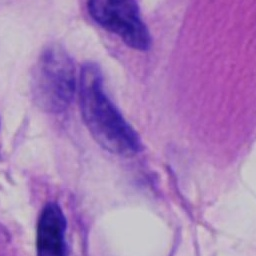

Supplement: Supplementary file 9 [file Data_Sheet_7.zip › HR-04/82_4.tiff]

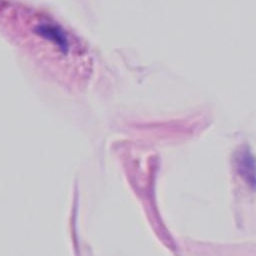

Supplement: Supplementary file 9 [file Data_Sheet_7.zip › HR-04/82_5.tiff]

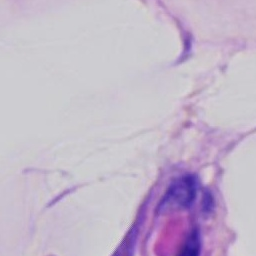

Supplement: Supplementary file 9 [file Data_Sheet_7.zip › HR-04/82_6.tiff]

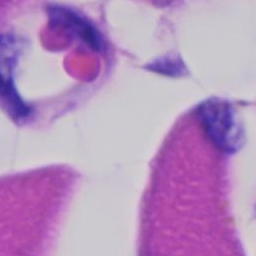

Supplement: Supplementary file 9 [file Data_Sheet_7.zip › HR-04/82_7.tiff]

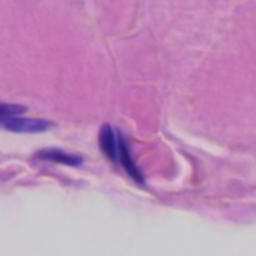

Supplement: Supplementary file 9 [file Data_Sheet_7.zip › HR-04/83_0.tiff]

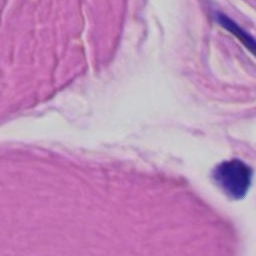

Supplement: Supplementary file 9 [file Data_Sheet_7.zip › HR-04/83_1.tiff]

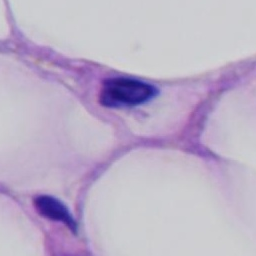

Supplement: Supplementary file 9 [file Data_Sheet_7.zip › HR-04/83_2.tiff]

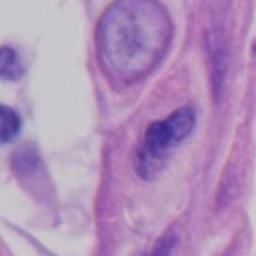

Supplement: Supplementary file 9 [file Data_Sheet_7.zip › HR-04/83_3.tiff]

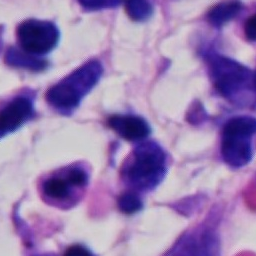

Supplement: Supplementary file 9 [file Data_Sheet_7.zip › HR-04/83_4.tiff]

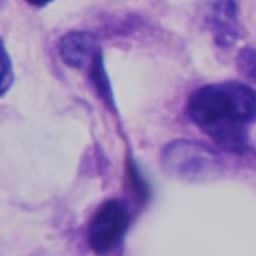

Supplement: Supplementary file 9 [file Data_Sheet_7.zip › HR-04/83_5.tiff]

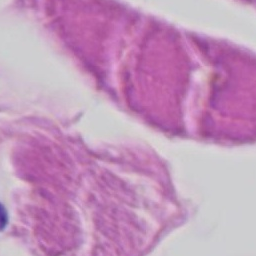

Supplement: Supplementary file 9 [file Data_Sheet_7.zip › HR-04/83_6.tiff]

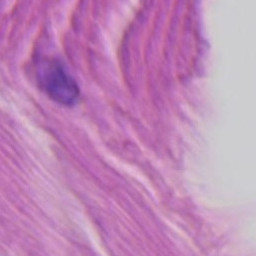

Supplement: Supplementary file 9 [file Data_Sheet_7.zip › HR-04/83_7.tiff]

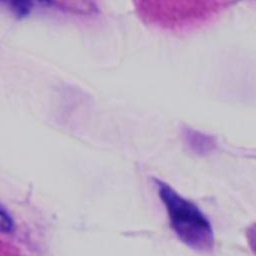

Supplement: Supplementary file 9 [file Data_Sheet_7.zip › HR-04/84_0.tiff]

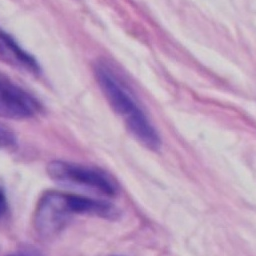

Supplement: Supplementary file 9 [file Data_Sheet_7.zip › HR-04/84_1.tiff]

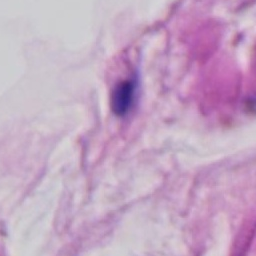

Supplement: Supplementary file 9 [file Data_Sheet_7.zip › HR-04/84_2.tiff]

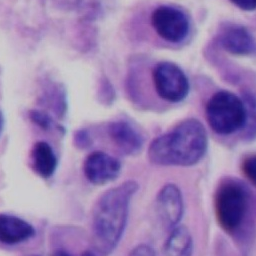

Supplement: Supplementary file 9 [file Data_Sheet_7.zip › HR-04/84_3.tiff]

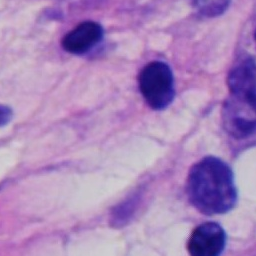

Supplement: Supplementary file 9 [file Data_Sheet_7.zip › HR-04/84_4.tiff]

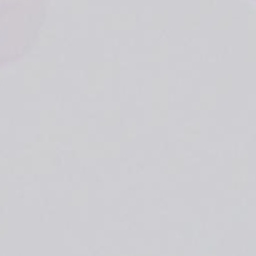

Supplement: Supplementary file 9 [file Data_Sheet_7.zip › HR-04/84_5.tiff]

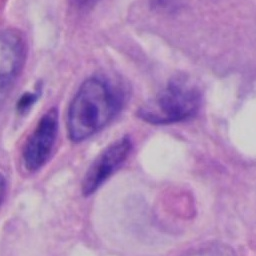

Supplement: Supplementary file 9 [file Data_Sheet_7.zip › HR-04/84_6.tiff]

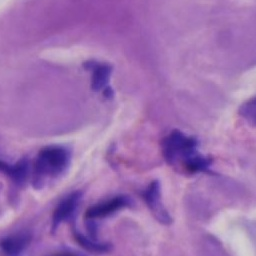

Supplement: Supplementary file 9 [file Data_Sheet_7.zip › HR-04/84_7.tiff]

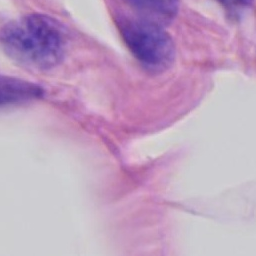

Supplement: Supplementary file 9 [file Data_Sheet_7.zip › HR-04/85_0.tiff]

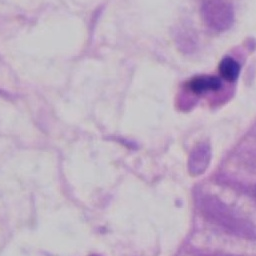

Supplement: Supplementary file 9 [file Data_Sheet_7.zip › HR-04/85_1.tiff]

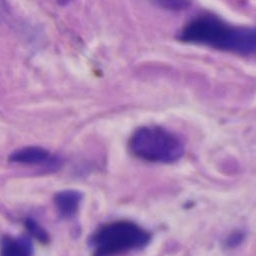

Supplement: Supplementary file 9 [file Data_Sheet_7.zip › HR-04/85_2.tiff]

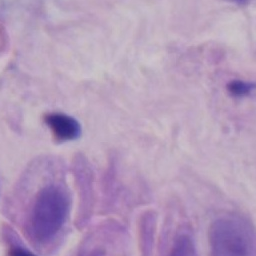

Supplement: Supplementary file 9 [file Data_Sheet_7.zip › HR-04/85_3.tiff]

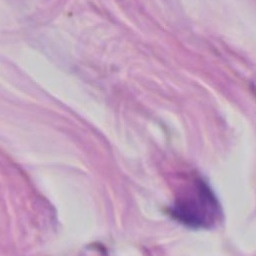

Supplement: Supplementary file 9 [file Data_Sheet_7.zip › HR-04/85_4.tiff]

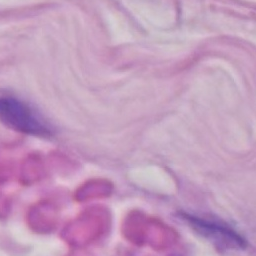

Supplement: Supplementary file 9 [file Data_Sheet_7.zip › HR-04/85_5.tiff]
