# Supplementary figures and images for: Feasibility study of single-image super-resolution scanning system based on deep learning for pathological diagnosis of oral epithelial dysplasia (part 10 of 21)
Source: Front Med (Lausanne). 2025 Mar 12;12:1550512. doi: 10.3389/fmed.2025.1550512 (PMC11936936; doi:10.3389/fmed.2025.1550512)

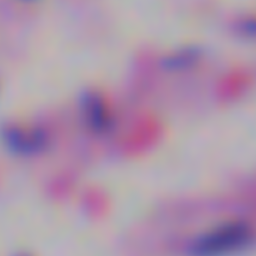

Supplement: Supplementary file 10 [file Data_Sheet_8.zip › LR-01/5_4.tiff]

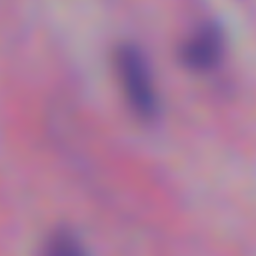

Supplement: Supplementary file 10 [file Data_Sheet_8.zip › LR-01/5_5.tiff]

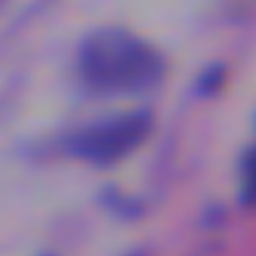

Supplement: Supplementary file 10 [file Data_Sheet_8.zip › LR-01/5_6.tiff]

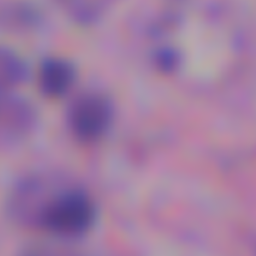

Supplement: Supplementary file 10 [file Data_Sheet_8.zip › LR-01/5_7.tiff]

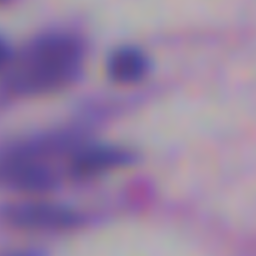

Supplement: Supplementary file 10 [file Data_Sheet_8.zip › LR-01/6_0.tiff]

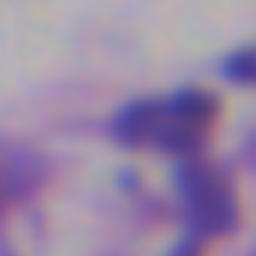

Supplement: Supplementary file 10 [file Data_Sheet_8.zip › LR-01/6_1.tiff]

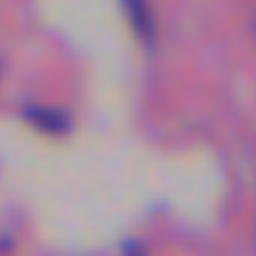

Supplement: Supplementary file 10 [file Data_Sheet_8.zip › LR-01/6_2.tiff]

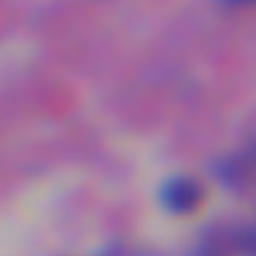

Supplement: Supplementary file 10 [file Data_Sheet_8.zip › LR-01/6_3.tiff]

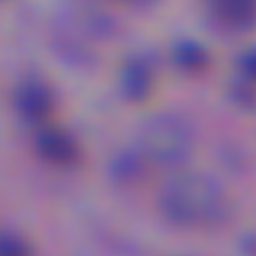

Supplement: Supplementary file 10 [file Data_Sheet_8.zip › LR-01/6_4.tiff]

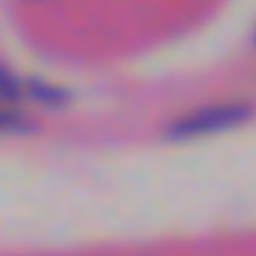

Supplement: Supplementary file 10 [file Data_Sheet_8.zip › LR-01/6_5.tiff]

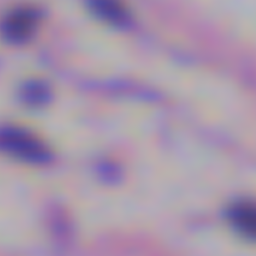

Supplement: Supplementary file 10 [file Data_Sheet_8.zip › LR-01/6_6.tiff]

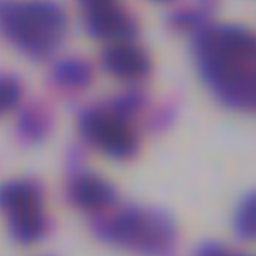

Supplement: Supplementary file 10 [file Data_Sheet_8.zip › LR-01/6_7.tiff]

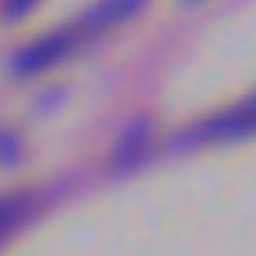

Supplement: Supplementary file 10 [file Data_Sheet_8.zip › LR-01/7_0.tiff]

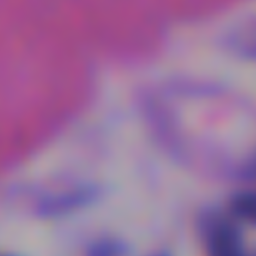

Supplement: Supplementary file 10 [file Data_Sheet_8.zip › LR-01/7_1.tiff]

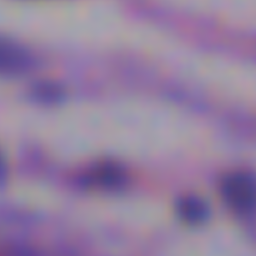

Supplement: Supplementary file 10 [file Data_Sheet_8.zip › LR-01/7_2.tiff]

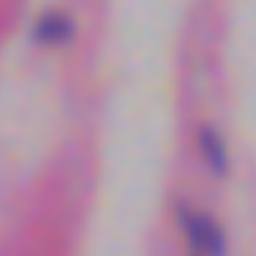

Supplement: Supplementary file 10 [file Data_Sheet_8.zip › LR-01/7_3.tiff]

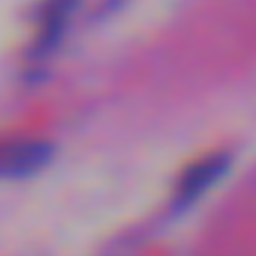

Supplement: Supplementary file 10 [file Data_Sheet_8.zip › LR-01/7_4.tiff]

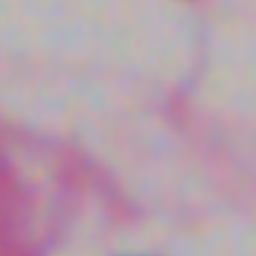

Supplement: Supplementary file 10 [file Data_Sheet_8.zip › LR-01/7_5.tiff]

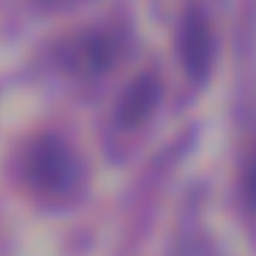

Supplement: Supplementary file 10 [file Data_Sheet_8.zip › LR-01/7_6.tiff]

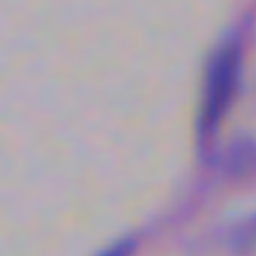

Supplement: Supplementary file 10 [file Data_Sheet_8.zip › LR-01/7_7.tiff]

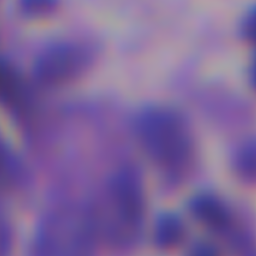

Supplement: Supplementary file 10 [file Data_Sheet_8.zip › LR-01/8_0.tiff]

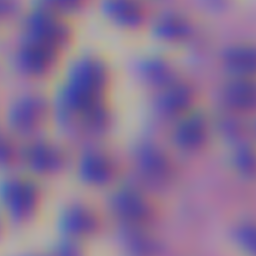

Supplement: Supplementary file 10 [file Data_Sheet_8.zip › LR-01/8_1.tiff]

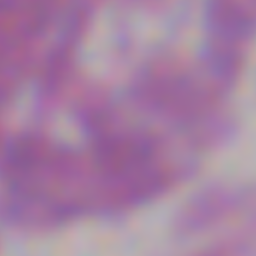

Supplement: Supplementary file 10 [file Data_Sheet_8.zip › LR-01/8_2.tiff]

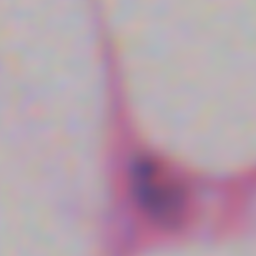

Supplement: Supplementary file 10 [file Data_Sheet_8.zip › LR-01/8_3.tiff]

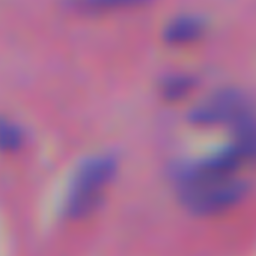

Supplement: Supplementary file 10 [file Data_Sheet_8.zip › LR-01/8_4.tiff]

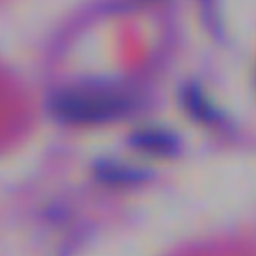

Supplement: Supplementary file 10 [file Data_Sheet_8.zip › LR-01/8_5.tiff]

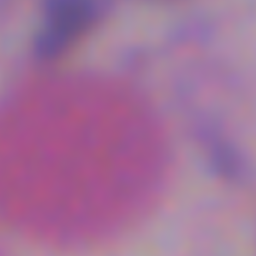

Supplement: Supplementary file 10 [file Data_Sheet_8.zip › LR-01/8_6.tiff]

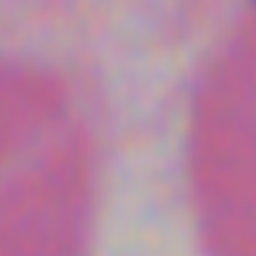

Supplement: Supplementary file 10 [file Data_Sheet_8.zip › LR-01/8_7.tiff]

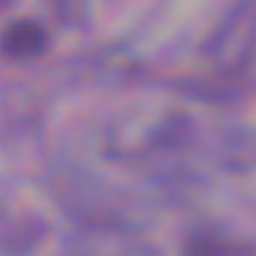

Supplement: Supplementary file 10 [file Data_Sheet_8.zip › LR-01/9_0.tiff]

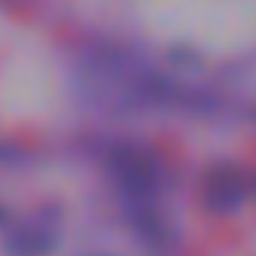

Supplement: Supplementary file 10 [file Data_Sheet_8.zip › LR-01/9_1.tiff]

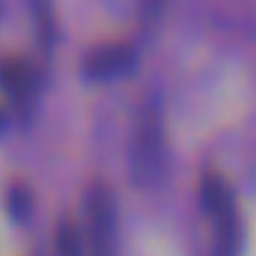

Supplement: Supplementary file 10 [file Data_Sheet_8.zip › LR-01/9_2.tiff]

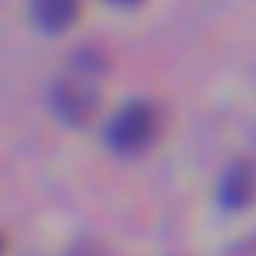

Supplement: Supplementary file 10 [file Data_Sheet_8.zip › LR-01/9_3.tiff]

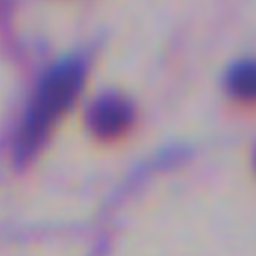

Supplement: Supplementary file 10 [file Data_Sheet_8.zip › LR-01/9_4.tiff]

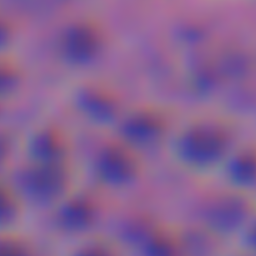

Supplement: Supplementary file 10 [file Data_Sheet_8.zip › LR-01/9_5.tiff]

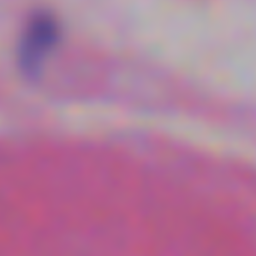

Supplement: Supplementary file 10 [file Data_Sheet_8.zip › LR-01/9_6.tiff]

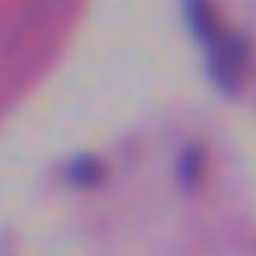

Supplement: Supplementary file 10 [file Data_Sheet_8.zip › LR-01/9_7.tiff]

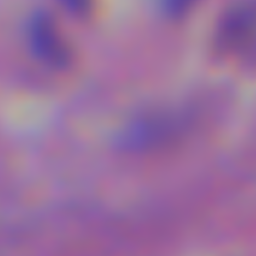

Supplement: Supplementary file 11 [file Data_Sheet_9.zip › LR-02/29_6.tiff]

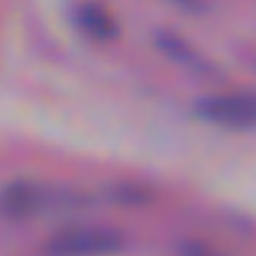

Supplement: Supplementary file 11 [file Data_Sheet_9.zip › LR-02/29_7.tiff]

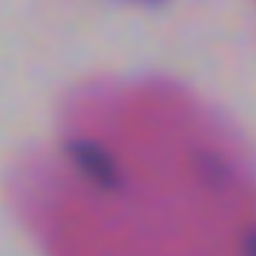

Supplement: Supplementary file 11 [file Data_Sheet_9.zip › LR-02/30_0.tiff]

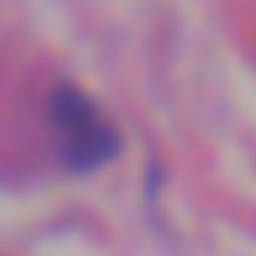

Supplement: Supplementary file 11 [file Data_Sheet_9.zip › LR-02/30_1.tiff]

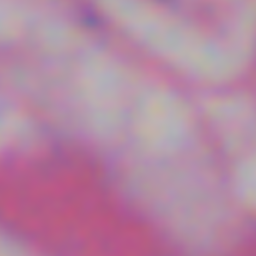

Supplement: Supplementary file 11 [file Data_Sheet_9.zip › LR-02/30_2.tiff]

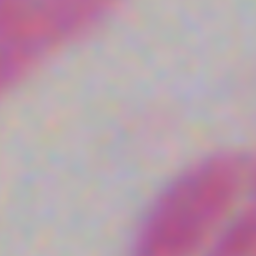

Supplement: Supplementary file 11 [file Data_Sheet_9.zip › LR-02/30_3.tiff]

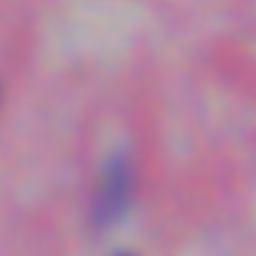

Supplement: Supplementary file 11 [file Data_Sheet_9.zip › LR-02/30_4.tiff]

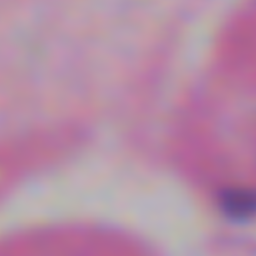

Supplement: Supplementary file 11 [file Data_Sheet_9.zip › LR-02/30_5.tiff]

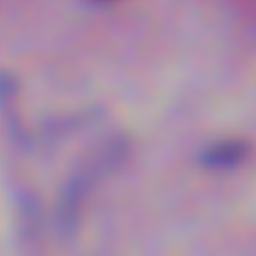

Supplement: Supplementary file 11 [file Data_Sheet_9.zip › LR-02/30_6.tiff]

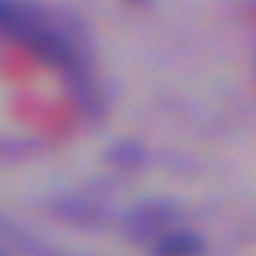

Supplement: Supplementary file 11 [file Data_Sheet_9.zip › LR-02/30_7.tiff]

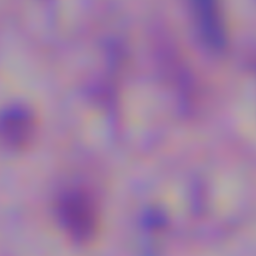

Supplement: Supplementary file 11 [file Data_Sheet_9.zip › LR-02/31_0.tiff]

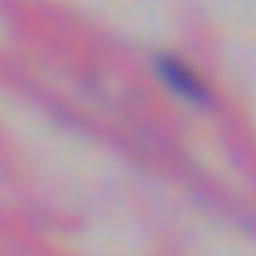

Supplement: Supplementary file 11 [file Data_Sheet_9.zip › LR-02/31_1.tiff]

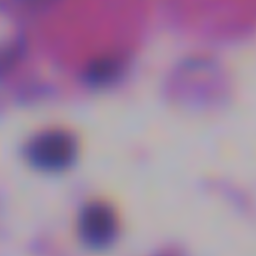

Supplement: Supplementary file 11 [file Data_Sheet_9.zip › LR-02/31_2.tiff]

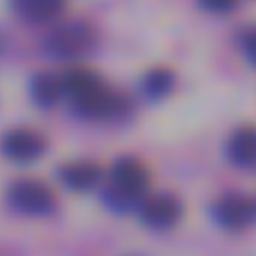

Supplement: Supplementary file 11 [file Data_Sheet_9.zip › LR-02/31_3.tiff]

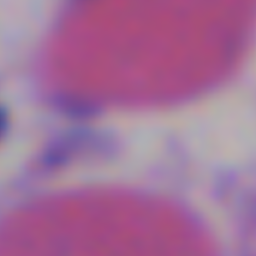

Supplement: Supplementary file 11 [file Data_Sheet_9.zip › LR-02/31_4.tiff]

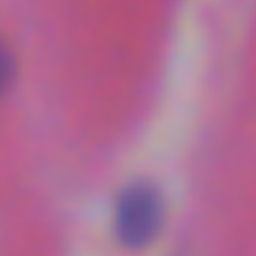

Supplement: Supplementary file 11 [file Data_Sheet_9.zip › LR-02/31_5.tiff]

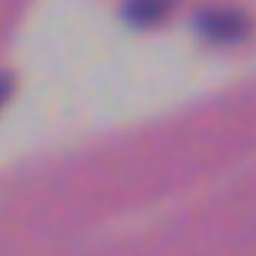

Supplement: Supplementary file 11 [file Data_Sheet_9.zip › LR-02/31_6.tiff]

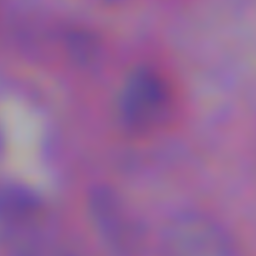

Supplement: Supplementary file 11 [file Data_Sheet_9.zip › LR-02/31_7.tiff]

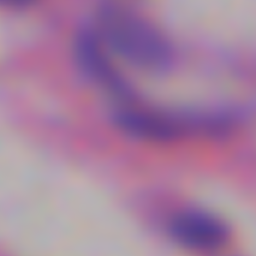

Supplement: Supplementary file 11 [file Data_Sheet_9.zip › LR-02/32_0.tiff]

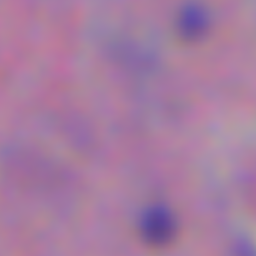

Supplement: Supplementary file 11 [file Data_Sheet_9.zip › LR-02/32_1.tiff]

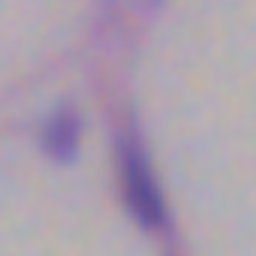

Supplement: Supplementary file 11 [file Data_Sheet_9.zip › LR-02/32_2.tiff]

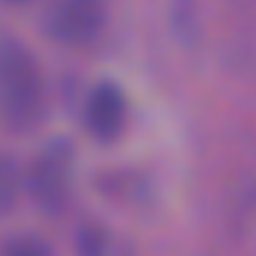

Supplement: Supplementary file 11 [file Data_Sheet_9.zip › LR-02/32_3.tiff]

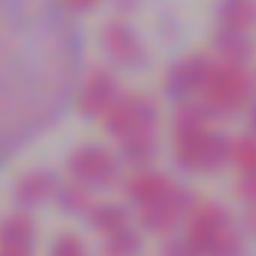

Supplement: Supplementary file 11 [file Data_Sheet_9.zip › LR-02/32_4.tiff]

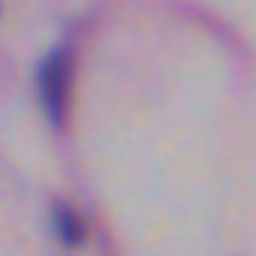

Supplement: Supplementary file 11 [file Data_Sheet_9.zip › LR-02/32_5.tiff]

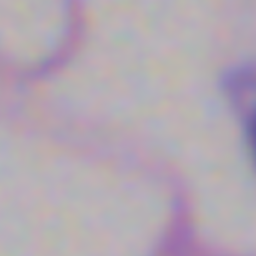

Supplement: Supplementary file 11 [file Data_Sheet_9.zip › LR-02/32_6.tiff]

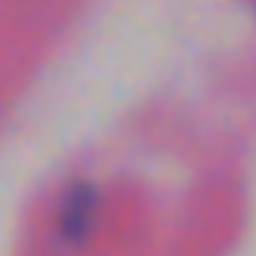

Supplement: Supplementary file 11 [file Data_Sheet_9.zip › LR-02/32_7.tiff]

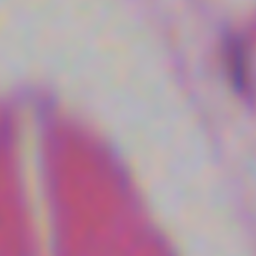

Supplement: Supplementary file 11 [file Data_Sheet_9.zip › LR-02/33_0.tiff]

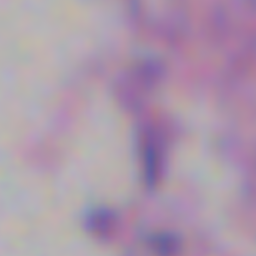

Supplement: Supplementary file 11 [file Data_Sheet_9.zip › LR-02/33_1.tiff]

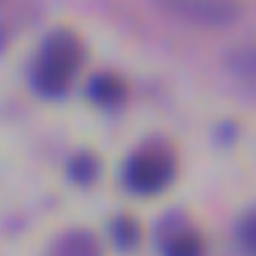

Supplement: Supplementary file 11 [file Data_Sheet_9.zip › LR-02/33_2.tiff]

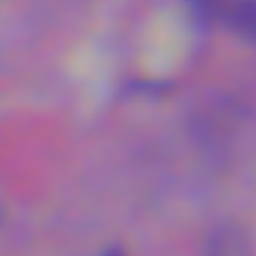

Supplement: Supplementary file 11 [file Data_Sheet_9.zip › LR-02/33_3.tiff]

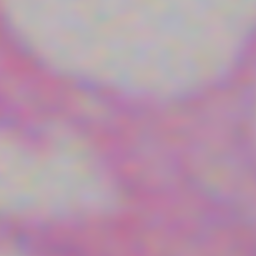

Supplement: Supplementary file 11 [file Data_Sheet_9.zip › LR-02/33_4.tiff]

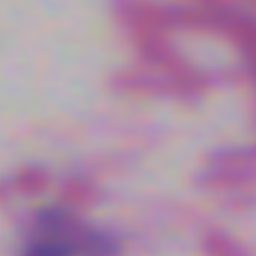

Supplement: Supplementary file 11 [file Data_Sheet_9.zip › LR-02/33_5.tiff]

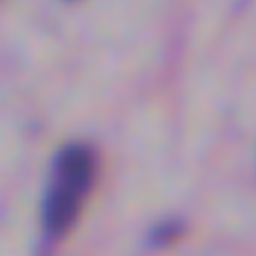

Supplement: Supplementary file 11 [file Data_Sheet_9.zip › LR-02/33_6.tiff]

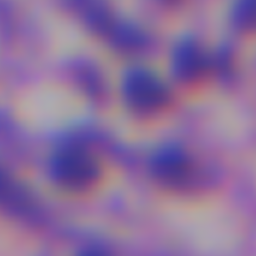

Supplement: Supplementary file 11 [file Data_Sheet_9.zip › LR-02/33_7.tiff]

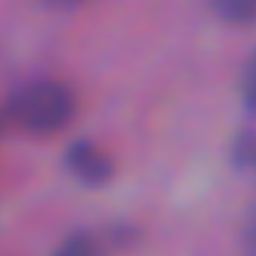

Supplement: Supplementary file 11 [file Data_Sheet_9.zip › LR-02/34_0.tiff]

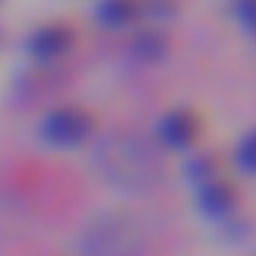

Supplement: Supplementary file 11 [file Data_Sheet_9.zip › LR-02/34_1.tiff]

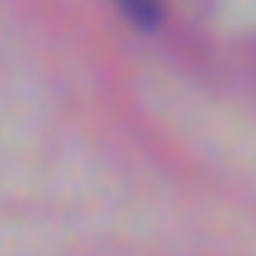

Supplement: Supplementary file 11 [file Data_Sheet_9.zip › LR-02/34_2.tiff]

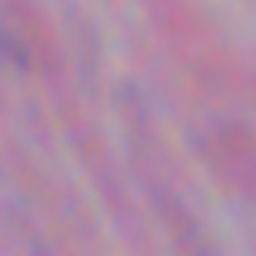

Supplement: Supplementary file 11 [file Data_Sheet_9.zip › LR-02/34_3.tiff]

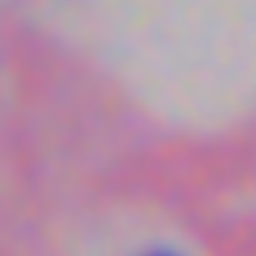

Supplement: Supplementary file 11 [file Data_Sheet_9.zip › LR-02/34_4.tiff]

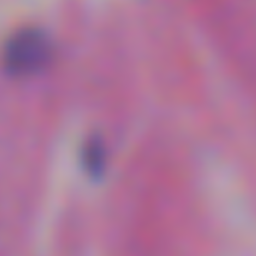

Supplement: Supplementary file 11 [file Data_Sheet_9.zip › LR-02/34_5.tiff]

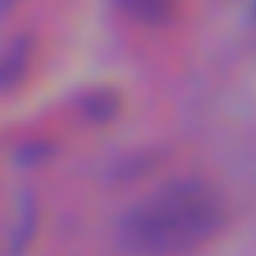

Supplement: Supplementary file 11 [file Data_Sheet_9.zip › LR-02/34_6.tiff]

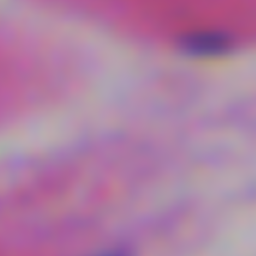

Supplement: Supplementary file 11 [file Data_Sheet_9.zip › LR-02/34_7.tiff]

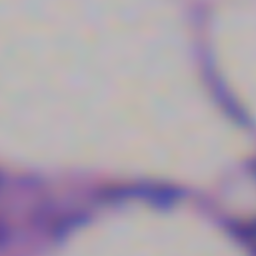

Supplement: Supplementary file 11 [file Data_Sheet_9.zip › LR-02/35_0.tiff]

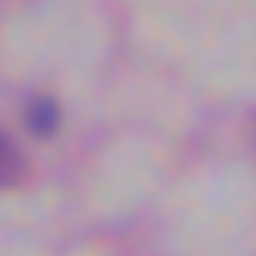

Supplement: Supplementary file 11 [file Data_Sheet_9.zip › LR-02/35_1.tiff]

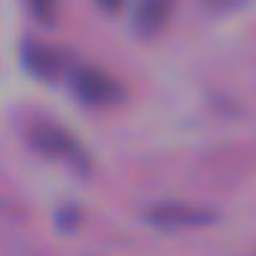

Supplement: Supplementary file 11 [file Data_Sheet_9.zip › LR-02/35_2.tiff]

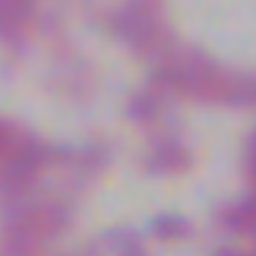

Supplement: Supplementary file 11 [file Data_Sheet_9.zip › LR-02/35_3.tiff]

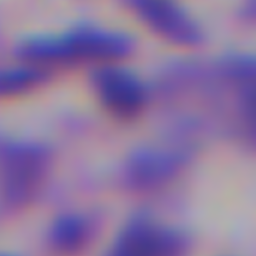

Supplement: Supplementary file 11 [file Data_Sheet_9.zip › LR-02/35_4.tiff]

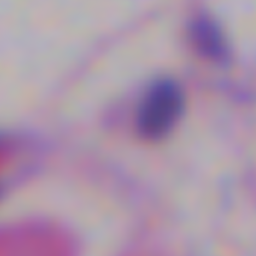

Supplement: Supplementary file 11 [file Data_Sheet_9.zip › LR-02/35_5.tiff]

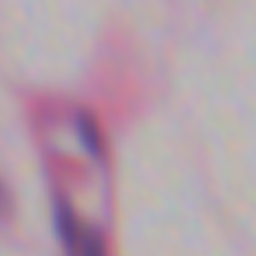

Supplement: Supplementary file 11 [file Data_Sheet_9.zip › LR-02/35_6.tiff]

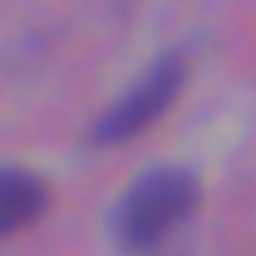

Supplement: Supplementary file 11 [file Data_Sheet_9.zip › LR-02/35_7.tiff]

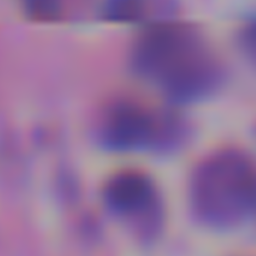

Supplement: Supplementary file 11 [file Data_Sheet_9.zip › LR-02/36_0.tiff]

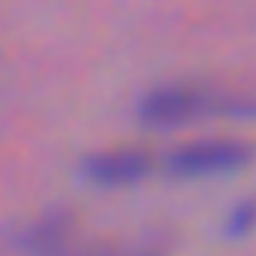

Supplement: Supplementary file 11 [file Data_Sheet_9.zip › LR-02/36_1.tiff]

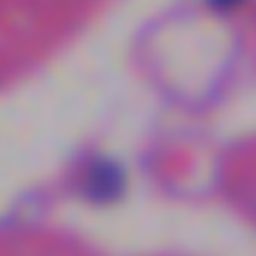

Supplement: Supplementary file 11 [file Data_Sheet_9.zip › LR-02/36_2.tiff]

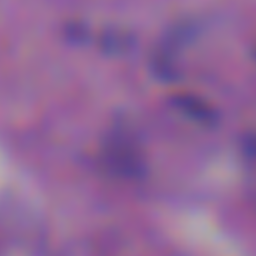

Supplement: Supplementary file 11 [file Data_Sheet_9.zip › LR-02/36_3.tiff]

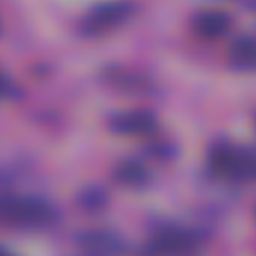

Supplement: Supplementary file 11 [file Data_Sheet_9.zip › LR-02/36_4.tiff]

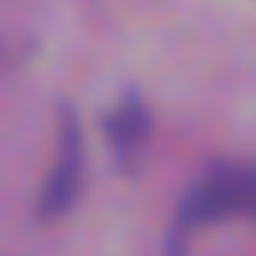

Supplement: Supplementary file 11 [file Data_Sheet_9.zip › LR-02/36_5.tiff]

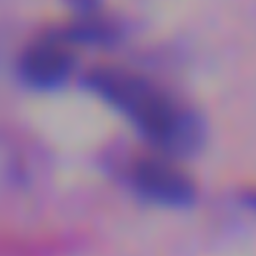

Supplement: Supplementary file 11 [file Data_Sheet_9.zip › LR-02/36_6.tiff]

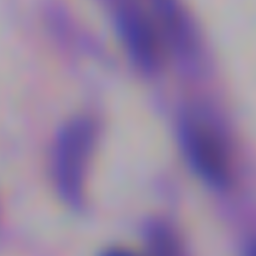

Supplement: Supplementary file 11 [file Data_Sheet_9.zip › LR-02/36_7.tiff]

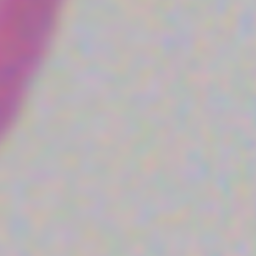

Supplement: Supplementary file 11 [file Data_Sheet_9.zip › LR-02/37_0.tiff]

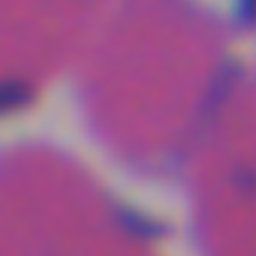

Supplement: Supplementary file 11 [file Data_Sheet_9.zip › LR-02/37_1.tiff]

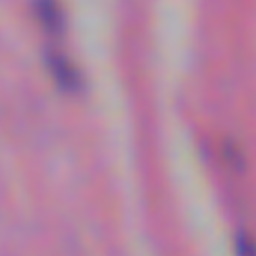

Supplement: Supplementary file 11 [file Data_Sheet_9.zip › LR-02/37_2.tiff]

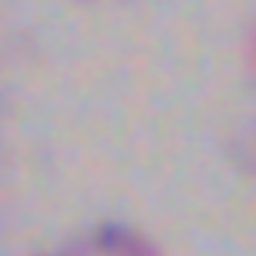

Supplement: Supplementary file 11 [file Data_Sheet_9.zip › LR-02/37_3.tiff]

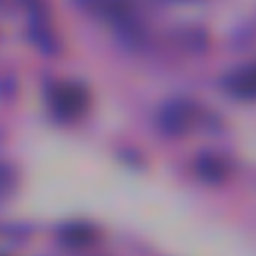

Supplement: Supplementary file 11 [file Data_Sheet_9.zip › LR-02/37_4.tiff]

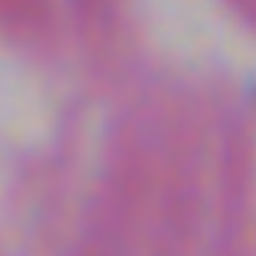

Supplement: Supplementary file 11 [file Data_Sheet_9.zip › LR-02/37_5.tiff]
