# Supplementary figures and images for: Feasibility study of single-image super-resolution scanning system based on deep learning for pathological diagnosis of oral epithelial dysplasia (part 12 of 21)
Source: Front Med (Lausanne). 2025 Mar 12;12:1550512. doi: 10.3389/fmed.2025.1550512 (PMC11936936; doi:10.3389/fmed.2025.1550512)

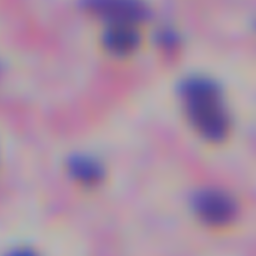

Supplement: Supplementary file 11 [file Data_Sheet_9.zip › LR-02/50_2.tiff]

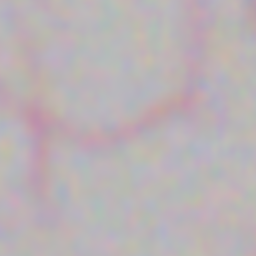

Supplement: Supplementary file 11 [file Data_Sheet_9.zip › LR-02/50_3.tiff]

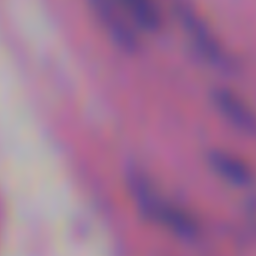

Supplement: Supplementary file 11 [file Data_Sheet_9.zip › LR-02/50_4.tiff]

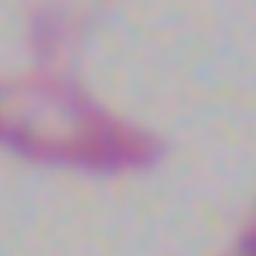

Supplement: Supplementary file 11 [file Data_Sheet_9.zip › LR-02/50_5.tiff]

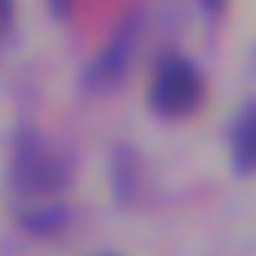

Supplement: Supplementary file 11 [file Data_Sheet_9.zip › LR-02/50_6.tiff]

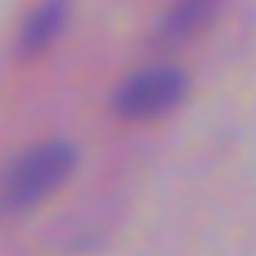

Supplement: Supplementary file 11 [file Data_Sheet_9.zip › LR-02/50_7.tiff]

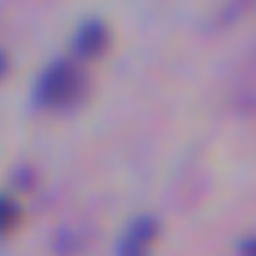

Supplement: Supplementary file 11 [file Data_Sheet_9.zip › LR-02/51_0.tiff]

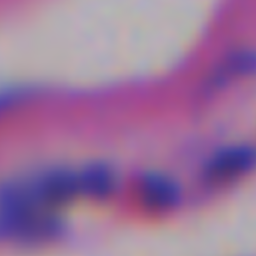

Supplement: Supplementary file 11 [file Data_Sheet_9.zip › LR-02/51_1.tiff]

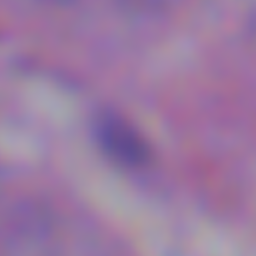

Supplement: Supplementary file 11 [file Data_Sheet_9.zip › LR-02/51_2.tiff]

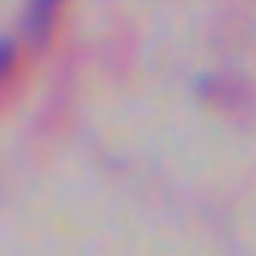

Supplement: Supplementary file 11 [file Data_Sheet_9.zip › LR-02/51_3.tiff]

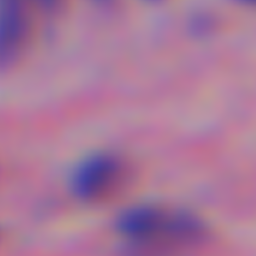

Supplement: Supplementary file 11 [file Data_Sheet_9.zip › LR-02/51_4.tiff]

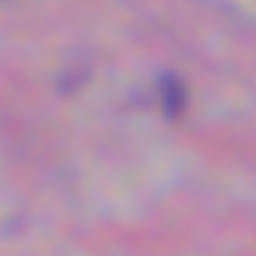

Supplement: Supplementary file 11 [file Data_Sheet_9.zip › LR-02/51_5.tiff]

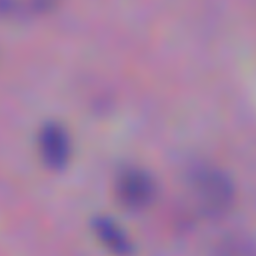

Supplement: Supplementary file 11 [file Data_Sheet_9.zip › LR-02/51_6.tiff]

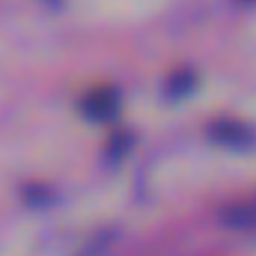

Supplement: Supplementary file 11 [file Data_Sheet_9.zip › LR-02/51_7.tiff]

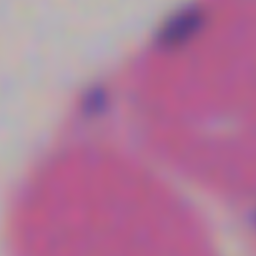

Supplement: Supplementary file 11 [file Data_Sheet_9.zip › LR-02/52_0.tiff]

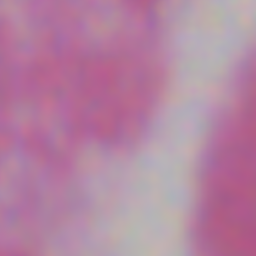

Supplement: Supplementary file 11 [file Data_Sheet_9.zip › LR-02/52_1.tiff]

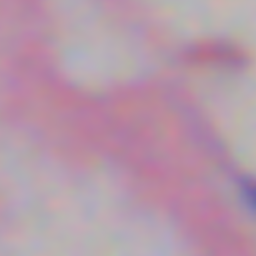

Supplement: Supplementary file 11 [file Data_Sheet_9.zip › LR-02/52_2.tiff]

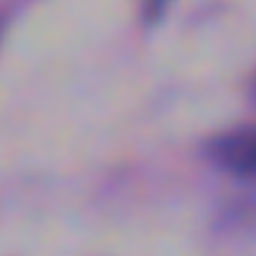

Supplement: Supplementary file 11 [file Data_Sheet_9.zip › LR-02/52_3.tiff]

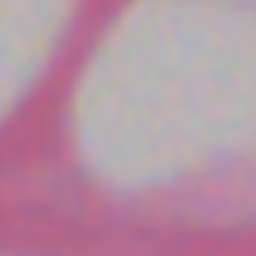

Supplement: Supplementary file 11 [file Data_Sheet_9.zip › LR-02/52_4.tiff]

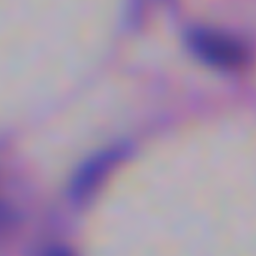

Supplement: Supplementary file 11 [file Data_Sheet_9.zip › LR-02/52_5.tiff]

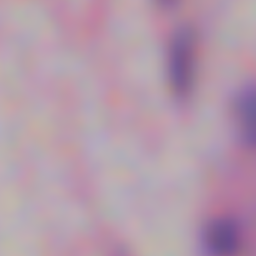

Supplement: Supplementary file 11 [file Data_Sheet_9.zip › LR-02/52_6.tiff]

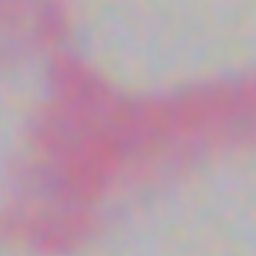

Supplement: Supplementary file 11 [file Data_Sheet_9.zip › LR-02/52_7.tiff]

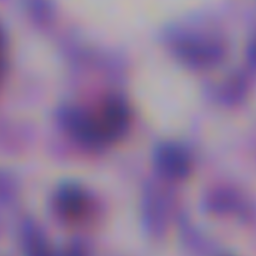

Supplement: Supplementary file 11 [file Data_Sheet_9.zip › LR-02/53_0.tiff]

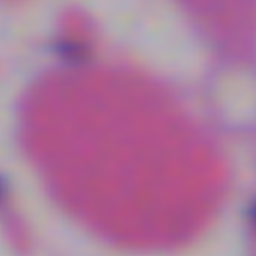

Supplement: Supplementary file 11 [file Data_Sheet_9.zip › LR-02/53_1.tiff]

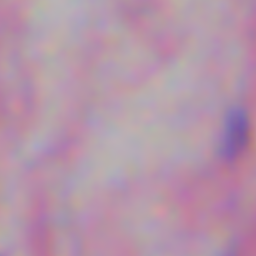

Supplement: Supplementary file 11 [file Data_Sheet_9.zip › LR-02/53_2.tiff]

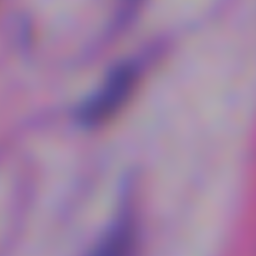

Supplement: Supplementary file 11 [file Data_Sheet_9.zip › LR-02/53_3.tiff]

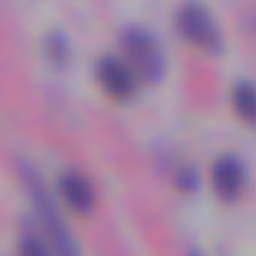

Supplement: Supplementary file 11 [file Data_Sheet_9.zip › LR-02/53_4.tiff]

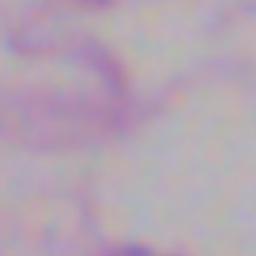

Supplement: Supplementary file 11 [file Data_Sheet_9.zip › LR-02/53_5.tiff]

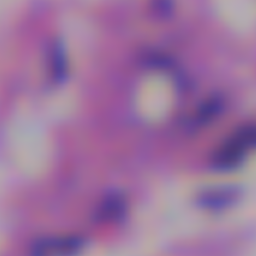

Supplement: Supplementary file 11 [file Data_Sheet_9.zip › LR-02/53_6.tiff]

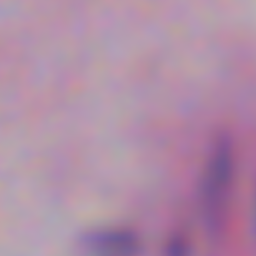

Supplement: Supplementary file 11 [file Data_Sheet_9.zip › LR-02/53_7.tiff]

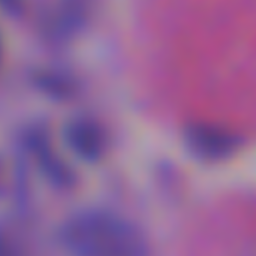

Supplement: Supplementary file 11 [file Data_Sheet_9.zip › LR-02/54_0.tiff]

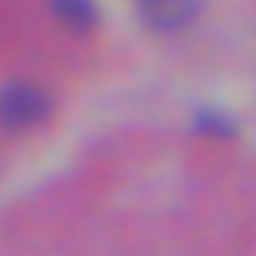

Supplement: Supplementary file 11 [file Data_Sheet_9.zip › LR-02/54_1.tiff]

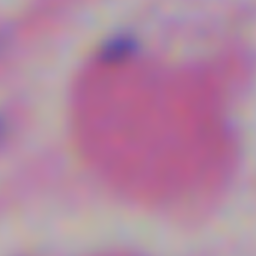

Supplement: Supplementary file 11 [file Data_Sheet_9.zip › LR-02/54_2.tiff]

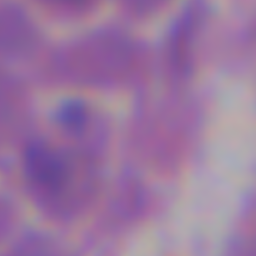

Supplement: Supplementary file 11 [file Data_Sheet_9.zip › LR-02/54_3.tiff]

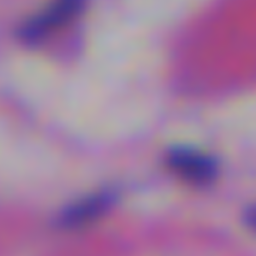

Supplement: Supplementary file 11 [file Data_Sheet_9.zip › LR-02/54_4.tiff]

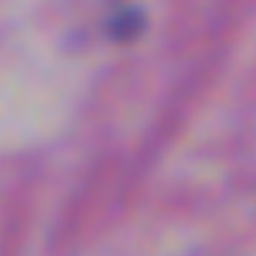

Supplement: Supplementary file 11 [file Data_Sheet_9.zip › LR-02/54_5.tiff]

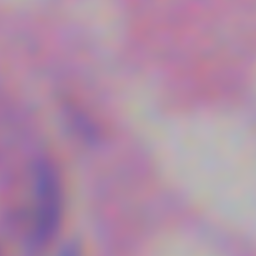

Supplement: Supplementary file 12 [file Data_Sheet_10.zip › LR-03/21_0.tiff]

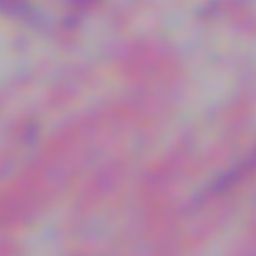

Supplement: Supplementary file 12 [file Data_Sheet_10.zip › LR-03/21_1.tiff]

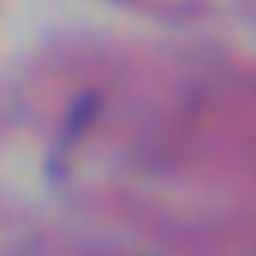

Supplement: Supplementary file 12 [file Data_Sheet_10.zip › LR-03/21_2.tiff]

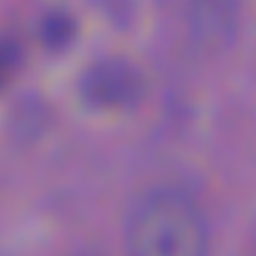

Supplement: Supplementary file 12 [file Data_Sheet_10.zip › LR-03/21_3.tiff]

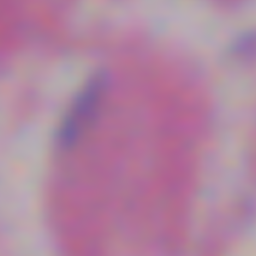

Supplement: Supplementary file 12 [file Data_Sheet_10.zip › LR-03/21_4.tiff]

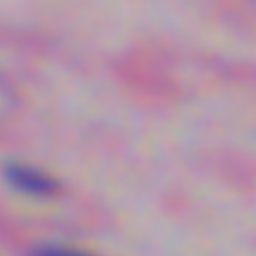

Supplement: Supplementary file 12 [file Data_Sheet_10.zip › LR-03/21_5.tiff]

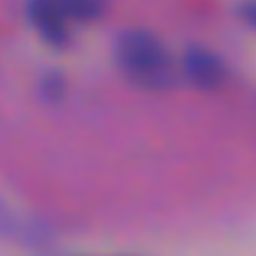

Supplement: Supplementary file 12 [file Data_Sheet_10.zip › LR-03/21_6.tiff]

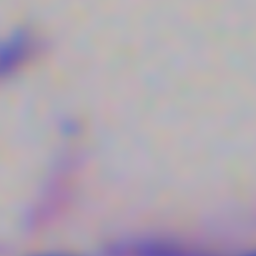

Supplement: Supplementary file 12 [file Data_Sheet_10.zip › LR-03/21_7.tiff]

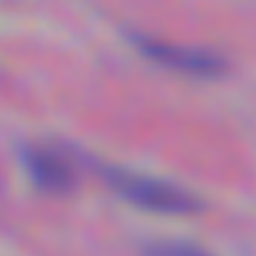

Supplement: Supplementary file 12 [file Data_Sheet_10.zip › LR-03/22_0.tiff]

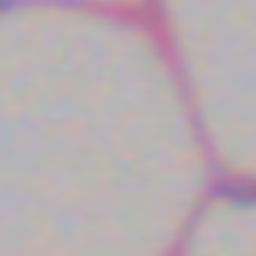

Supplement: Supplementary file 12 [file Data_Sheet_10.zip › LR-03/22_1.tiff]

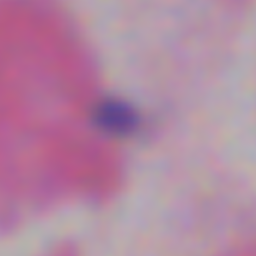

Supplement: Supplementary file 12 [file Data_Sheet_10.zip › LR-03/22_2.tiff]

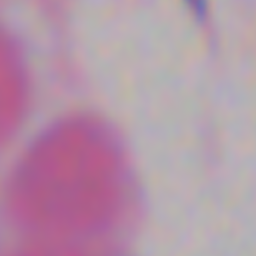

Supplement: Supplementary file 12 [file Data_Sheet_10.zip › LR-03/22_3.tiff]

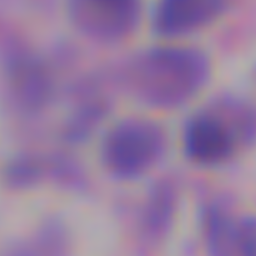

Supplement: Supplementary file 12 [file Data_Sheet_10.zip › LR-03/22_4.tiff]

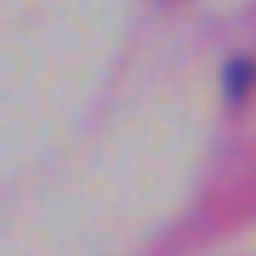

Supplement: Supplementary file 12 [file Data_Sheet_10.zip › LR-03/22_5.tiff]

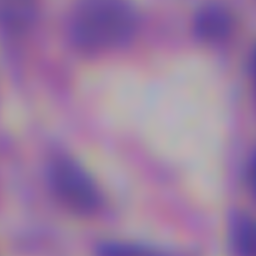

Supplement: Supplementary file 12 [file Data_Sheet_10.zip › LR-03/22_6.tiff]

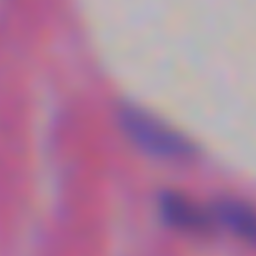

Supplement: Supplementary file 12 [file Data_Sheet_10.zip › LR-03/22_7.tiff]

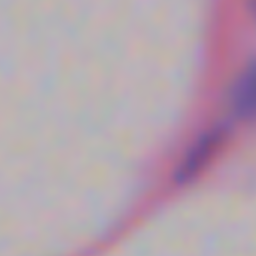

Supplement: Supplementary file 12 [file Data_Sheet_10.zip › LR-03/23_0.tiff]

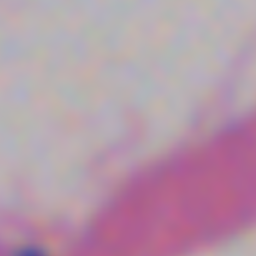

Supplement: Supplementary file 12 [file Data_Sheet_10.zip › LR-03/23_1.tiff]

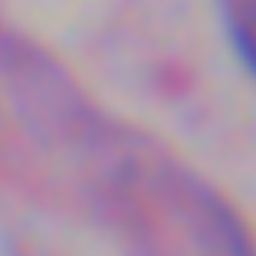

Supplement: Supplementary file 12 [file Data_Sheet_10.zip › LR-03/23_2.tiff]

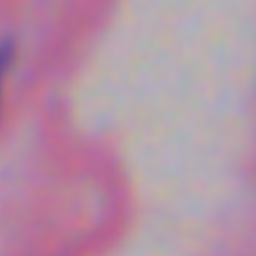

Supplement: Supplementary file 12 [file Data_Sheet_10.zip › LR-03/23_3.tiff]

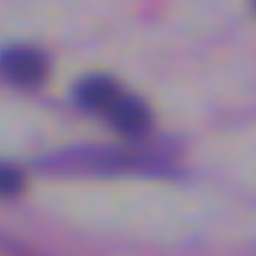

Supplement: Supplementary file 12 [file Data_Sheet_10.zip › LR-03/23_4.tiff]

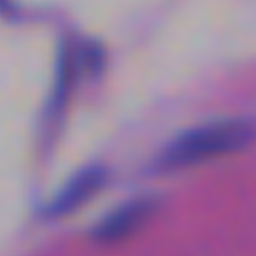

Supplement: Supplementary file 12 [file Data_Sheet_10.zip › LR-03/23_5.tiff]

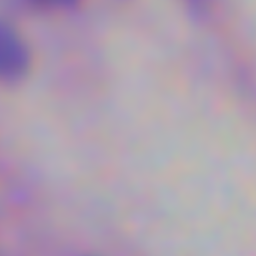

Supplement: Supplementary file 12 [file Data_Sheet_10.zip › LR-03/23_6.tiff]

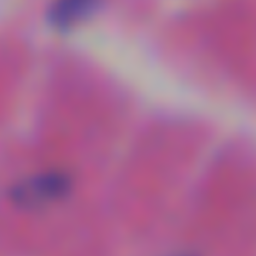

Supplement: Supplementary file 12 [file Data_Sheet_10.zip › LR-03/23_7.tiff]

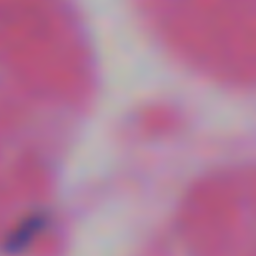

Supplement: Supplementary file 12 [file Data_Sheet_10.zip › LR-03/24_0.tiff]

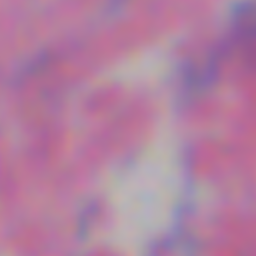

Supplement: Supplementary file 12 [file Data_Sheet_10.zip › LR-03/24_1.tiff]

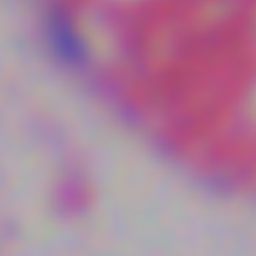

Supplement: Supplementary file 12 [file Data_Sheet_10.zip › LR-03/24_2.tiff]

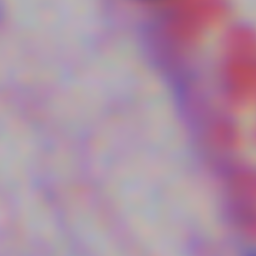

Supplement: Supplementary file 12 [file Data_Sheet_10.zip › LR-03/24_3.tiff]

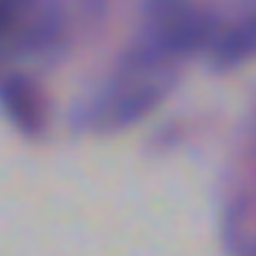

Supplement: Supplementary file 12 [file Data_Sheet_10.zip › LR-03/24_4.tiff]

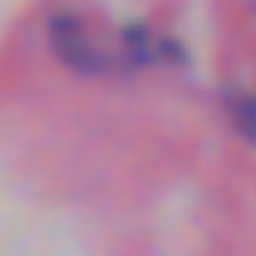

Supplement: Supplementary file 12 [file Data_Sheet_10.zip › LR-03/24_5.tiff]

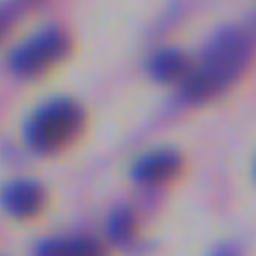

Supplement: Supplementary file 12 [file Data_Sheet_10.zip › LR-03/24_6.tiff]

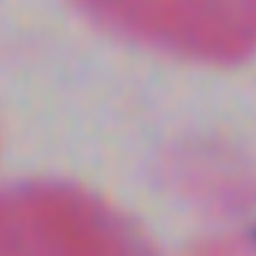

Supplement: Supplementary file 12 [file Data_Sheet_10.zip › LR-03/24_7.tiff]

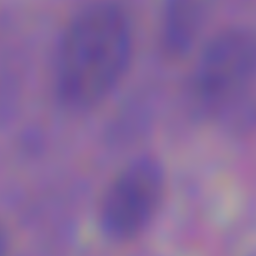

Supplement: Supplementary file 12 [file Data_Sheet_10.zip › LR-03/25_0.tiff]

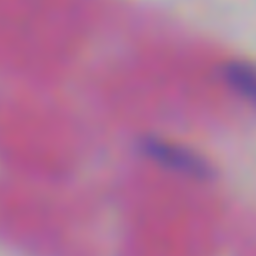

Supplement: Supplementary file 12 [file Data_Sheet_10.zip › LR-03/25_1.tiff]

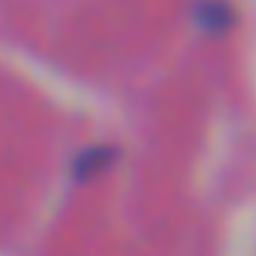

Supplement: Supplementary file 12 [file Data_Sheet_10.zip › LR-03/25_2.tiff]

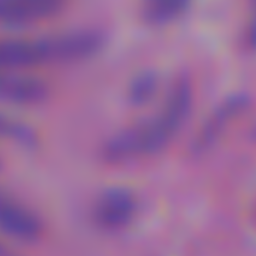

Supplement: Supplementary file 12 [file Data_Sheet_10.zip › LR-03/25_3.tiff]

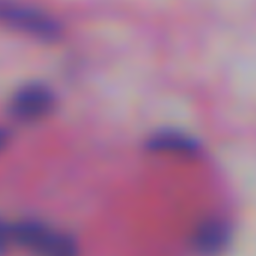

Supplement: Supplementary file 12 [file Data_Sheet_10.zip › LR-03/25_4.tiff]

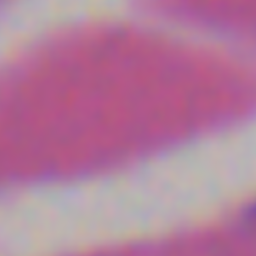

Supplement: Supplementary file 12 [file Data_Sheet_10.zip › LR-03/25_5.tiff]

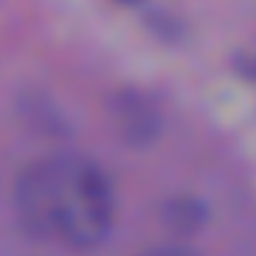

Supplement: Supplementary file 12 [file Data_Sheet_10.zip › LR-03/25_6.tiff]

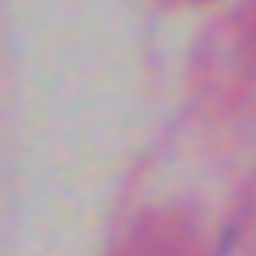

Supplement: Supplementary file 12 [file Data_Sheet_10.zip › LR-03/25_7.tiff]

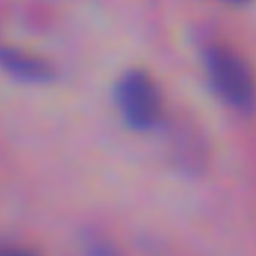

Supplement: Supplementary file 12 [file Data_Sheet_10.zip › LR-03/26_0.tiff]

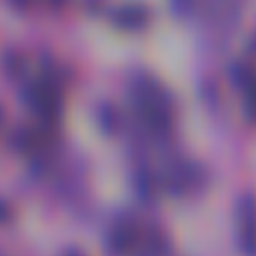

Supplement: Supplementary file 12 [file Data_Sheet_10.zip › LR-03/26_1.tiff]

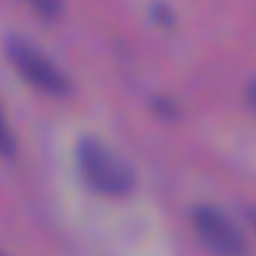

Supplement: Supplementary file 12 [file Data_Sheet_10.zip › LR-03/26_2.tiff]

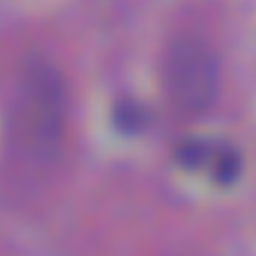

Supplement: Supplementary file 12 [file Data_Sheet_10.zip › LR-03/26_3.tiff]

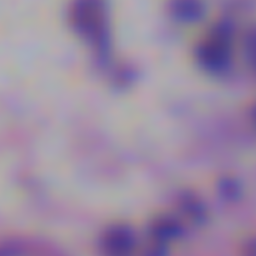

Supplement: Supplementary file 12 [file Data_Sheet_10.zip › LR-03/26_4.tiff]

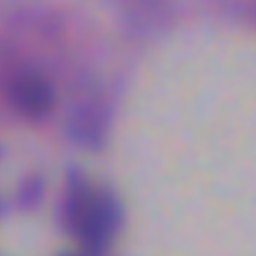

Supplement: Supplementary file 12 [file Data_Sheet_10.zip › LR-03/26_5.tiff]

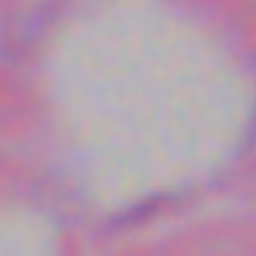

Supplement: Supplementary file 12 [file Data_Sheet_10.zip › LR-03/26_6.tiff]

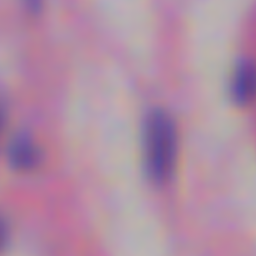

Supplement: Supplementary file 12 [file Data_Sheet_10.zip › LR-03/26_7.tiff]

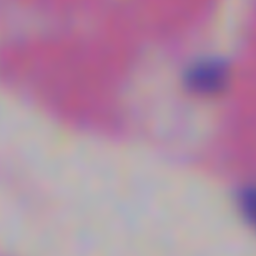

Supplement: Supplementary file 12 [file Data_Sheet_10.zip › LR-03/27_0.tiff]

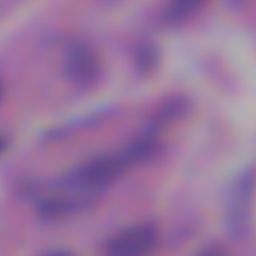

Supplement: Supplementary file 12 [file Data_Sheet_10.zip › LR-03/27_1.tiff]

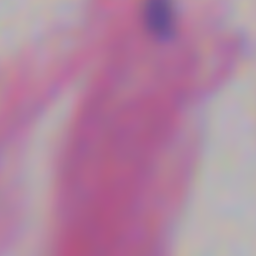

Supplement: Supplementary file 12 [file Data_Sheet_10.zip › LR-03/27_2.tiff]

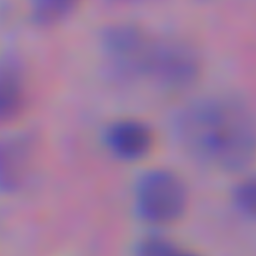

Supplement: Supplementary file 12 [file Data_Sheet_10.zip › LR-03/27_3.tiff]

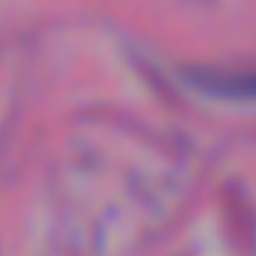

Supplement: Supplementary file 12 [file Data_Sheet_10.zip › LR-03/27_4.tiff]

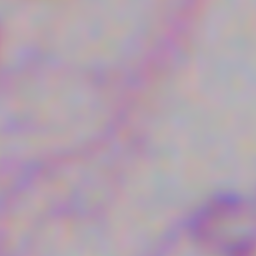

Supplement: Supplementary file 12 [file Data_Sheet_10.zip › LR-03/27_5.tiff]

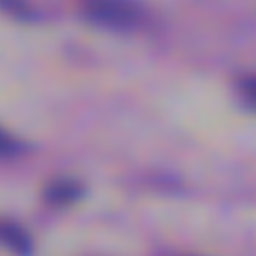

Supplement: Supplementary file 12 [file Data_Sheet_10.zip › LR-03/27_6.tiff]

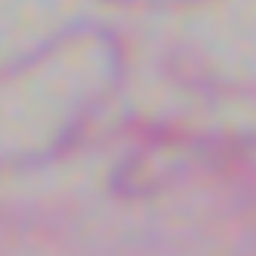

Supplement: Supplementary file 12 [file Data_Sheet_10.zip › LR-03/27_7.tiff]

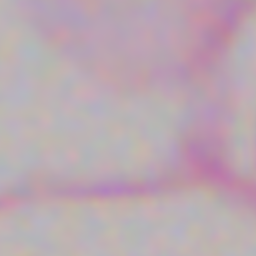

Supplement: Supplementary file 12 [file Data_Sheet_10.zip › LR-03/28_0.tiff]

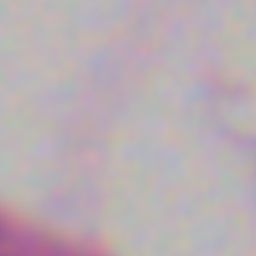

Supplement: Supplementary file 12 [file Data_Sheet_10.zip › LR-03/28_1.tiff]

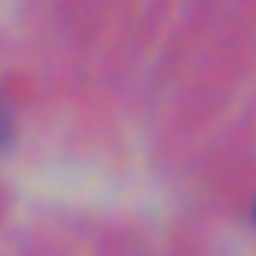

Supplement: Supplementary file 12 [file Data_Sheet_10.zip › LR-03/28_2.tiff]

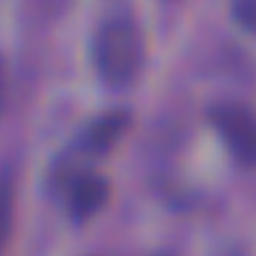

Supplement: Supplementary file 12 [file Data_Sheet_10.zip › LR-03/28_3.tiff]

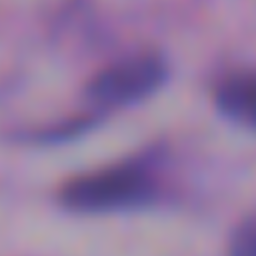

Supplement: Supplementary file 12 [file Data_Sheet_10.zip › LR-03/28_4.tiff]

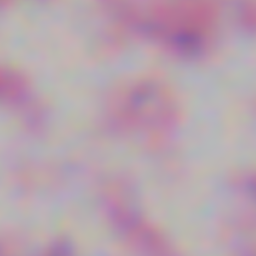

Supplement: Supplementary file 12 [file Data_Sheet_10.zip › LR-03/28_5.tiff]

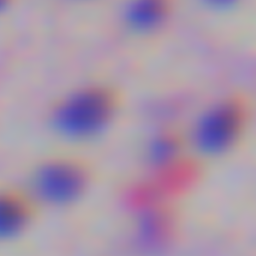

Supplement: Supplementary file 12 [file Data_Sheet_10.zip › LR-03/28_6.tiff]

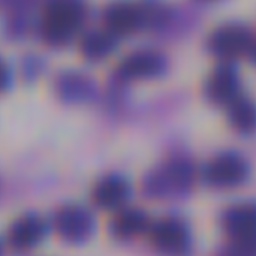

Supplement: Supplementary file 12 [file Data_Sheet_10.zip › LR-03/28_7.tiff]
