# Supplementary figures and images for: Feasibility study of single-image super-resolution scanning system based on deep learning for pathological diagnosis of oral epithelial dysplasia (part 14 of 21)
Source: Front Med (Lausanne). 2025 Mar 12;12:1550512. doi: 10.3389/fmed.2025.1550512 (PMC11936936; doi:10.3389/fmed.2025.1550512)

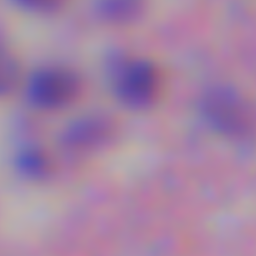

Supplement: Supplementary file 12 [file Data_Sheet_10.zip › LR-03/71_6.tiff]

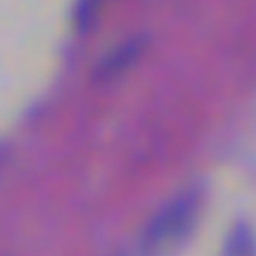

Supplement: Supplementary file 12 [file Data_Sheet_10.zip › LR-03/71_7.tiff]

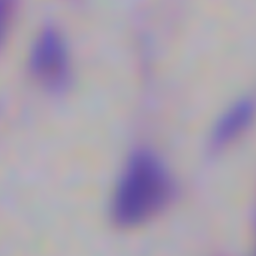

Supplement: Supplementary file 12 [file Data_Sheet_10.zip › LR-03/72_0.tiff]

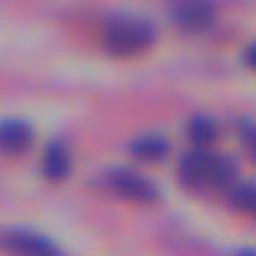

Supplement: Supplementary file 12 [file Data_Sheet_10.zip › LR-03/72_1.tiff]

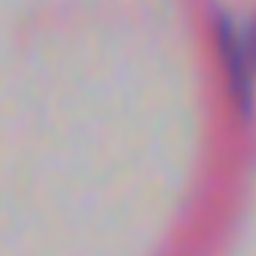

Supplement: Supplementary file 12 [file Data_Sheet_10.zip › LR-03/72_2.tiff]

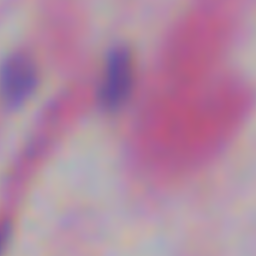

Supplement: Supplementary file 12 [file Data_Sheet_10.zip › LR-03/72_3.tiff]

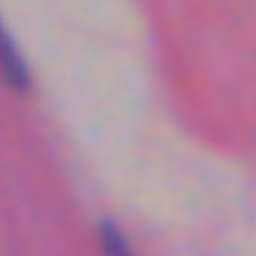

Supplement: Supplementary file 12 [file Data_Sheet_10.zip › LR-03/72_4.tiff]

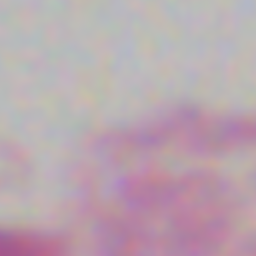

Supplement: Supplementary file 12 [file Data_Sheet_10.zip › LR-03/72_5.tiff]

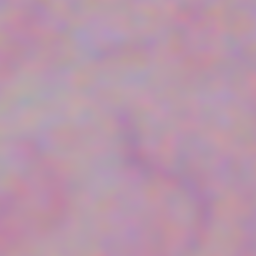

Supplement: Supplementary file 12 [file Data_Sheet_10.zip › LR-03/72_6.tiff]

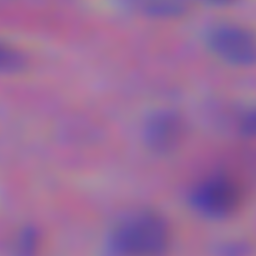

Supplement: Supplementary file 12 [file Data_Sheet_10.zip › LR-03/72_7.tiff]

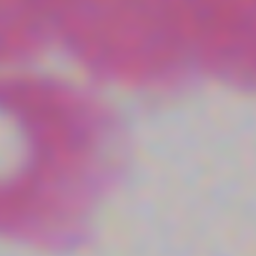

Supplement: Supplementary file 12 [file Data_Sheet_10.zip › LR-03/73_0.tiff]

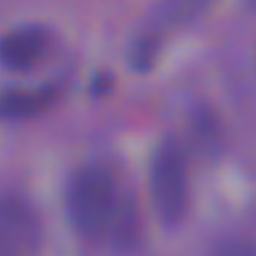

Supplement: Supplementary file 12 [file Data_Sheet_10.zip › LR-03/73_1.tiff]

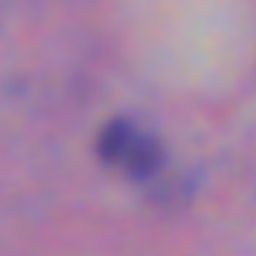

Supplement: Supplementary file 12 [file Data_Sheet_10.zip › LR-03/73_2.tiff]

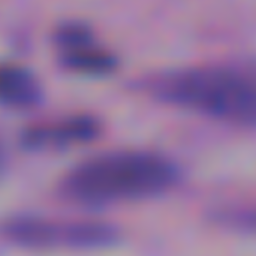

Supplement: Supplementary file 12 [file Data_Sheet_10.zip › LR-03/73_3.tiff]

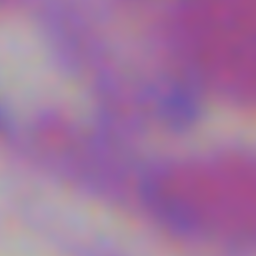

Supplement: Supplementary file 12 [file Data_Sheet_10.zip › LR-03/73_4.tiff]

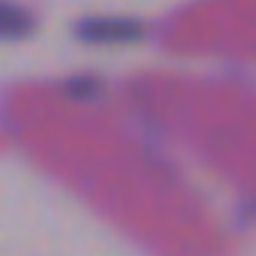

Supplement: Supplementary file 12 [file Data_Sheet_10.zip › LR-03/73_5.tiff]

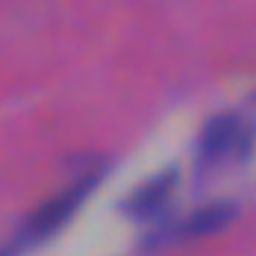

Supplement: Supplementary file 12 [file Data_Sheet_10.zip › LR-03/73_6.tiff]

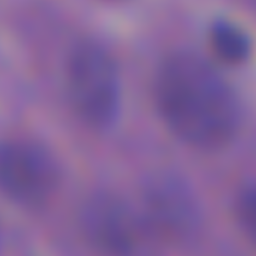

Supplement: Supplementary file 12 [file Data_Sheet_10.zip › LR-03/73_7.tiff]

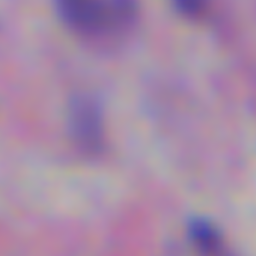

Supplement: Supplementary file 12 [file Data_Sheet_10.zip › LR-03/74_0.tiff]

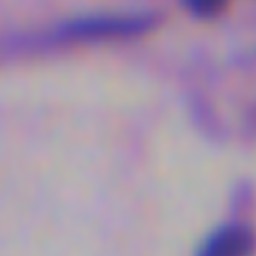

Supplement: Supplementary file 12 [file Data_Sheet_10.zip › LR-03/74_1.tiff]

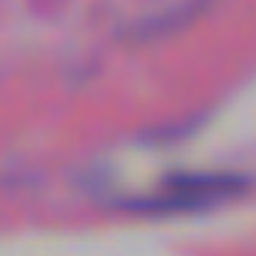

Supplement: Supplementary file 12 [file Data_Sheet_10.zip › LR-03/74_2.tiff]

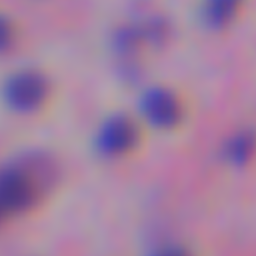

Supplement: Supplementary file 12 [file Data_Sheet_10.zip › LR-03/74_3.tiff]

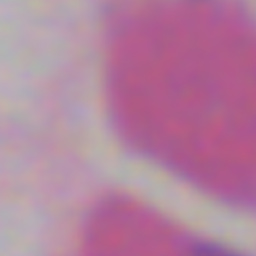

Supplement: Supplementary file 12 [file Data_Sheet_10.zip › LR-03/74_4.tiff]

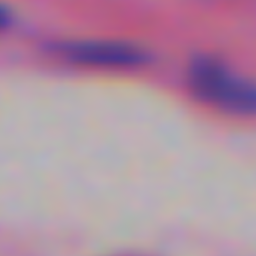

Supplement: Supplementary file 12 [file Data_Sheet_10.zip › LR-03/74_5.tiff]

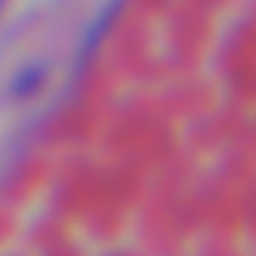

Supplement: Supplementary file 12 [file Data_Sheet_10.zip › LR-03/74_6.tiff]

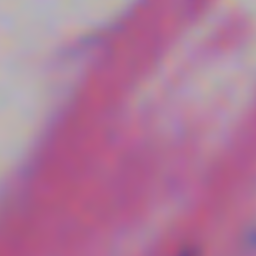

Supplement: Supplementary file 12 [file Data_Sheet_10.zip › LR-03/74_7.tiff]

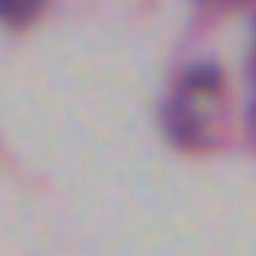

Supplement: Supplementary file 12 [file Data_Sheet_10.zip › LR-03/75_0.tiff]

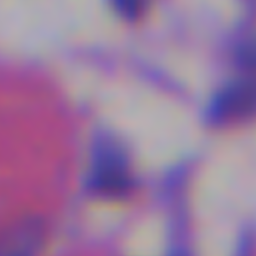

Supplement: Supplementary file 12 [file Data_Sheet_10.zip › LR-03/75_1.tiff]

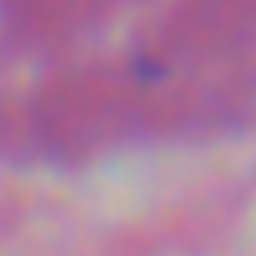

Supplement: Supplementary file 12 [file Data_Sheet_10.zip › LR-03/75_2.tiff]

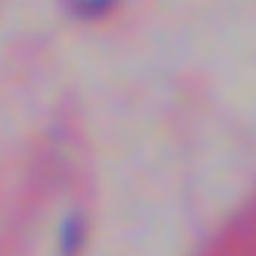

Supplement: Supplementary file 12 [file Data_Sheet_10.zip › LR-03/75_3.tiff]

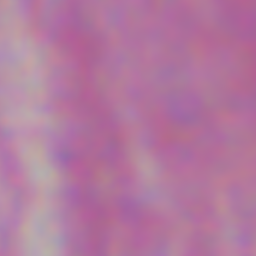

Supplement: Supplementary file 12 [file Data_Sheet_10.zip › LR-03/75_4.tiff]

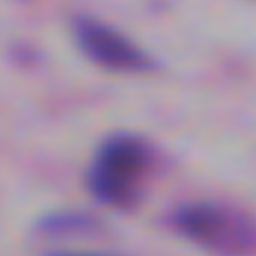

Supplement: Supplementary file 12 [file Data_Sheet_10.zip › LR-03/75_5.tiff]

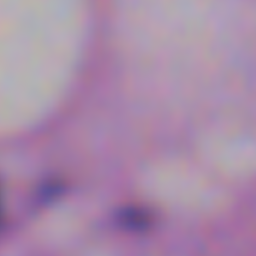

Supplement: Supplementary file 12 [file Data_Sheet_10.zip › LR-03/75_6.tiff]

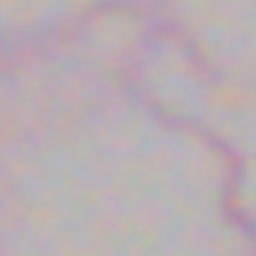

Supplement: Supplementary file 12 [file Data_Sheet_10.zip › LR-03/75_7.tiff]

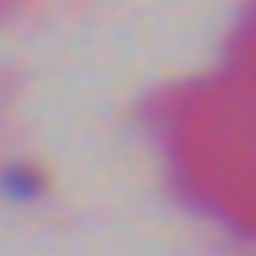

Supplement: Supplementary file 12 [file Data_Sheet_10.zip › LR-03/76_0.tiff]

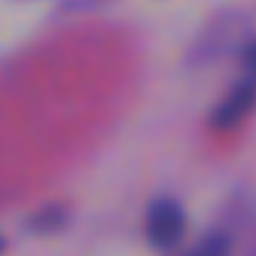

Supplement: Supplementary file 12 [file Data_Sheet_10.zip › LR-03/76_1.tiff]

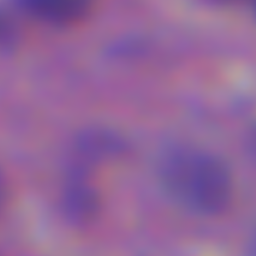

Supplement: Supplementary file 13 [file Data_Sheet_11.zip › LR-04/100_0.tiff]

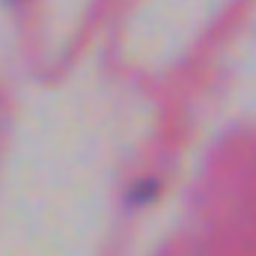

Supplement: Supplementary file 13 [file Data_Sheet_11.zip › LR-04/100_1.tiff]

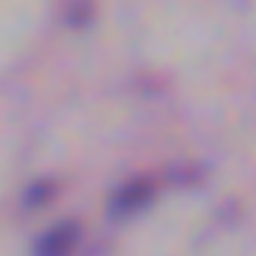

Supplement: Supplementary file 13 [file Data_Sheet_11.zip › LR-04/100_2.tiff]

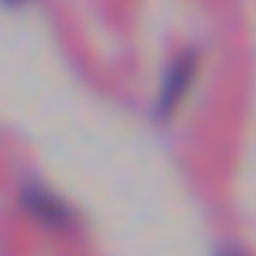

Supplement: Supplementary file 13 [file Data_Sheet_11.zip › LR-04/100_3.tiff]

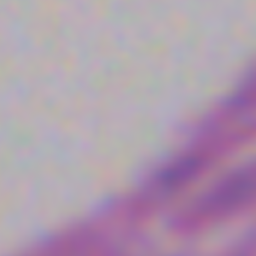

Supplement: Supplementary file 13 [file Data_Sheet_11.zip › LR-04/100_4.tiff]

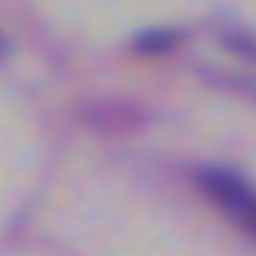

Supplement: Supplementary file 13 [file Data_Sheet_11.zip › LR-04/100_5.tiff]

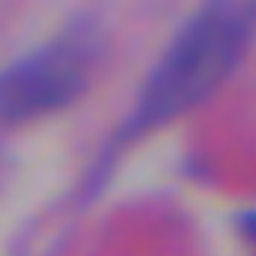

Supplement: Supplementary file 13 [file Data_Sheet_11.zip › LR-04/100_6.tiff]

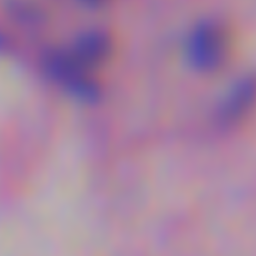

Supplement: Supplementary file 13 [file Data_Sheet_11.zip › LR-04/100_7.tiff]

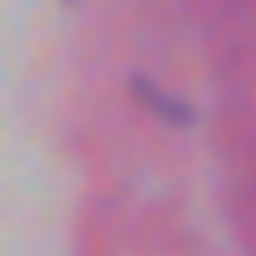

Supplement: Supplementary file 13 [file Data_Sheet_11.zip › LR-04/101_0.tiff]

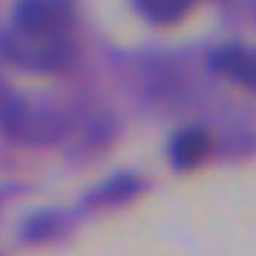

Supplement: Supplementary file 13 [file Data_Sheet_11.zip › LR-04/101_1.tiff]

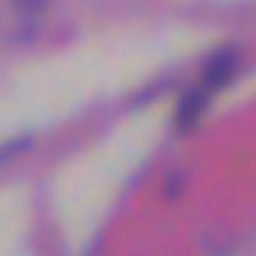

Supplement: Supplementary file 13 [file Data_Sheet_11.zip › LR-04/101_2.tiff]

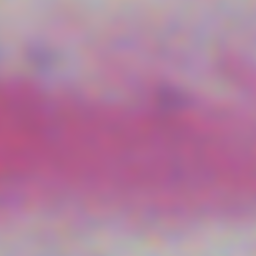

Supplement: Supplementary file 13 [file Data_Sheet_11.zip › LR-04/101_3.tiff]

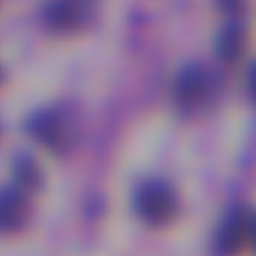

Supplement: Supplementary file 13 [file Data_Sheet_11.zip › LR-04/101_4.tiff]

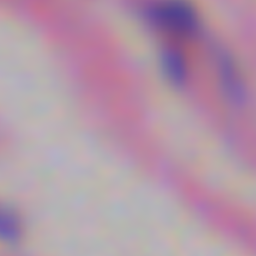

Supplement: Supplementary file 13 [file Data_Sheet_11.zip › LR-04/101_5.tiff]

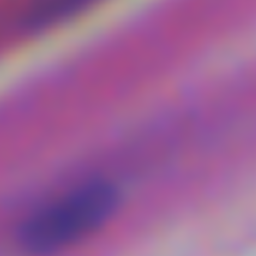

Supplement: Supplementary file 13 [file Data_Sheet_11.zip › LR-04/101_6.tiff]

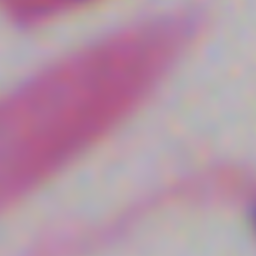

Supplement: Supplementary file 13 [file Data_Sheet_11.zip › LR-04/101_7.tiff]

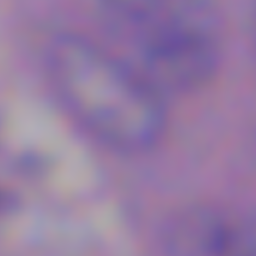

Supplement: Supplementary file 13 [file Data_Sheet_11.zip › LR-04/102_0.tiff]

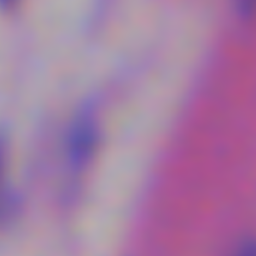

Supplement: Supplementary file 13 [file Data_Sheet_11.zip › LR-04/102_1.tiff]

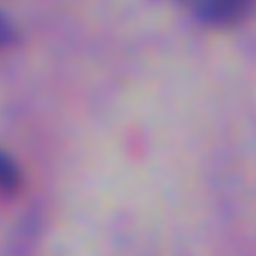

Supplement: Supplementary file 13 [file Data_Sheet_11.zip › LR-04/102_2.tiff]

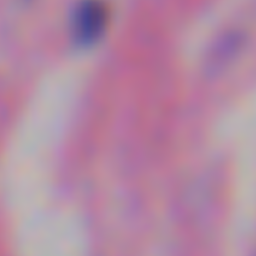

Supplement: Supplementary file 13 [file Data_Sheet_11.zip › LR-04/102_3.tiff]

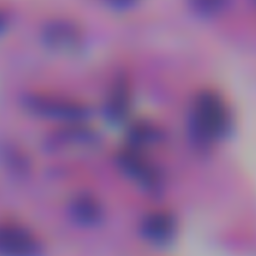

Supplement: Supplementary file 13 [file Data_Sheet_11.zip › LR-04/102_4.tiff]

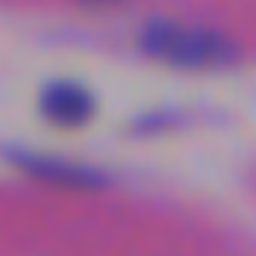

Supplement: Supplementary file 13 [file Data_Sheet_11.zip › LR-04/102_5.tiff]

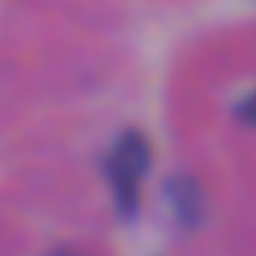

Supplement: Supplementary file 13 [file Data_Sheet_11.zip › LR-04/102_6.tiff]

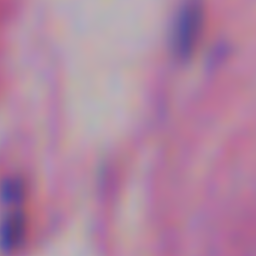

Supplement: Supplementary file 13 [file Data_Sheet_11.zip › LR-04/102_7.tiff]

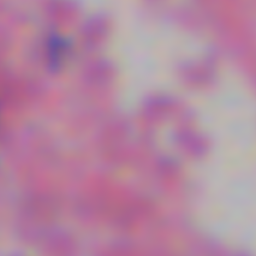

Supplement: Supplementary file 13 [file Data_Sheet_11.zip › LR-04/103_0.tiff]

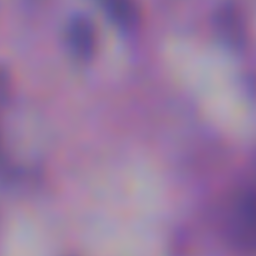

Supplement: Supplementary file 13 [file Data_Sheet_11.zip › LR-04/103_1.tiff]

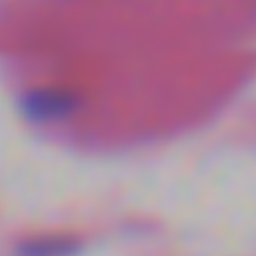

Supplement: Supplementary file 13 [file Data_Sheet_11.zip › LR-04/103_2.tiff]

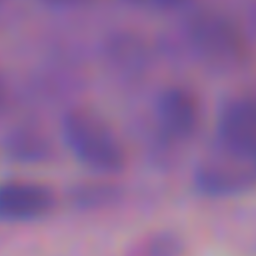

Supplement: Supplementary file 13 [file Data_Sheet_11.zip › LR-04/103_3.tiff]

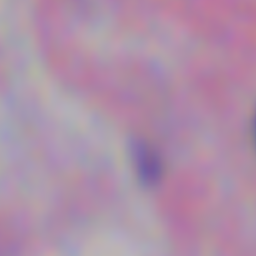

Supplement: Supplementary file 13 [file Data_Sheet_11.zip › LR-04/103_4.tiff]

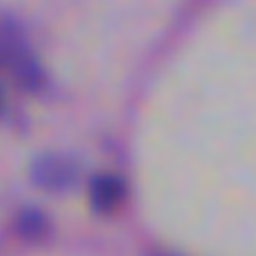

Supplement: Supplementary file 13 [file Data_Sheet_11.zip › LR-04/103_5.tiff]

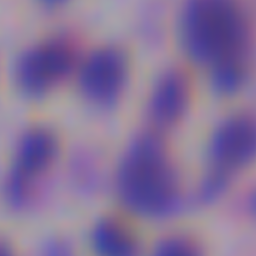

Supplement: Supplementary file 13 [file Data_Sheet_11.zip › LR-04/103_6.tiff]

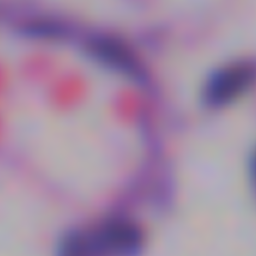

Supplement: Supplementary file 13 [file Data_Sheet_11.zip › LR-04/103_7.tiff]

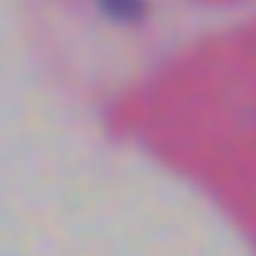

Supplement: Supplementary file 13 [file Data_Sheet_11.zip › LR-04/104_0.tiff]

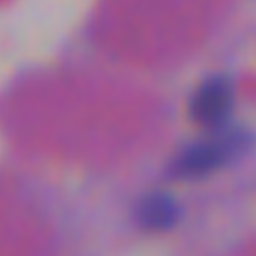

Supplement: Supplementary file 13 [file Data_Sheet_11.zip › LR-04/104_1.tiff]

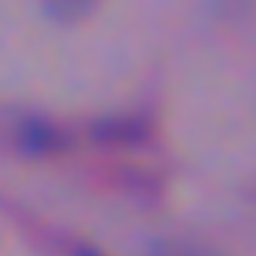

Supplement: Supplementary file 13 [file Data_Sheet_11.zip › LR-04/104_2.tiff]

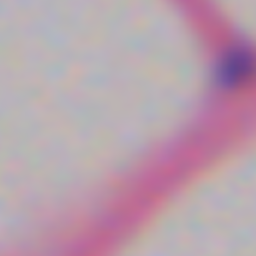

Supplement: Supplementary file 13 [file Data_Sheet_11.zip › LR-04/104_3.tiff]

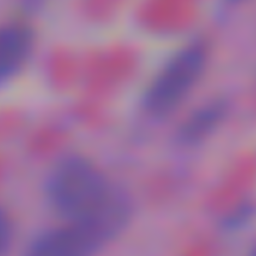

Supplement: Supplementary file 13 [file Data_Sheet_11.zip › LR-04/104_4.tiff]

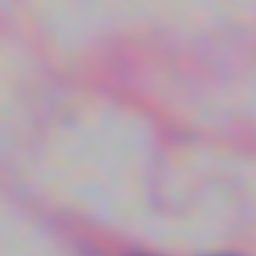

Supplement: Supplementary file 13 [file Data_Sheet_11.zip › LR-04/104_5.tiff]

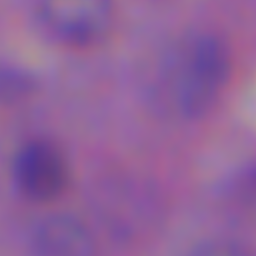

Supplement: Supplementary file 13 [file Data_Sheet_11.zip › LR-04/104_6.tiff]

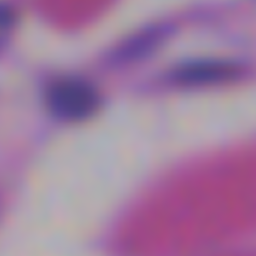

Supplement: Supplementary file 13 [file Data_Sheet_11.zip › LR-04/104_7.tiff]

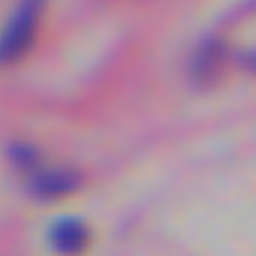

Supplement: Supplementary file 13 [file Data_Sheet_11.zip › LR-04/105_0.tiff]

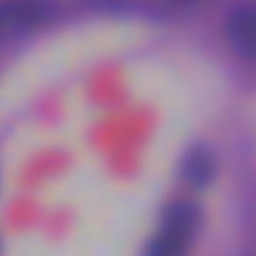

Supplement: Supplementary file 13 [file Data_Sheet_11.zip › LR-04/105_1.tiff]

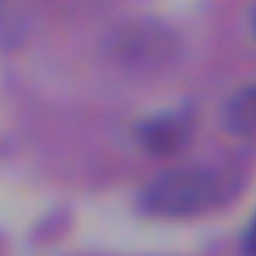

Supplement: Supplementary file 13 [file Data_Sheet_11.zip › LR-04/105_2.tiff]

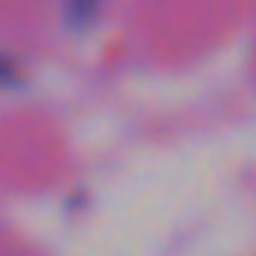

Supplement: Supplementary file 13 [file Data_Sheet_11.zip › LR-04/105_3.tiff]

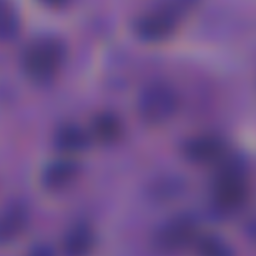

Supplement: Supplementary file 13 [file Data_Sheet_11.zip › LR-04/105_4.tiff]

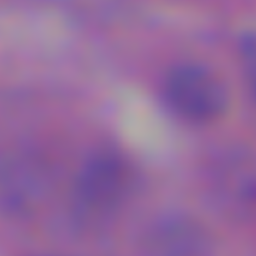

Supplement: Supplementary file 13 [file Data_Sheet_11.zip › LR-04/105_5.tiff]

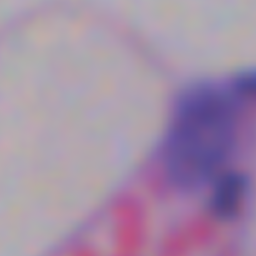

Supplement: Supplementary file 13 [file Data_Sheet_11.zip › LR-04/105_6.tiff]

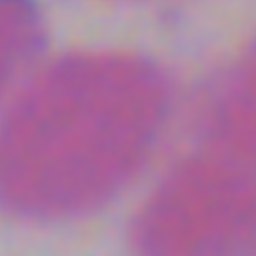

Supplement: Supplementary file 13 [file Data_Sheet_11.zip › LR-04/105_7.tiff]

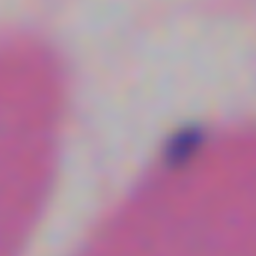

Supplement: Supplementary file 13 [file Data_Sheet_11.zip › LR-04/106_0.tiff]

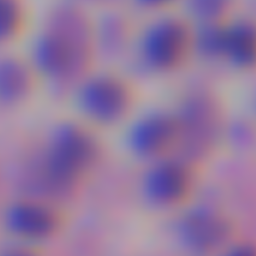

Supplement: Supplementary file 13 [file Data_Sheet_11.zip › LR-04/106_1.tiff]

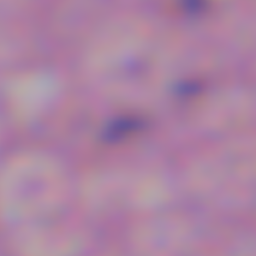

Supplement: Supplementary file 13 [file Data_Sheet_11.zip › LR-04/106_2.tiff]

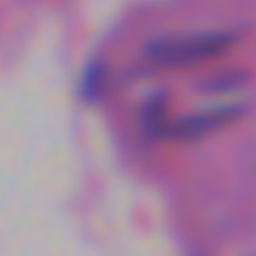

Supplement: Supplementary file 13 [file Data_Sheet_11.zip › LR-04/106_3.tiff]

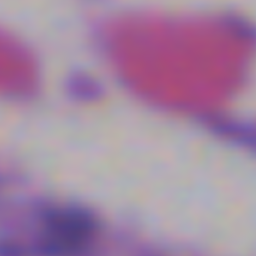

Supplement: Supplementary file 13 [file Data_Sheet_11.zip › LR-04/106_4.tiff]

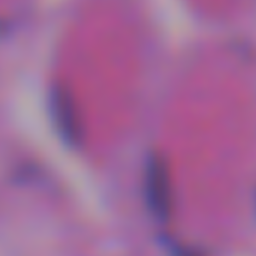

Supplement: Supplementary file 13 [file Data_Sheet_11.zip › LR-04/106_5.tiff]

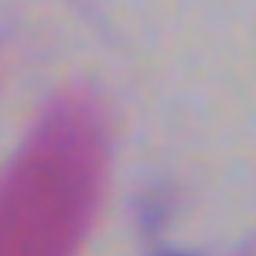

Supplement: Supplementary file 13 [file Data_Sheet_11.zip › LR-04/106_6.tiff]

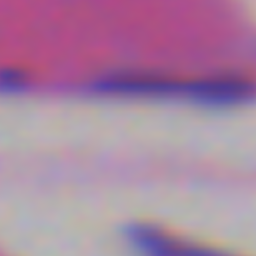

Supplement: Supplementary file 13 [file Data_Sheet_11.zip › LR-04/106_7.tiff]

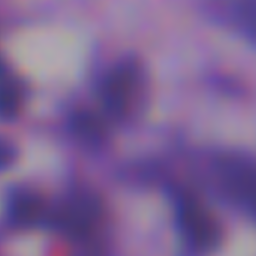

Supplement: Supplementary file 13 [file Data_Sheet_11.zip › LR-04/107_0.tiff]

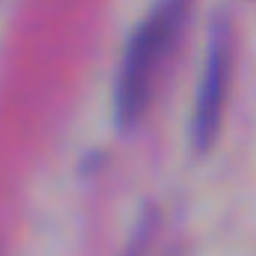

Supplement: Supplementary file 13 [file Data_Sheet_11.zip › LR-04/107_1.tiff]

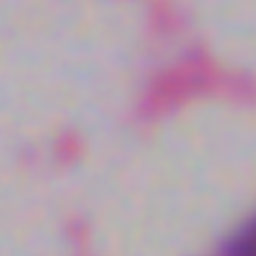

Supplement: Supplementary file 13 [file Data_Sheet_11.zip › LR-04/107_2.tiff]

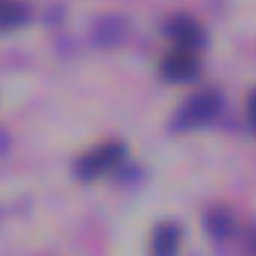

Supplement: Supplementary file 13 [file Data_Sheet_11.zip › LR-04/107_3.tiff]

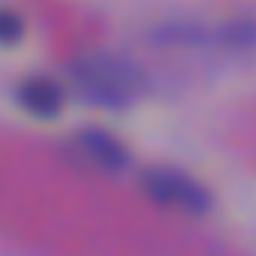

Supplement: Supplementary file 13 [file Data_Sheet_11.zip › LR-04/107_4.tiff]

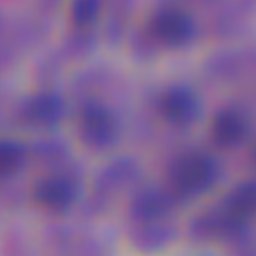

Supplement: Supplementary file 13 [file Data_Sheet_11.zip › LR-04/107_5.tiff]

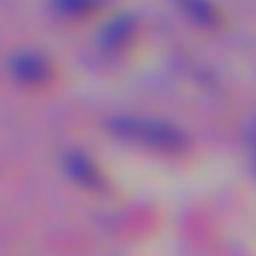

Supplement: Supplementary file 13 [file Data_Sheet_11.zip › LR-04/107_6.tiff]

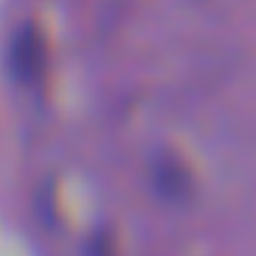

Supplement: Supplementary file 13 [file Data_Sheet_11.zip › LR-04/107_7.tiff]
