# Supplementary figures and images for: Feasibility study of single-image super-resolution scanning system based on deep learning for pathological diagnosis of oral epithelial dysplasia (part 13 of 21)
Source: Front Med (Lausanne). 2025 Mar 12;12:1550512. doi: 10.3389/fmed.2025.1550512 (PMC11936936; doi:10.3389/fmed.2025.1550512)

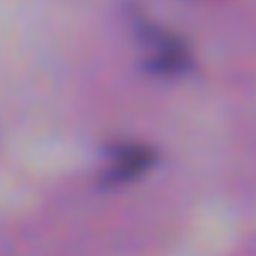

Supplement: Supplementary file 12 [file Data_Sheet_10.zip › LR-03/29_0.tiff]

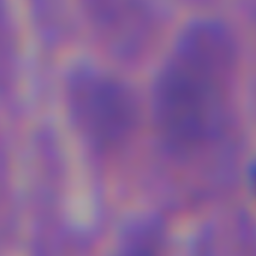

Supplement: Supplementary file 12 [file Data_Sheet_10.zip › LR-03/29_1.tiff]

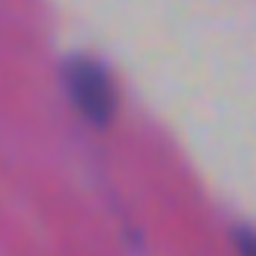

Supplement: Supplementary file 12 [file Data_Sheet_10.zip › LR-03/29_2.tiff]

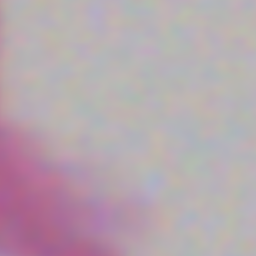

Supplement: Supplementary file 12 [file Data_Sheet_10.zip › LR-03/29_3.tiff]

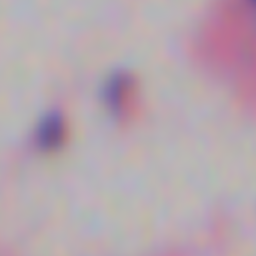

Supplement: Supplementary file 12 [file Data_Sheet_10.zip › LR-03/29_4.tiff]

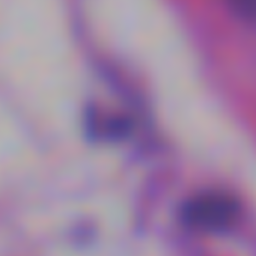

Supplement: Supplementary file 12 [file Data_Sheet_10.zip › LR-03/29_5.tiff]

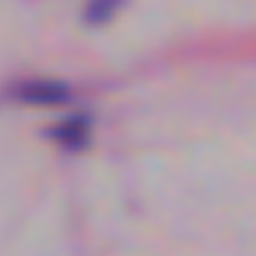

Supplement: Supplementary file 12 [file Data_Sheet_10.zip › LR-03/60_0.tiff]

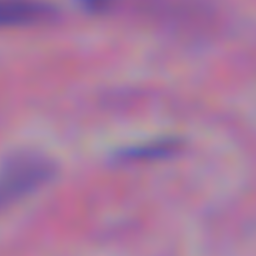

Supplement: Supplementary file 12 [file Data_Sheet_10.zip › LR-03/60_1.tiff]

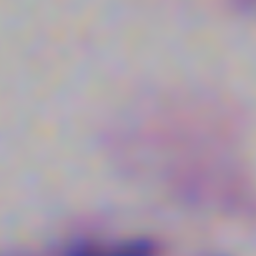

Supplement: Supplementary file 12 [file Data_Sheet_10.zip › LR-03/60_2.tiff]

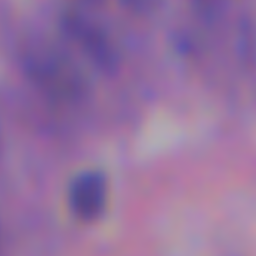

Supplement: Supplementary file 12 [file Data_Sheet_10.zip › LR-03/60_3.tiff]

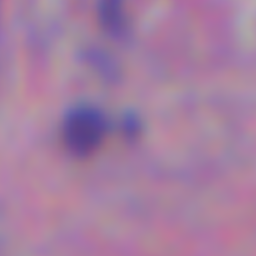

Supplement: Supplementary file 12 [file Data_Sheet_10.zip › LR-03/60_4.tiff]

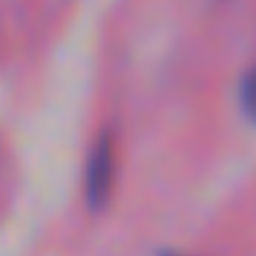

Supplement: Supplementary file 12 [file Data_Sheet_10.zip › LR-03/60_5.tiff]

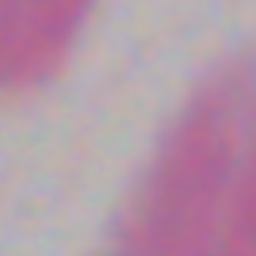

Supplement: Supplementary file 12 [file Data_Sheet_10.zip › LR-03/60_6.tiff]

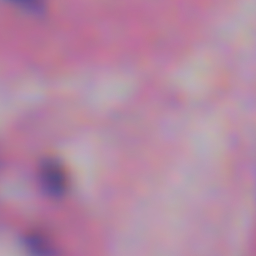

Supplement: Supplementary file 12 [file Data_Sheet_10.zip › LR-03/60_7.tiff]

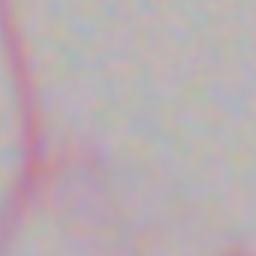

Supplement: Supplementary file 12 [file Data_Sheet_10.zip › LR-03/61_0.tiff]

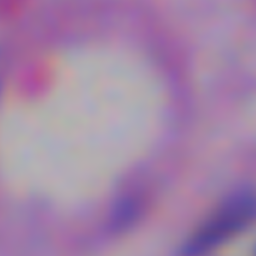

Supplement: Supplementary file 12 [file Data_Sheet_10.zip › LR-03/61_1.tiff]

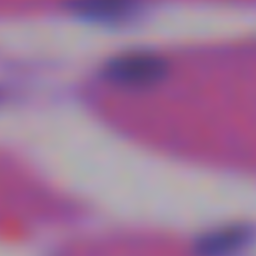

Supplement: Supplementary file 12 [file Data_Sheet_10.zip › LR-03/61_2.tiff]

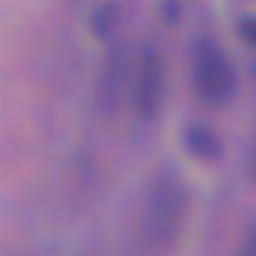

Supplement: Supplementary file 12 [file Data_Sheet_10.zip › LR-03/61_3.tiff]

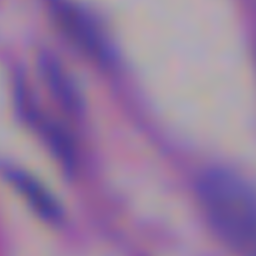

Supplement: Supplementary file 12 [file Data_Sheet_10.zip › LR-03/61_4.tiff]

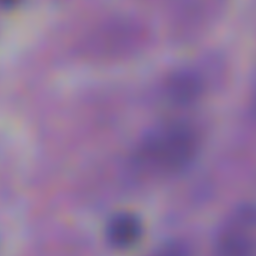

Supplement: Supplementary file 12 [file Data_Sheet_10.zip › LR-03/61_5.tiff]

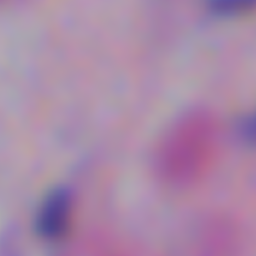

Supplement: Supplementary file 12 [file Data_Sheet_10.zip › LR-03/61_6.tiff]

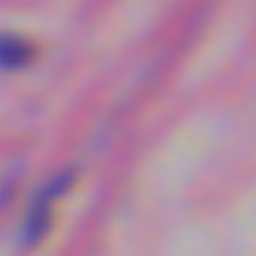

Supplement: Supplementary file 12 [file Data_Sheet_10.zip › LR-03/61_7.tiff]

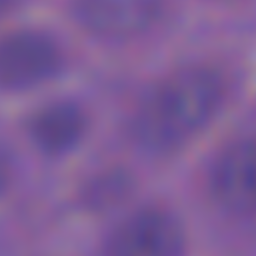

Supplement: Supplementary file 12 [file Data_Sheet_10.zip › LR-03/62_0.tiff]

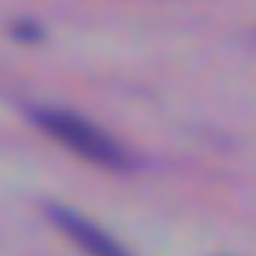

Supplement: Supplementary file 12 [file Data_Sheet_10.zip › LR-03/62_1.tiff]

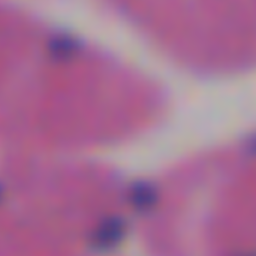

Supplement: Supplementary file 12 [file Data_Sheet_10.zip › LR-03/62_2.tiff]

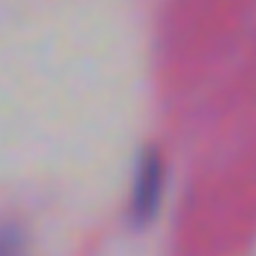

Supplement: Supplementary file 12 [file Data_Sheet_10.zip › LR-03/62_3.tiff]

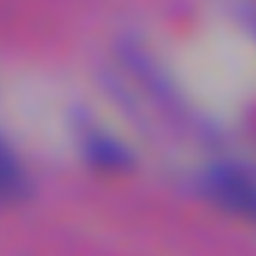

Supplement: Supplementary file 12 [file Data_Sheet_10.zip › LR-03/62_4.tiff]

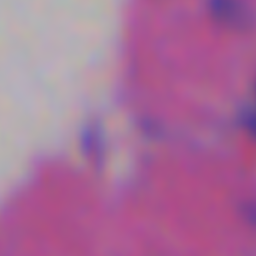

Supplement: Supplementary file 12 [file Data_Sheet_10.zip › LR-03/62_5.tiff]

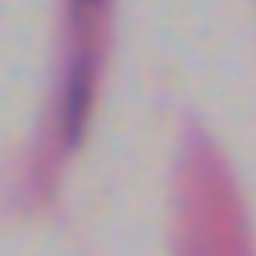

Supplement: Supplementary file 12 [file Data_Sheet_10.zip › LR-03/62_6.tiff]

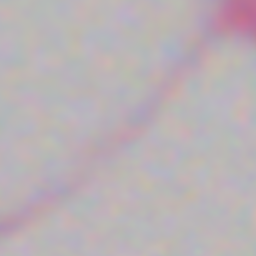

Supplement: Supplementary file 12 [file Data_Sheet_10.zip › LR-03/62_7.tiff]

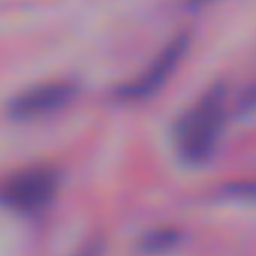

Supplement: Supplementary file 12 [file Data_Sheet_10.zip › LR-03/63_0.tiff]

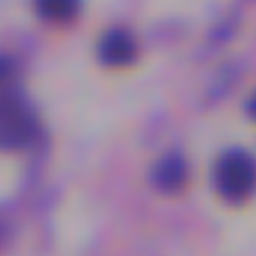

Supplement: Supplementary file 12 [file Data_Sheet_10.zip › LR-03/63_1.tiff]

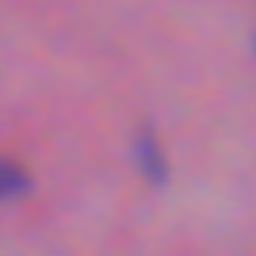

Supplement: Supplementary file 12 [file Data_Sheet_10.zip › LR-03/63_2.tiff]

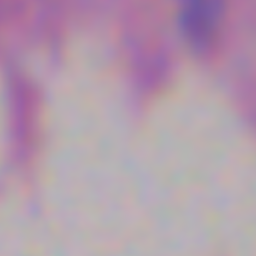

Supplement: Supplementary file 12 [file Data_Sheet_10.zip › LR-03/63_3.tiff]

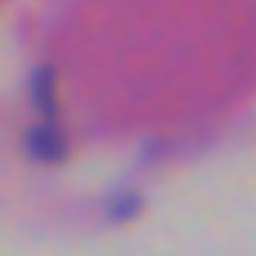

Supplement: Supplementary file 12 [file Data_Sheet_10.zip › LR-03/63_4.tiff]

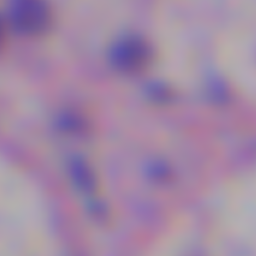

Supplement: Supplementary file 12 [file Data_Sheet_10.zip › LR-03/63_5.tiff]

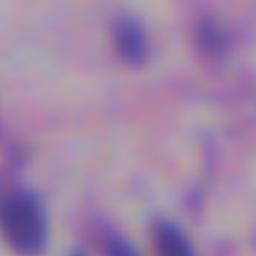

Supplement: Supplementary file 12 [file Data_Sheet_10.zip › LR-03/63_6.tiff]

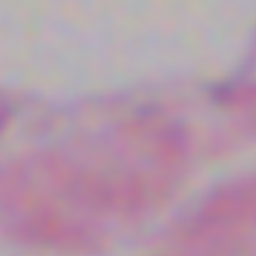

Supplement: Supplementary file 12 [file Data_Sheet_10.zip › LR-03/63_7.tiff]

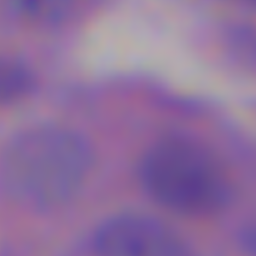

Supplement: Supplementary file 12 [file Data_Sheet_10.zip › LR-03/64_0.tiff]

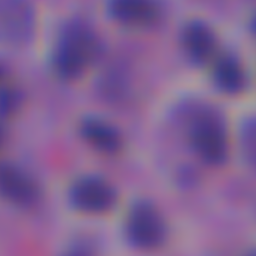

Supplement: Supplementary file 12 [file Data_Sheet_10.zip › LR-03/64_1.tiff]

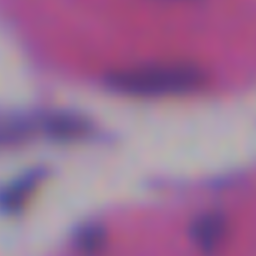

Supplement: Supplementary file 12 [file Data_Sheet_10.zip › LR-03/64_2.tiff]

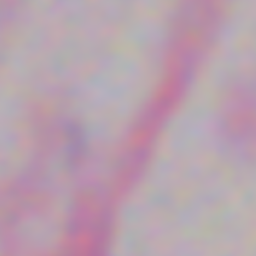

Supplement: Supplementary file 12 [file Data_Sheet_10.zip › LR-03/64_3.tiff]

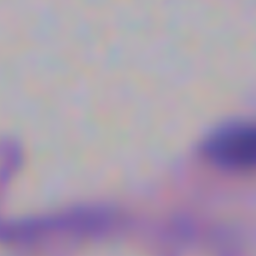

Supplement: Supplementary file 12 [file Data_Sheet_10.zip › LR-03/64_4.tiff]

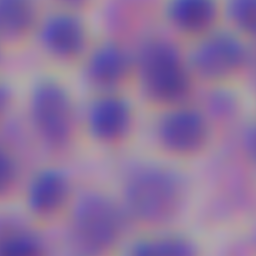

Supplement: Supplementary file 12 [file Data_Sheet_10.zip › LR-03/64_5.tiff]

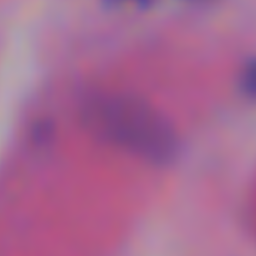

Supplement: Supplementary file 12 [file Data_Sheet_10.zip › LR-03/64_6.tiff]

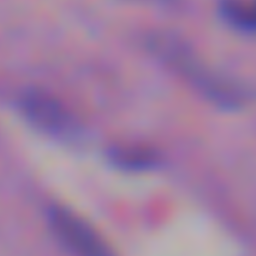

Supplement: Supplementary file 12 [file Data_Sheet_10.zip › LR-03/64_7.tiff]

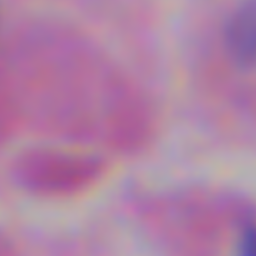

Supplement: Supplementary file 12 [file Data_Sheet_10.zip › LR-03/65_0.tiff]

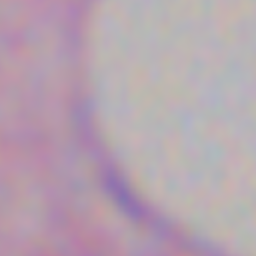

Supplement: Supplementary file 12 [file Data_Sheet_10.zip › LR-03/65_1.tiff]

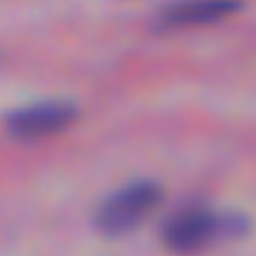

Supplement: Supplementary file 12 [file Data_Sheet_10.zip › LR-03/65_2.tiff]

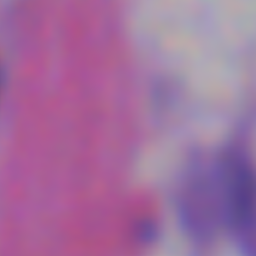

Supplement: Supplementary file 12 [file Data_Sheet_10.zip › LR-03/65_3.tiff]

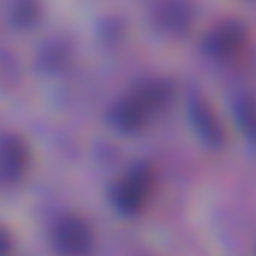

Supplement: Supplementary file 12 [file Data_Sheet_10.zip › LR-03/65_4.tiff]

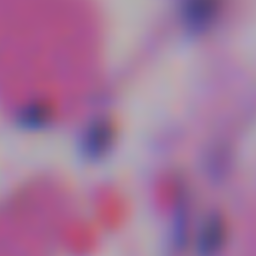

Supplement: Supplementary file 12 [file Data_Sheet_10.zip › LR-03/65_5.tiff]

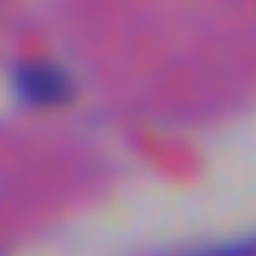

Supplement: Supplementary file 12 [file Data_Sheet_10.zip › LR-03/65_6.tiff]

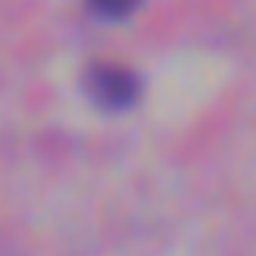

Supplement: Supplementary file 12 [file Data_Sheet_10.zip › LR-03/65_7.tiff]

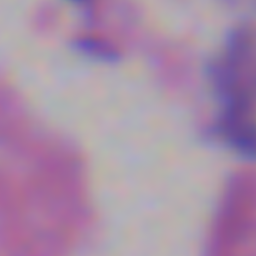

Supplement: Supplementary file 12 [file Data_Sheet_10.zip › LR-03/66_0.tiff]

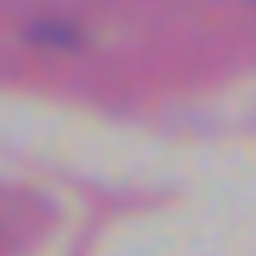

Supplement: Supplementary file 12 [file Data_Sheet_10.zip › LR-03/66_1.tiff]

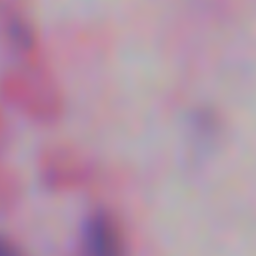

Supplement: Supplementary file 12 [file Data_Sheet_10.zip › LR-03/66_2.tiff]

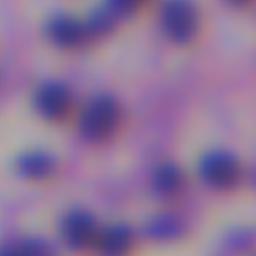

Supplement: Supplementary file 12 [file Data_Sheet_10.zip › LR-03/66_3.tiff]

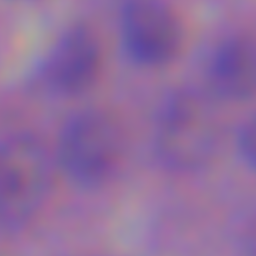

Supplement: Supplementary file 12 [file Data_Sheet_10.zip › LR-03/66_4.tiff]

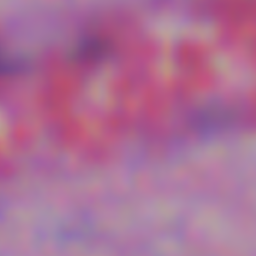

Supplement: Supplementary file 12 [file Data_Sheet_10.zip › LR-03/66_5.tiff]

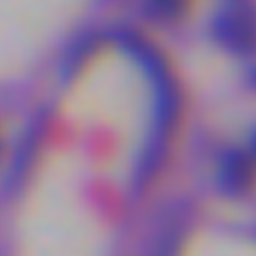

Supplement: Supplementary file 12 [file Data_Sheet_10.zip › LR-03/66_6.tiff]

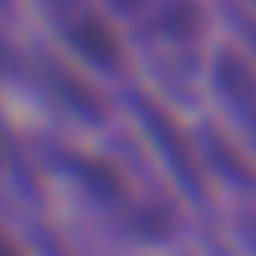

Supplement: Supplementary file 12 [file Data_Sheet_10.zip › LR-03/66_7.tiff]

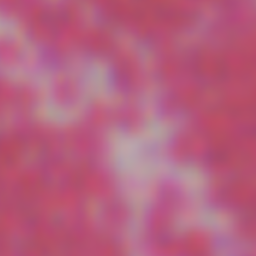

Supplement: Supplementary file 12 [file Data_Sheet_10.zip › LR-03/67_0.tiff]

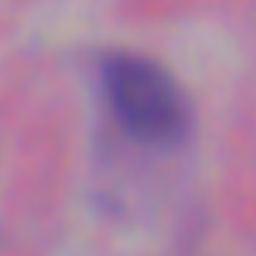

Supplement: Supplementary file 12 [file Data_Sheet_10.zip › LR-03/67_1.tiff]

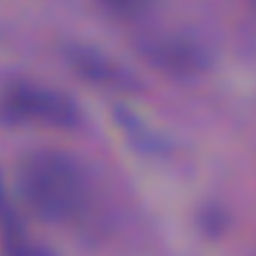

Supplement: Supplementary file 12 [file Data_Sheet_10.zip › LR-03/67_2.tiff]

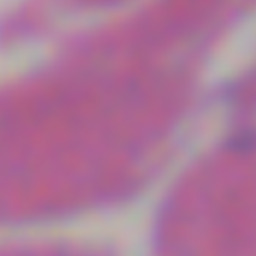

Supplement: Supplementary file 12 [file Data_Sheet_10.zip › LR-03/67_3.tiff]

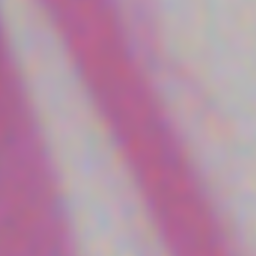

Supplement: Supplementary file 12 [file Data_Sheet_10.zip › LR-03/67_4.tiff]

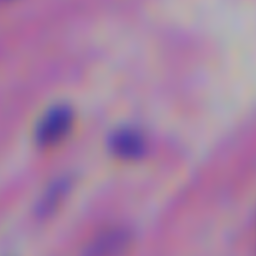

Supplement: Supplementary file 12 [file Data_Sheet_10.zip › LR-03/67_5.tiff]

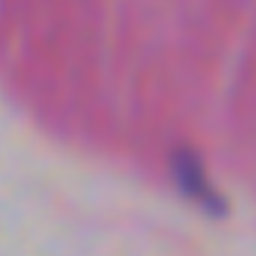

Supplement: Supplementary file 12 [file Data_Sheet_10.zip › LR-03/67_6.tiff]

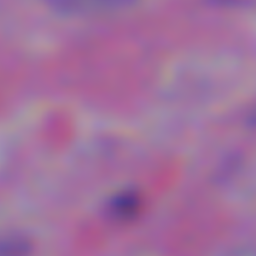

Supplement: Supplementary file 12 [file Data_Sheet_10.zip › LR-03/67_7.tiff]

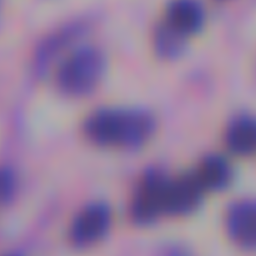

Supplement: Supplementary file 12 [file Data_Sheet_10.zip › LR-03/68_0.tiff]

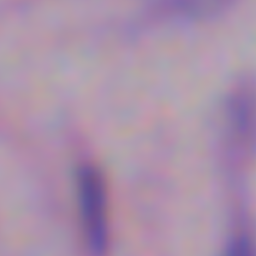

Supplement: Supplementary file 12 [file Data_Sheet_10.zip › LR-03/68_1.tiff]

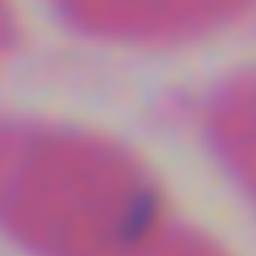

Supplement: Supplementary file 12 [file Data_Sheet_10.zip › LR-03/68_2.tiff]

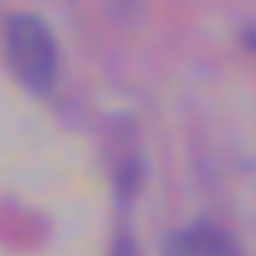

Supplement: Supplementary file 12 [file Data_Sheet_10.zip › LR-03/68_3.tiff]

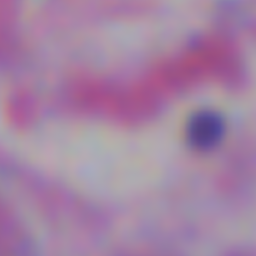

Supplement: Supplementary file 12 [file Data_Sheet_10.zip › LR-03/68_4.tiff]

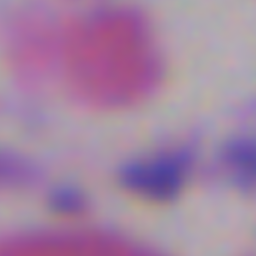

Supplement: Supplementary file 12 [file Data_Sheet_10.zip › LR-03/68_5.tiff]

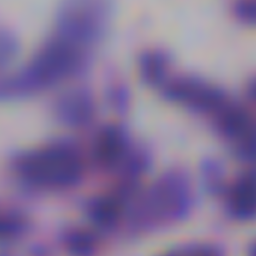

Supplement: Supplementary file 12 [file Data_Sheet_10.zip › LR-03/68_6.tiff]

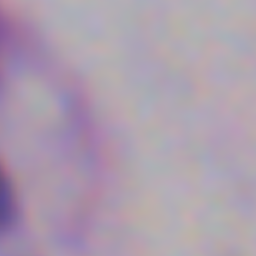

Supplement: Supplementary file 12 [file Data_Sheet_10.zip › LR-03/68_7.tiff]

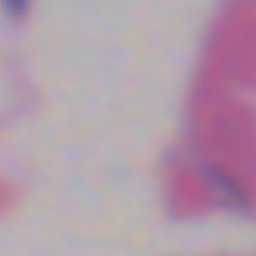

Supplement: Supplementary file 12 [file Data_Sheet_10.zip › LR-03/69_0.tiff]

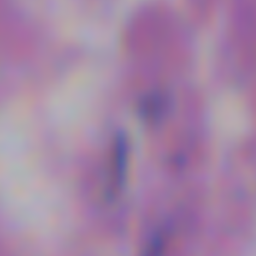

Supplement: Supplementary file 12 [file Data_Sheet_10.zip › LR-03/69_1.tiff]

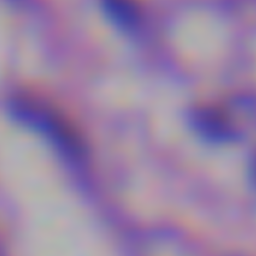

Supplement: Supplementary file 12 [file Data_Sheet_10.zip › LR-03/69_2.tiff]

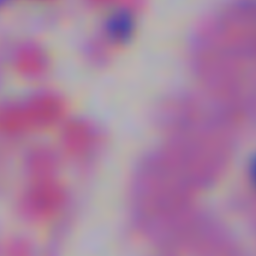

Supplement: Supplementary file 12 [file Data_Sheet_10.zip › LR-03/69_3.tiff]

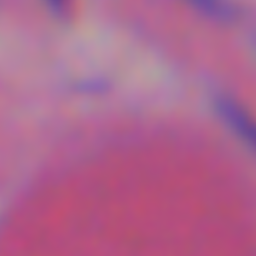

Supplement: Supplementary file 12 [file Data_Sheet_10.zip › LR-03/69_4.tiff]

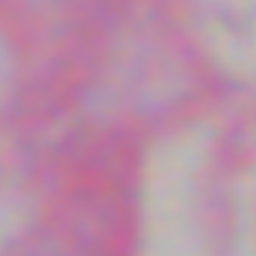

Supplement: Supplementary file 12 [file Data_Sheet_10.zip › LR-03/69_5.tiff]

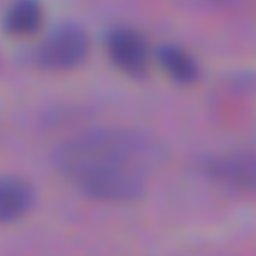

Supplement: Supplementary file 12 [file Data_Sheet_10.zip › LR-03/69_6.tiff]

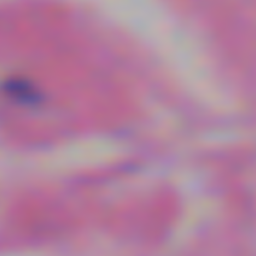

Supplement: Supplementary file 12 [file Data_Sheet_10.zip › LR-03/69_7.tiff]

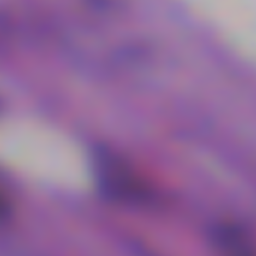

Supplement: Supplementary file 12 [file Data_Sheet_10.zip › LR-03/70_0.tiff]

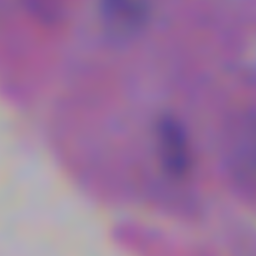

Supplement: Supplementary file 12 [file Data_Sheet_10.zip › LR-03/70_1.tiff]

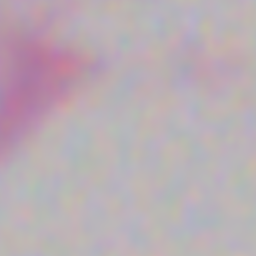

Supplement: Supplementary file 12 [file Data_Sheet_10.zip › LR-03/70_2.tiff]

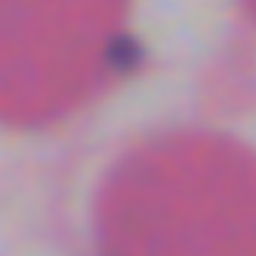

Supplement: Supplementary file 12 [file Data_Sheet_10.zip › LR-03/70_3.tiff]

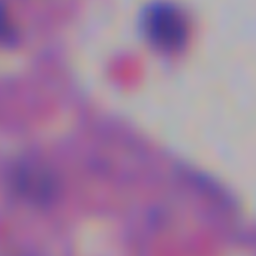

Supplement: Supplementary file 12 [file Data_Sheet_10.zip › LR-03/70_4.tiff]

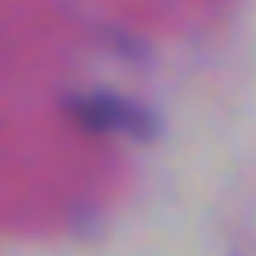

Supplement: Supplementary file 12 [file Data_Sheet_10.zip › LR-03/70_5.tiff]

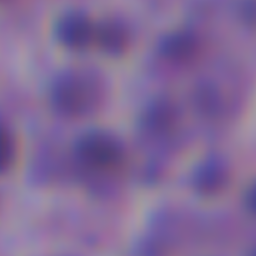

Supplement: Supplementary file 12 [file Data_Sheet_10.zip › LR-03/70_6.tiff]

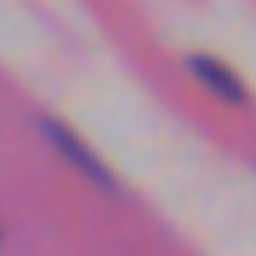

Supplement: Supplementary file 12 [file Data_Sheet_10.zip › LR-03/70_7.tiff]

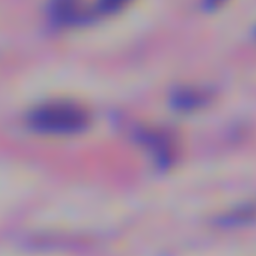

Supplement: Supplementary file 12 [file Data_Sheet_10.zip › LR-03/71_0.tiff]

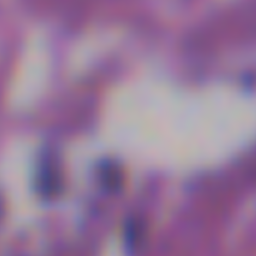

Supplement: Supplementary file 12 [file Data_Sheet_10.zip › LR-03/71_1.tiff]

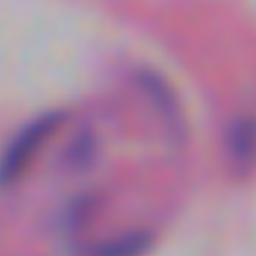

Supplement: Supplementary file 12 [file Data_Sheet_10.zip › LR-03/71_2.tiff]

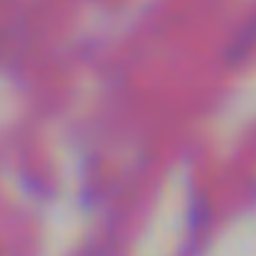

Supplement: Supplementary file 12 [file Data_Sheet_10.zip › LR-03/71_3.tiff]

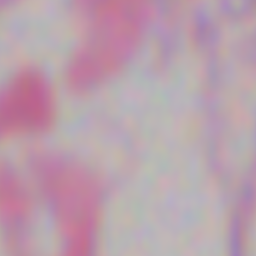

Supplement: Supplementary file 12 [file Data_Sheet_10.zip › LR-03/71_4.tiff]

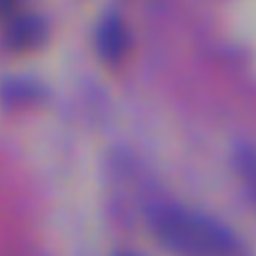

Supplement: Supplementary file 12 [file Data_Sheet_10.zip › LR-03/71_5.tiff]
