# Supplementary figures and images for: Feasibility study of single-image super-resolution scanning system based on deep learning for pathological diagnosis of oral epithelial dysplasia (part 16 of 21)
Source: Front Med (Lausanne). 2025 Mar 12;12:1550512. doi: 10.3389/fmed.2025.1550512 (PMC11936936; doi:10.3389/fmed.2025.1550512)

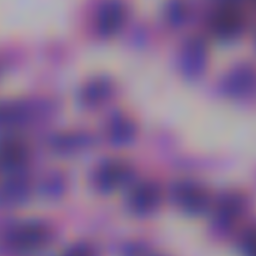

Supplement: Supplementary file 13 [file Data_Sheet_11.zip › LR-04/88_6.tiff]

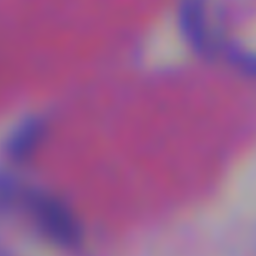

Supplement: Supplementary file 13 [file Data_Sheet_11.zip › LR-04/88_7.tiff]

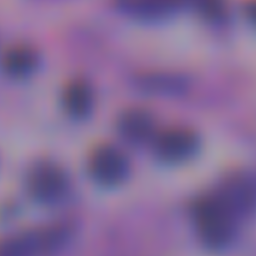

Supplement: Supplementary file 13 [file Data_Sheet_11.zip › LR-04/89_0.tiff]

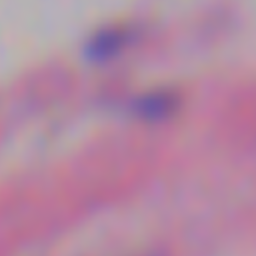

Supplement: Supplementary file 13 [file Data_Sheet_11.zip › LR-04/89_1.tiff]

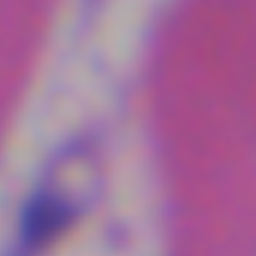

Supplement: Supplementary file 13 [file Data_Sheet_11.zip › LR-04/89_2.tiff]

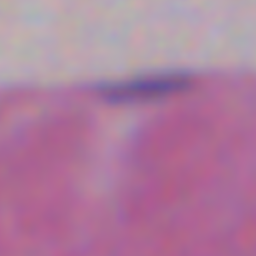

Supplement: Supplementary file 13 [file Data_Sheet_11.zip › LR-04/89_3.tiff]

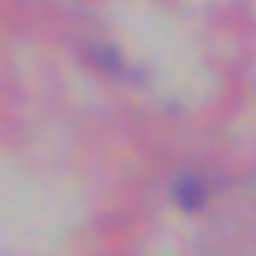

Supplement: Supplementary file 13 [file Data_Sheet_11.zip › LR-04/89_4.tiff]

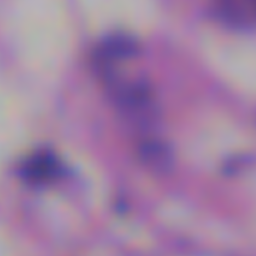

Supplement: Supplementary file 13 [file Data_Sheet_11.zip › LR-04/89_5.tiff]

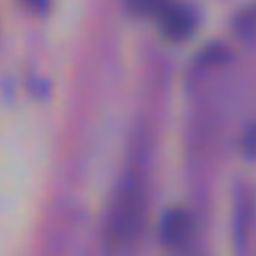

Supplement: Supplementary file 13 [file Data_Sheet_11.zip › LR-04/89_6.tiff]

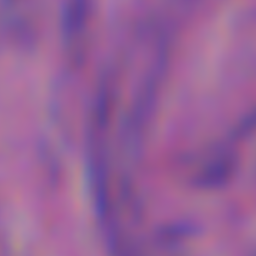

Supplement: Supplementary file 13 [file Data_Sheet_11.zip › LR-04/89_7.tiff]

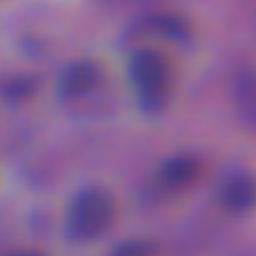

Supplement: Supplementary file 13 [file Data_Sheet_11.zip › LR-04/90_0.tiff]

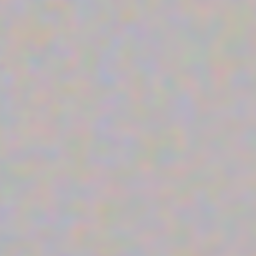

Supplement: Supplementary file 13 [file Data_Sheet_11.zip › LR-04/90_1.tiff]

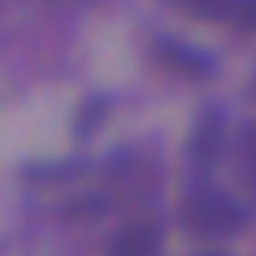

Supplement: Supplementary file 13 [file Data_Sheet_11.zip › LR-04/90_2.tiff]

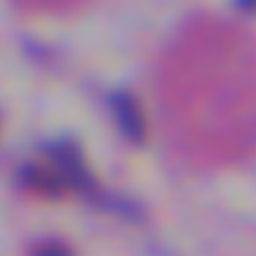

Supplement: Supplementary file 13 [file Data_Sheet_11.zip › LR-04/90_3.tiff]

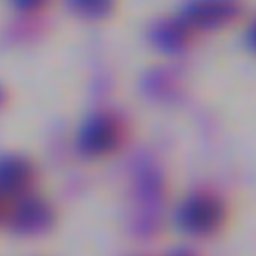

Supplement: Supplementary file 13 [file Data_Sheet_11.zip › LR-04/90_4.tiff]

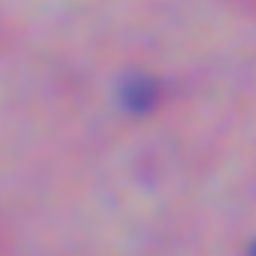

Supplement: Supplementary file 13 [file Data_Sheet_11.zip › LR-04/90_5.tiff]

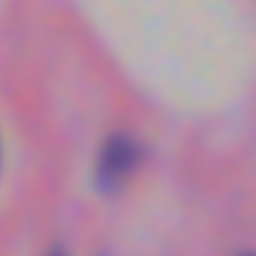

Supplement: Supplementary file 13 [file Data_Sheet_11.zip › LR-04/90_6.tiff]

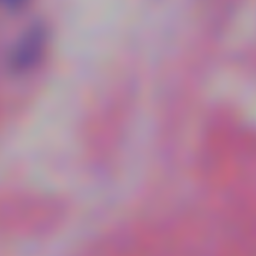

Supplement: Supplementary file 13 [file Data_Sheet_11.zip › LR-04/90_7.tiff]

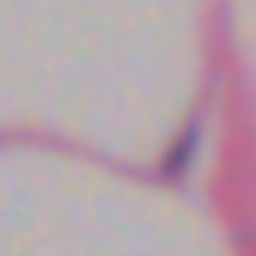

Supplement: Supplementary file 13 [file Data_Sheet_11.zip › LR-04/91_0.tiff]

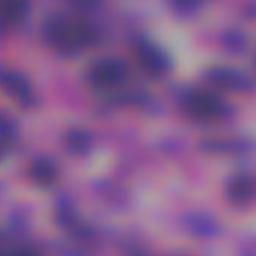

Supplement: Supplementary file 13 [file Data_Sheet_11.zip › LR-04/91_1.tiff]

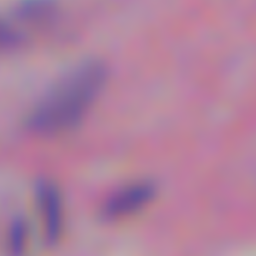

Supplement: Supplementary file 13 [file Data_Sheet_11.zip › LR-04/91_2.tiff]

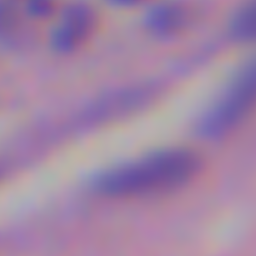

Supplement: Supplementary file 13 [file Data_Sheet_11.zip › LR-04/91_3.tiff]

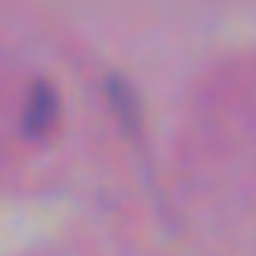

Supplement: Supplementary file 13 [file Data_Sheet_11.zip › LR-04/91_4.tiff]

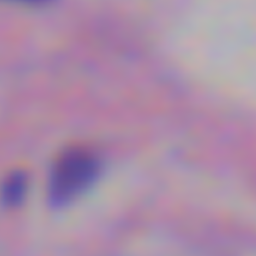

Supplement: Supplementary file 13 [file Data_Sheet_11.zip › LR-04/91_5.tiff]

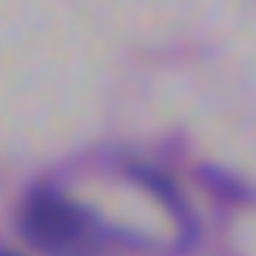

Supplement: Supplementary file 13 [file Data_Sheet_11.zip › LR-04/91_6.tiff]

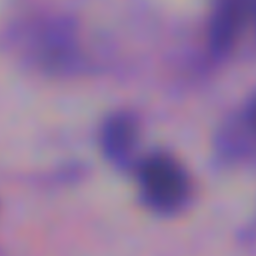

Supplement: Supplementary file 13 [file Data_Sheet_11.zip › LR-04/91_7.tiff]

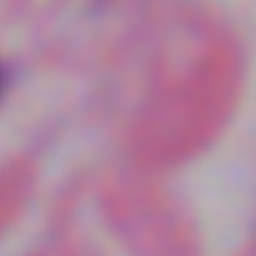

Supplement: Supplementary file 13 [file Data_Sheet_11.zip › LR-04/92_0.tiff]

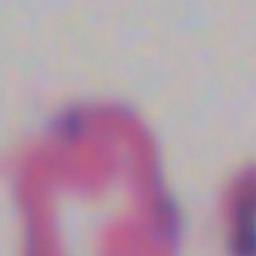

Supplement: Supplementary file 13 [file Data_Sheet_11.zip › LR-04/92_1.tiff]

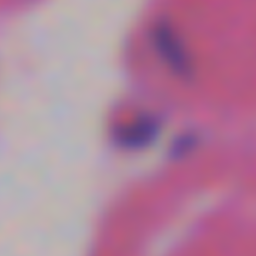

Supplement: Supplementary file 13 [file Data_Sheet_11.zip › LR-04/92_2.tiff]

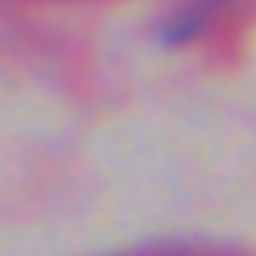

Supplement: Supplementary file 13 [file Data_Sheet_11.zip › LR-04/92_3.tiff]

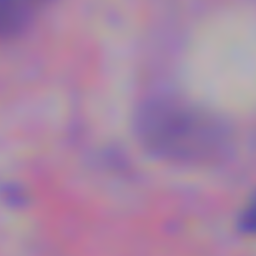

Supplement: Supplementary file 13 [file Data_Sheet_11.zip › LR-04/92_4.tiff]

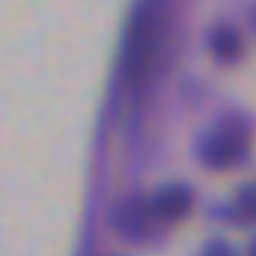

Supplement: Supplementary file 13 [file Data_Sheet_11.zip › LR-04/92_5.tiff]

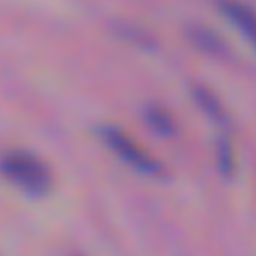

Supplement: Supplementary file 13 [file Data_Sheet_11.zip › LR-04/92_6.tiff]

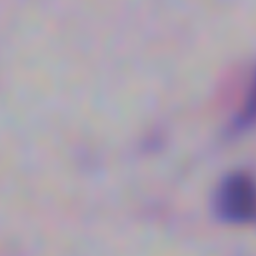

Supplement: Supplementary file 13 [file Data_Sheet_11.zip › LR-04/92_7.tiff]

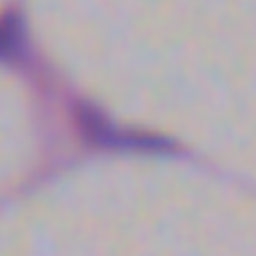

Supplement: Supplementary file 13 [file Data_Sheet_11.zip › LR-04/93_0.tiff]

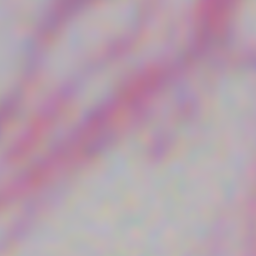

Supplement: Supplementary file 13 [file Data_Sheet_11.zip › LR-04/93_1.tiff]

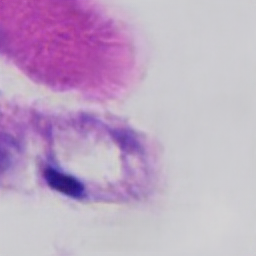

Supplement: Supplementary file 14 [file Data_Sheet_12.zip › SR-01/0_0.tiff]

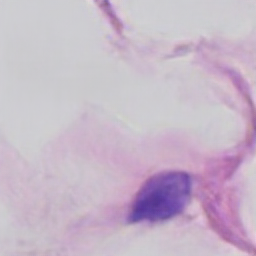

Supplement: Supplementary file 14 [file Data_Sheet_12.zip › SR-01/0_1.tiff]

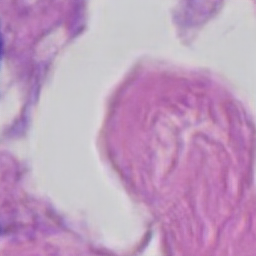

Supplement: Supplementary file 14 [file Data_Sheet_12.zip › SR-01/0_2.tiff]

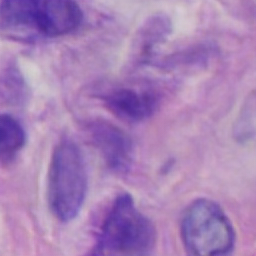

Supplement: Supplementary file 14 [file Data_Sheet_12.zip › SR-01/0_3.tiff]

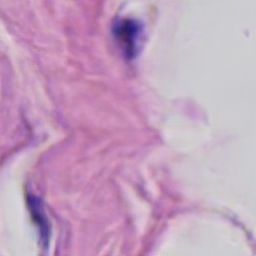

Supplement: Supplementary file 14 [file Data_Sheet_12.zip › SR-01/0_4.tiff]

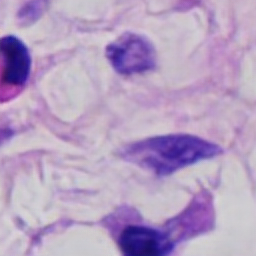

Supplement: Supplementary file 14 [file Data_Sheet_12.zip › SR-01/0_5.tiff]

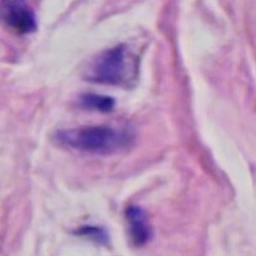

Supplement: Supplementary file 14 [file Data_Sheet_12.zip › SR-01/0_6.tiff]

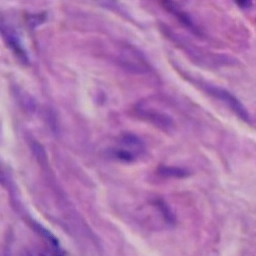

Supplement: Supplementary file 14 [file Data_Sheet_12.zip › SR-01/0_7.tiff]

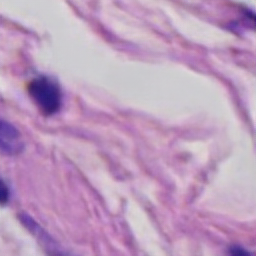

Supplement: Supplementary file 14 [file Data_Sheet_12.zip › SR-01/10_0.tiff]

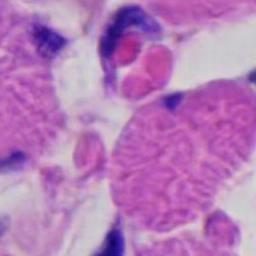

Supplement: Supplementary file 14 [file Data_Sheet_12.zip › SR-01/10_1.tiff]

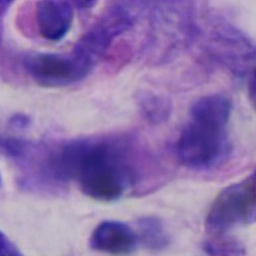

Supplement: Supplementary file 14 [file Data_Sheet_12.zip › SR-01/10_2.tiff]

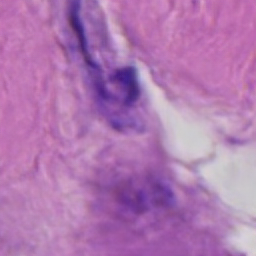

Supplement: Supplementary file 14 [file Data_Sheet_12.zip › SR-01/10_3.tiff]

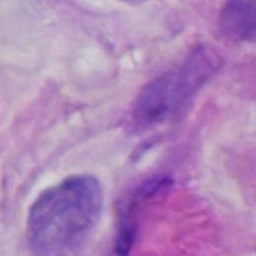

Supplement: Supplementary file 14 [file Data_Sheet_12.zip › SR-01/10_4.tiff]

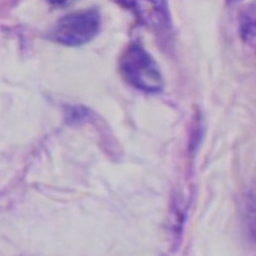

Supplement: Supplementary file 14 [file Data_Sheet_12.zip › SR-01/10_5.tiff]

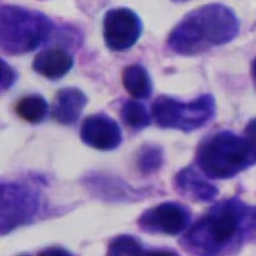

Supplement: Supplementary file 14 [file Data_Sheet_12.zip › SR-01/10_6.tiff]

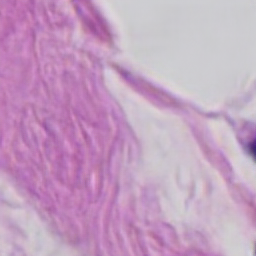

Supplement: Supplementary file 14 [file Data_Sheet_12.zip › SR-01/10_7.tiff]

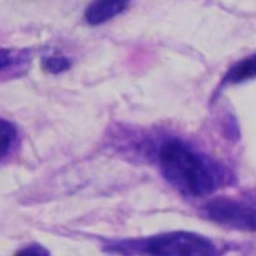

Supplement: Supplementary file 14 [file Data_Sheet_12.zip › SR-01/11_0.tiff]

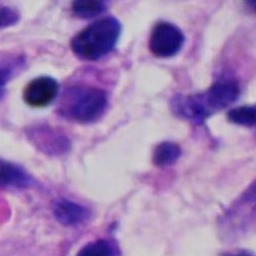

Supplement: Supplementary file 14 [file Data_Sheet_12.zip › SR-01/11_1.tiff]

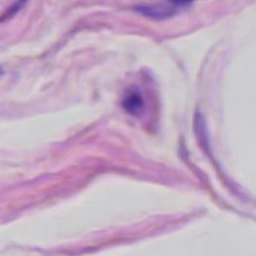

Supplement: Supplementary file 14 [file Data_Sheet_12.zip › SR-01/11_2.tiff]

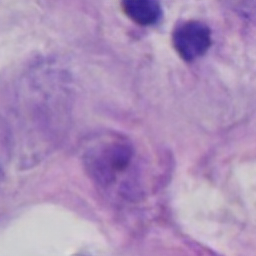

Supplement: Supplementary file 14 [file Data_Sheet_12.zip › SR-01/11_3.tiff]

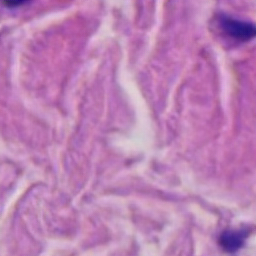

Supplement: Supplementary file 14 [file Data_Sheet_12.zip › SR-01/11_4.tiff]

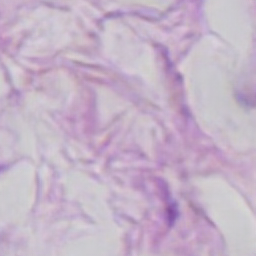

Supplement: Supplementary file 14 [file Data_Sheet_12.zip › SR-01/11_5.tiff]

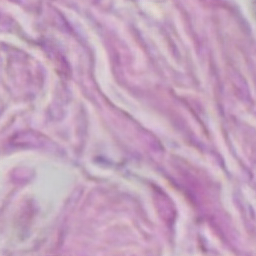

Supplement: Supplementary file 14 [file Data_Sheet_12.zip › SR-01/11_6.tiff]

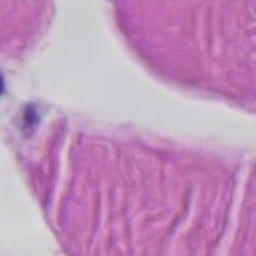

Supplement: Supplementary file 14 [file Data_Sheet_12.zip › SR-01/11_7.tiff]

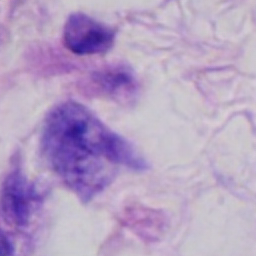

Supplement: Supplementary file 14 [file Data_Sheet_12.zip › SR-01/12_0.tiff]

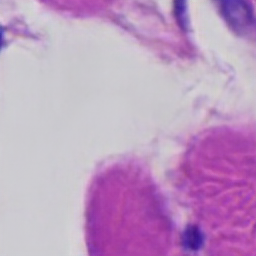

Supplement: Supplementary file 14 [file Data_Sheet_12.zip › SR-01/12_1.tiff]

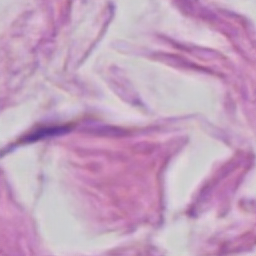

Supplement: Supplementary file 14 [file Data_Sheet_12.zip › SR-01/12_2.tiff]

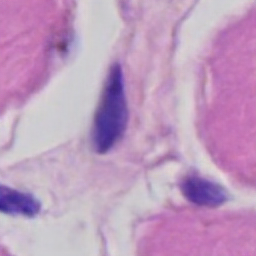

Supplement: Supplementary file 14 [file Data_Sheet_12.zip › SR-01/12_3.tiff]

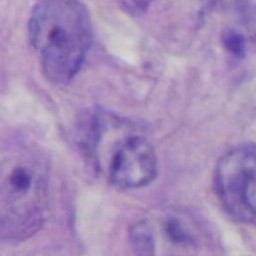

Supplement: Supplementary file 14 [file Data_Sheet_12.zip › SR-01/12_4.tiff]

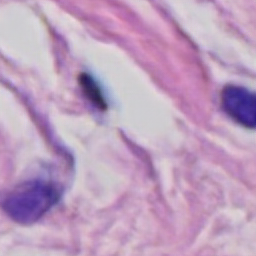

Supplement: Supplementary file 14 [file Data_Sheet_12.zip › SR-01/12_5.tiff]

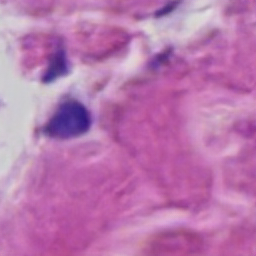

Supplement: Supplementary file 14 [file Data_Sheet_12.zip › SR-01/12_6.tiff]

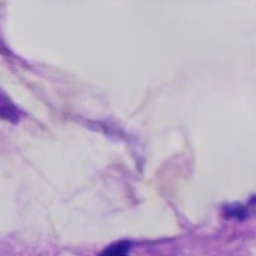

Supplement: Supplementary file 14 [file Data_Sheet_12.zip › SR-01/12_7.tiff]

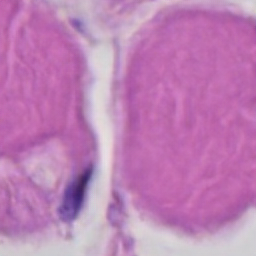

Supplement: Supplementary file 14 [file Data_Sheet_12.zip › SR-01/13_0.tiff]

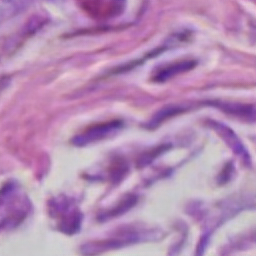

Supplement: Supplementary file 14 [file Data_Sheet_12.zip › SR-01/13_1.tiff]

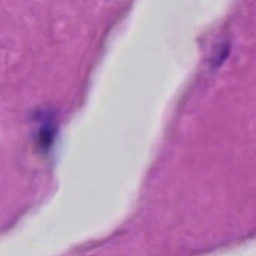

Supplement: Supplementary file 14 [file Data_Sheet_12.zip › SR-01/13_2.tiff]

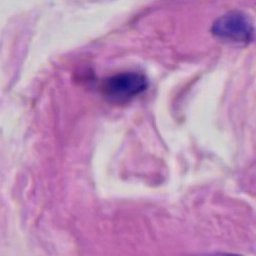

Supplement: Supplementary file 14 [file Data_Sheet_12.zip › SR-01/13_3.tiff]

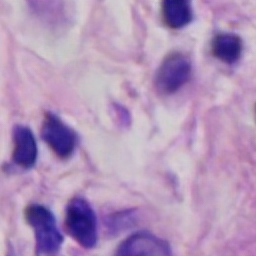

Supplement: Supplementary file 14 [file Data_Sheet_12.zip › SR-01/13_4.tiff]

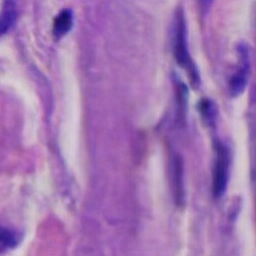

Supplement: Supplementary file 14 [file Data_Sheet_12.zip › SR-01/13_5.tiff]

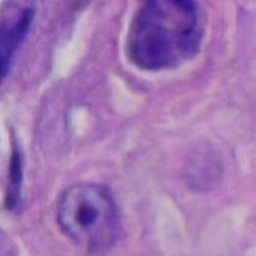

Supplement: Supplementary file 14 [file Data_Sheet_12.zip › SR-01/13_6.tiff]

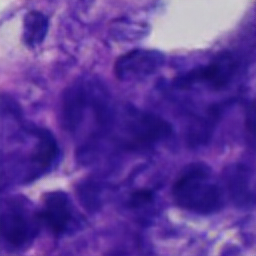

Supplement: Supplementary file 14 [file Data_Sheet_12.zip › SR-01/13_7.tiff]

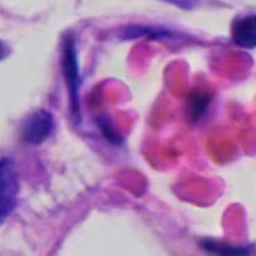

Supplement: Supplementary file 14 [file Data_Sheet_12.zip › SR-01/14_0.tiff]

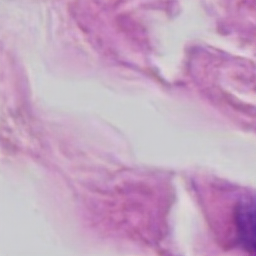

Supplement: Supplementary file 14 [file Data_Sheet_12.zip › SR-01/14_1.tiff]

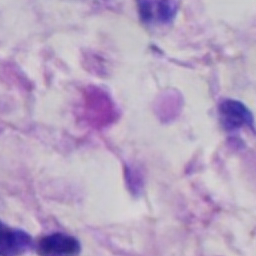

Supplement: Supplementary file 14 [file Data_Sheet_12.zip › SR-01/14_2.tiff]

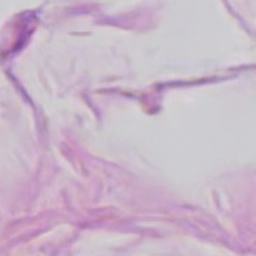

Supplement: Supplementary file 14 [file Data_Sheet_12.zip › SR-01/14_3.tiff]

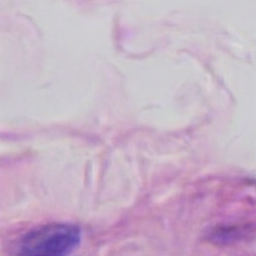

Supplement: Supplementary file 14 [file Data_Sheet_12.zip › SR-01/14_4.tiff]

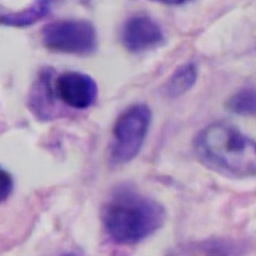

Supplement: Supplementary file 14 [file Data_Sheet_12.zip › SR-01/14_5.tiff]

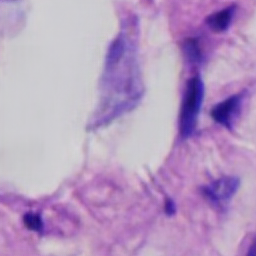

Supplement: Supplementary file 14 [file Data_Sheet_12.zip › SR-01/14_6.tiff]

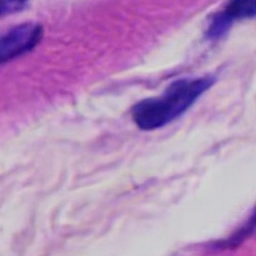

Supplement: Supplementary file 14 [file Data_Sheet_12.zip › SR-01/14_7.tiff]

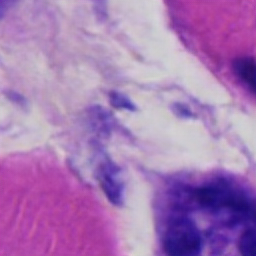

Supplement: Supplementary file 14 [file Data_Sheet_12.zip › SR-01/15_0.tiff]

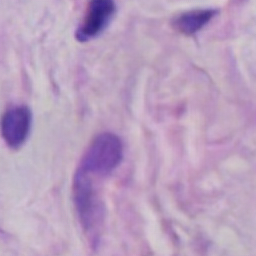

Supplement: Supplementary file 14 [file Data_Sheet_12.zip › SR-01/15_1.tiff]

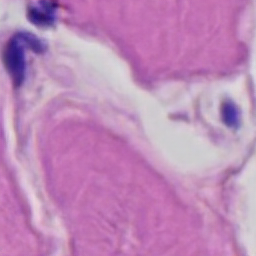

Supplement: Supplementary file 14 [file Data_Sheet_12.zip › SR-01/15_2.tiff]

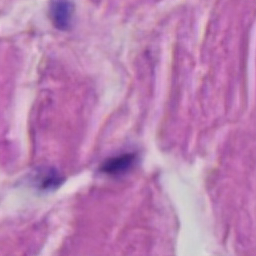

Supplement: Supplementary file 14 [file Data_Sheet_12.zip › SR-01/15_3.tiff]

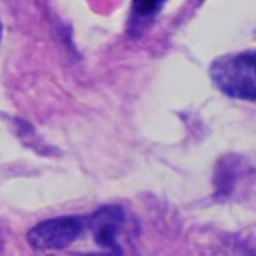

Supplement: Supplementary file 14 [file Data_Sheet_12.zip › SR-01/15_4.tiff]

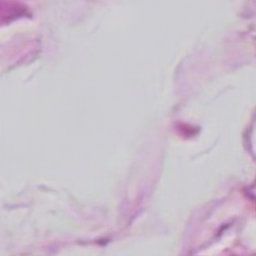

Supplement: Supplementary file 14 [file Data_Sheet_12.zip › SR-01/15_5.tiff]

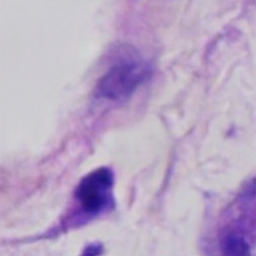

Supplement: Supplementary file 14 [file Data_Sheet_12.zip › SR-01/15_6.tiff]

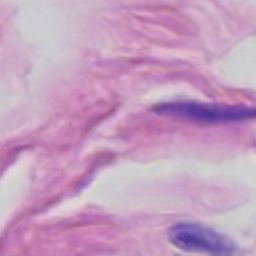

Supplement: Supplementary file 14 [file Data_Sheet_12.zip › SR-01/15_7.tiff]

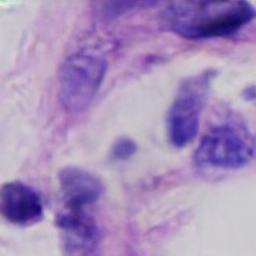

Supplement: Supplementary file 14 [file Data_Sheet_12.zip › SR-01/16_0.tiff]

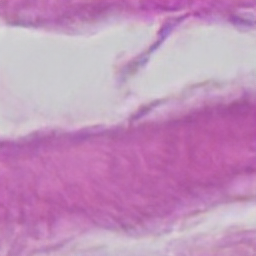

Supplement: Supplementary file 14 [file Data_Sheet_12.zip › SR-01/16_1.tiff]

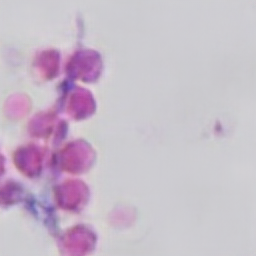

Supplement: Supplementary file 14 [file Data_Sheet_12.zip › SR-01/16_2.tiff]

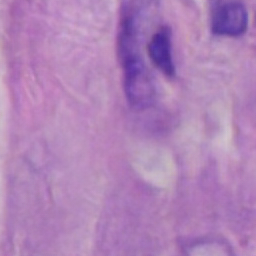

Supplement: Supplementary file 14 [file Data_Sheet_12.zip › SR-01/16_3.tiff]

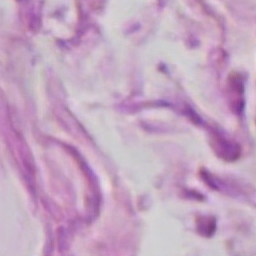

Supplement: Supplementary file 14 [file Data_Sheet_12.zip › SR-01/16_4.tiff]

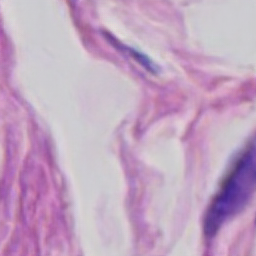

Supplement: Supplementary file 14 [file Data_Sheet_12.zip › SR-01/16_5.tiff]

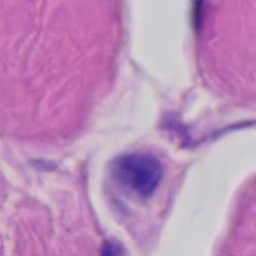

Supplement: Supplementary file 14 [file Data_Sheet_12.zip › SR-01/16_6.tiff]

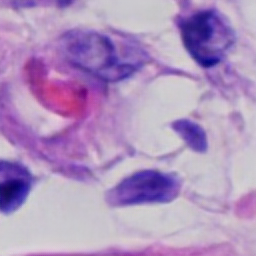

Supplement: Supplementary file 14 [file Data_Sheet_12.zip › SR-01/16_7.tiff]
