# Supplementary figures and images for: Feasibility study of single-image super-resolution scanning system based on deep learning for pathological diagnosis of oral epithelial dysplasia (part 18 of 21)
Source: Front Med (Lausanne). 2025 Mar 12;12:1550512. doi: 10.3389/fmed.2025.1550512 (PMC11936936; doi:10.3389/fmed.2025.1550512)

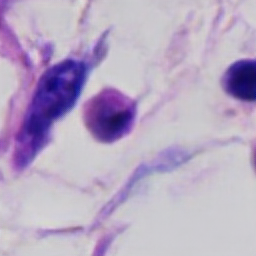

Supplement: Supplementary file 14 [file Data_Sheet_12.zip › SR-01/9_4.tiff]

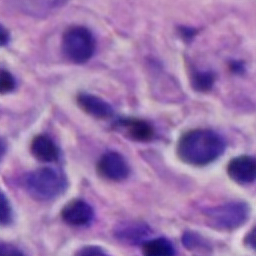

Supplement: Supplementary file 14 [file Data_Sheet_12.zip › SR-01/9_5.tiff]

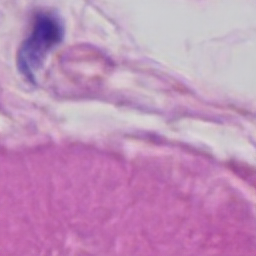

Supplement: Supplementary file 14 [file Data_Sheet_12.zip › SR-01/9_6.tiff]

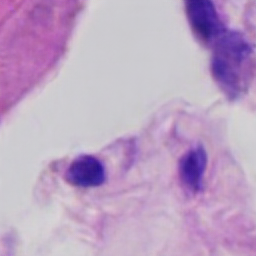

Supplement: Supplementary file 14 [file Data_Sheet_12.zip › SR-01/9_7.tiff]

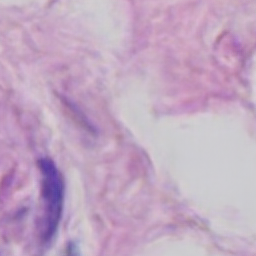

Supplement: Supplementary file 15 [file Data_Sheet_13.zip › SR-02/21_0.tiff]

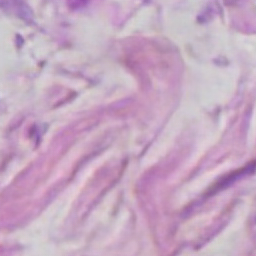

Supplement: Supplementary file 15 [file Data_Sheet_13.zip › SR-02/21_1.tiff]

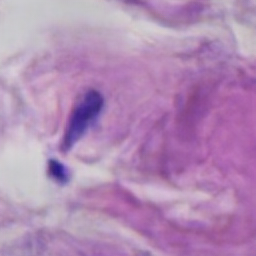

Supplement: Supplementary file 15 [file Data_Sheet_13.zip › SR-02/21_2.tiff]

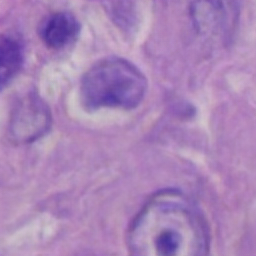

Supplement: Supplementary file 15 [file Data_Sheet_13.zip › SR-02/21_3.tiff]

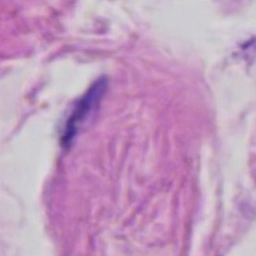

Supplement: Supplementary file 15 [file Data_Sheet_13.zip › SR-02/21_4.tiff]

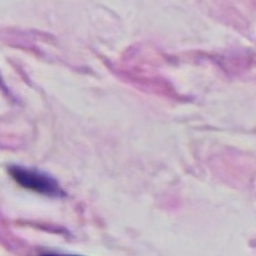

Supplement: Supplementary file 15 [file Data_Sheet_13.zip › SR-02/21_5.tiff]

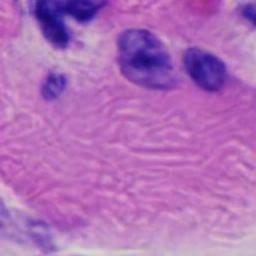

Supplement: Supplementary file 15 [file Data_Sheet_13.zip › SR-02/21_6.tiff]

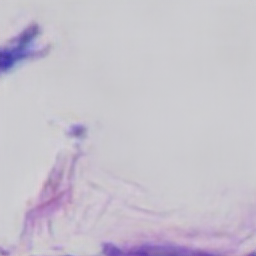

Supplement: Supplementary file 15 [file Data_Sheet_13.zip › SR-02/21_7.tiff]

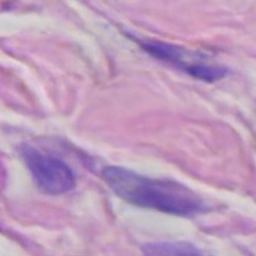

Supplement: Supplementary file 15 [file Data_Sheet_13.zip › SR-02/22_0.tiff]

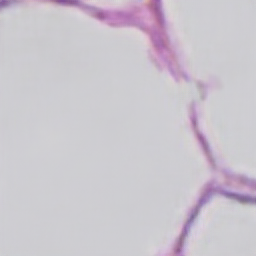

Supplement: Supplementary file 15 [file Data_Sheet_13.zip › SR-02/22_1.tiff]

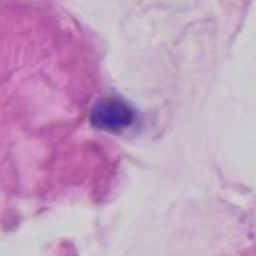

Supplement: Supplementary file 15 [file Data_Sheet_13.zip › SR-02/22_2.tiff]

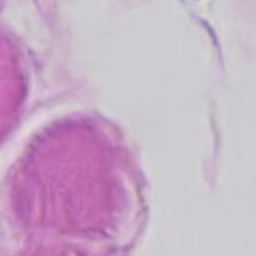

Supplement: Supplementary file 15 [file Data_Sheet_13.zip › SR-02/22_3.tiff]

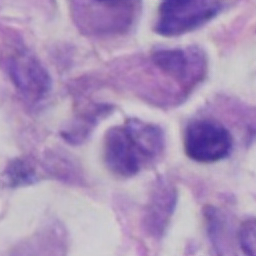

Supplement: Supplementary file 15 [file Data_Sheet_13.zip › SR-02/22_4.tiff]

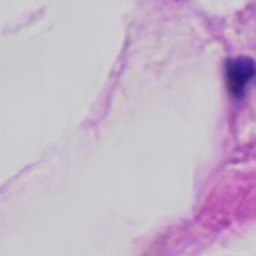

Supplement: Supplementary file 15 [file Data_Sheet_13.zip › SR-02/22_5.tiff]

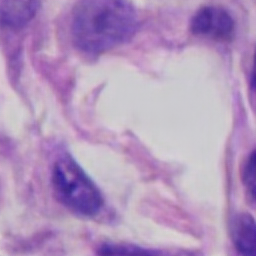

Supplement: Supplementary file 15 [file Data_Sheet_13.zip › SR-02/22_6.tiff]

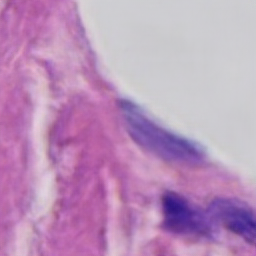

Supplement: Supplementary file 15 [file Data_Sheet_13.zip › SR-02/22_7.tiff]

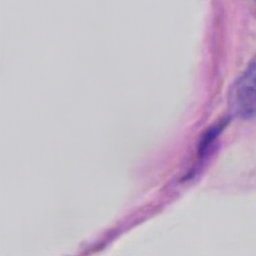

Supplement: Supplementary file 15 [file Data_Sheet_13.zip › SR-02/23_0.tiff]

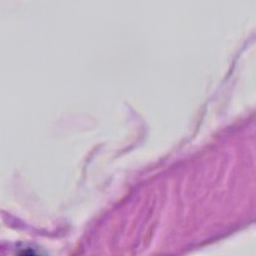

Supplement: Supplementary file 15 [file Data_Sheet_13.zip › SR-02/23_1.tiff]

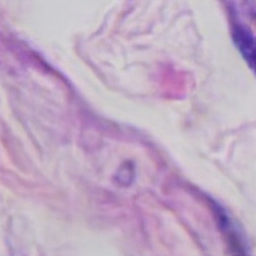

Supplement: Supplementary file 15 [file Data_Sheet_13.zip › SR-02/23_2.tiff]

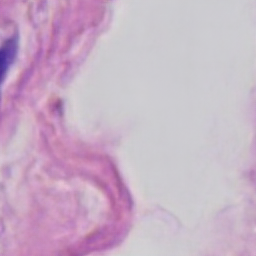

Supplement: Supplementary file 15 [file Data_Sheet_13.zip › SR-02/23_3.tiff]

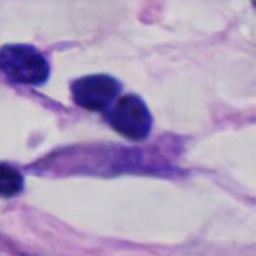

Supplement: Supplementary file 15 [file Data_Sheet_13.zip › SR-02/23_4.tiff]

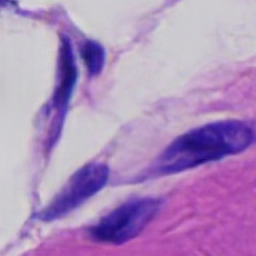

Supplement: Supplementary file 15 [file Data_Sheet_13.zip › SR-02/23_5.tiff]

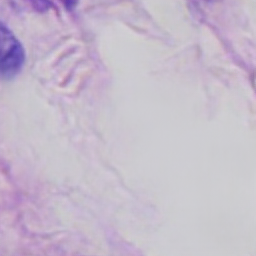

Supplement: Supplementary file 15 [file Data_Sheet_13.zip › SR-02/23_6.tiff]

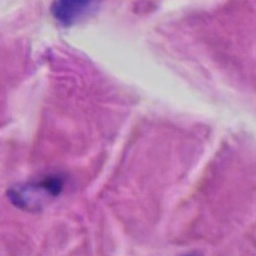

Supplement: Supplementary file 15 [file Data_Sheet_13.zip › SR-02/23_7.tiff]

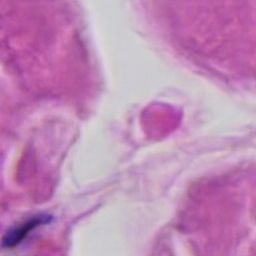

Supplement: Supplementary file 15 [file Data_Sheet_13.zip › SR-02/24_0.tiff]

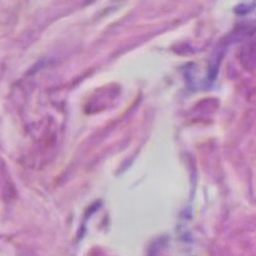

Supplement: Supplementary file 15 [file Data_Sheet_13.zip › SR-02/24_1.tiff]

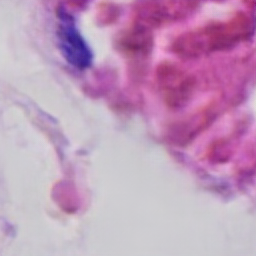

Supplement: Supplementary file 15 [file Data_Sheet_13.zip › SR-02/24_2.tiff]

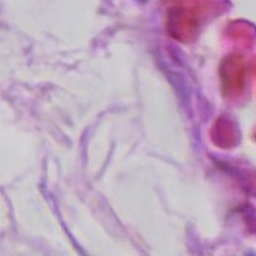

Supplement: Supplementary file 15 [file Data_Sheet_13.zip › SR-02/24_3.tiff]

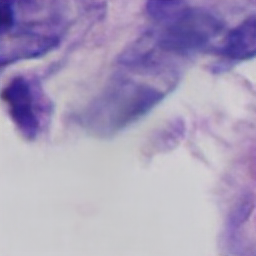

Supplement: Supplementary file 15 [file Data_Sheet_13.zip › SR-02/24_4.tiff]

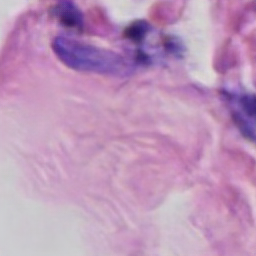

Supplement: Supplementary file 15 [file Data_Sheet_13.zip › SR-02/24_5.tiff]

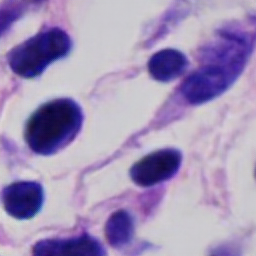

Supplement: Supplementary file 15 [file Data_Sheet_13.zip › SR-02/24_6.tiff]

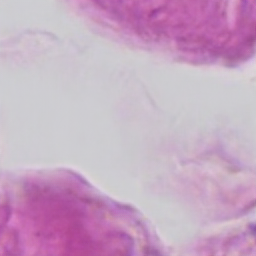

Supplement: Supplementary file 15 [file Data_Sheet_13.zip › SR-02/24_7.tiff]

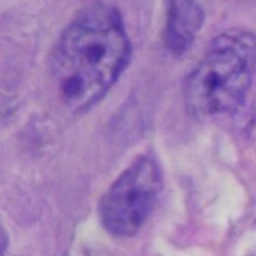

Supplement: Supplementary file 15 [file Data_Sheet_13.zip › SR-02/25_0.tiff]

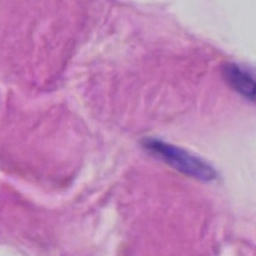

Supplement: Supplementary file 15 [file Data_Sheet_13.zip › SR-02/25_1.tiff]

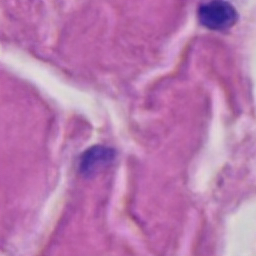

Supplement: Supplementary file 15 [file Data_Sheet_13.zip › SR-02/25_2.tiff]

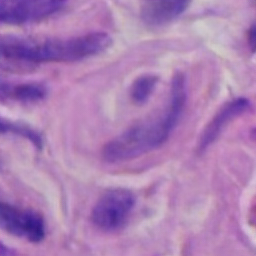

Supplement: Supplementary file 15 [file Data_Sheet_13.zip › SR-02/25_3.tiff]

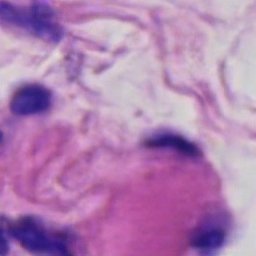

Supplement: Supplementary file 15 [file Data_Sheet_13.zip › SR-02/25_4.tiff]

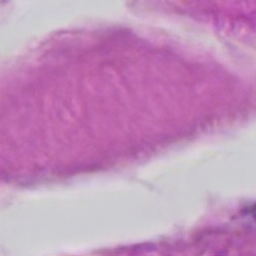

Supplement: Supplementary file 15 [file Data_Sheet_13.zip › SR-02/25_5.tiff]

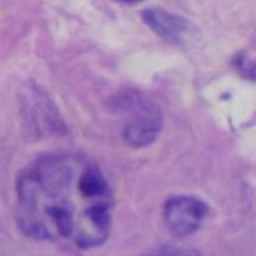

Supplement: Supplementary file 15 [file Data_Sheet_13.zip › SR-02/25_6.tiff]

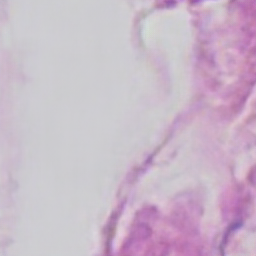

Supplement: Supplementary file 15 [file Data_Sheet_13.zip › SR-02/25_7.tiff]

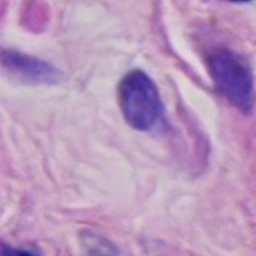

Supplement: Supplementary file 15 [file Data_Sheet_13.zip › SR-02/26_0.tiff]

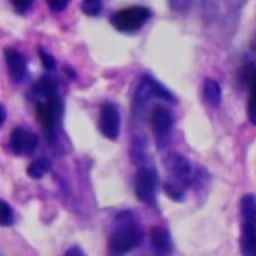

Supplement: Supplementary file 15 [file Data_Sheet_13.zip › SR-02/26_1.tiff]

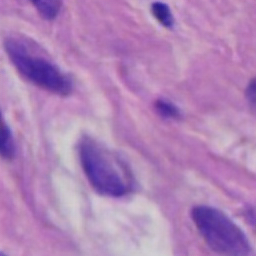

Supplement: Supplementary file 15 [file Data_Sheet_13.zip › SR-02/26_2.tiff]

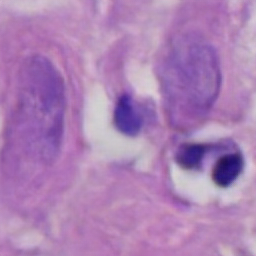

Supplement: Supplementary file 15 [file Data_Sheet_13.zip › SR-02/26_3.tiff]

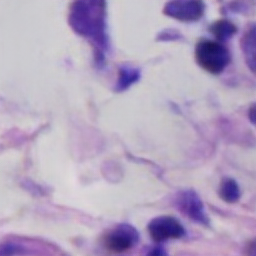

Supplement: Supplementary file 15 [file Data_Sheet_13.zip › SR-02/26_4.tiff]

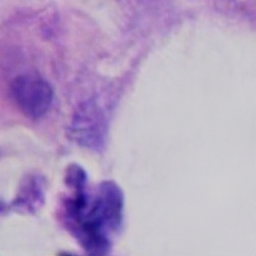

Supplement: Supplementary file 15 [file Data_Sheet_13.zip › SR-02/26_5.tiff]

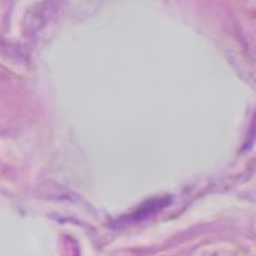

Supplement: Supplementary file 15 [file Data_Sheet_13.zip › SR-02/26_6.tiff]

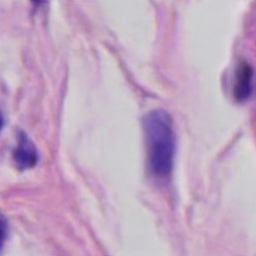

Supplement: Supplementary file 15 [file Data_Sheet_13.zip › SR-02/26_7.tiff]

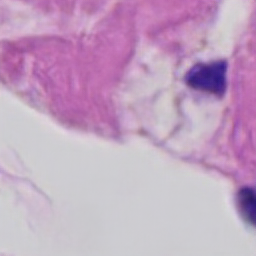

Supplement: Supplementary file 15 [file Data_Sheet_13.zip › SR-02/27_0.tiff]

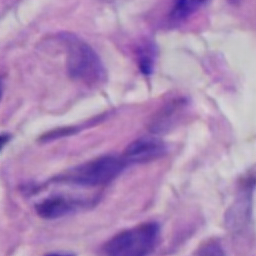

Supplement: Supplementary file 15 [file Data_Sheet_13.zip › SR-02/27_1.tiff]

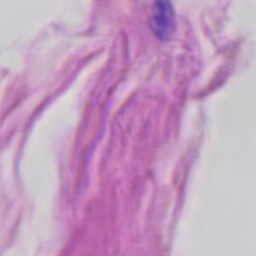

Supplement: Supplementary file 15 [file Data_Sheet_13.zip › SR-02/27_2.tiff]

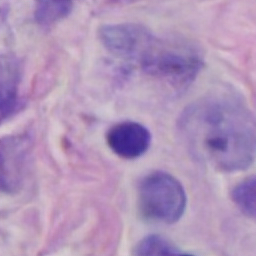

Supplement: Supplementary file 15 [file Data_Sheet_13.zip › SR-02/27_3.tiff]

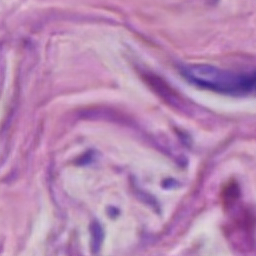

Supplement: Supplementary file 15 [file Data_Sheet_13.zip › SR-02/27_4.tiff]

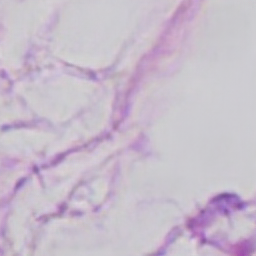

Supplement: Supplementary file 15 [file Data_Sheet_13.zip › SR-02/27_5.tiff]

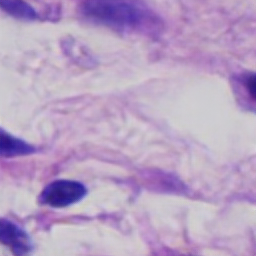

Supplement: Supplementary file 15 [file Data_Sheet_13.zip › SR-02/27_6.tiff]

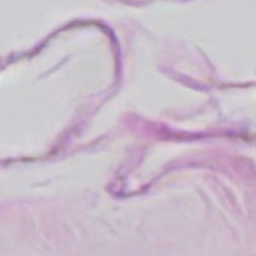

Supplement: Supplementary file 15 [file Data_Sheet_13.zip › SR-02/27_7.tiff]

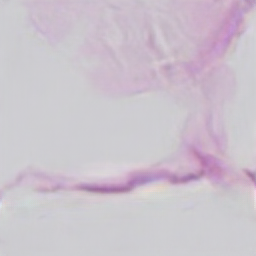

Supplement: Supplementary file 15 [file Data_Sheet_13.zip › SR-02/28_0.tiff]

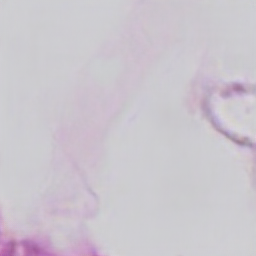

Supplement: Supplementary file 15 [file Data_Sheet_13.zip › SR-02/28_1.tiff]

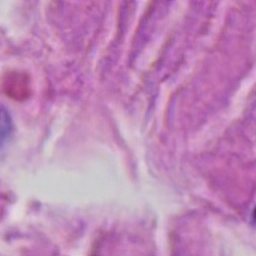

Supplement: Supplementary file 15 [file Data_Sheet_13.zip › SR-02/28_2.tiff]

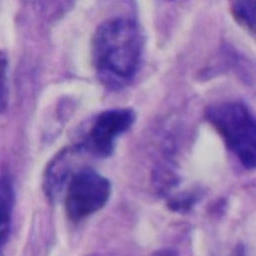

Supplement: Supplementary file 15 [file Data_Sheet_13.zip › SR-02/28_3.tiff]

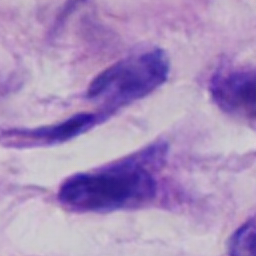

Supplement: Supplementary file 15 [file Data_Sheet_13.zip › SR-02/28_4.tiff]

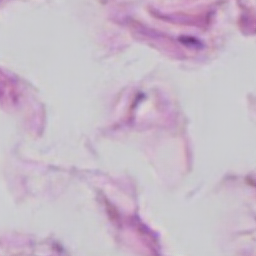

Supplement: Supplementary file 15 [file Data_Sheet_13.zip › SR-02/28_5.tiff]

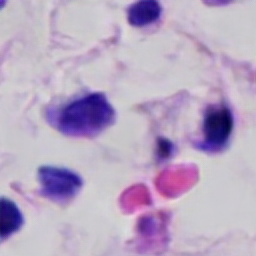

Supplement: Supplementary file 15 [file Data_Sheet_13.zip › SR-02/28_6.tiff]

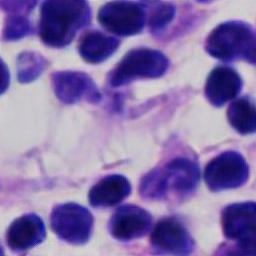

Supplement: Supplementary file 15 [file Data_Sheet_13.zip › SR-02/28_7.tiff]

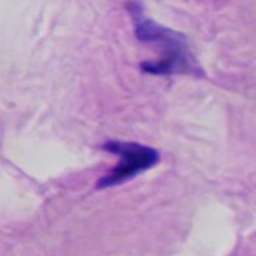

Supplement: Supplementary file 15 [file Data_Sheet_13.zip › SR-02/29_0.tiff]

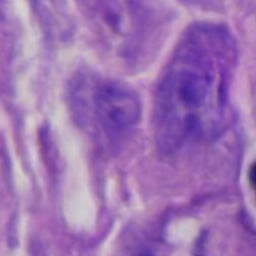

Supplement: Supplementary file 15 [file Data_Sheet_13.zip › SR-02/29_1.tiff]

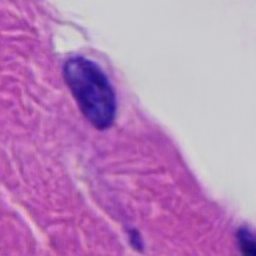

Supplement: Supplementary file 15 [file Data_Sheet_13.zip › SR-02/29_2.tiff]

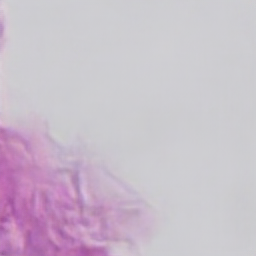

Supplement: Supplementary file 15 [file Data_Sheet_13.zip › SR-02/29_3.tiff]

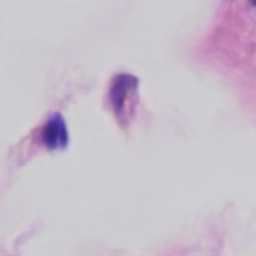

Supplement: Supplementary file 15 [file Data_Sheet_13.zip › SR-02/29_4.tiff]

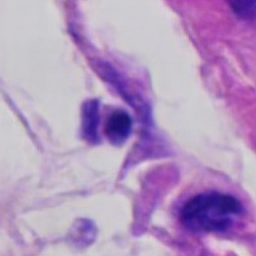

Supplement: Supplementary file 15 [file Data_Sheet_13.zip › SR-02/29_5.tiff]

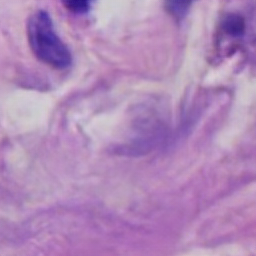

Supplement: Supplementary file 15 [file Data_Sheet_13.zip › SR-02/29_6.tiff]

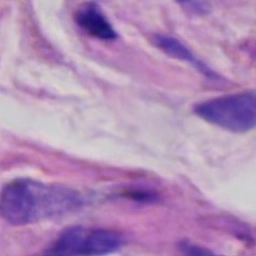

Supplement: Supplementary file 15 [file Data_Sheet_13.zip › SR-02/29_7.tiff]

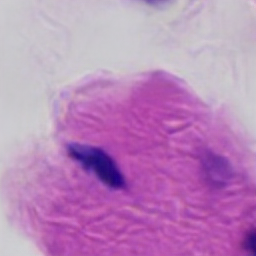

Supplement: Supplementary file 15 [file Data_Sheet_13.zip › SR-02/30_0.tiff]

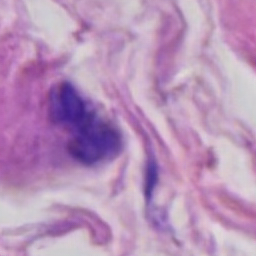

Supplement: Supplementary file 15 [file Data_Sheet_13.zip › SR-02/30_1.tiff]

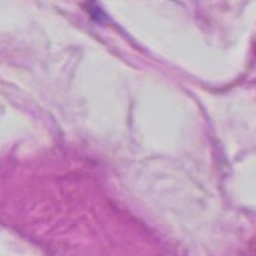

Supplement: Supplementary file 15 [file Data_Sheet_13.zip › SR-02/30_2.tiff]

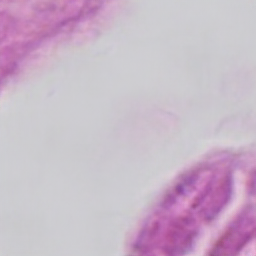

Supplement: Supplementary file 15 [file Data_Sheet_13.zip › SR-02/30_3.tiff]

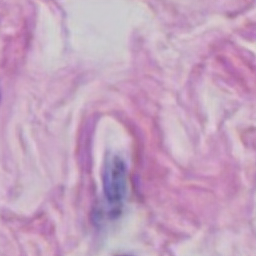

Supplement: Supplementary file 15 [file Data_Sheet_13.zip › SR-02/30_4.tiff]

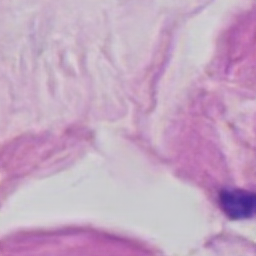

Supplement: Supplementary file 15 [file Data_Sheet_13.zip › SR-02/30_5.tiff]

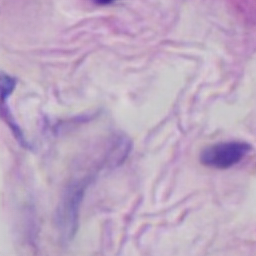

Supplement: Supplementary file 15 [file Data_Sheet_13.zip › SR-02/30_6.tiff]

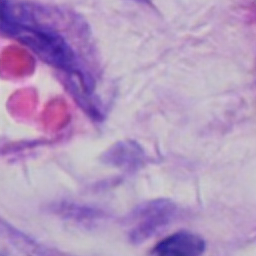

Supplement: Supplementary file 15 [file Data_Sheet_13.zip › SR-02/30_7.tiff]

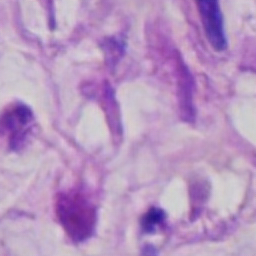

Supplement: Supplementary file 15 [file Data_Sheet_13.zip › SR-02/31_0.tiff]

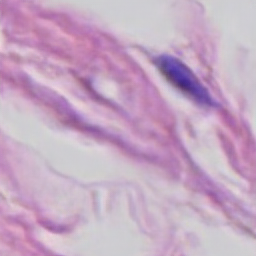

Supplement: Supplementary file 15 [file Data_Sheet_13.zip › SR-02/31_1.tiff]

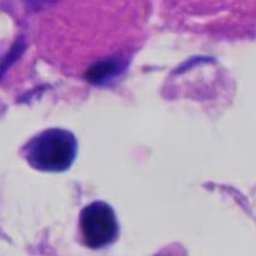

Supplement: Supplementary file 15 [file Data_Sheet_13.zip › SR-02/31_2.tiff]

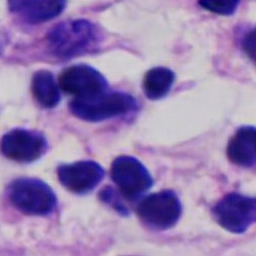

Supplement: Supplementary file 15 [file Data_Sheet_13.zip › SR-02/31_3.tiff]

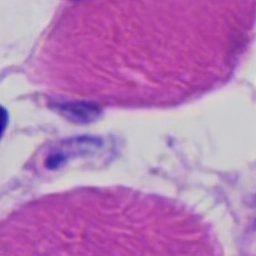

Supplement: Supplementary file 15 [file Data_Sheet_13.zip › SR-02/31_4.tiff]

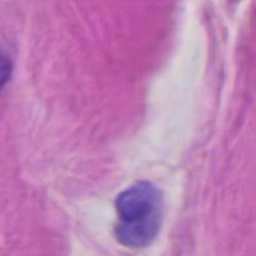

Supplement: Supplementary file 15 [file Data_Sheet_13.zip › SR-02/31_5.tiff]

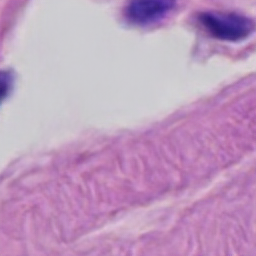

Supplement: Supplementary file 15 [file Data_Sheet_13.zip › SR-02/31_6.tiff]

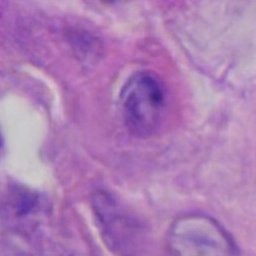

Supplement: Supplementary file 15 [file Data_Sheet_13.zip › SR-02/31_7.tiff]

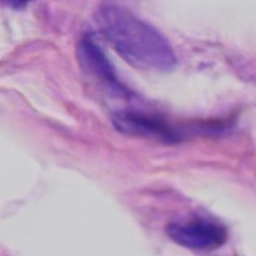

Supplement: Supplementary file 15 [file Data_Sheet_13.zip › SR-02/32_0.tiff]

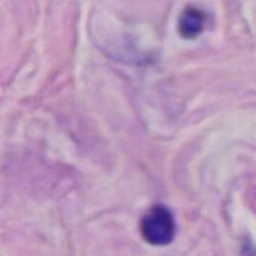

Supplement: Supplementary file 15 [file Data_Sheet_13.zip › SR-02/32_1.tiff]

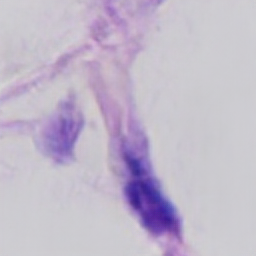

Supplement: Supplementary file 15 [file Data_Sheet_13.zip › SR-02/32_2.tiff]

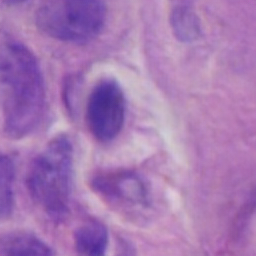

Supplement: Supplementary file 15 [file Data_Sheet_13.zip › SR-02/32_3.tiff]

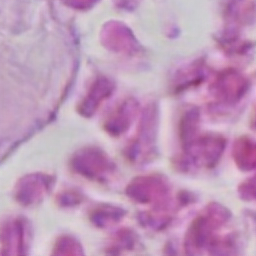

Supplement: Supplementary file 15 [file Data_Sheet_13.zip › SR-02/32_4.tiff]

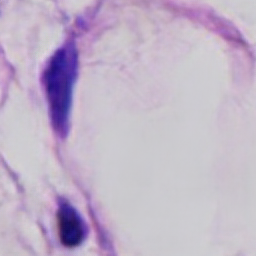

Supplement: Supplementary file 15 [file Data_Sheet_13.zip › SR-02/32_5.tiff]

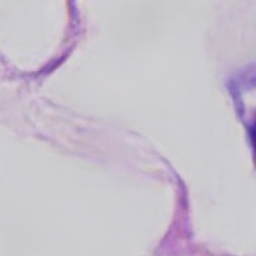

Supplement: Supplementary file 15 [file Data_Sheet_13.zip › SR-02/32_6.tiff]

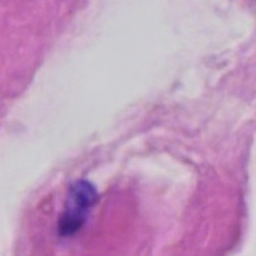

Supplement: Supplementary file 15 [file Data_Sheet_13.zip › SR-02/32_7.tiff]
