# Supplementary figures and images for: Feasibility study of single-image super-resolution scanning system based on deep learning for pathological diagnosis of oral epithelial dysplasia (part 17 of 21)
Source: Front Med (Lausanne). 2025 Mar 12;12:1550512. doi: 10.3389/fmed.2025.1550512 (PMC11936936; doi:10.3389/fmed.2025.1550512)

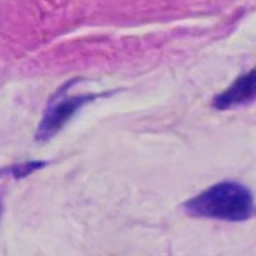

Supplement: Supplementary file 14 [file Data_Sheet_12.zip › SR-01/17_0.tiff]

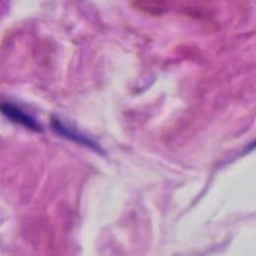

Supplement: Supplementary file 14 [file Data_Sheet_12.zip › SR-01/17_1.tiff]

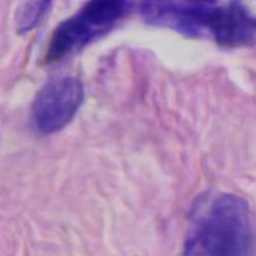

Supplement: Supplementary file 14 [file Data_Sheet_12.zip › SR-01/17_2.tiff]

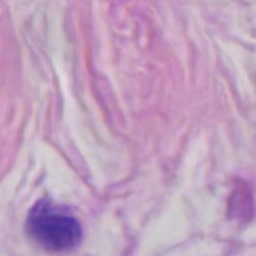

Supplement: Supplementary file 14 [file Data_Sheet_12.zip › SR-01/17_3.tiff]

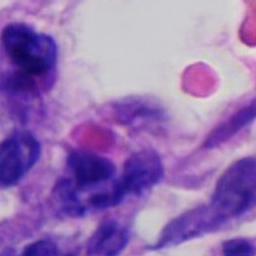

Supplement: Supplementary file 14 [file Data_Sheet_12.zip › SR-01/17_4.tiff]

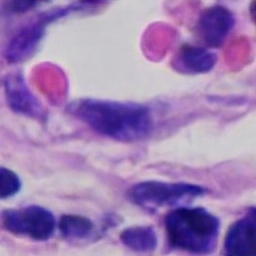

Supplement: Supplementary file 14 [file Data_Sheet_12.zip › SR-01/17_5.tiff]

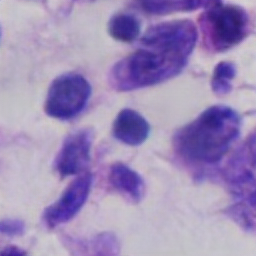

Supplement: Supplementary file 14 [file Data_Sheet_12.zip › SR-01/17_6.tiff]

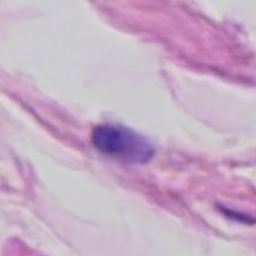

Supplement: Supplementary file 14 [file Data_Sheet_12.zip › SR-01/17_7.tiff]

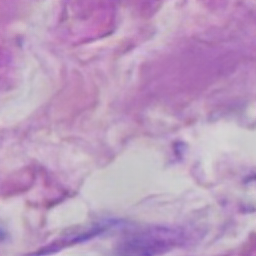

Supplement: Supplementary file 14 [file Data_Sheet_12.zip › SR-01/18_0.tiff]

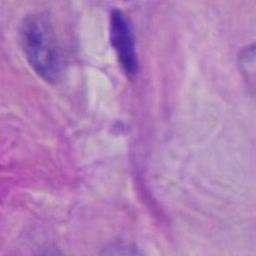

Supplement: Supplementary file 14 [file Data_Sheet_12.zip › SR-01/18_1.tiff]

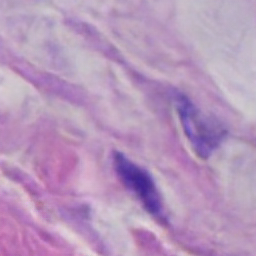

Supplement: Supplementary file 14 [file Data_Sheet_12.zip › SR-01/18_2.tiff]

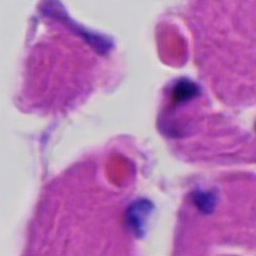

Supplement: Supplementary file 14 [file Data_Sheet_12.zip › SR-01/18_3.tiff]

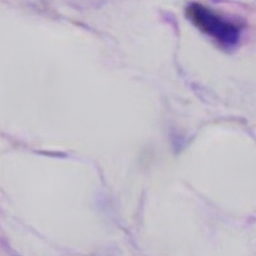

Supplement: Supplementary file 14 [file Data_Sheet_12.zip › SR-01/18_4.tiff]

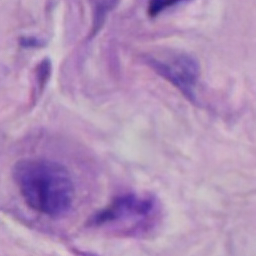

Supplement: Supplementary file 14 [file Data_Sheet_12.zip › SR-01/18_5.tiff]

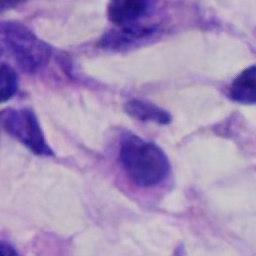

Supplement: Supplementary file 14 [file Data_Sheet_12.zip › SR-01/18_6.tiff]

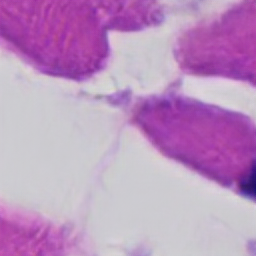

Supplement: Supplementary file 14 [file Data_Sheet_12.zip › SR-01/18_7.tiff]

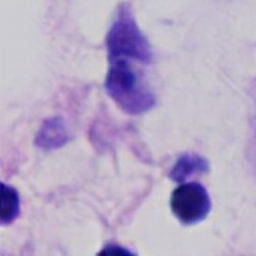

Supplement: Supplementary file 14 [file Data_Sheet_12.zip › SR-01/19_0.tiff]

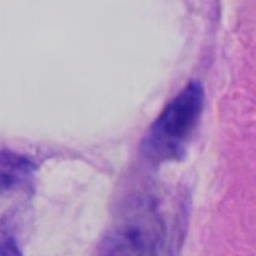

Supplement: Supplementary file 14 [file Data_Sheet_12.zip › SR-01/19_1.tiff]

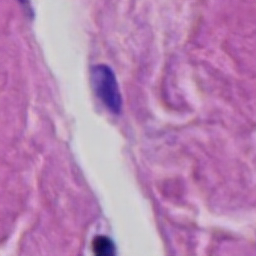

Supplement: Supplementary file 14 [file Data_Sheet_12.zip › SR-01/19_2.tiff]

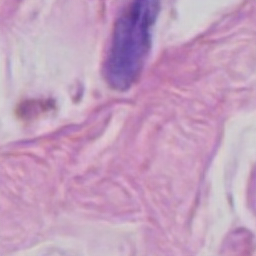

Supplement: Supplementary file 14 [file Data_Sheet_12.zip › SR-01/19_3.tiff]

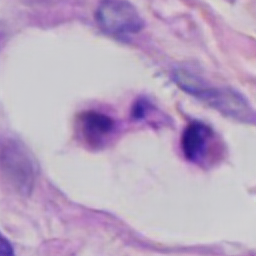

Supplement: Supplementary file 14 [file Data_Sheet_12.zip › SR-01/19_4.tiff]

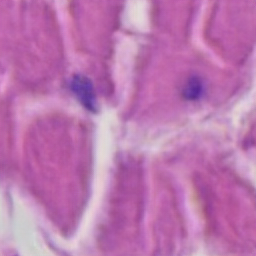

Supplement: Supplementary file 14 [file Data_Sheet_12.zip › SR-01/19_5.tiff]

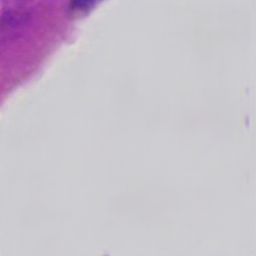

Supplement: Supplementary file 14 [file Data_Sheet_12.zip › SR-01/19_6.tiff]

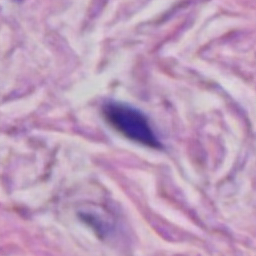

Supplement: Supplementary file 14 [file Data_Sheet_12.zip › SR-01/19_7.tiff]

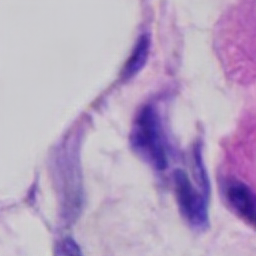

Supplement: Supplementary file 14 [file Data_Sheet_12.zip › SR-01/1_0.tiff]

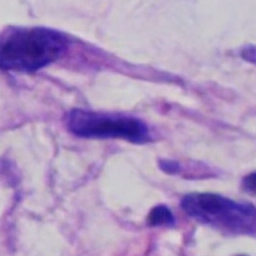

Supplement: Supplementary file 14 [file Data_Sheet_12.zip › SR-01/1_1.tiff]

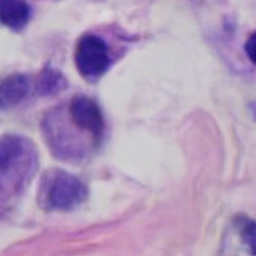

Supplement: Supplementary file 14 [file Data_Sheet_12.zip › SR-01/1_2.tiff]

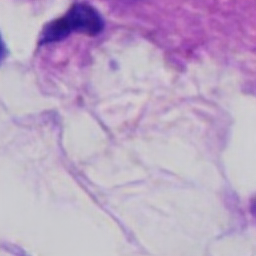

Supplement: Supplementary file 14 [file Data_Sheet_12.zip › SR-01/1_3.tiff]

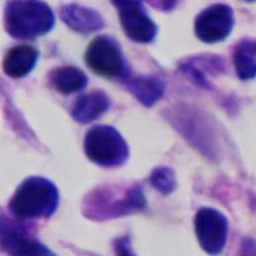

Supplement: Supplementary file 14 [file Data_Sheet_12.zip › SR-01/1_4.tiff]

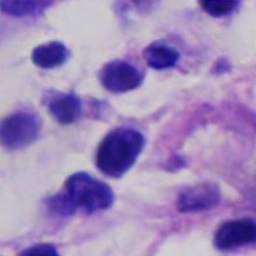

Supplement: Supplementary file 14 [file Data_Sheet_12.zip › SR-01/1_5.tiff]

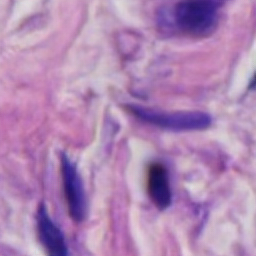

Supplement: Supplementary file 14 [file Data_Sheet_12.zip › SR-01/1_6.tiff]

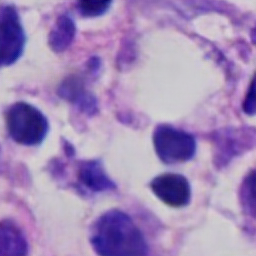

Supplement: Supplementary file 14 [file Data_Sheet_12.zip › SR-01/1_7.tiff]

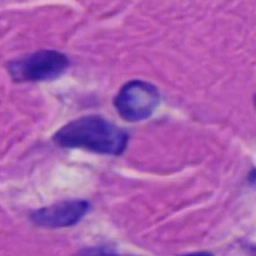

Supplement: Supplementary file 14 [file Data_Sheet_12.zip › SR-01/20_0.tiff]

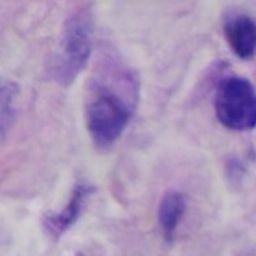

Supplement: Supplementary file 14 [file Data_Sheet_12.zip › SR-01/20_1.tiff]

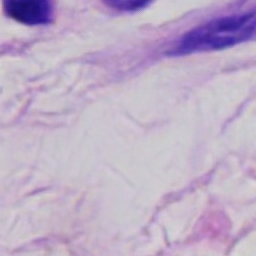

Supplement: Supplementary file 14 [file Data_Sheet_12.zip › SR-01/20_2.tiff]

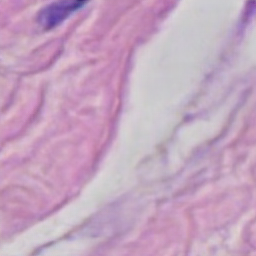

Supplement: Supplementary file 14 [file Data_Sheet_12.zip › SR-01/20_3.tiff]

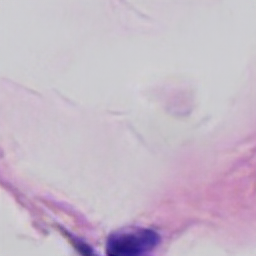

Supplement: Supplementary file 14 [file Data_Sheet_12.zip › SR-01/20_4.tiff]

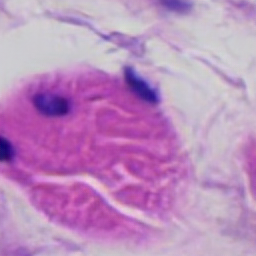

Supplement: Supplementary file 14 [file Data_Sheet_12.zip › SR-01/20_5.tiff]

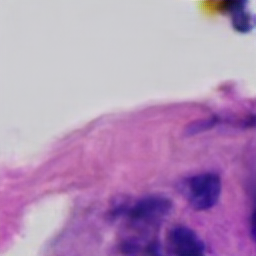

Supplement: Supplementary file 14 [file Data_Sheet_12.zip › SR-01/20_6.tiff]

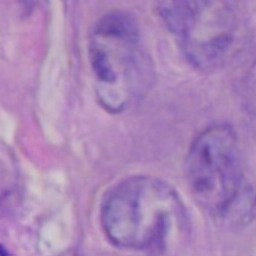

Supplement: Supplementary file 14 [file Data_Sheet_12.zip › SR-01/20_7.tiff]

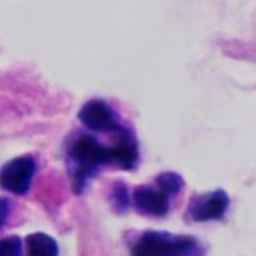

Supplement: Supplementary file 14 [file Data_Sheet_12.zip › SR-01/2_0.tiff]

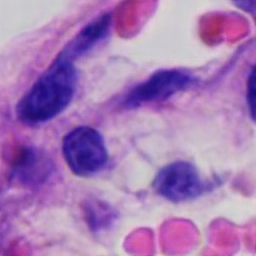

Supplement: Supplementary file 14 [file Data_Sheet_12.zip › SR-01/2_1.tiff]

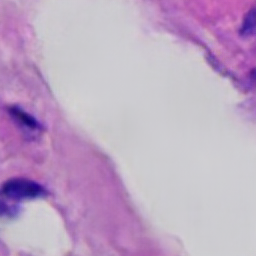

Supplement: Supplementary file 14 [file Data_Sheet_12.zip › SR-01/2_2.tiff]

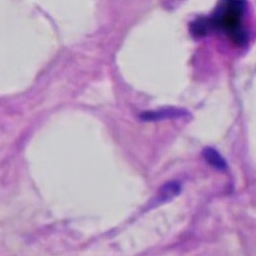

Supplement: Supplementary file 14 [file Data_Sheet_12.zip › SR-01/2_3.tiff]

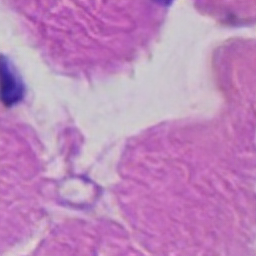

Supplement: Supplementary file 14 [file Data_Sheet_12.zip › SR-01/2_4.tiff]

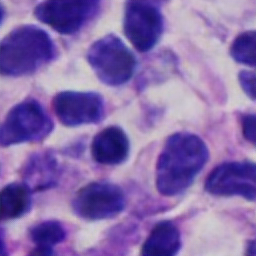

Supplement: Supplementary file 14 [file Data_Sheet_12.zip › SR-01/2_5.tiff]

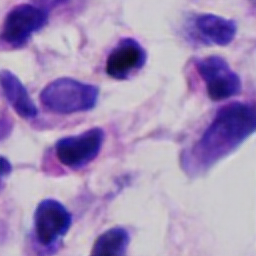

Supplement: Supplementary file 14 [file Data_Sheet_12.zip › SR-01/2_6.tiff]

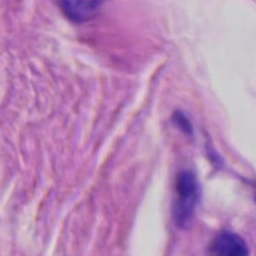

Supplement: Supplementary file 14 [file Data_Sheet_12.zip › SR-01/2_7.tiff]

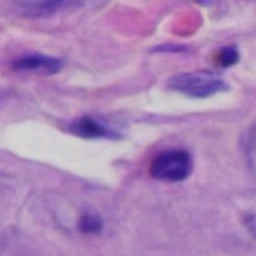

Supplement: Supplementary file 14 [file Data_Sheet_12.zip › SR-01/3_0.tiff]

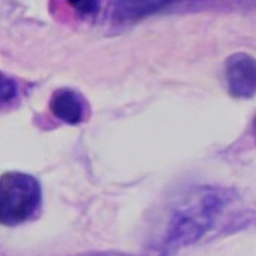

Supplement: Supplementary file 14 [file Data_Sheet_12.zip › SR-01/3_1.tiff]

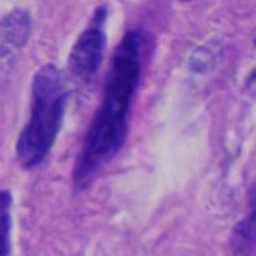

Supplement: Supplementary file 14 [file Data_Sheet_12.zip › SR-01/3_2.tiff]

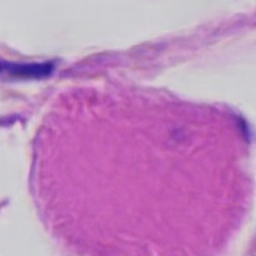

Supplement: Supplementary file 14 [file Data_Sheet_12.zip › SR-01/3_3.tiff]

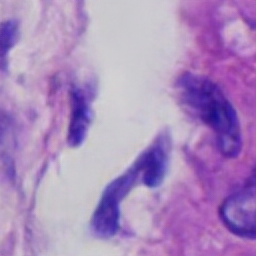

Supplement: Supplementary file 14 [file Data_Sheet_12.zip › SR-01/3_4.tiff]

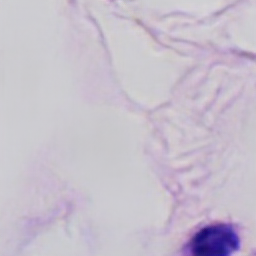

Supplement: Supplementary file 14 [file Data_Sheet_12.zip › SR-01/3_5.tiff]

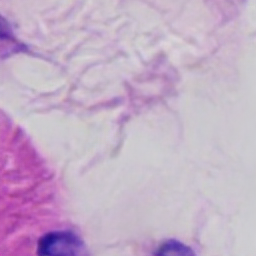

Supplement: Supplementary file 14 [file Data_Sheet_12.zip › SR-01/3_6.tiff]

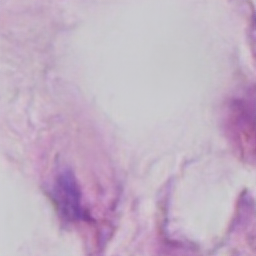

Supplement: Supplementary file 14 [file Data_Sheet_12.zip › SR-01/3_7.tiff]

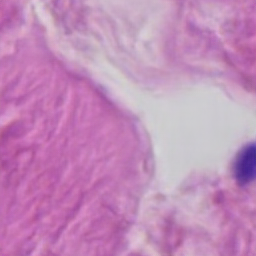

Supplement: Supplementary file 14 [file Data_Sheet_12.zip › SR-01/4_0.tiff]

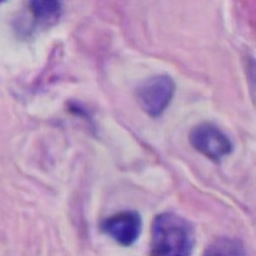

Supplement: Supplementary file 14 [file Data_Sheet_12.zip › SR-01/4_1.tiff]

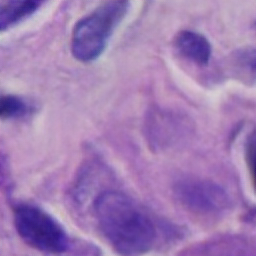

Supplement: Supplementary file 14 [file Data_Sheet_12.zip › SR-01/4_2.tiff]

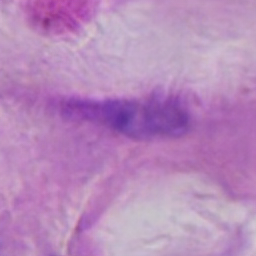

Supplement: Supplementary file 14 [file Data_Sheet_12.zip › SR-01/4_3.tiff]

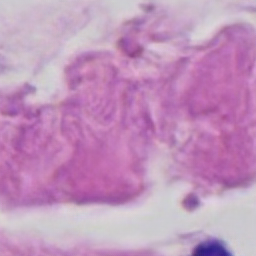

Supplement: Supplementary file 14 [file Data_Sheet_12.zip › SR-01/4_4.tiff]

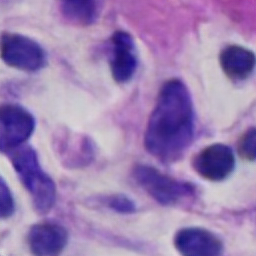

Supplement: Supplementary file 14 [file Data_Sheet_12.zip › SR-01/4_5.tiff]

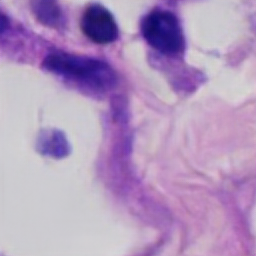

Supplement: Supplementary file 14 [file Data_Sheet_12.zip › SR-01/4_6.tiff]

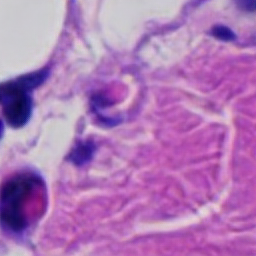

Supplement: Supplementary file 14 [file Data_Sheet_12.zip › SR-01/4_7.tiff]

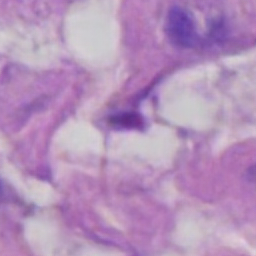

Supplement: Supplementary file 14 [file Data_Sheet_12.zip › SR-01/5_0.tiff]

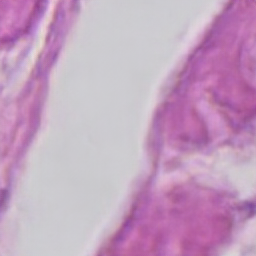

Supplement: Supplementary file 14 [file Data_Sheet_12.zip › SR-01/5_1.tiff]

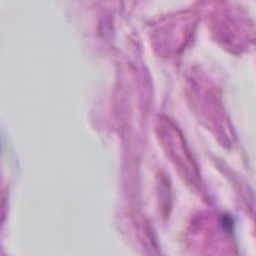

Supplement: Supplementary file 14 [file Data_Sheet_12.zip › SR-01/5_2.tiff]

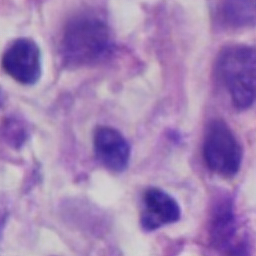

Supplement: Supplementary file 14 [file Data_Sheet_12.zip › SR-01/5_3.tiff]

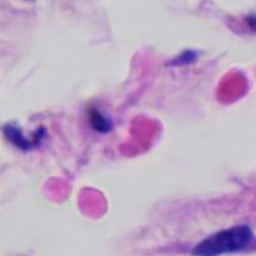

Supplement: Supplementary file 14 [file Data_Sheet_12.zip › SR-01/5_4.tiff]

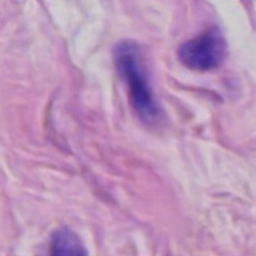

Supplement: Supplementary file 14 [file Data_Sheet_12.zip › SR-01/5_5.tiff]

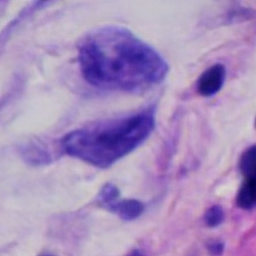

Supplement: Supplementary file 14 [file Data_Sheet_12.zip › SR-01/5_6.tiff]

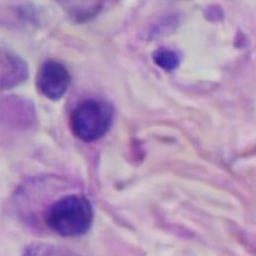

Supplement: Supplementary file 14 [file Data_Sheet_12.zip › SR-01/5_7.tiff]

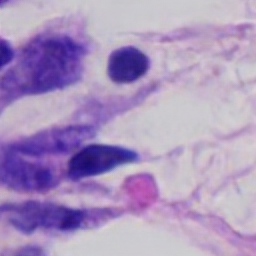

Supplement: Supplementary file 14 [file Data_Sheet_12.zip › SR-01/6_0.tiff]

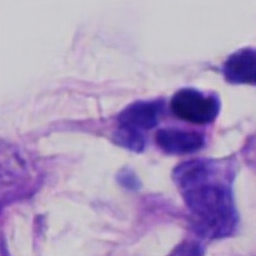

Supplement: Supplementary file 14 [file Data_Sheet_12.zip › SR-01/6_1.tiff]

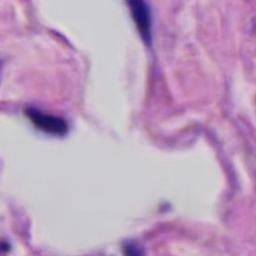

Supplement: Supplementary file 14 [file Data_Sheet_12.zip › SR-01/6_2.tiff]

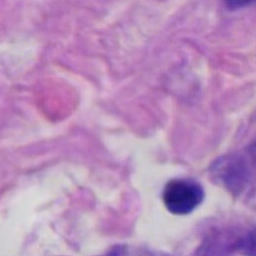

Supplement: Supplementary file 14 [file Data_Sheet_12.zip › SR-01/6_3.tiff]

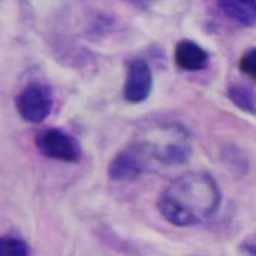

Supplement: Supplementary file 14 [file Data_Sheet_12.zip › SR-01/6_4.tiff]

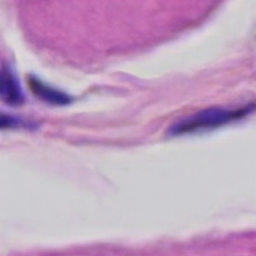

Supplement: Supplementary file 14 [file Data_Sheet_12.zip › SR-01/6_5.tiff]

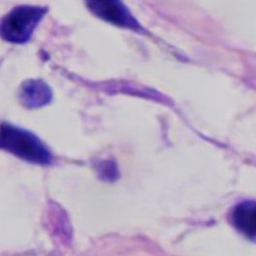

Supplement: Supplementary file 14 [file Data_Sheet_12.zip › SR-01/6_6.tiff]

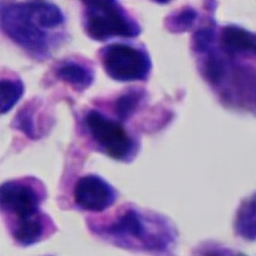

Supplement: Supplementary file 14 [file Data_Sheet_12.zip › SR-01/6_7.tiff]

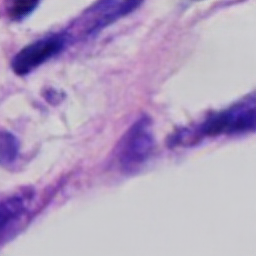

Supplement: Supplementary file 14 [file Data_Sheet_12.zip › SR-01/7_0.tiff]

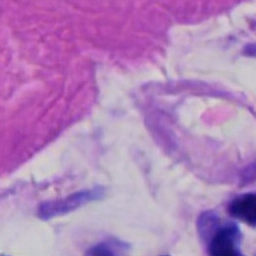

Supplement: Supplementary file 14 [file Data_Sheet_12.zip › SR-01/7_1.tiff]

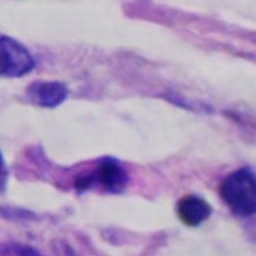

Supplement: Supplementary file 14 [file Data_Sheet_12.zip › SR-01/7_2.tiff]

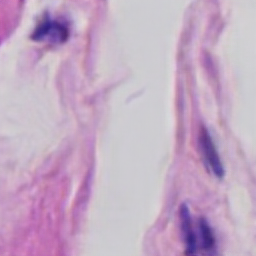

Supplement: Supplementary file 14 [file Data_Sheet_12.zip › SR-01/7_3.tiff]

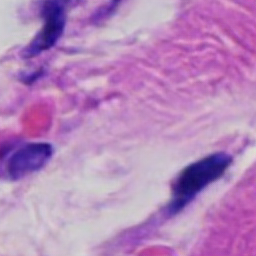

Supplement: Supplementary file 14 [file Data_Sheet_12.zip › SR-01/7_4.tiff]

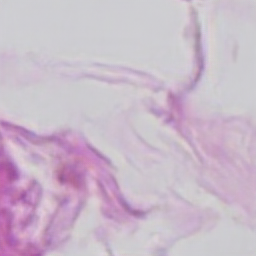

Supplement: Supplementary file 14 [file Data_Sheet_12.zip › SR-01/7_5.tiff]

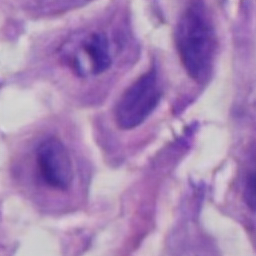

Supplement: Supplementary file 14 [file Data_Sheet_12.zip › SR-01/7_6.tiff]

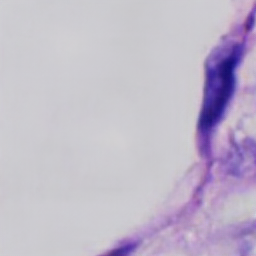

Supplement: Supplementary file 14 [file Data_Sheet_12.zip › SR-01/7_7.tiff]

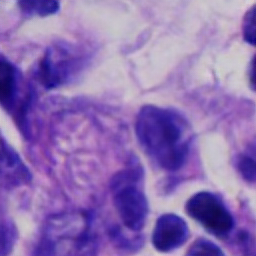

Supplement: Supplementary file 14 [file Data_Sheet_12.zip › SR-01/8_0.tiff]

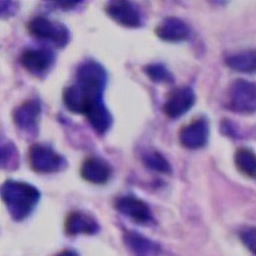

Supplement: Supplementary file 14 [file Data_Sheet_12.zip › SR-01/8_1.tiff]

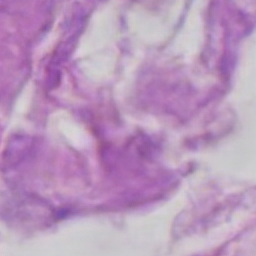

Supplement: Supplementary file 14 [file Data_Sheet_12.zip › SR-01/8_2.tiff]

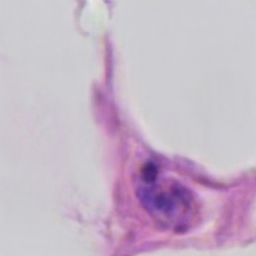

Supplement: Supplementary file 14 [file Data_Sheet_12.zip › SR-01/8_3.tiff]

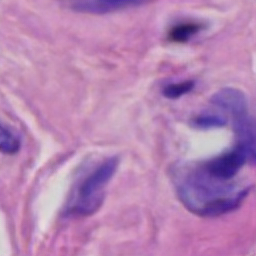

Supplement: Supplementary file 14 [file Data_Sheet_12.zip › SR-01/8_4.tiff]

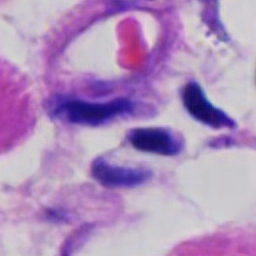

Supplement: Supplementary file 14 [file Data_Sheet_12.zip › SR-01/8_5.tiff]

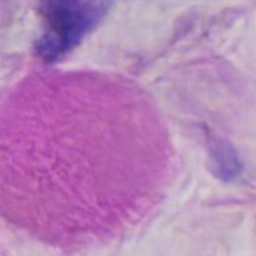

Supplement: Supplementary file 14 [file Data_Sheet_12.zip › SR-01/8_6.tiff]

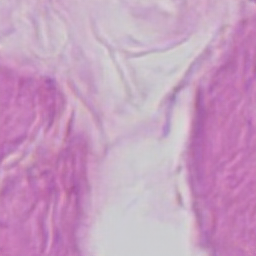

Supplement: Supplementary file 14 [file Data_Sheet_12.zip › SR-01/8_7.tiff]

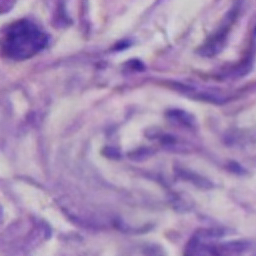

Supplement: Supplementary file 14 [file Data_Sheet_12.zip › SR-01/9_0.tiff]

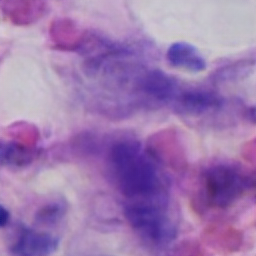

Supplement: Supplementary file 14 [file Data_Sheet_12.zip › SR-01/9_1.tiff]

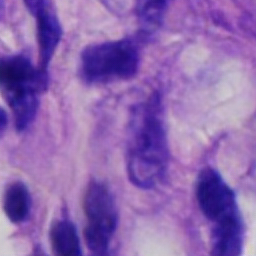

Supplement: Supplementary file 14 [file Data_Sheet_12.zip › SR-01/9_2.tiff]

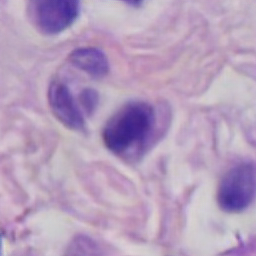

Supplement: Supplementary file 14 [file Data_Sheet_12.zip › SR-01/9_3.tiff]
