# Supplementary figures and images for: Feasibility study of single-image super-resolution scanning system based on deep learning for pathological diagnosis of oral epithelial dysplasia (part 20 of 21)
Source: Front Med (Lausanne). 2025 Mar 12;12:1550512. doi: 10.3389/fmed.2025.1550512 (PMC11936936; doi:10.3389/fmed.2025.1550512)

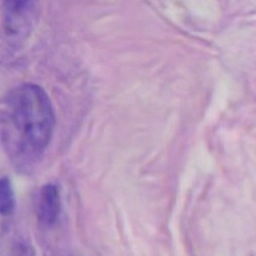

Supplement: Supplementary file 15 [file Data_Sheet_13.zip › SR-02/45_4.tiff]

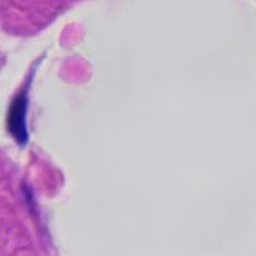

Supplement: Supplementary file 15 [file Data_Sheet_13.zip › SR-02/45_5.tiff]

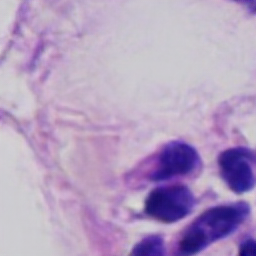

Supplement: Supplementary file 15 [file Data_Sheet_13.zip › SR-02/45_6.tiff]

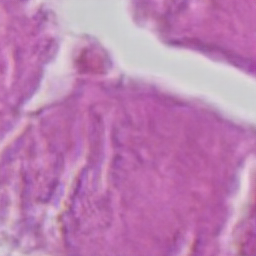

Supplement: Supplementary file 15 [file Data_Sheet_13.zip › SR-02/45_7.tiff]

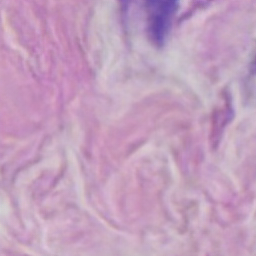

Supplement: Supplementary file 16 [file Data_Sheet_14.zip › SR-03/56_0.tiff]

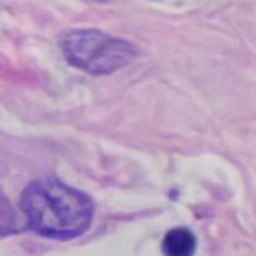

Supplement: Supplementary file 16 [file Data_Sheet_14.zip › SR-03/56_1.tiff]

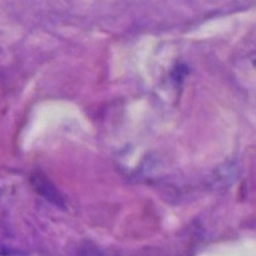

Supplement: Supplementary file 16 [file Data_Sheet_14.zip › SR-03/56_2.tiff]

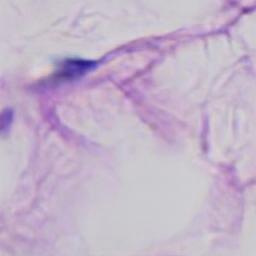

Supplement: Supplementary file 16 [file Data_Sheet_14.zip › SR-03/56_3.tiff]

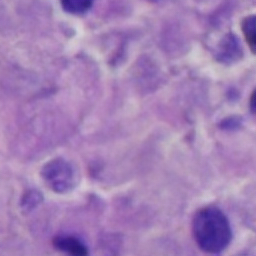

Supplement: Supplementary file 16 [file Data_Sheet_14.zip › SR-03/56_4.tiff]

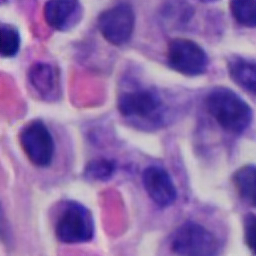

Supplement: Supplementary file 16 [file Data_Sheet_14.zip › SR-03/56_5.tiff]

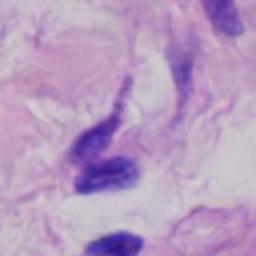

Supplement: Supplementary file 16 [file Data_Sheet_14.zip › SR-03/56_6.tiff]

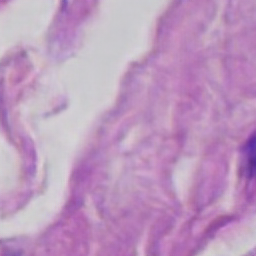

Supplement: Supplementary file 16 [file Data_Sheet_14.zip › SR-03/56_7.tiff]

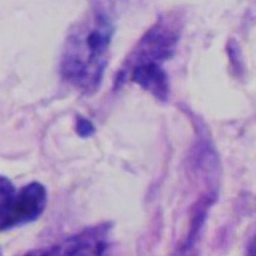

Supplement: Supplementary file 16 [file Data_Sheet_14.zip › SR-03/57_0.tiff]

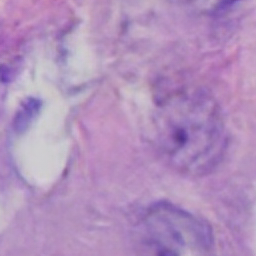

Supplement: Supplementary file 16 [file Data_Sheet_14.zip › SR-03/57_1.tiff]

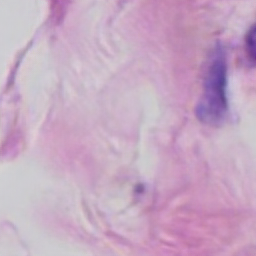

Supplement: Supplementary file 16 [file Data_Sheet_14.zip › SR-03/57_2.tiff]

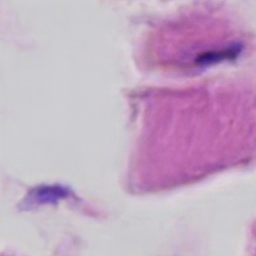

Supplement: Supplementary file 16 [file Data_Sheet_14.zip › SR-03/57_3.tiff]

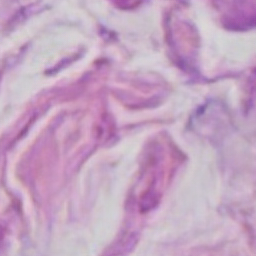

Supplement: Supplementary file 16 [file Data_Sheet_14.zip › SR-03/57_4.tiff]

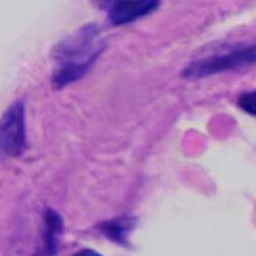

Supplement: Supplementary file 16 [file Data_Sheet_14.zip › SR-03/57_5.tiff]

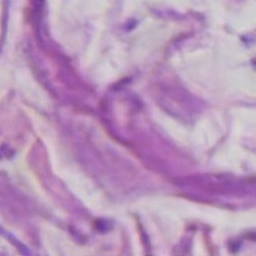

Supplement: Supplementary file 16 [file Data_Sheet_14.zip › SR-03/57_6.tiff]

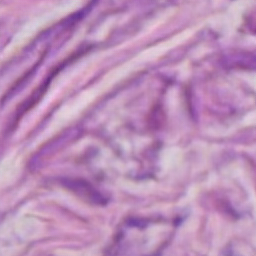

Supplement: Supplementary file 16 [file Data_Sheet_14.zip › SR-03/57_7.tiff]

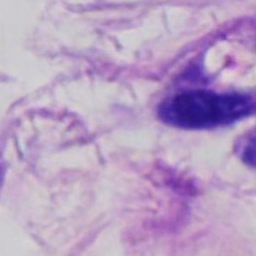

Supplement: Supplementary file 16 [file Data_Sheet_14.zip › SR-03/58_0.tiff]

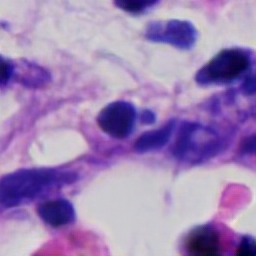

Supplement: Supplementary file 16 [file Data_Sheet_14.zip › SR-03/58_1.tiff]

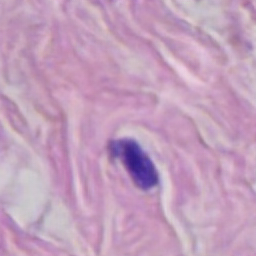

Supplement: Supplementary file 16 [file Data_Sheet_14.zip › SR-03/58_2.tiff]

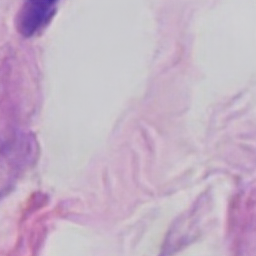

Supplement: Supplementary file 16 [file Data_Sheet_14.zip › SR-03/58_3.tiff]

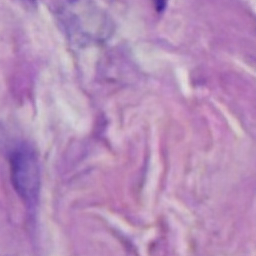

Supplement: Supplementary file 16 [file Data_Sheet_14.zip › SR-03/58_4.tiff]

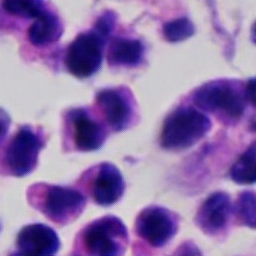

Supplement: Supplementary file 16 [file Data_Sheet_14.zip › SR-03/58_5.tiff]

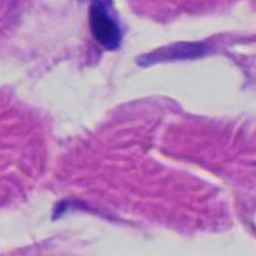

Supplement: Supplementary file 16 [file Data_Sheet_14.zip › SR-03/58_6.tiff]

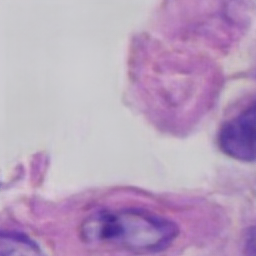

Supplement: Supplementary file 16 [file Data_Sheet_14.zip › SR-03/58_7.tiff]

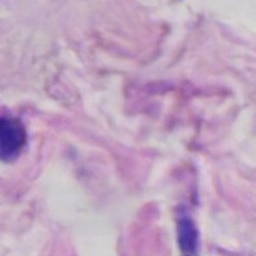

Supplement: Supplementary file 16 [file Data_Sheet_14.zip › SR-03/59_0.tiff]

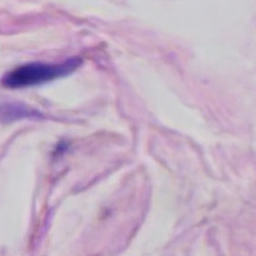

Supplement: Supplementary file 16 [file Data_Sheet_14.zip › SR-03/59_1.tiff]

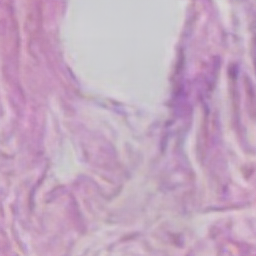

Supplement: Supplementary file 16 [file Data_Sheet_14.zip › SR-03/59_2.tiff]

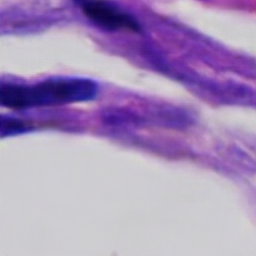

Supplement: Supplementary file 16 [file Data_Sheet_14.zip › SR-03/59_3.tiff]

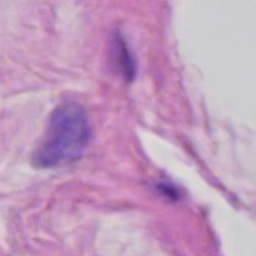

Supplement: Supplementary file 16 [file Data_Sheet_14.zip › SR-03/59_4.tiff]

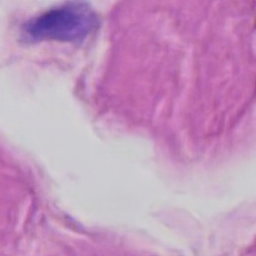

Supplement: Supplementary file 16 [file Data_Sheet_14.zip › SR-03/59_5.tiff]

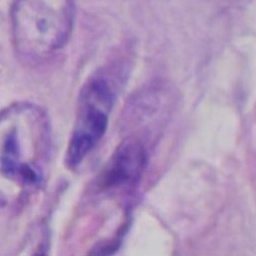

Supplement: Supplementary file 16 [file Data_Sheet_14.zip › SR-03/59_6.tiff]

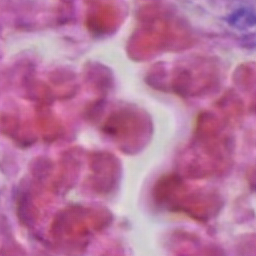

Supplement: Supplementary file 16 [file Data_Sheet_14.zip › SR-03/59_7.tiff]

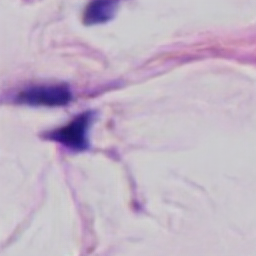

Supplement: Supplementary file 16 [file Data_Sheet_14.zip › SR-03/60_0.tiff]

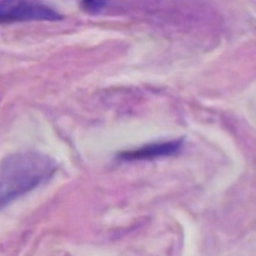

Supplement: Supplementary file 16 [file Data_Sheet_14.zip › SR-03/60_1.tiff]

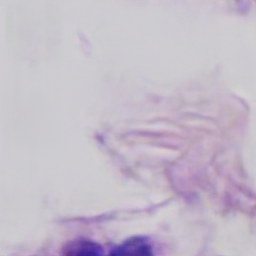

Supplement: Supplementary file 16 [file Data_Sheet_14.zip › SR-03/60_2.tiff]

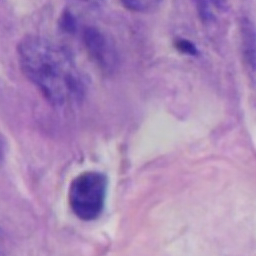

Supplement: Supplementary file 16 [file Data_Sheet_14.zip › SR-03/60_3.tiff]

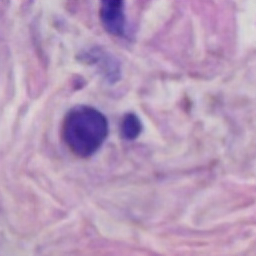

Supplement: Supplementary file 16 [file Data_Sheet_14.zip › SR-03/60_4.tiff]

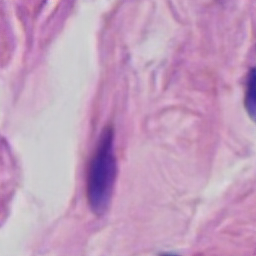

Supplement: Supplementary file 16 [file Data_Sheet_14.zip › SR-03/60_5.tiff]

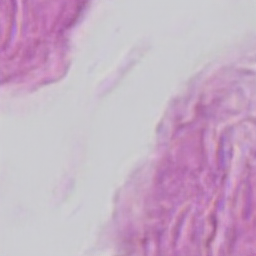

Supplement: Supplementary file 16 [file Data_Sheet_14.zip › SR-03/60_6.tiff]

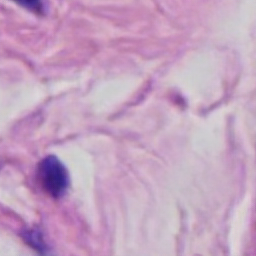

Supplement: Supplementary file 16 [file Data_Sheet_14.zip › SR-03/60_7.tiff]

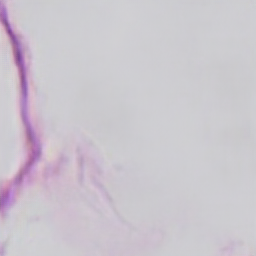

Supplement: Supplementary file 16 [file Data_Sheet_14.zip › SR-03/61_0.tiff]

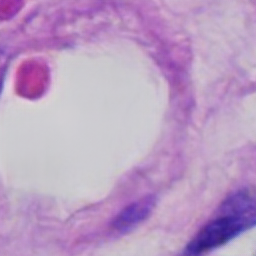

Supplement: Supplementary file 16 [file Data_Sheet_14.zip › SR-03/61_1.tiff]

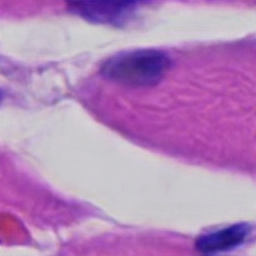

Supplement: Supplementary file 16 [file Data_Sheet_14.zip › SR-03/61_2.tiff]

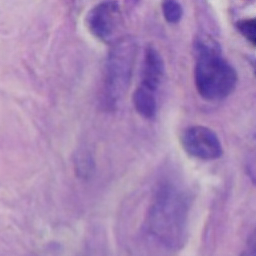

Supplement: Supplementary file 16 [file Data_Sheet_14.zip › SR-03/61_3.tiff]

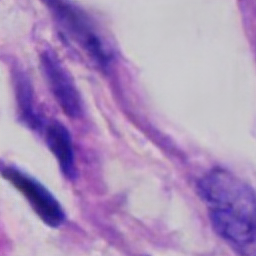

Supplement: Supplementary file 16 [file Data_Sheet_14.zip › SR-03/61_4.tiff]

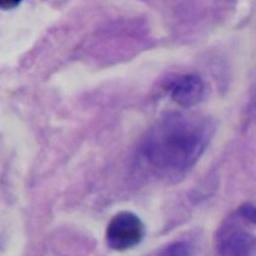

Supplement: Supplementary file 16 [file Data_Sheet_14.zip › SR-03/61_5.tiff]

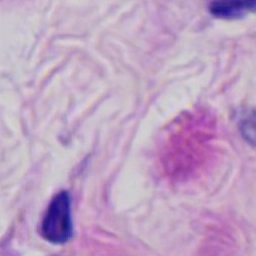

Supplement: Supplementary file 16 [file Data_Sheet_14.zip › SR-03/61_6.tiff]

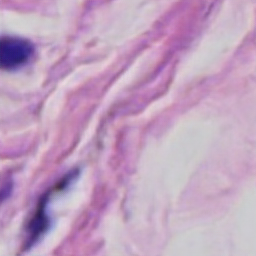

Supplement: Supplementary file 16 [file Data_Sheet_14.zip › SR-03/61_7.tiff]

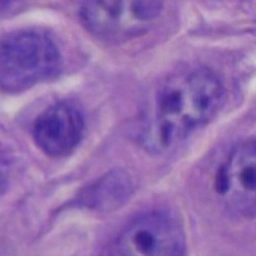

Supplement: Supplementary file 16 [file Data_Sheet_14.zip › SR-03/62_0.tiff]

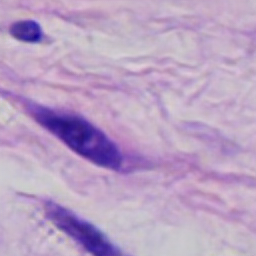

Supplement: Supplementary file 16 [file Data_Sheet_14.zip › SR-03/62_1.tiff]

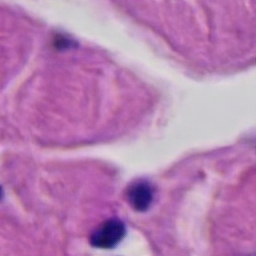

Supplement: Supplementary file 16 [file Data_Sheet_14.zip › SR-03/62_2.tiff]

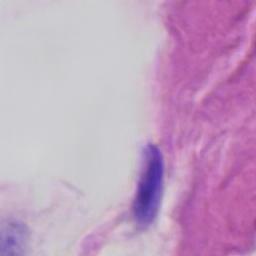

Supplement: Supplementary file 16 [file Data_Sheet_14.zip › SR-03/62_3.tiff]

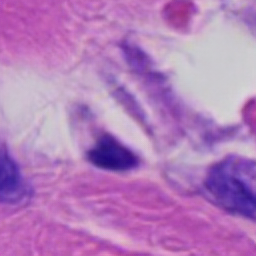

Supplement: Supplementary file 16 [file Data_Sheet_14.zip › SR-03/62_4.tiff]

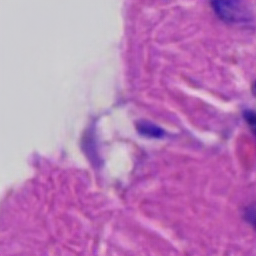

Supplement: Supplementary file 16 [file Data_Sheet_14.zip › SR-03/62_5.tiff]

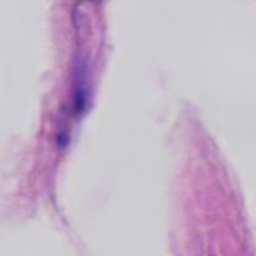

Supplement: Supplementary file 16 [file Data_Sheet_14.zip › SR-03/62_6.tiff]

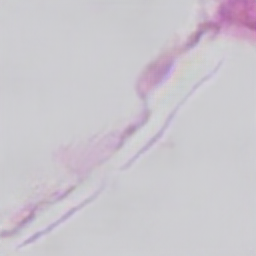

Supplement: Supplementary file 16 [file Data_Sheet_14.zip › SR-03/62_7.tiff]

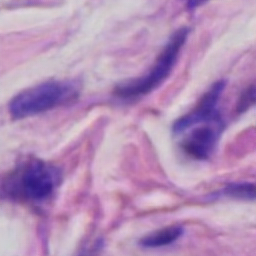

Supplement: Supplementary file 16 [file Data_Sheet_14.zip › SR-03/63_0.tiff]

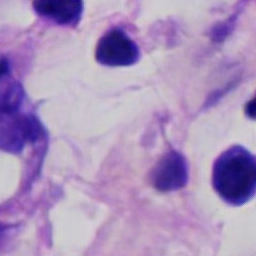

Supplement: Supplementary file 16 [file Data_Sheet_14.zip › SR-03/63_1.tiff]

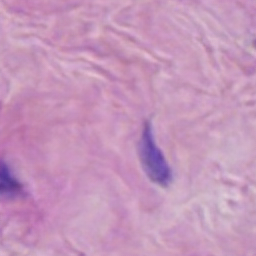

Supplement: Supplementary file 16 [file Data_Sheet_14.zip › SR-03/63_2.tiff]

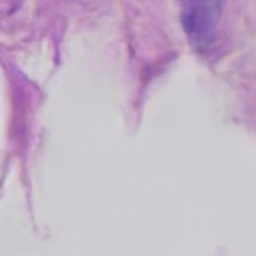

Supplement: Supplementary file 16 [file Data_Sheet_14.zip › SR-03/63_3.tiff]

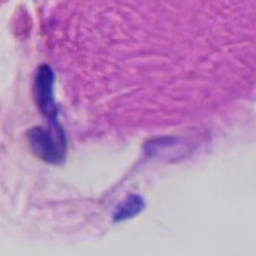

Supplement: Supplementary file 16 [file Data_Sheet_14.zip › SR-03/63_4.tiff]

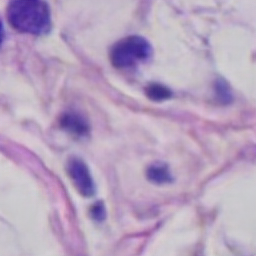

Supplement: Supplementary file 16 [file Data_Sheet_14.zip › SR-03/63_5.tiff]

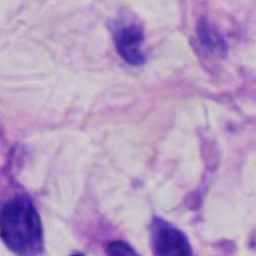

Supplement: Supplementary file 16 [file Data_Sheet_14.zip › SR-03/63_6.tiff]

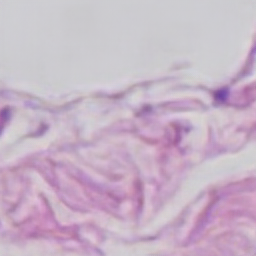

Supplement: Supplementary file 16 [file Data_Sheet_14.zip › SR-03/63_7.tiff]

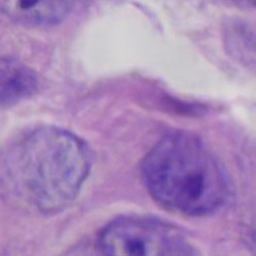

Supplement: Supplementary file 16 [file Data_Sheet_14.zip › SR-03/64_0.tiff]

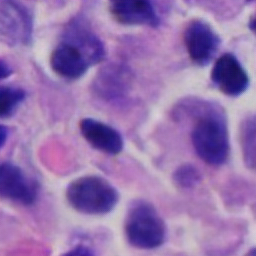

Supplement: Supplementary file 16 [file Data_Sheet_14.zip › SR-03/64_1.tiff]

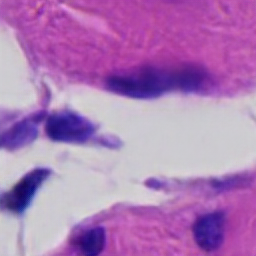

Supplement: Supplementary file 16 [file Data_Sheet_14.zip › SR-03/64_2.tiff]

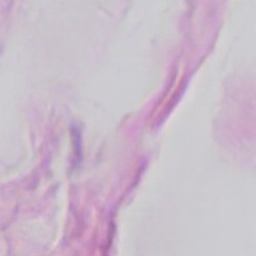

Supplement: Supplementary file 16 [file Data_Sheet_14.zip › SR-03/64_3.tiff]

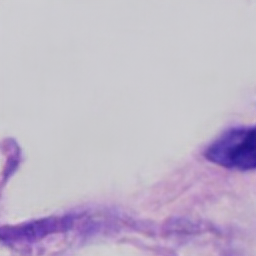

Supplement: Supplementary file 16 [file Data_Sheet_14.zip › SR-03/64_4.tiff]

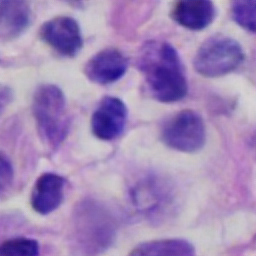

Supplement: Supplementary file 16 [file Data_Sheet_14.zip › SR-03/64_5.tiff]

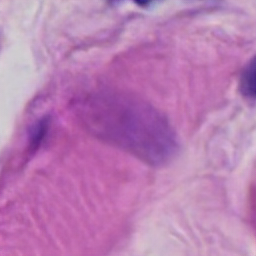

Supplement: Supplementary file 16 [file Data_Sheet_14.zip › SR-03/64_6.tiff]

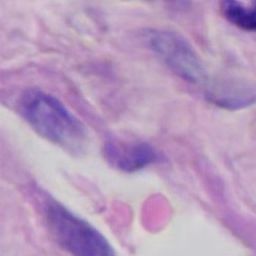

Supplement: Supplementary file 16 [file Data_Sheet_14.zip › SR-03/64_7.tiff]

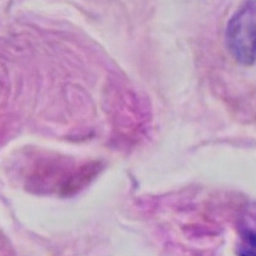

Supplement: Supplementary file 16 [file Data_Sheet_14.zip › SR-03/65_0.tiff]

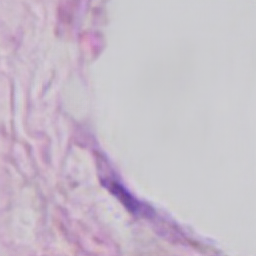

Supplement: Supplementary file 16 [file Data_Sheet_14.zip › SR-03/65_1.tiff]

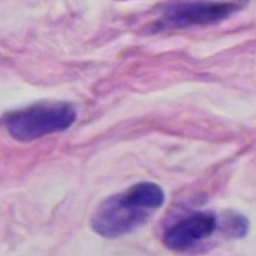

Supplement: Supplementary file 16 [file Data_Sheet_14.zip › SR-03/65_2.tiff]

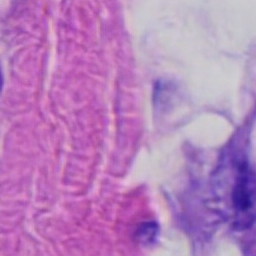

Supplement: Supplementary file 16 [file Data_Sheet_14.zip › SR-03/65_3.tiff]

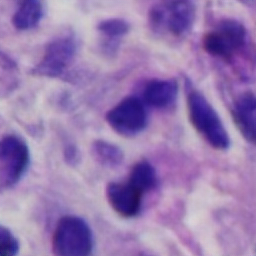

Supplement: Supplementary file 16 [file Data_Sheet_14.zip › SR-03/65_4.tiff]

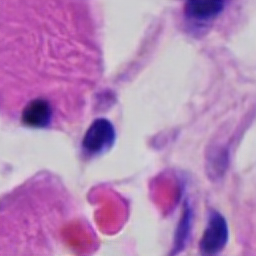

Supplement: Supplementary file 16 [file Data_Sheet_14.zip › SR-03/65_5.tiff]

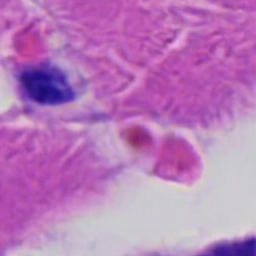

Supplement: Supplementary file 16 [file Data_Sheet_14.zip › SR-03/65_6.tiff]

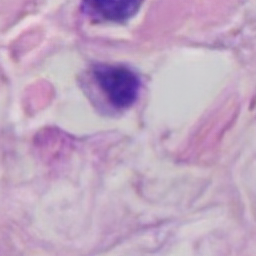

Supplement: Supplementary file 16 [file Data_Sheet_14.zip › SR-03/65_7.tiff]

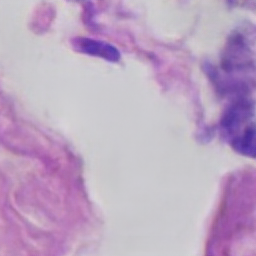

Supplement: Supplementary file 16 [file Data_Sheet_14.zip › SR-03/66_0.tiff]

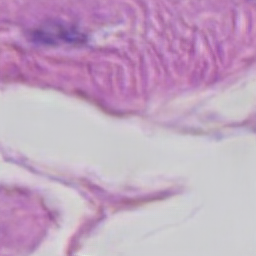

Supplement: Supplementary file 16 [file Data_Sheet_14.zip › SR-03/66_1.tiff]

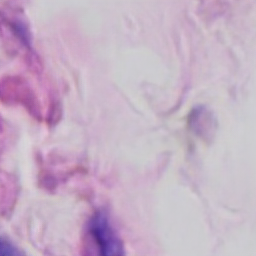

Supplement: Supplementary file 16 [file Data_Sheet_14.zip › SR-03/66_2.tiff]

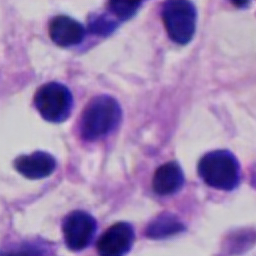

Supplement: Supplementary file 16 [file Data_Sheet_14.zip › SR-03/66_3.tiff]

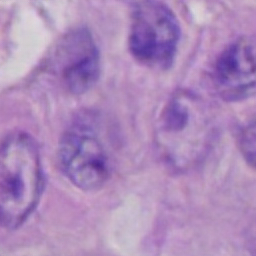

Supplement: Supplementary file 16 [file Data_Sheet_14.zip › SR-03/66_4.tiff]

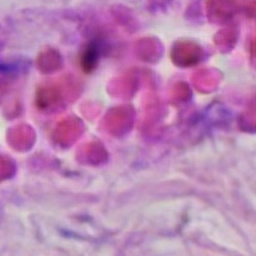

Supplement: Supplementary file 16 [file Data_Sheet_14.zip › SR-03/66_5.tiff]

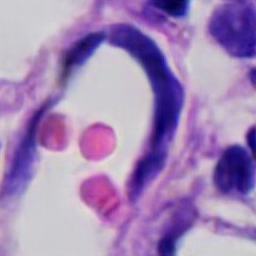

Supplement: Supplementary file 16 [file Data_Sheet_14.zip › SR-03/66_6.tiff]

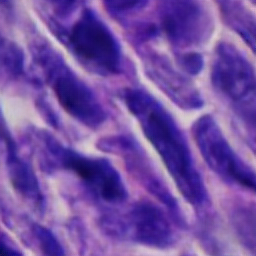

Supplement: Supplementary file 16 [file Data_Sheet_14.zip › SR-03/66_7.tiff]

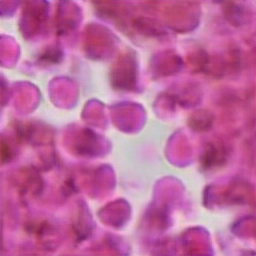

Supplement: Supplementary file 16 [file Data_Sheet_14.zip › SR-03/67_0.tiff]

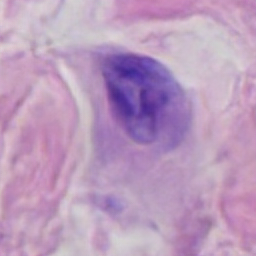

Supplement: Supplementary file 16 [file Data_Sheet_14.zip › SR-03/67_1.tiff]

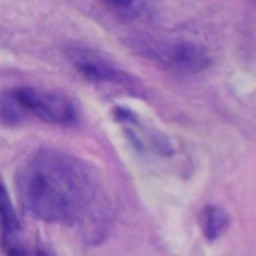

Supplement: Supplementary file 16 [file Data_Sheet_14.zip › SR-03/67_2.tiff]

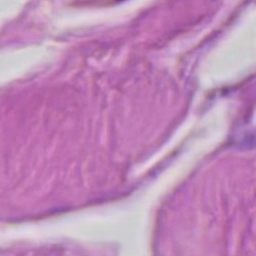

Supplement: Supplementary file 16 [file Data_Sheet_14.zip › SR-03/67_3.tiff]

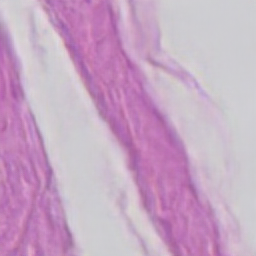

Supplement: Supplementary file 16 [file Data_Sheet_14.zip › SR-03/67_4.tiff]

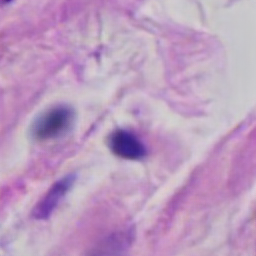

Supplement: Supplementary file 16 [file Data_Sheet_14.zip › SR-03/67_5.tiff]

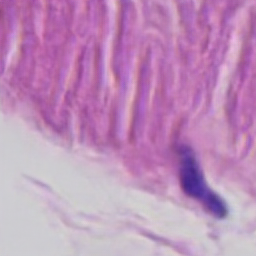

Supplement: Supplementary file 16 [file Data_Sheet_14.zip › SR-03/67_6.tiff]

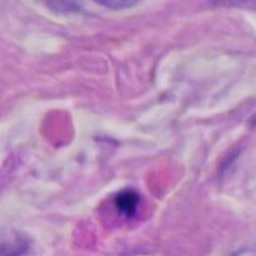

Supplement: Supplementary file 16 [file Data_Sheet_14.zip › SR-03/67_7.tiff]
