# Supplementary figures and images for: Feasibility study of single-image super-resolution scanning system based on deep learning for pathological diagnosis of oral epithelial dysplasia (part 19 of 21)
Source: Front Med (Lausanne). 2025 Mar 12;12:1550512. doi: 10.3389/fmed.2025.1550512 (PMC11936936; doi:10.3389/fmed.2025.1550512)

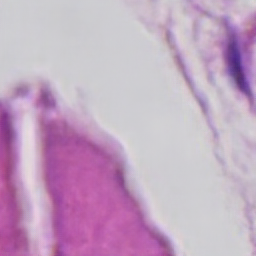

Supplement: Supplementary file 15 [file Data_Sheet_13.zip › SR-02/33_0.tiff]

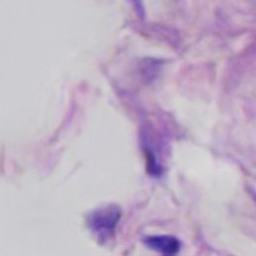

Supplement: Supplementary file 15 [file Data_Sheet_13.zip › SR-02/33_1.tiff]

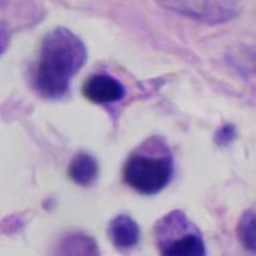

Supplement: Supplementary file 15 [file Data_Sheet_13.zip › SR-02/33_2.tiff]

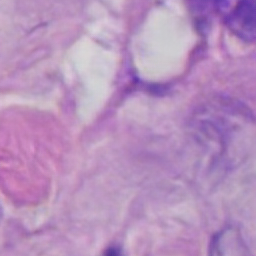

Supplement: Supplementary file 15 [file Data_Sheet_13.zip › SR-02/33_3.tiff]

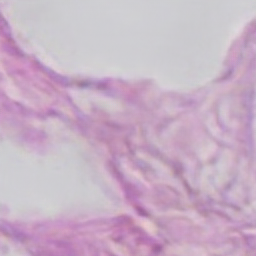

Supplement: Supplementary file 15 [file Data_Sheet_13.zip › SR-02/33_4.tiff]

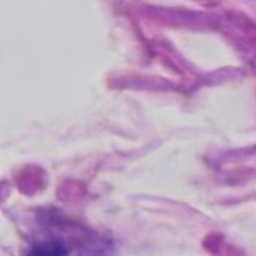

Supplement: Supplementary file 15 [file Data_Sheet_13.zip › SR-02/33_5.tiff]

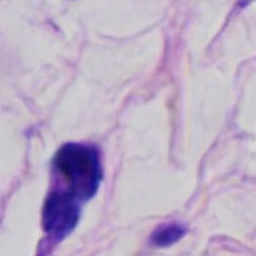

Supplement: Supplementary file 15 [file Data_Sheet_13.zip › SR-02/33_6.tiff]

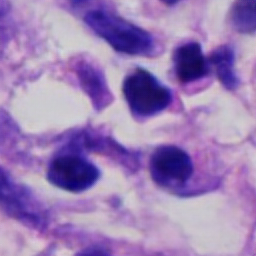

Supplement: Supplementary file 15 [file Data_Sheet_13.zip › SR-02/33_7.tiff]

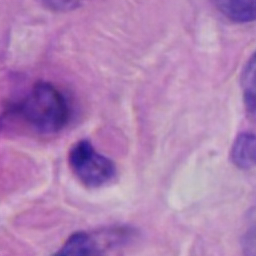

Supplement: Supplementary file 15 [file Data_Sheet_13.zip › SR-02/34_0.tiff]

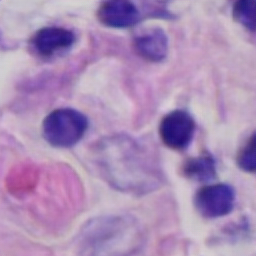

Supplement: Supplementary file 15 [file Data_Sheet_13.zip › SR-02/34_1.tiff]

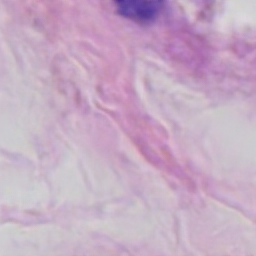

Supplement: Supplementary file 15 [file Data_Sheet_13.zip › SR-02/34_2.tiff]

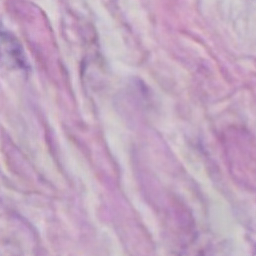

Supplement: Supplementary file 15 [file Data_Sheet_13.zip › SR-02/34_3.tiff]

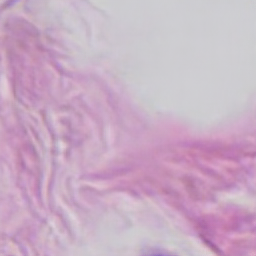

Supplement: Supplementary file 15 [file Data_Sheet_13.zip › SR-02/34_4.tiff]

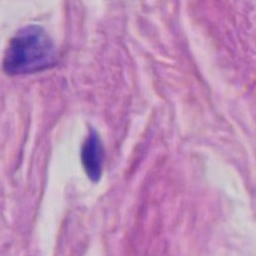

Supplement: Supplementary file 15 [file Data_Sheet_13.zip › SR-02/34_5.tiff]

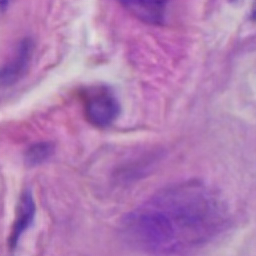

Supplement: Supplementary file 15 [file Data_Sheet_13.zip › SR-02/34_6.tiff]

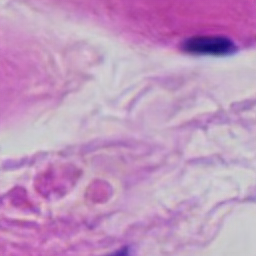

Supplement: Supplementary file 15 [file Data_Sheet_13.zip › SR-02/34_7.tiff]

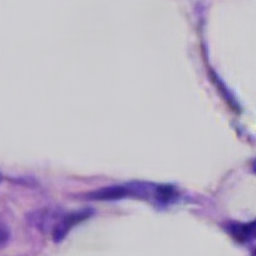

Supplement: Supplementary file 15 [file Data_Sheet_13.zip › SR-02/35_0.tiff]

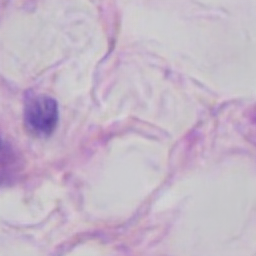

Supplement: Supplementary file 15 [file Data_Sheet_13.zip › SR-02/35_1.tiff]

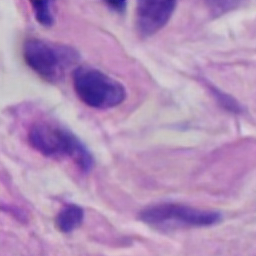

Supplement: Supplementary file 15 [file Data_Sheet_13.zip › SR-02/35_2.tiff]

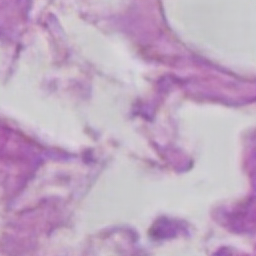

Supplement: Supplementary file 15 [file Data_Sheet_13.zip › SR-02/35_3.tiff]

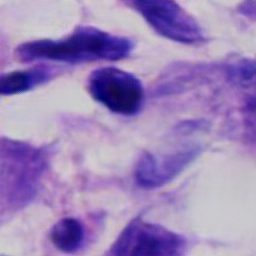

Supplement: Supplementary file 15 [file Data_Sheet_13.zip › SR-02/35_4.tiff]

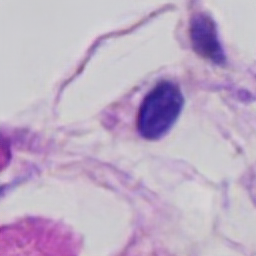

Supplement: Supplementary file 15 [file Data_Sheet_13.zip › SR-02/35_5.tiff]

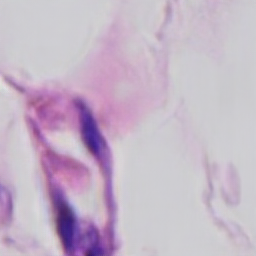

Supplement: Supplementary file 15 [file Data_Sheet_13.zip › SR-02/35_6.tiff]

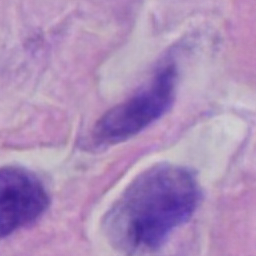

Supplement: Supplementary file 15 [file Data_Sheet_13.zip › SR-02/35_7.tiff]

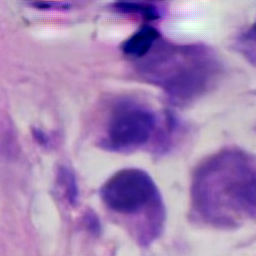

Supplement: Supplementary file 15 [file Data_Sheet_13.zip › SR-02/36_0.tiff]

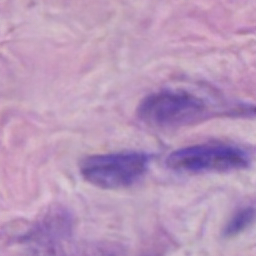

Supplement: Supplementary file 15 [file Data_Sheet_13.zip › SR-02/36_1.tiff]

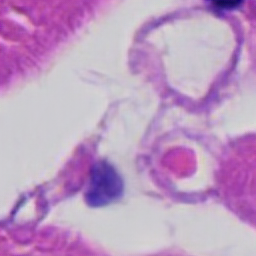

Supplement: Supplementary file 15 [file Data_Sheet_13.zip › SR-02/36_2.tiff]

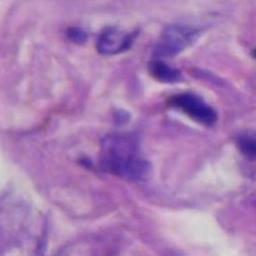

Supplement: Supplementary file 15 [file Data_Sheet_13.zip › SR-02/36_3.tiff]

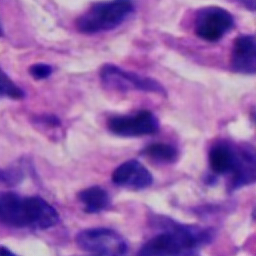

Supplement: Supplementary file 15 [file Data_Sheet_13.zip › SR-02/36_4.tiff]

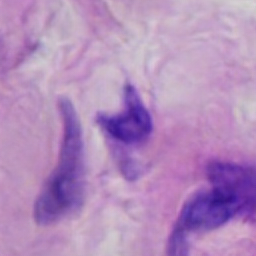

Supplement: Supplementary file 15 [file Data_Sheet_13.zip › SR-02/36_5.tiff]

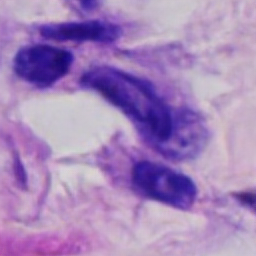

Supplement: Supplementary file 15 [file Data_Sheet_13.zip › SR-02/36_6.tiff]

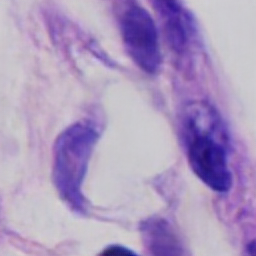

Supplement: Supplementary file 15 [file Data_Sheet_13.zip › SR-02/36_7.tiff]

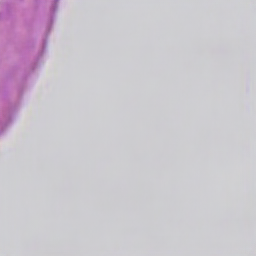

Supplement: Supplementary file 15 [file Data_Sheet_13.zip › SR-02/37_0.tiff]

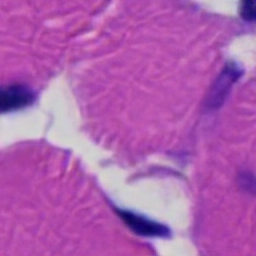

Supplement: Supplementary file 15 [file Data_Sheet_13.zip › SR-02/37_1.tiff]

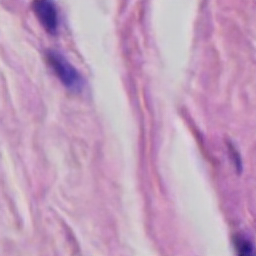

Supplement: Supplementary file 15 [file Data_Sheet_13.zip › SR-02/37_2.tiff]

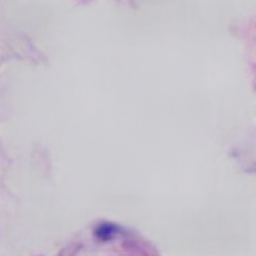

Supplement: Supplementary file 15 [file Data_Sheet_13.zip › SR-02/37_3.tiff]

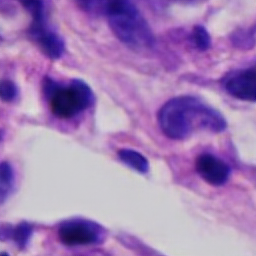

Supplement: Supplementary file 15 [file Data_Sheet_13.zip › SR-02/37_4.tiff]

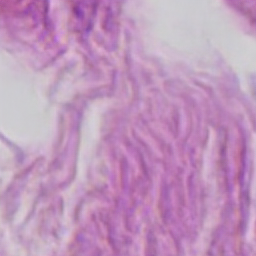

Supplement: Supplementary file 15 [file Data_Sheet_13.zip › SR-02/37_5.tiff]

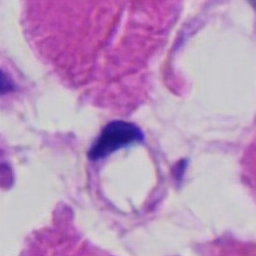

Supplement: Supplementary file 15 [file Data_Sheet_13.zip › SR-02/37_6.tiff]

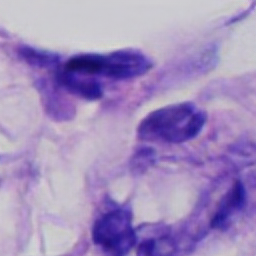

Supplement: Supplementary file 15 [file Data_Sheet_13.zip › SR-02/37_7.tiff]

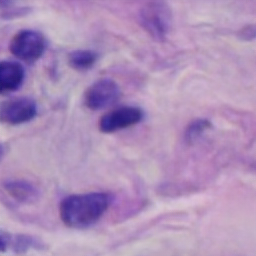

Supplement: Supplementary file 15 [file Data_Sheet_13.zip › SR-02/38_0.tiff]

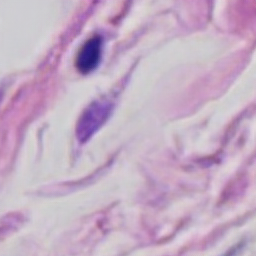

Supplement: Supplementary file 15 [file Data_Sheet_13.zip › SR-02/38_1.tiff]

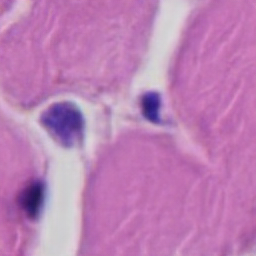

Supplement: Supplementary file 15 [file Data_Sheet_13.zip › SR-02/38_2.tiff]

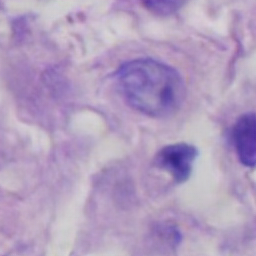

Supplement: Supplementary file 15 [file Data_Sheet_13.zip › SR-02/38_3.tiff]

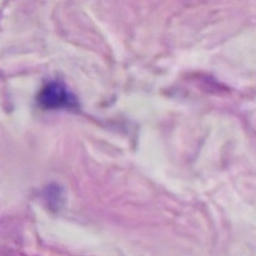

Supplement: Supplementary file 15 [file Data_Sheet_13.zip › SR-02/38_4.tiff]

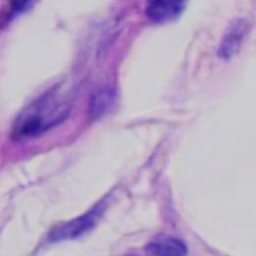

Supplement: Supplementary file 15 [file Data_Sheet_13.zip › SR-02/38_5.tiff]

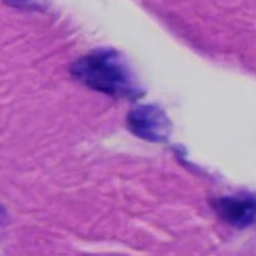

Supplement: Supplementary file 15 [file Data_Sheet_13.zip › SR-02/38_6.tiff]

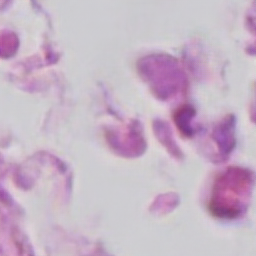

Supplement: Supplementary file 15 [file Data_Sheet_13.zip › SR-02/38_7.tiff]

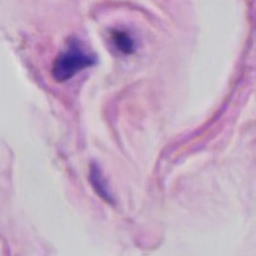

Supplement: Supplementary file 15 [file Data_Sheet_13.zip › SR-02/39_0.tiff]

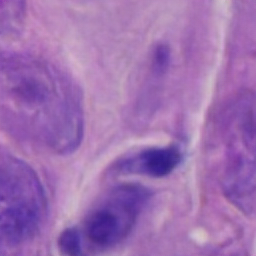

Supplement: Supplementary file 15 [file Data_Sheet_13.zip › SR-02/39_1.tiff]

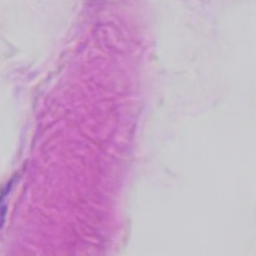

Supplement: Supplementary file 15 [file Data_Sheet_13.zip › SR-02/39_2.tiff]

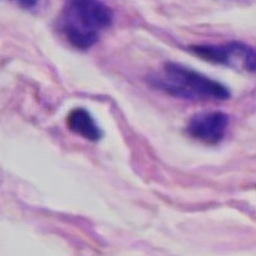

Supplement: Supplementary file 15 [file Data_Sheet_13.zip › SR-02/39_3.tiff]

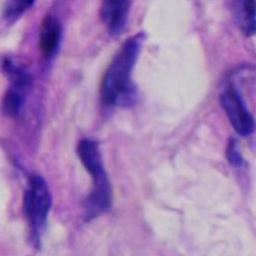

Supplement: Supplementary file 15 [file Data_Sheet_13.zip › SR-02/39_4.tiff]

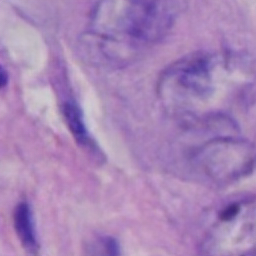

Supplement: Supplementary file 15 [file Data_Sheet_13.zip › SR-02/39_5.tiff]

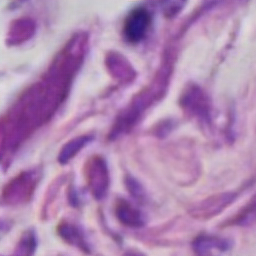

Supplement: Supplementary file 15 [file Data_Sheet_13.zip › SR-02/39_6.tiff]

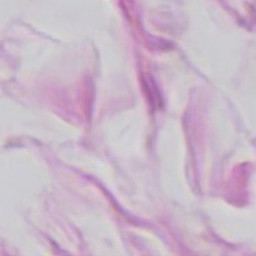

Supplement: Supplementary file 15 [file Data_Sheet_13.zip › SR-02/39_7.tiff]

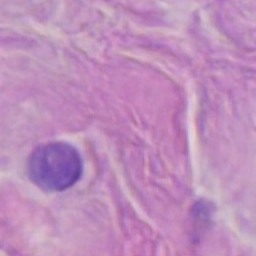

Supplement: Supplementary file 15 [file Data_Sheet_13.zip › SR-02/40_0.tiff]

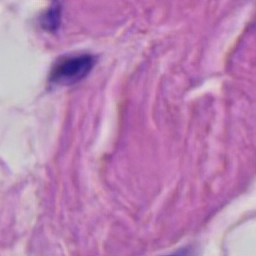

Supplement: Supplementary file 15 [file Data_Sheet_13.zip › SR-02/40_1.tiff]

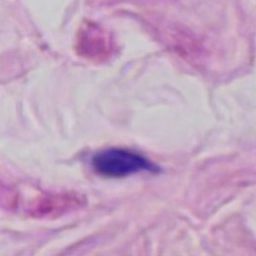

Supplement: Supplementary file 15 [file Data_Sheet_13.zip › SR-02/40_2.tiff]

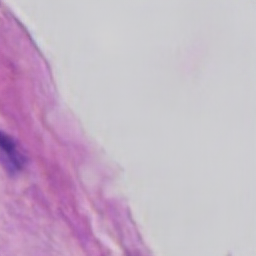

Supplement: Supplementary file 15 [file Data_Sheet_13.zip › SR-02/40_3.tiff]

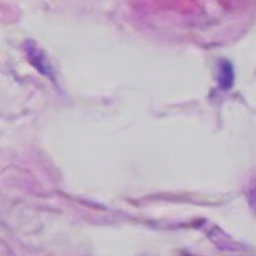

Supplement: Supplementary file 15 [file Data_Sheet_13.zip › SR-02/40_4.tiff]

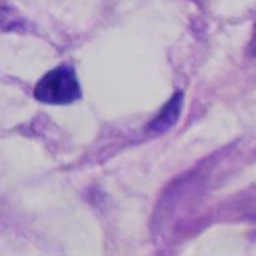

Supplement: Supplementary file 15 [file Data_Sheet_13.zip › SR-02/40_5.tiff]

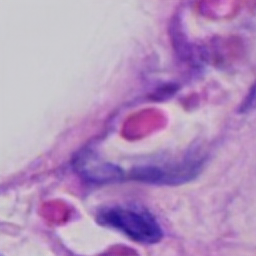

Supplement: Supplementary file 15 [file Data_Sheet_13.zip › SR-02/40_6.tiff]

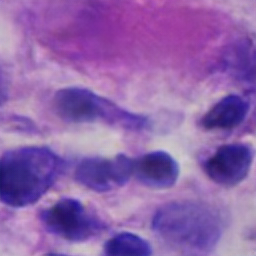

Supplement: Supplementary file 15 [file Data_Sheet_13.zip › SR-02/40_7.tiff]

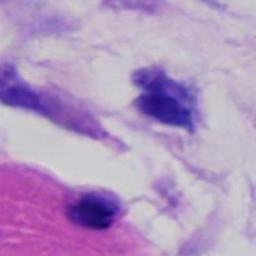

Supplement: Supplementary file 15 [file Data_Sheet_13.zip › SR-02/41_0.tiff]

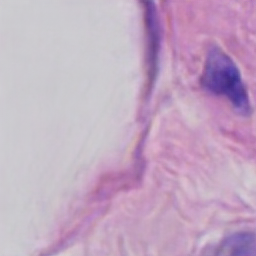

Supplement: Supplementary file 15 [file Data_Sheet_13.zip › SR-02/41_1.tiff]

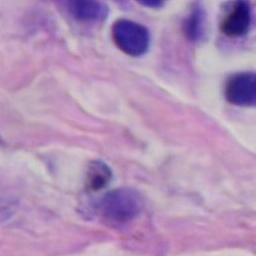

Supplement: Supplementary file 15 [file Data_Sheet_13.zip › SR-02/41_2.tiff]

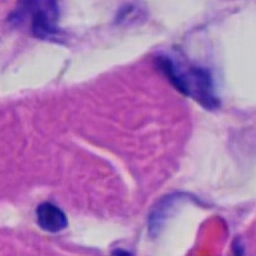

Supplement: Supplementary file 15 [file Data_Sheet_13.zip › SR-02/41_3.tiff]

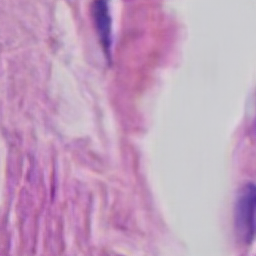

Supplement: Supplementary file 15 [file Data_Sheet_13.zip › SR-02/41_4.tiff]

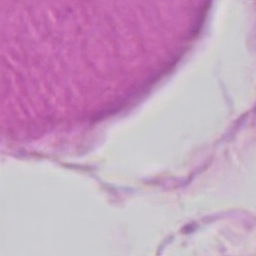

Supplement: Supplementary file 15 [file Data_Sheet_13.zip › SR-02/41_5.tiff]

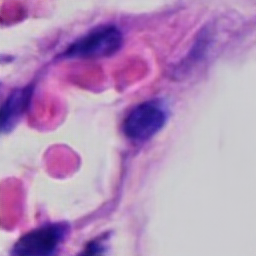

Supplement: Supplementary file 15 [file Data_Sheet_13.zip › SR-02/41_6.tiff]

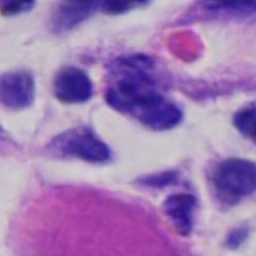

Supplement: Supplementary file 15 [file Data_Sheet_13.zip › SR-02/41_7.tiff]

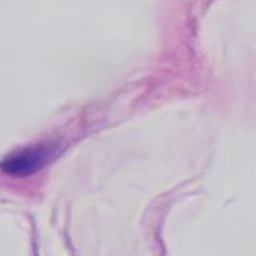

Supplement: Supplementary file 15 [file Data_Sheet_13.zip › SR-02/42_0.tiff]

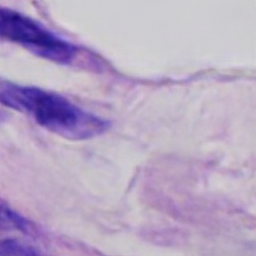

Supplement: Supplementary file 15 [file Data_Sheet_13.zip › SR-02/42_1.tiff]

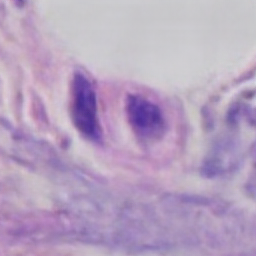

Supplement: Supplementary file 15 [file Data_Sheet_13.zip › SR-02/42_2.tiff]

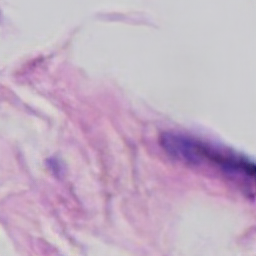

Supplement: Supplementary file 15 [file Data_Sheet_13.zip › SR-02/42_3.tiff]

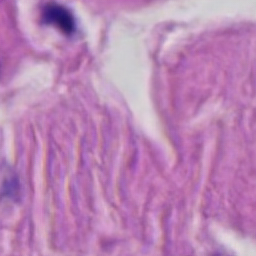

Supplement: Supplementary file 15 [file Data_Sheet_13.zip › SR-02/42_4.tiff]

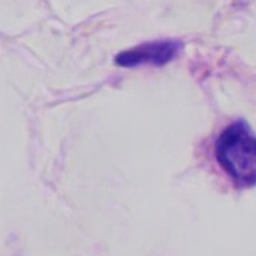

Supplement: Supplementary file 15 [file Data_Sheet_13.zip › SR-02/42_5.tiff]

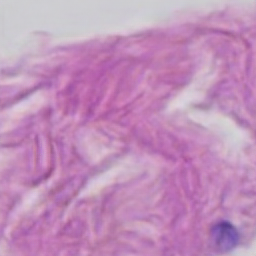

Supplement: Supplementary file 15 [file Data_Sheet_13.zip › SR-02/42_6.tiff]

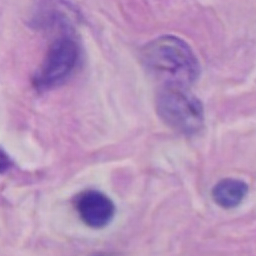

Supplement: Supplementary file 15 [file Data_Sheet_13.zip › SR-02/42_7.tiff]

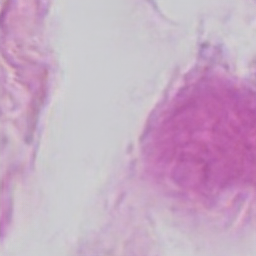

Supplement: Supplementary file 15 [file Data_Sheet_13.zip › SR-02/43_0.tiff]

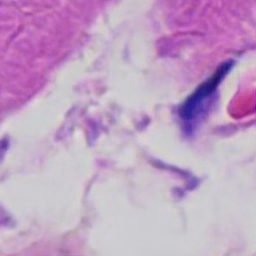

Supplement: Supplementary file 15 [file Data_Sheet_13.zip › SR-02/43_1.tiff]

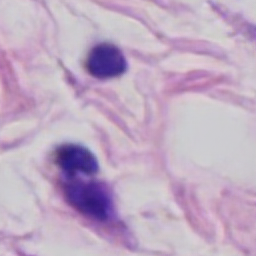

Supplement: Supplementary file 15 [file Data_Sheet_13.zip › SR-02/43_2.tiff]

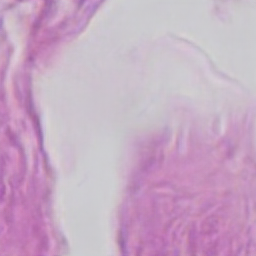

Supplement: Supplementary file 15 [file Data_Sheet_13.zip › SR-02/43_3.tiff]

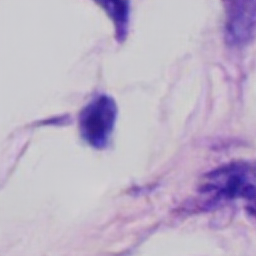

Supplement: Supplementary file 15 [file Data_Sheet_13.zip › SR-02/43_4.tiff]

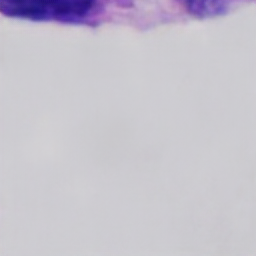

Supplement: Supplementary file 15 [file Data_Sheet_13.zip › SR-02/43_5.tiff]

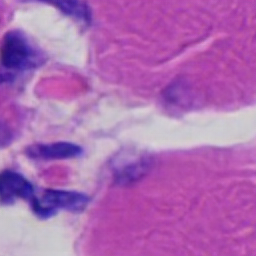

Supplement: Supplementary file 15 [file Data_Sheet_13.zip › SR-02/43_6.tiff]

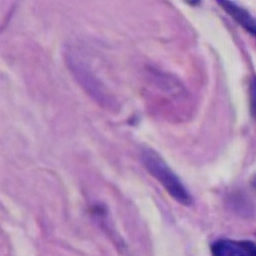

Supplement: Supplementary file 15 [file Data_Sheet_13.zip › SR-02/43_7.tiff]

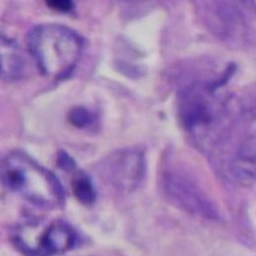

Supplement: Supplementary file 15 [file Data_Sheet_13.zip › SR-02/44_0.tiff]

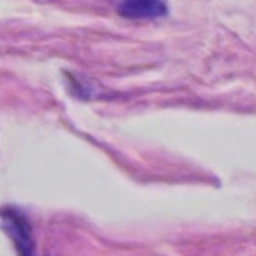

Supplement: Supplementary file 15 [file Data_Sheet_13.zip › SR-02/44_1.tiff]

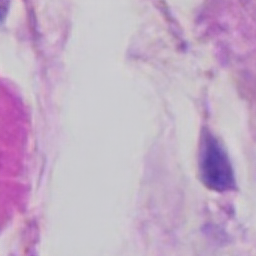

Supplement: Supplementary file 15 [file Data_Sheet_13.zip › SR-02/44_2.tiff]

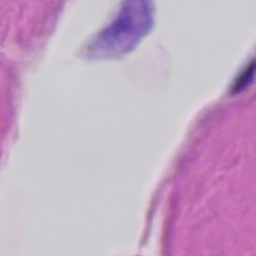

Supplement: Supplementary file 15 [file Data_Sheet_13.zip › SR-02/44_3.tiff]

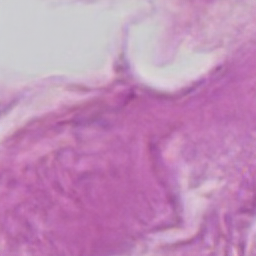

Supplement: Supplementary file 15 [file Data_Sheet_13.zip › SR-02/44_4.tiff]

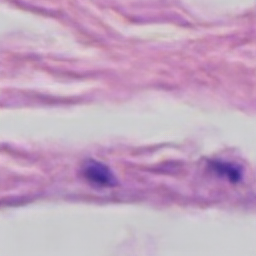

Supplement: Supplementary file 15 [file Data_Sheet_13.zip › SR-02/44_5.tiff]

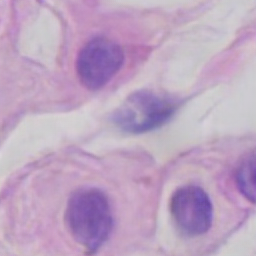

Supplement: Supplementary file 15 [file Data_Sheet_13.zip › SR-02/44_6.tiff]

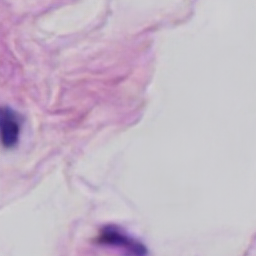

Supplement: Supplementary file 15 [file Data_Sheet_13.zip › SR-02/44_7.tiff]

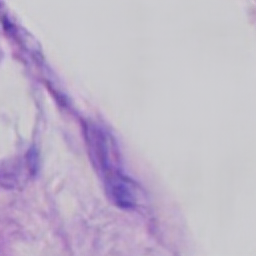

Supplement: Supplementary file 15 [file Data_Sheet_13.zip › SR-02/45_0.tiff]

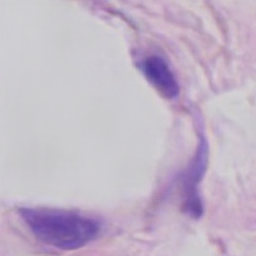

Supplement: Supplementary file 15 [file Data_Sheet_13.zip › SR-02/45_1.tiff]

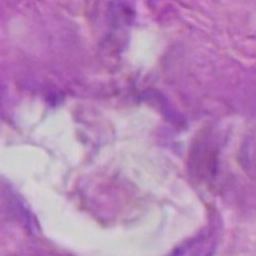

Supplement: Supplementary file 15 [file Data_Sheet_13.zip › SR-02/45_2.tiff]

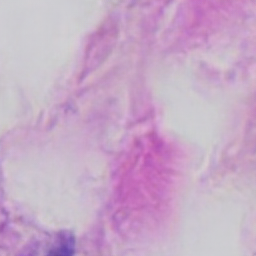

Supplement: Supplementary file 15 [file Data_Sheet_13.zip › SR-02/45_3.tiff]
