# Supplementary figures and images for: Feasibility study of single-image super-resolution scanning system based on deep learning for pathological diagnosis of oral epithelial dysplasia (part 21 of 21)
Source: Front Med (Lausanne). 2025 Mar 12;12:1550512. doi: 10.3389/fmed.2025.1550512 (PMC11936936; doi:10.3389/fmed.2025.1550512)

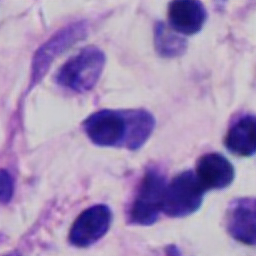

Supplement: Supplementary file 16 [file Data_Sheet_14.zip › SR-03/68_0.tiff]

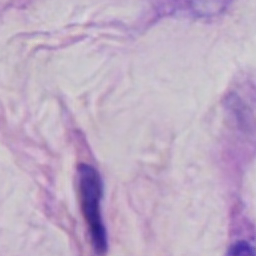

Supplement: Supplementary file 16 [file Data_Sheet_14.zip › SR-03/68_1.tiff]

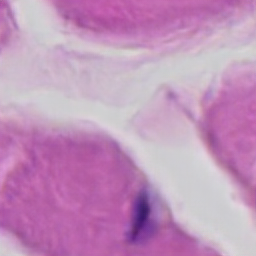

Supplement: Supplementary file 16 [file Data_Sheet_14.zip › SR-03/68_2.tiff]

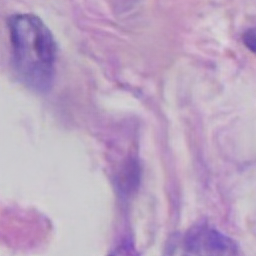

Supplement: Supplementary file 16 [file Data_Sheet_14.zip › SR-03/68_3.tiff]

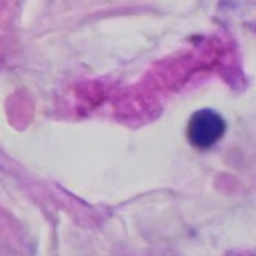

Supplement: Supplementary file 16 [file Data_Sheet_14.zip › SR-03/68_4.tiff]

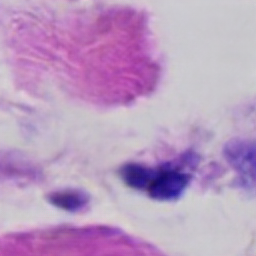

Supplement: Supplementary file 16 [file Data_Sheet_14.zip › SR-03/68_5.tiff]

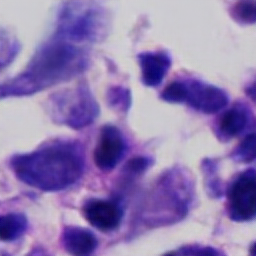

Supplement: Supplementary file 16 [file Data_Sheet_14.zip › SR-03/68_6.tiff]

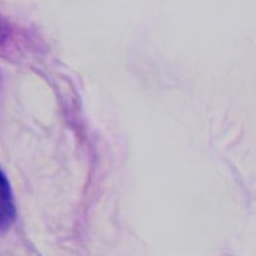

Supplement: Supplementary file 16 [file Data_Sheet_14.zip › SR-03/68_7.tiff]

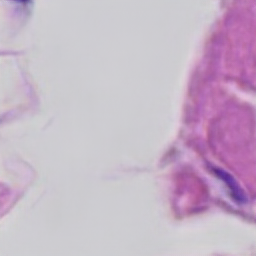

Supplement: Supplementary file 16 [file Data_Sheet_14.zip › SR-03/69_0.tiff]

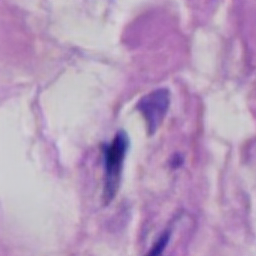

Supplement: Supplementary file 16 [file Data_Sheet_14.zip › SR-03/69_1.tiff]

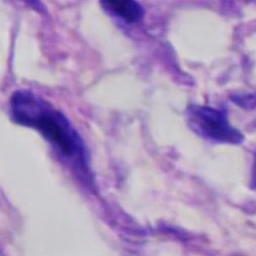

Supplement: Supplementary file 16 [file Data_Sheet_14.zip › SR-03/69_2.tiff]

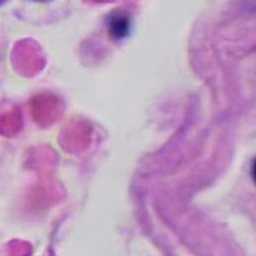

Supplement: Supplementary file 16 [file Data_Sheet_14.zip › SR-03/69_3.tiff]

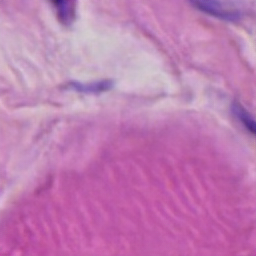

Supplement: Supplementary file 16 [file Data_Sheet_14.zip › SR-03/69_4.tiff]

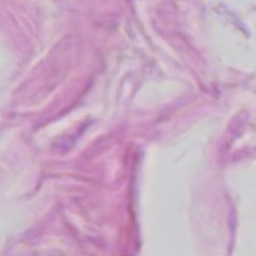

Supplement: Supplementary file 16 [file Data_Sheet_14.zip › SR-03/69_5.tiff]

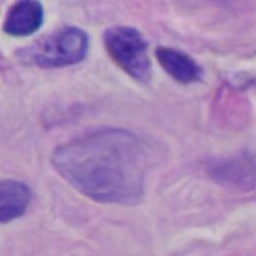

Supplement: Supplementary file 16 [file Data_Sheet_14.zip › SR-03/69_6.tiff]

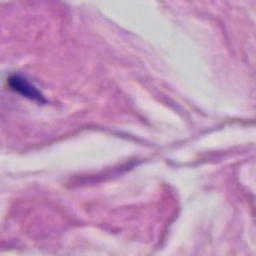

Supplement: Supplementary file 16 [file Data_Sheet_14.zip › SR-03/69_7.tiff]

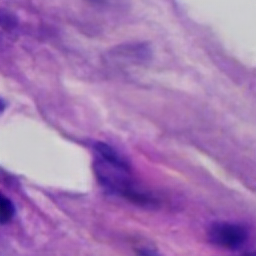

Supplement: Supplementary file 16 [file Data_Sheet_14.zip › SR-03/70_0.tiff]

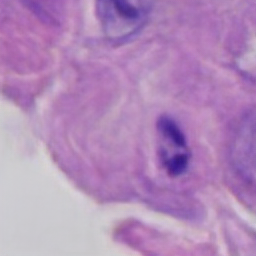

Supplement: Supplementary file 16 [file Data_Sheet_14.zip › SR-03/70_1.tiff]

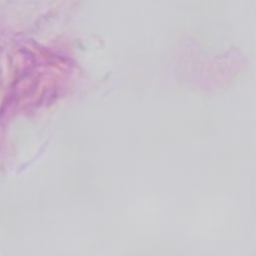

Supplement: Supplementary file 16 [file Data_Sheet_14.zip › SR-03/70_2.tiff]

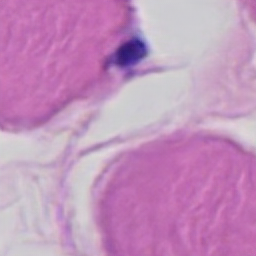

Supplement: Supplementary file 16 [file Data_Sheet_14.zip › SR-03/70_3.tiff]

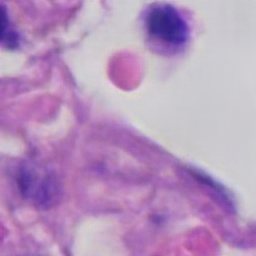

Supplement: Supplementary file 16 [file Data_Sheet_14.zip › SR-03/70_4.tiff]

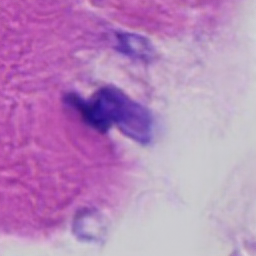

Supplement: Supplementary file 16 [file Data_Sheet_14.zip › SR-03/70_5.tiff]

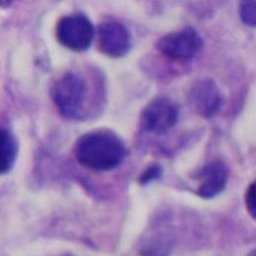

Supplement: Supplementary file 16 [file Data_Sheet_14.zip › SR-03/70_6.tiff]

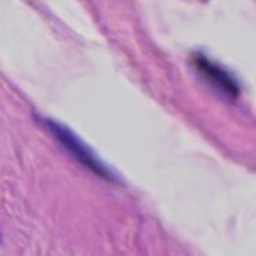

Supplement: Supplementary file 16 [file Data_Sheet_14.zip › SR-03/70_7.tiff]

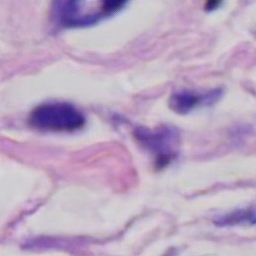

Supplement: Supplementary file 16 [file Data_Sheet_14.zip › SR-03/71_0.tiff]

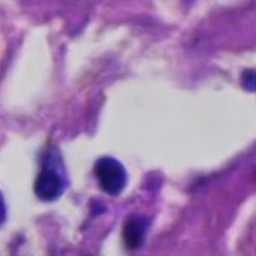

Supplement: Supplementary file 16 [file Data_Sheet_14.zip › SR-03/71_1.tiff]

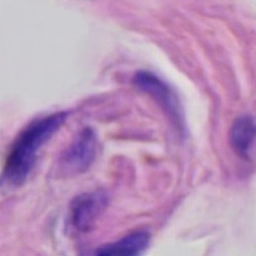

Supplement: Supplementary file 16 [file Data_Sheet_14.zip › SR-03/71_2.tiff]

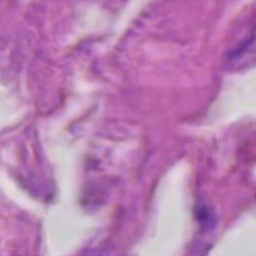

Supplement: Supplementary file 16 [file Data_Sheet_14.zip › SR-03/71_3.tiff]

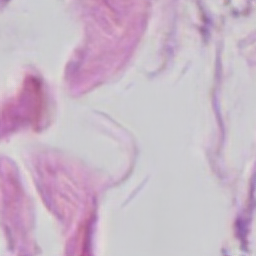

Supplement: Supplementary file 16 [file Data_Sheet_14.zip › SR-03/71_4.tiff]

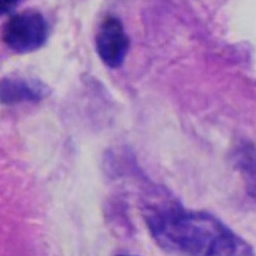

Supplement: Supplementary file 16 [file Data_Sheet_14.zip › SR-03/71_5.tiff]

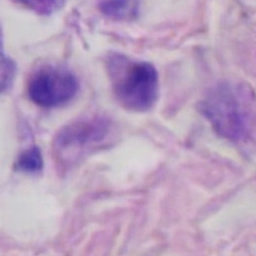

Supplement: Supplementary file 16 [file Data_Sheet_14.zip › SR-03/71_6.tiff]

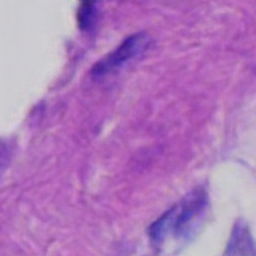

Supplement: Supplementary file 16 [file Data_Sheet_14.zip › SR-03/71_7.tiff]

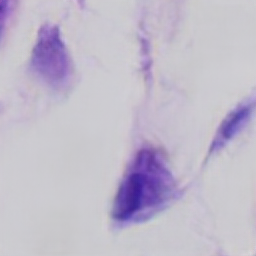

Supplement: Supplementary file 16 [file Data_Sheet_14.zip › SR-03/72_0.tiff]

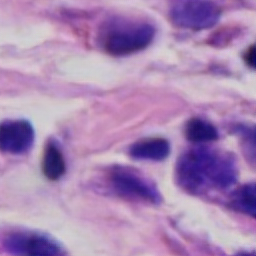

Supplement: Supplementary file 16 [file Data_Sheet_14.zip › SR-03/72_1.tiff]

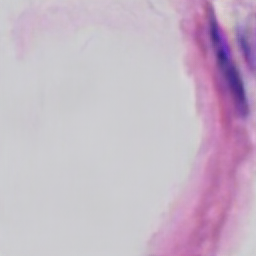

Supplement: Supplementary file 16 [file Data_Sheet_14.zip › SR-03/72_2.tiff]

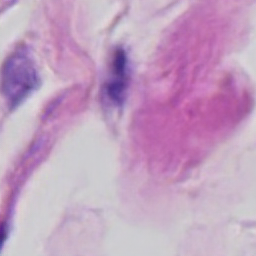

Supplement: Supplementary file 16 [file Data_Sheet_14.zip › SR-03/72_3.tiff]

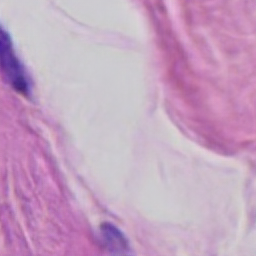

Supplement: Supplementary file 16 [file Data_Sheet_14.zip › SR-03/72_4.tiff]

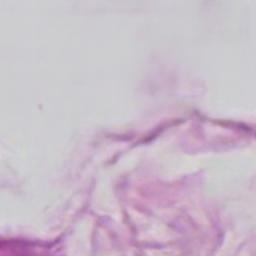

Supplement: Supplementary file 16 [file Data_Sheet_14.zip › SR-03/72_5.tiff]

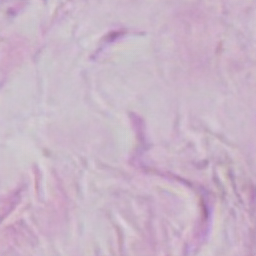

Supplement: Supplementary file 16 [file Data_Sheet_14.zip › SR-03/72_6.tiff]

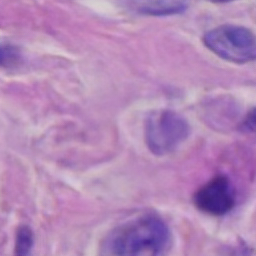

Supplement: Supplementary file 16 [file Data_Sheet_14.zip › SR-03/72_7.tiff]

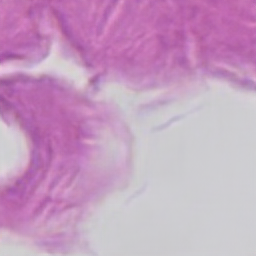

Supplement: Supplementary file 16 [file Data_Sheet_14.zip › SR-03/73_0.tiff]

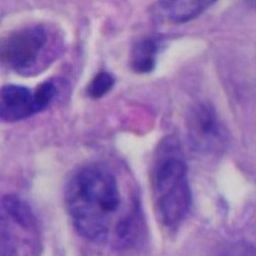

Supplement: Supplementary file 16 [file Data_Sheet_14.zip › SR-03/73_1.tiff]

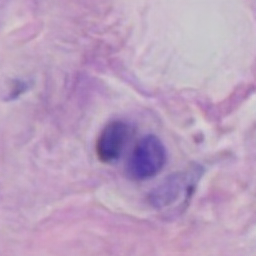

Supplement: Supplementary file 16 [file Data_Sheet_14.zip › SR-03/73_2.tiff]

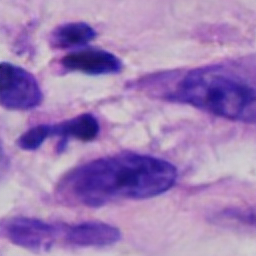

Supplement: Supplementary file 16 [file Data_Sheet_14.zip › SR-03/73_3.tiff]

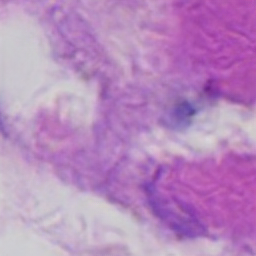

Supplement: Supplementary file 16 [file Data_Sheet_14.zip › SR-03/73_4.tiff]

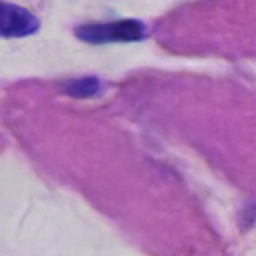

Supplement: Supplementary file 16 [file Data_Sheet_14.zip › SR-03/73_5.tiff]

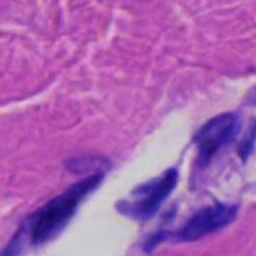

Supplement: Supplementary file 16 [file Data_Sheet_14.zip › SR-03/73_6.tiff]

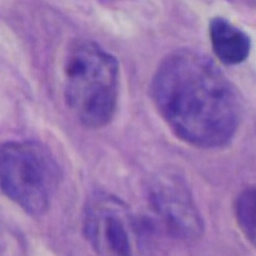

Supplement: Supplementary file 16 [file Data_Sheet_14.zip › SR-03/73_7.tiff]

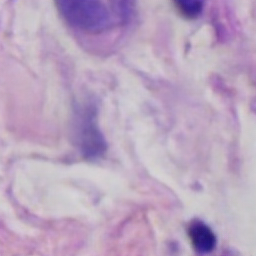

Supplement: Supplementary file 16 [file Data_Sheet_14.zip › SR-03/74_0.tiff]

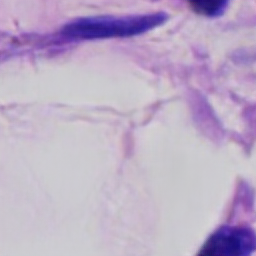

Supplement: Supplementary file 16 [file Data_Sheet_14.zip › SR-03/74_1.tiff]

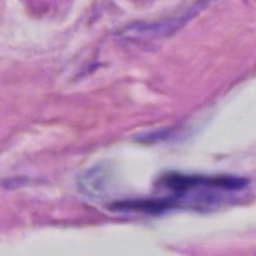

Supplement: Supplementary file 16 [file Data_Sheet_14.zip › SR-03/74_2.tiff]

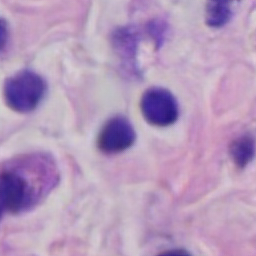

Supplement: Supplementary file 16 [file Data_Sheet_14.zip › SR-03/74_3.tiff]

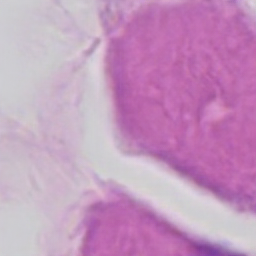

Supplement: Supplementary file 16 [file Data_Sheet_14.zip › SR-03/74_4.tiff]

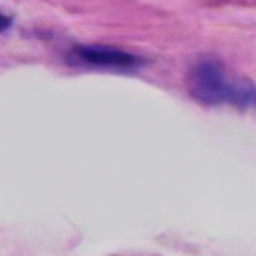

Supplement: Supplementary file 16 [file Data_Sheet_14.zip › SR-03/74_5.tiff]

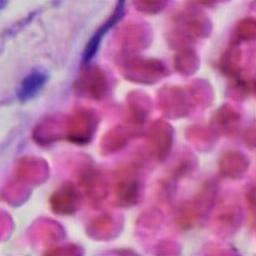

Supplement: Supplementary file 16 [file Data_Sheet_14.zip › SR-03/74_6.tiff]

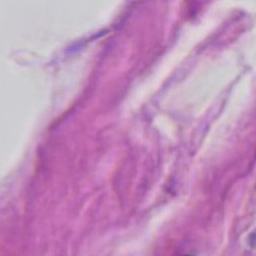

Supplement: Supplementary file 16 [file Data_Sheet_14.zip › SR-03/74_7.tiff]

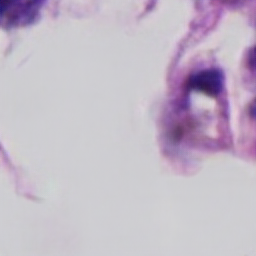

Supplement: Supplementary file 16 [file Data_Sheet_14.zip › SR-03/75_0.tiff]

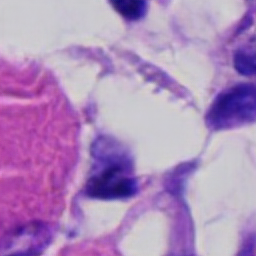

Supplement: Supplementary file 16 [file Data_Sheet_14.zip › SR-03/75_1.tiff]

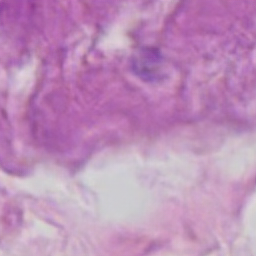

Supplement: Supplementary file 16 [file Data_Sheet_14.zip › SR-03/75_2.tiff]

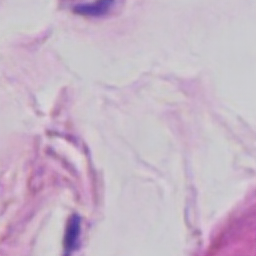

Supplement: Supplementary file 16 [file Data_Sheet_14.zip › SR-03/75_3.tiff]

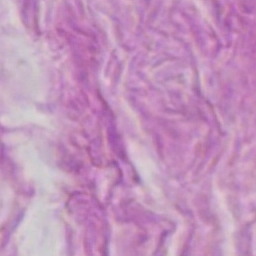

Supplement: Supplementary file 16 [file Data_Sheet_14.zip › SR-03/75_4.tiff]

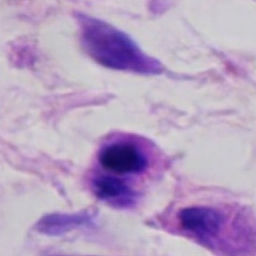

Supplement: Supplementary file 16 [file Data_Sheet_14.zip › SR-03/75_5.tiff]

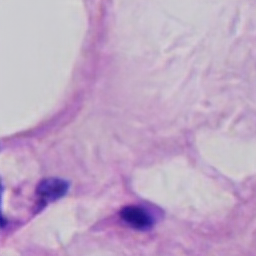

Supplement: Supplementary file 16 [file Data_Sheet_14.zip › SR-03/75_6.tiff]

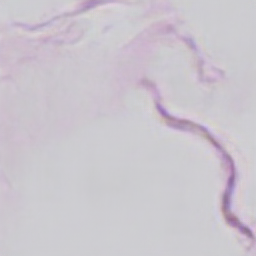

Supplement: Supplementary file 16 [file Data_Sheet_14.zip › SR-03/75_7.tiff]

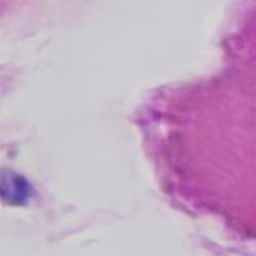

Supplement: Supplementary file 16 [file Data_Sheet_14.zip › SR-03/76_0.tiff]

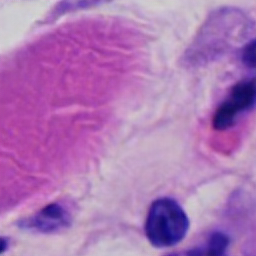

Supplement: Supplementary file 16 [file Data_Sheet_14.zip › SR-03/76_1.tiff]

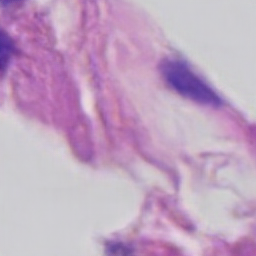

Supplement: Supplementary file 16 [file Data_Sheet_14.zip › SR-03/76_2.tiff]

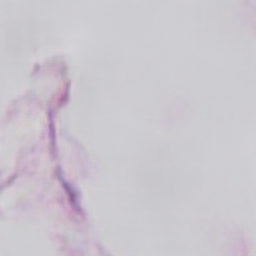

Supplement: Supplementary file 16 [file Data_Sheet_14.zip › SR-03/76_3.tiff]

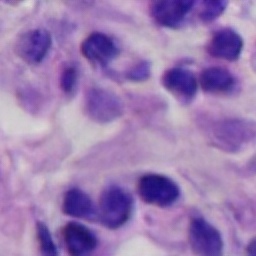

Supplement: Supplementary file 16 [file Data_Sheet_14.zip › SR-03/76_4.tiff]

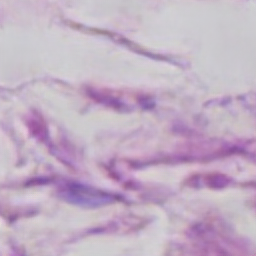

Supplement: Supplementary file 16 [file Data_Sheet_14.zip › SR-03/76_5.tiff]

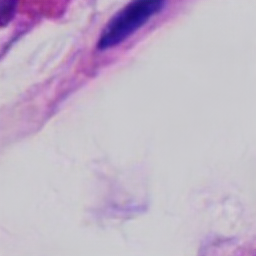

Supplement: Supplementary file 16 [file Data_Sheet_14.zip › SR-03/76_6.tiff]

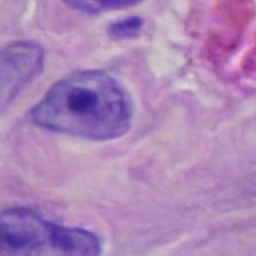

Supplement: Supplementary file 16 [file Data_Sheet_14.zip › SR-03/76_7.tiff]

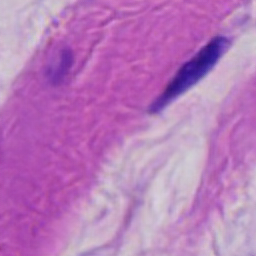

Supplement: Supplementary file 16 [file Data_Sheet_14.zip › SR-03/77_0.tiff]

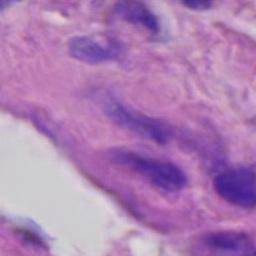

Supplement: Supplementary file 16 [file Data_Sheet_14.zip › SR-03/77_1.tiff]

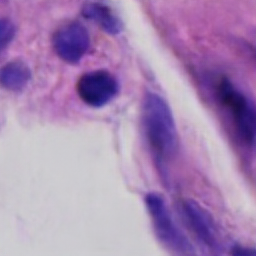

Supplement: Supplementary file 16 [file Data_Sheet_14.zip › SR-03/77_2.tiff]

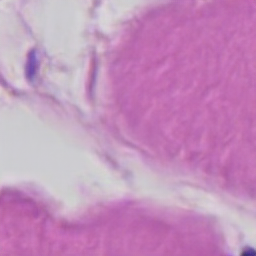

Supplement: Supplementary file 16 [file Data_Sheet_14.zip › SR-03/77_3.tiff]

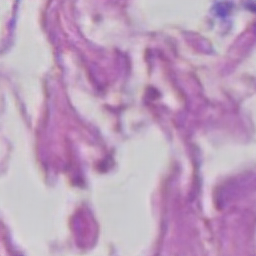

Supplement: Supplementary file 16 [file Data_Sheet_14.zip › SR-03/77_4.tiff]

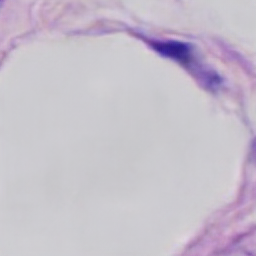

Supplement: Supplementary file 16 [file Data_Sheet_14.zip › SR-03/77_5.tiff]

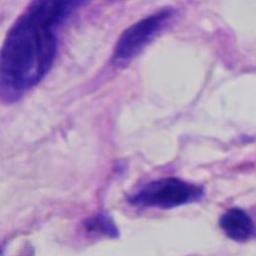

Supplement: Supplementary file 16 [file Data_Sheet_14.zip › SR-03/77_6.tiff]

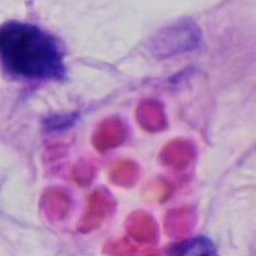

Supplement: Supplementary file 16 [file Data_Sheet_14.zip › SR-03/77_7.tiff]

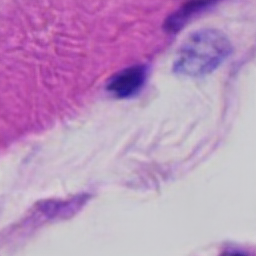

Supplement: Supplementary file 16 [file Data_Sheet_14.zip › SR-03/78_0.tiff]

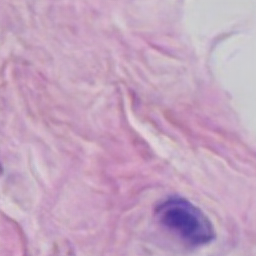

Supplement: Supplementary file 16 [file Data_Sheet_14.zip › SR-03/78_1.tiff]

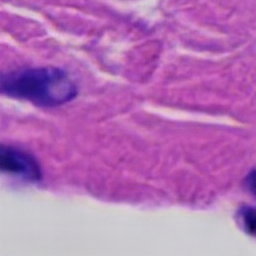

Supplement: Supplementary file 16 [file Data_Sheet_14.zip › SR-03/78_2.tiff]

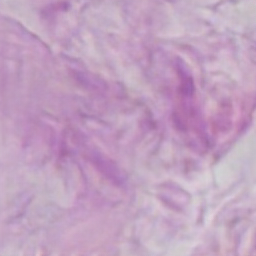

Supplement: Supplementary file 16 [file Data_Sheet_14.zip › SR-03/78_3.tiff]

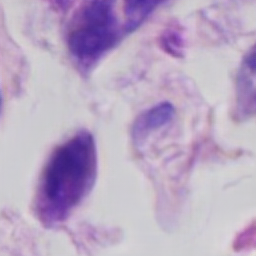

Supplement: Supplementary file 16 [file Data_Sheet_14.zip › SR-03/78_4.tiff]

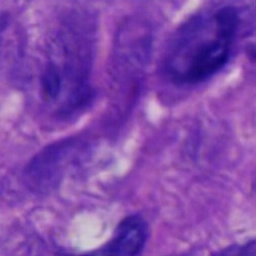

Supplement: Supplementary file 16 [file Data_Sheet_14.zip › SR-03/78_5.tiff]

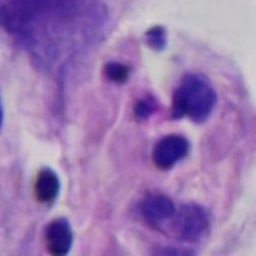

Supplement: Supplementary file 16 [file Data_Sheet_14.zip › SR-03/78_6.tiff]

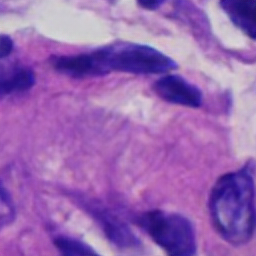

Supplement: Supplementary file 16 [file Data_Sheet_14.zip › SR-03/78_7.tiff]

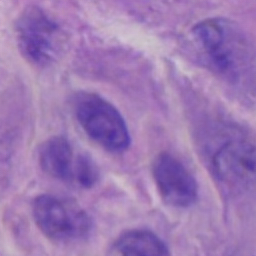

Supplement: Supplementary file 16 [file Data_Sheet_14.zip › SR-03/79_0.tiff]

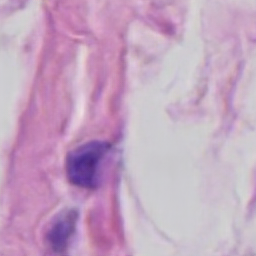

Supplement: Supplementary file 16 [file Data_Sheet_14.zip › SR-03/79_1.tiff]

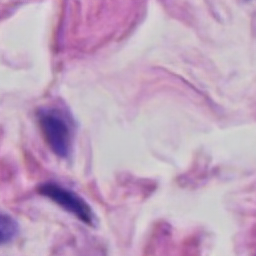

Supplement: Supplementary file 16 [file Data_Sheet_14.zip › SR-03/79_2.tiff]

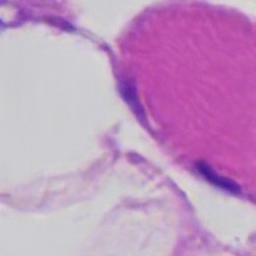

Supplement: Supplementary file 16 [file Data_Sheet_14.zip › SR-03/79_3.tiff]

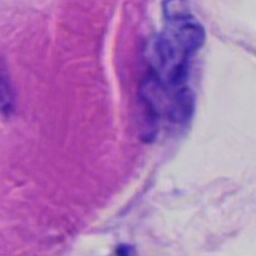

Supplement: Supplementary file 16 [file Data_Sheet_14.zip › SR-03/79_4.tiff]

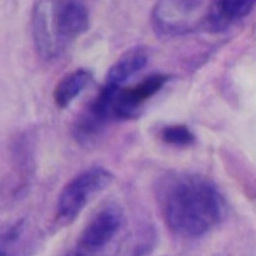

Supplement: Supplementary file 16 [file Data_Sheet_14.zip › SR-03/79_5.tiff]

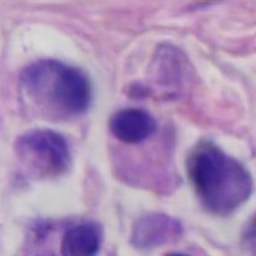

Supplement: Supplementary file 16 [file Data_Sheet_14.zip › SR-03/79_6.tiff]

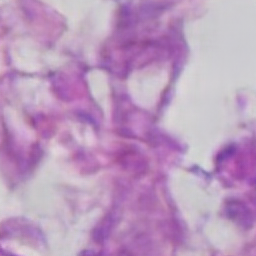

Supplement: Supplementary file 16 [file Data_Sheet_14.zip › SR-03/79_7.tiff]
